# Stable Isotope Phosphate Labelling of Diverse Metabolites is Enabled by a Family of ^18^O‐Phosphoramidites**

**DOI:** 10.1002/anie.202112457

**Published:** 2021-11-23

**Authors:** Thomas M. Haas, Stephan Mundinger, Danye Qiu, Nikolaus Jork, Kevin Ritter, Tobias Dürr‐Mayer, Alexander Ripp, Adolfo Saiardi, Gabriel Schaaf, Henning J. Jessen

**Affiliations:** ^1^ Institute of Organic Chemistry Albert-Ludwigs-Universität Freiburg Albertstrasse 21 79102 Freiburg im Breisgau Germany; ^2^ CIBSS—The Center for Biological Signaling Studies &, Spemann Graduate School of Biology and Medicine (SGBM) Albert-Ludwigs-Universität Freiburg Germany; ^3^ Medical Research Council, Laboratory for molecular Cell Biology University College London UK; ^4^ INRES—Institut für Nutzpflanzenwissenschaften und Ressourcenschutz Universität Bonn Karlrobert-Kreiten-Strasse 13 53115 Bonn Germany

**Keywords:** capillary electrophoresis, mass spectrometry, nucleotides, phosphorylation, stable isotope labelling

## Abstract

Stable isotope labelling is state‐of‐the‐art in quantitative mass spectrometry, yet often accessing the required standards is cumbersome and very expensive. Here, a unifying synthetic concept for ^18^O‐labelled phosphates is presented, based on a family of modified ^18^O_2_‐phosphoramidite reagents. This toolbox offers access to major classes of biologically highly relevant phosphorylated metabolites as their isotopologues including nucleotides, inositol phosphates, ‐pyrophosphates, and inorganic polyphosphates. ^18^O‐enrichment ratios >95 % and good yields are obtained consistently in gram‐scale reactions, while enabling late‐stage labelling. We demonstrate the utility of the ^18^O‐labelled inositol phosphates and pyrophosphates by assignment of these metabolites from different biological matrices. We demonstrate that phosphate neutral loss is negligible in an analytical setup employing capillary electrophoresis electrospray ionisation triple quadrupole mass spectrometry.

## Graphical abstract

A family of ^18^O_2_‐phosphoramidites facilitates synthetic access on gram‐scale to various isotopically pure ^18^O‐labelled phosphate products, like nucleotides, inositol phosphates, polyphosphates, and DNA. The utility of these ^18^O‐natural products is underlined in the assignment of various metabolites from biological matrices using capillary electrophoresis electrospray ionisation triple quadrupole mass spectrometry.

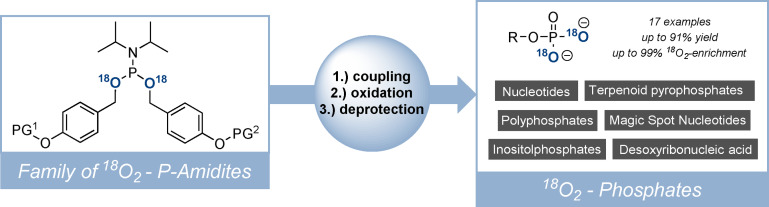

## Introduction

Isotopologues are molecular entities that differ only in isotopic composition.^[1]^ Their chemical properties are almost identical, yet they can be readily distinguished by several analytical methods. This ambivalence has been the basis for many applications in chemistry, biology and medicine for decades.[^2^, ^3^, ^4^, ^5^, ^6^, ^7^] The synthesis of isotopologues is called isotope labelling and can be categorised into stable isotope labelling (SIL) and radiolabelling.^[8]^

The phosphate group is among the most important functional groups in organisms, playing a pivotal role in energy transfer, enzyme activation and genetic information storage.^[9]^ For more than 50 years, isotope labelling of phosphate groups has contributed to our understanding of phosphate reactivity and function. SIL in the context of phosphates focuses on oxygen isotopes, since the P‐isotopes ^32^P and ^33^P are beta‐emitting radionuclides (*t*_1/2, 32P_=14 d, *t*_1/2, 33P_=25 d).^[10]^ Potentially, both ^17^O and ^18^O are suitable isotopes for phosphate SIL. The higher natural abundance of ^18^O and its mass‐shift of *M*+2 render this isotope more useful for mass‐spectrometry‐based applications, while important applications for the NMR‐active ^17^O nucleus exist.^[11]^

**Figure 1:**
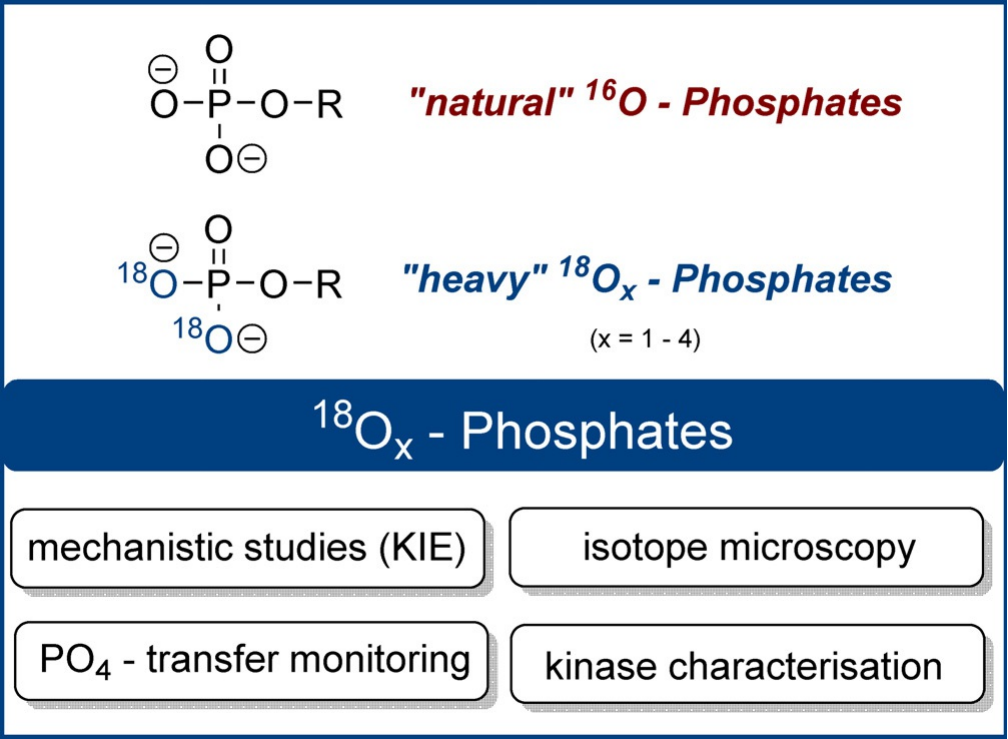
Structures of natural ^16^O_4_‐phosphates and labelled ^18^O_x_‐phosphates. ^18^O_2_‐phosphate is shown as an example (KIE: kinetic isotope effect).

Since the 1970s ^18^O‐phosphates have been used as probes to clarify questions in chemistry and biology (Figure 1). ^18^O‐phosphates were indispensable, for example, in the elucidation of structure, reactivity, and reaction mechanisms involving phosphate esters and anhydrides. Already in 1977, Gorenstein et al. elucidated metaphosphate‐involving mechanisms in phosphate ester hydrolysis reactions by exploiting ^18^O‐kinetic isotope effects.^[12]^ In 1988, Takeuchi et al. used ^18^O‐ATP to determine Mg^2+^ coordination sites by Raman spectroscopy.^[13]^ In 1990, Cleland described how ^18^O‐labelled phosphate esters could be used to study phosphate transesterification mechanisms. He emphasised that “the use of [secondary] ^18^O […] isotope effects has proved a powerful tool for studying transition state structure in phosphoryl transfer reactions.”^[14]^ Later, similar approaches to clarify the catalytic mechanism of RNase A were developed.^[15]^ Additionally, Lee et al. investigated ^18^O‐kinetic leaving group effects in 2011 to gain mechanistic evidence for an SN_i_‐type reaction of a trehalose‐6‐phosphate synthase.^[16]^ Another application of ^18^O‐phosphate esters was presented by Hamasaki et al. in 2013 by synthesising ^18^O‐labelled RNA as an alternative to fluorescently labelled RNA. The ^18^O‐RNA was then visualised by isotope microscopy in human cells.^[17]^

Phosphate groups, usually from P‐anhydrides (e.g. ATP), can be transferred to nucleophiles in vivo. Detailed studies into phosphate transfer events are essential for our understanding of complex cellular processes.^[18]^ In this context, ^18^O‐phosphates proved particularly powerful. In 1976, Midlefort and Rose used an ^18^O‐isotope scrambling method to clarify transient dephosphorylation reactions of ATP catalysed by glutamine synthetase.^[19]^ Phelan et al. characterised adenylation efficiency of non‐ribosomal peptide synthetases by measuring pyrophosphate exchange of ^18^O_4_‐ATP.^[20]^ Scian et al. investigated the mechanism of ^18^O_4_‐ATP hydrolysis catalysed by human P‐glycoprotein.^[21]^ Boyer elucidated ATPase's mechanism by analysing ^18^O‐exchange between ^18^O_4_‐ATP, P_i_ and water under uncoupling conditions. In his Nobel lecture he stated: “The use of the ^18^O‐exchange measurements to study the process provided a crucial insight.”^[22]^

γ‐^18^O_*x*_‐ATP phosphorylation analysis also became a key tool in kinase characterisation, because the ^18^O‐approach is non‐radioactive and does not require fluorescent substrates.

Zhou et al. used ^18^O_4_‐ATP and MS/MS to identify the phosphorylation sites within substrates of a human tyrosine/serine kinase.^[23]^ Analogously, Sulbaran et al. characterised kinase phosphorylation sites on myosin regulatory light chains.^[24]^ Fu et al. reported a kinase assay based on ^18^O_4_‐ATP, to determine effectiveness and specificity of kinase inhibitors.^[25]^ Molden et al. demonstrated the application of γ‐^18^O_4_‐ATP in nucleo to follow protein phosphorylation rates.^[26]^ Furthermore, several proteomics‐based assays with ^18^O_*x*_‐ATP (*x*=2–4) were developed, identifying substrates of specific kinases of interest.[^27^, ^28^, ^29^, ^30^, ^31^]

These various applications underline the tremendous potential of ^18^O‐labelled phosphates to serve as tool compounds for addressing chemical and biological questions. Notably, most methods rely on synthetically well‐studied ^18^O_*x*_‐ATP as a probe. Other ^18^O‐labelled phosphates or phosphoanhydrides are scarce. The high potential of ^18^O‐labelled phosphates beyond ^18^O‐ATP is insufficiently exploited in large part because of a “dearth of synthetic methods”.^[32]^

**Scheme 1:**
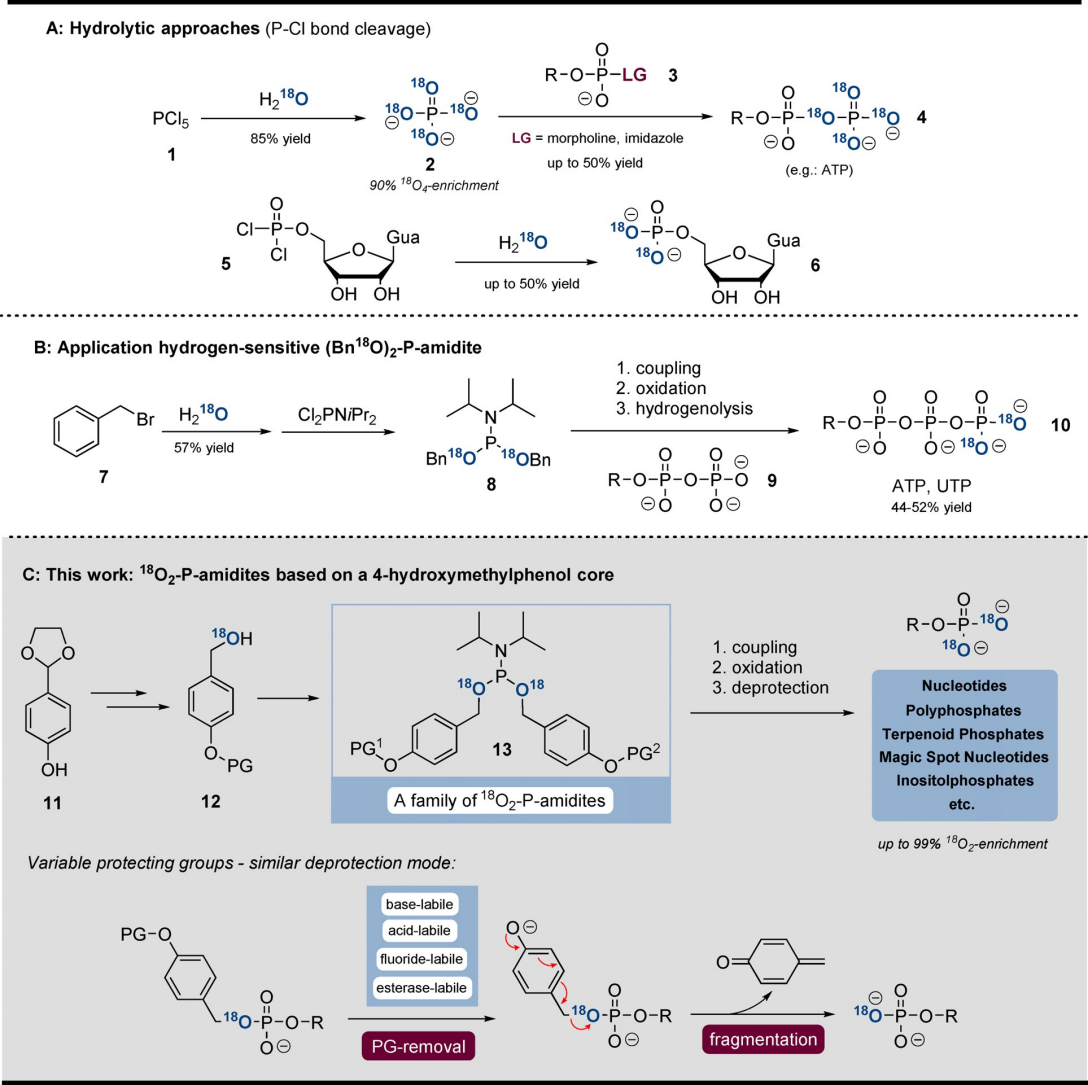
A) hydrolytic approaches towards ^18^O_4_‐phosphates. B) Application of (Bn^18^O)_2_‐P‐amidite towards labelled NTPs. C) New synthetic concept towards ^18^O_2_‐phosphates. The general deprotection mode of all ^18^O‐P‐amidites developed is shown.

The most common strategies are based on hydrolysis of P−Cl bonds (Scheme 1 A). Especially ^18^O_4_‐P_i_ (**2**) is an important building block. Formed by hydrolysis of PCl_5_ (**1**) in H_2_^18^O, ^18^O_4_P_i_ (**2**) may undergo S_N_‐reaction with activated P^V^‐electrophiles (**3**), leading to ^18^O_4_‐containing phosphoanhydrides (**4**).[^13^, ^33^, ^34^] Furthermore, hydrolysis of phosphorodichloridates in H_2_^18^O is suitable to access ^18^O_2_‐monophosphates.^[35]^ ^18^O‐labelled RNA was obtained by Hamasaki et al. by using H_2_^18^O as solvent in the oxidation step of solid‐phase synthesis.^[17]^ Also, the Stec reaction is suitable to incorporate ^18^O from benzaldehyde into phosphoramidates.^[36]^ In addition, enzymatic approaches were described to access γ‐^18^O_4_‐ATP and γ‐^18^O_4_‐GTP from ^18^O_4_‐P_i_ (**2**).^[37]^ Our group presented a method for γ‐^18^O_2_‐NTP (**10**) synthesis based on an ^18^O_2_‐dibenzyl‐P‐amidite **8**.^[38]^ However, during H_2_‐reduction, some nucleobases (C, G) were also reduced and substantial amounts of P‐anhydrides (>20 %) were hydrolysed. Therefore, even though there are several methods available for the synthesis of ^18^O_*x*_‐phosphates, significant improvements regarding substrate scope, yields, scalability, ^18^O‐enrichment ratios, and reduction of H_2_^18^O consumption are required to unleash the true potential of ^18^O‐phosphate labelling.

Herein, we describe a unifying and modular concept for the synthesis of ^18^O_2_‐phosphate products, addressing the limitations specified above. The concept involves a family of ^18^O_2_‐phosphoramidites (**13**), equipped with various protecting groups (removable by piperidine, DBU, H^+^, F^−^, or esterases (Scheme 1 C)). After triggering deprotection, the fragmentation proceeds analogously via self‐immolation of a divergently modified ^18^O‐4‐(hydroxymethyl)phenol adapter (Scheme 1 C). The broad applicability of the P‐amidite family is demonstrated by the synthesis of various important naturally occurring phosphates: nucleotides, inorganic polyphosphates (polyphosphates thereafter), terpenoid phosphates, magic spot nucleotides, inositol phosphates, and DNA. The synthesis relies on telescoping sequences of phosphitylation, oxidation and deprotection^[39]^ and usually results in high yields and complete ^18^O incorporation while enabling gram‐scale synthesis of labelled products. We demonstrate the utility of phosphate‐labelled inositol phosphates in capillary electrophoresis mass‐spectrometry‐based (CE‐ESI‐QqQ) analytics. Our CE‐ESI‐QqQ setup enables accurate assignment of analytes without neutral loss from diverse biological matrices.

## Results and Discussion

### Synthesis of Functionalised ^18^O‐4‐(Hydroxymethyl)phenols

**Scheme 2:**
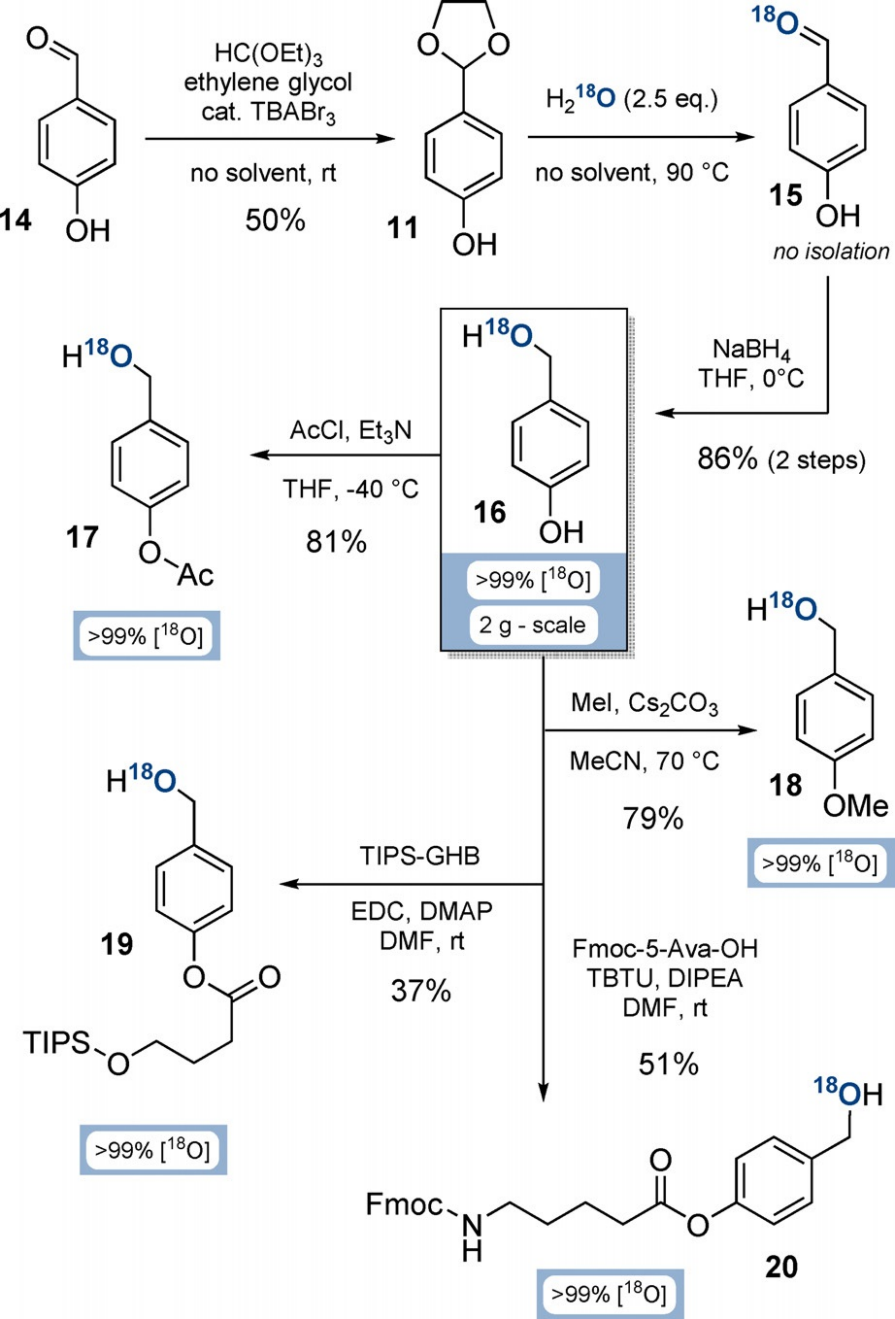
Syntheses of functionalised ^18^O‐(hydroxymethyl)phenols from 4‐hydroxybenzaldehyde (**16**). Abbreviations: TBABr_3_: tetrabutylammonium tribromide; THF: tetrahydrofurane; TIPS‐GHB: 4‐((triisopropylsilyl)oxy)butanoate; EDC: 1‐ethyl‐3‐(3‐dimethylaminopropyl)carbodiimide; DMAP: 4‐dimethylaminopyridine; DMF: dimethylformamide; Fmoc‐5‐Ava‐OH: 5‐(Fmoc‐amino)valeric acid; TBTU: 2‐(1*H*‐benzotriazole‐1‐yl)‐1,1,3,3‐tetramethylaminium tetrafluoroborate; DIPEA: diisopropylethylamine.

The key intermediate on the way to ^18^O‐P‐amidites is ^18^O‐4‐(hydroxymethyl)phenol (**16**, Scheme 2). Its synthesis commenced from 4‐hydroxybenzaldehyde (**14**) by acetalisation in 50 % yield on multi‐gram scale.^[40]^ To ensure the required purity (>99 %) of acetal **11**, we developed a crystallisation procedure from cyclohexane/ethylacetate. Other acetalisation conditions were unsuccessful, as p‐donor substituted benzacetals are highly prone to hydrolysis.^[41]^

The high tendency of acetal **11** to hydrolyse was subsequently exploited by melting a mixture of **11** and H_2_^18^O (2.5 equiv, >99 % isotopic purity). ^18^O‐Labelled aldehyde **15** was unstable towards humidity‐induced H_2_^16^O back‐exchange, resulting in loss of labelling efficiency. To overcome this issue, **15** was directly reduced with dry NaBH_4_ in triethylene glycol dimethyl ether to access key building block ^18^O‐(hydroxymethyl)phenol **16** in 86 % yield over 2 steps, and with an isotopic purity of >99 % on a multi gram‐scale.

Subsequently, the phenol group of ^18^O‐diol **16** was functionalised divergently to access various P‐amidite precursors under conservation of the ^18^O isotopic purity (Scheme 2): Acetylation towards **17** with AcCl and Et_3_N was performed in 81 % yield on gram‐scale. The methylated derivative **18** was accessed in 79 % yield on gram‐scale by using MeI and Cs_2_CO_3_. Silyl‐protected derivative **19** was obtained in 37 % yield by esterification of 4‐TIPSO‐butanoate with EDC and DMAP. Finally, ^18^O‐BigFM‐alcohol (**20**) was synthesised from 5‐Fmoc‐aminovaleric acid, TBTU and DIPEA in 51 % yield.

### Synthesis of ^18^O‐P‐Amidites

**Scheme 3:**
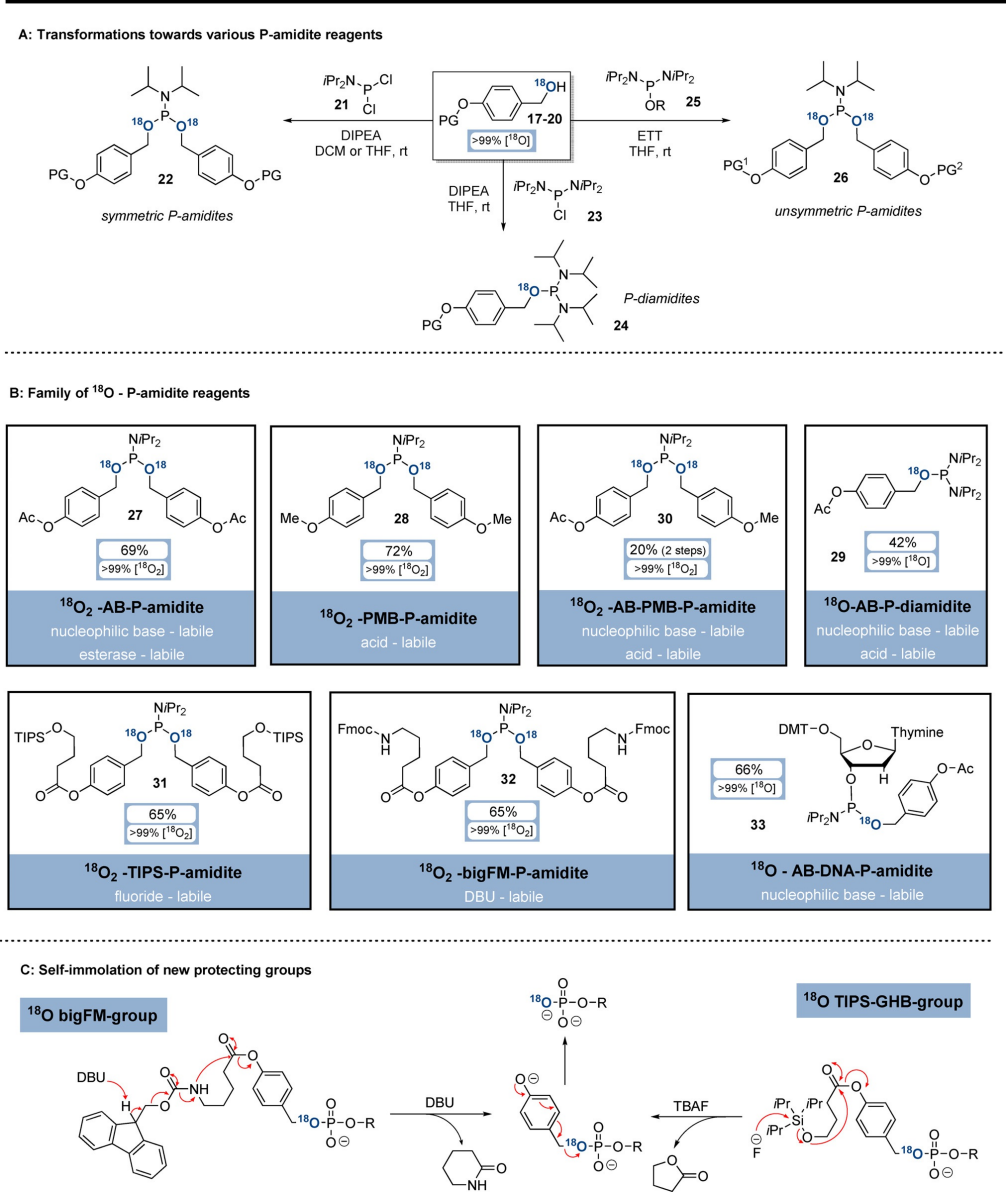
Synthesis of ^18^O‐P‐amidites (A,B). Self‐immolation of novel phosphate protecting groups after the trigger (C). Abbreviations: AB: *para*‐acetoxybenzyl;PMB: *para*‐methoxybenzyl, DCM: dichloromethane; ETT: 5‐ethylthio‐1*H*‐tetrazole.

In the next steps, the functionalised ^18^O‐alcohols were converted into the corresponding ^18^O‐P‐amidites (Scheme 3). Symmetric ^18^O_2_‐P‐amidites (**22**) could be accessed by subjecting functionalised ^18^O‐alcohols **17**–**20** to S_N_‐conditions with *i*Pr_2_N‐PCl_2_ (**21**) and Hünigs base (Scheme 3 A). ^18^O‐P‐diamidites (**24**) were synthesised analogously from (*i*Pr_2_N)_2_P‐Cl (**23**) and were then transformed with ETT and ^18^O‐alcohols to obtain unsymmetric ^18^O_2_‐P‐amidites (**26**). Application of these methods to ^18^O‐alcohols **17**–**20** provided access to a family of ^18^O‐P‐amidites (Scheme 3 B), equipped with orthogonal protecting groups. The isotopic enrichment was preserved during all transformations, and the P‐amidites were isolated in ^18^O/^16^O‐ratios of >99 %. ^18^O_2_‐AB‐P‐Amidite **27** was synthesised from ^18^O‐AB‐alcohol **17** in 69 % yield on gram‐scale. Its cleavage can be triggered with amine nucleophiles (e.g. pyrrolidine) or esterases.^[42]^ ^18^O_2_‐PMB‐P‐Amidite **28** was obtained from ^18^O‐PMB‐alcohol **18** in 72 % yield on gram‐scale. PMB is labile towards acids, such as trifluoroacetic acid.^[43]^ ^18^O‐AB‐P‐Diamidite **29** was accessed from ^18^O‐AB‐alcohol **17** in 42 % yield and was transformed further with ^18^O‐PMB alcohol **18** to unsymmetrically modified ^18^O_2_‐AB‐PMB‐P‐amidite **30**.

In addition, the novel P‐amidites **31** and **32** based on the 4‐(hydroxymethyl)phenol core were developed to enable further orthogonal deprotection conditions. TIPS‐containing ^18^O‐alcohol **19** was transformed to silyl‐protected ^18^O_2_‐P‐amidite **31** in 65 % yield. Its deprotection mechanism (Scheme 3 C) includes TBAF‐induced TIPS‐removal triggering 5‐ring lactonisation and fragmentation towards unprotected ^18^O‐phosphates. ^18^O_2_‐BigFM‐P‐amidite **32** was synthesised from ^18^O‐BigFM‐alcohol **20** in 65 % yield. The deprotection mechanism of **32** (Scheme 3 C) is based on a DBU‐induced self‐immolation cascade of Fmoc‐elimination, 6‐ring lactamisation and fragmentation, liberating ^18^O‐phosphates.

Finally, the DNA precursor ^18^O‐AB‐DMT‐thymine‐P‐amidite **33** was accessed in 66 % yield from ^18^O‐AB‐alcohol **17** and the corresponding P‐diamidite. P‐amidite **33** is fully compatible with automated solid‐phase DNA‐synthesis, as the AB‐group is removed under comparable conditions required for β‐CE‐group cleavage. The whole family of ^18^O‐P‐amidite reagents can be stored for months at −20 °C without substantial decomposition and deterioration of isotopic purity.

### Synthesis of ^18^O‐Phosphorylated Products

**Figure 2:**
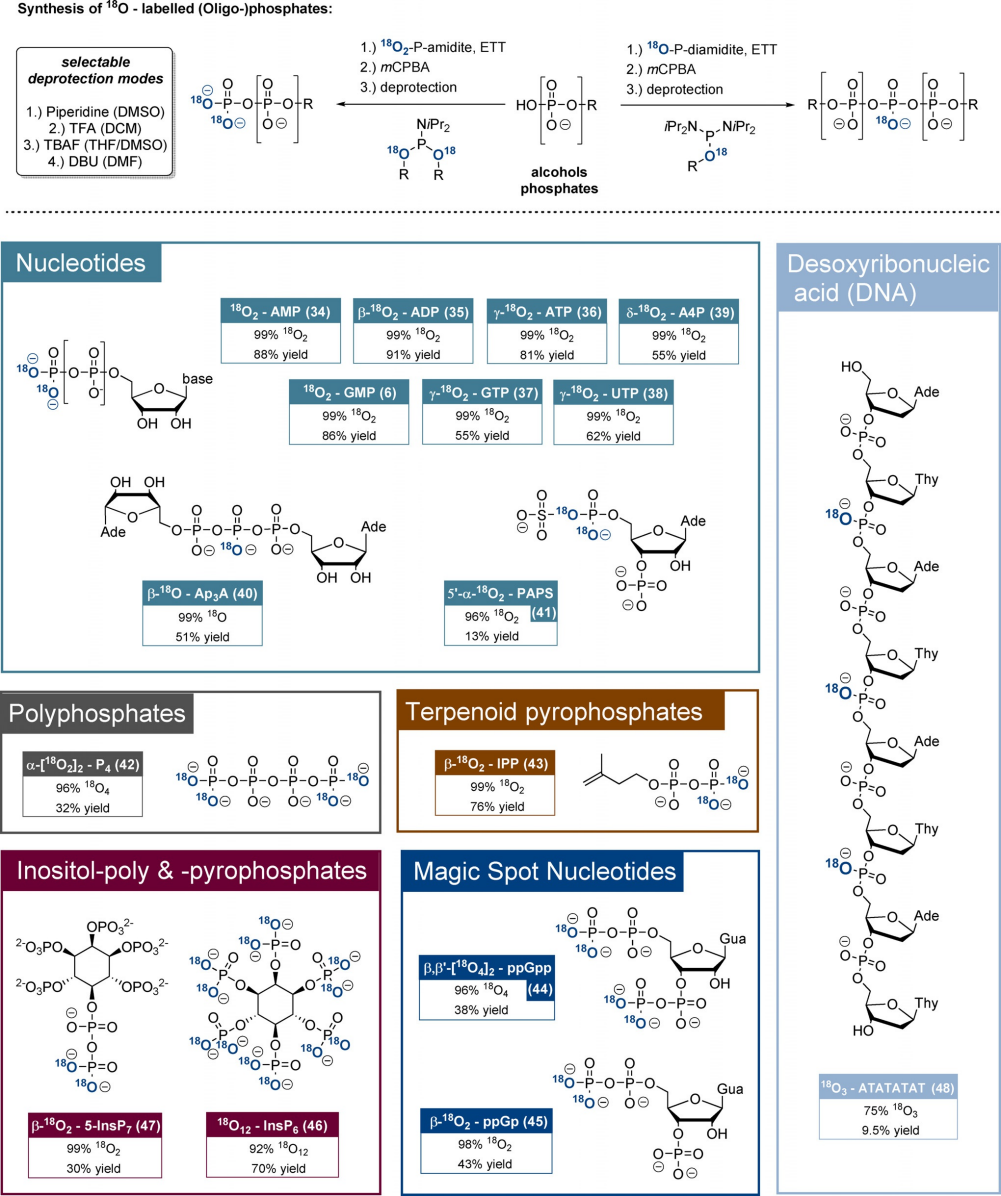
Top: Main synthetic concept towards the ^18^O_*x*_‐labelled natural products. DNA solid‐phase synthesis is not shown. Bottom: Overview of ^18^O‐labelled phosphate products, including yields and ^18^O_*x*_‐enrichment rates. The yields usually refer to the ^18^O_2_‐phosphate introduction procedure (coupling, oxidation, global deprotection) only, except in the cases of PAPS (**41**), ppGp (**45**) and DNA (see SI for detailed information). In the case of PAPS (**41**) the product represents an isotopomeric mixture, where only one isotopomer is shown. Abbreviations: DMSO: dimethylsulfoxide; *m*CPBA: *meta*‐chloroperbenzoic acid; DBU: diazabicycloundecene; AMP: adenosine‐5’‐monophosphate; GMP: guanosine‐5’‐monophosphate; ADP: adenosine‐5’‐diphosphate; ATP: adenosine‐5’‐triphosphate; GTP: guanosine‐5’‐triphosphate; UTP: uridine‐5’‐triphosphate; A4P: adenosine‐5’‐tetraphosphate; Ap3A: diadenosine triphosphate. IPP: isoprenylpyrophosphate; ppGpp: guanosine‐3’,5’‐bisdiphosphate; ppGp: guanosine‐3’‐phosphate‐5’‐diphosphate; PAPS: adenosine‐3’‐phosphate‐5’‐phosphosulfate.

^18^O_2_‐P‐amidites **27**–**33** were then applied in the syntheses of diverse ^18^O‐labelled phosphates mostly according to methods developed in our group in recent years.[^39^, ^44^] The general ^18^O_2_‐phosphorylation reactions (Figure 2, top) were based on ETT‐promoted phosphitylations of alcohols and phosphates by ^18^O_2_‐P‐amidites followed by oxidation and deprotection. In contrast, ^18^O‐P‐diamidites were used for phosphate dimerisation reactions. In many cases, the chemoselective nature of P‐amidite phosphitylation was exploited, so unprotected nucleotides were suitable starting materials. Detailed synthesis schemes are provided in the SI.

We initially evaluated the reagents regarding access to ^18^O‐labelled nucleotides (Figure 2, bottom). ^18^O_2_‐AMP (**34**) and ^18^O_2_‐GMP (**6**) were synthesised from the corresponding 2′,3′‐isopropylidene nucleosides using ^18^O_2_‐PMB‐P‐amidite **28** in yields of 88 % and 86 % as well as excellent ^18^O_2_‐enrichment ratios of 99 % after global deprotection. Nucleoside oligophosphates NP_*x*_ were accessed from NP_*x*−1_ precursors by using ^18^O_2_‐AB‐P‐amidite **27**. Accordingly, β‐^18^O_2_‐ADP (**35**), *γ*‐^18^O_2_‐ATP (**36**), γ‐^18^O_2_‐GTP (**37**), γ‐^18^O_2_‐UTP (**38**) and δ‐^18^O_2_‐AP_4_ (**39**) were isolated in yields of 55–91 % and consistently with high ^18^O_2_/^16^O_2_ ratios of >99 %. Notably, ^18^O_2_‐AMP and γ‐^18^O_2_‐ATP were synthesised on gram‐scales, underlining the method's robustness and the significant advantage of introducing the stable isotope labels in the final steps. Chemoselective dimerisation of AMP using ^18^O‐AB‐diamidite **29** gave β‐^18^O‐Ap3A (**40**) in 51 % yield and with 99 % ^18^O‐enrichment.

As additional targets, the first ^18^O‐labelled polyphosphate representative ^18^O_4_‐P4 (**42**) was accessed from pyrophosphate in 33 % yield using a bisphosphorylation procedure with ^18^O_2_‐AB‐P‐amidite **27** and with a >95 % ^18^O_4_‐enrichment. The terpenoid phosphate β‐^18^O_2_‐isoprenylpyrophosphate (**43**) was synthesised from isoprenylphosphate and TIPS‐protected ^18^O_2_‐P‐amidite **31** followed by deprotection in 78 % yield and 99 % ^18^O_2_‐enrichment.

^18^O_4_‐ppGpp as a representative of the important magic spot nucleotides was synthesised from guanosine‐3,5‐bisphosphate (pGp) using ^18^O_2_‐BigFM‐P‐amidite (**32**) in a chemoselective bisphosphorylation procedure.^[45]^ Other ^18^O_2_‐P‐amidites failed in the construction of magic spot nucleotides as the guanosine‐2’,3’‐cyclophosphate‐5’‐pyrosphosphate (ppGcp) byproduct was formed quantitatively under deprotection conditions. Only when applying ^18^O_2_‐BigFM‐P‐amidite, DBU‐induced deprotection led to a 1:1 mixture of ^18^O_4_‐ppGpp (**44**) and ^18^O_2_‐ppGcp. The products were separated and ^18^O_2_‐ppGcp was transformed into ^18^O_2_‐ppGp (**45**) by RNase T2. Consequently, ^18^O_4_‐ppGpp (**44**) and ^18^O_2_‐ppGp (**45**) originated from the same reaction mixture in yields of 38 % and 43 %. ^18^O_4_‐ and ^18^O_2_‐enrichment ratios were 96 % and 98 %, respectively.

Furthermore, several ^18^O‐labelled inositolphosphates were synthesised. ^18^O_12_‐InsP_6_ (**46**) with twelve ^18^O labels (M+24) was accessed directly from *myo*‐inositol with ^18^O_2_‐AB‐P‐amidite **27** in 70 % yield and with 92 % ^18^O_12_‐enrichment. ^18^O_2_‐5‐InsP_7_ was synthesised according to literature precedent from AB_10_‐(β‐CE)_2_‐InsP_6_ in 30 % yield and >99 % ^18^O_2_‐enrichment with the labels on the β‐phosphate. In this case, unsymmetric ^18^O_2_‐AB‐PMB‐P‐amidite (**30**) had to be used to ensure a >98 % purity of the product. Importantly, AB‐protected and thus cell‐permeable ^18^O‐prometabolites^[46]^ were isolated as intermediate products, offering possible applications for in cellulo transphosphorylation experiments. The flexible ^18^O‐(hydroxymethyl)phenol‐based P‐amidites compared favourably to the known (Bn^18^O)_2_‐P‐amidite, which was used for accessing ^18^O_2_‐1‐InsP_7_ (see SI).^[47]^ After hydrogenation, ^18^O_2_‐1‐InsP_7_ was isolated in a yield of 17 % and an ^18^O_2_‐enrichment ratio of 92 %. Moreover, partial anhydride hydrolysis during deprotection resulted in the formation of 20 % InsP_6_, which is difficult to remove.

The ^18^O‐labelled DNA sequence ^18^O_3_‐5’‐ATATATAT (**48**) was accessible by automated solid‐phase synthesis using DMT‐dT‐P‐(^18^OAB)‐N*i*Pr_2_ (**33**) and commercial DMT‐dA(N‐Bz)P‐(β‐CE)‐N*i*Pr_2_. AB‐ and β‐CE‐groups were deprotected using an aqueous mixture of ammonia and methylamine (AMA). The DNA sequence was isolated in 9.5 % yield and 75 % ^18^O_3_‐enrichment. The ^18^O‐DNA and its ^16^O_3_‐sibling show clearly separated mass spectra, despite the comparably moderate enrichment. Probably the aqueous DNA‐deprotection and oxidation steps impact the enrichment in comparison to other natural products.

**Scheme 4:**
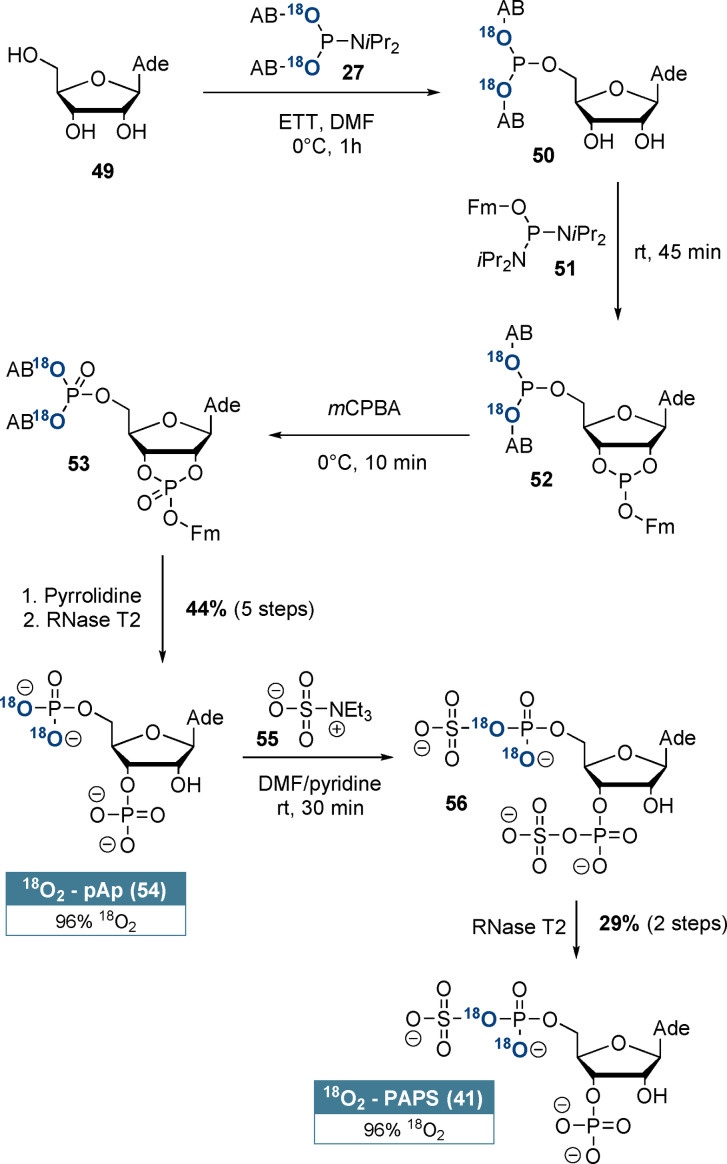
Synthesis of regioselectively labelled ^18^O_2_‐PAPS (**41**) from adenosine (**49**). pAp was the only intermediate product that required purification.

The synthesis of another important cofactor,^[48]^ ^18^O_2_‐labelled adenosine‐3’‐phosphate‐5’‐phosphosulfate (**41**, PAPS) is shown in detail in Scheme 4. Our synthesis sequence relies on telescoping of the first five steps and ensures selective ^18^O‐incorporation at the 5’‐position: Adenosine (**49**) was selectively 5’‐phosphitylated using ^18^O_2_‐AB‐P‐amidite **27**. Subsequently, cyclophosphate **52** was formed with (FmO)‐P‐diamidite (**51**) in a ring‐forming phosphitylation reaction. After oxidation and basic deprotection, RNase T2 catalysis induced regioselective cyclophosphate hydrolysis towards adenosine‐3’‐5’‐bisphosphate (**54**, pAp) in 44 % yield and with 96 % ^18^O_2_‐enrichment after 5 steps without intermediate purification. pAp (**54**) was chemoselectively bis‐sulfated by triethylamine‐*N*‐sulfonic acid (**55**) according to Horwitz et al.^[49]^ RNase T2 catalysis led to a net 3’‐desulfation, delivering ^18^O_2_‐PAPS (**41**) in 29 % yield from pAp and with 96 % ^18^O_2_‐enrichment. This novel synthesis of ^18^O_2_‐PAPS (**41**) further demonstrates the great utility of (hydroxymethyl)phenol‐based ^18^O_2_‐P‐amidites for isotope incorporation into complex molecular frameworks.

### Determination of InsPs in Biological Samples by CE‐MS

We have recently reported the use of ^13^C‐labelled inositol phosphates and pyrophosphates as internal references for quantitative mass spectrometry.^[50]^ This approach requires the transformation of ^13^C‐labelled glucose into *myo*‐inositol with three consecutive enzymatic reactions.^[51]^ ^18^O labelling could be an interesting alternative due to the lower cost of the ^18^O label and since it would not require the initial enzymatic conversion of glucose into inositol. However, these advantages would only be relevant if neutral phosphate loss resulting from in‐source fragmentation could be avoided.^[52]^

To evaluate the utility of the new internal references ([^18^O_12_] InsP_6_ (**46**), [^18^O_2_] 5‐InsP_7_ (**47**), [^18^O_2_] 1‐InsP_7_), we used several biological extracts (mammalian cells, slime‐mold, plant), which were spiked with the compounds. Next, we resolved the extracts by capillary electrophoresis and analysed them by on‐line mass spectrometry. While an ESI‐QToF system produced substantial neutral loss (ca. 10 %),^[50]^ this was not the case in an ESI‐QqQ system, a common MS analyser for quantitative studies, where we found less than 0.3 % of neutral phosphate loss (Supplementary Figure 11).

**Figure 3:**
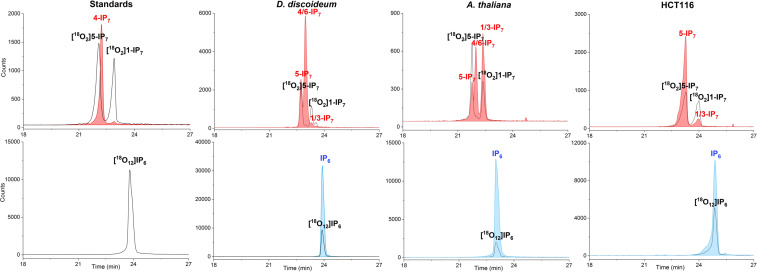
Electropherograms of InsP_7_ and InsP_6_ in standards and cell extracts (*Dyctyostelium discoideum*, *Arabidopsis thaliana* and mammalian HCT116 cells) spiked with the heavy isotopologues. The spiked‐in [^18^O_2_] 5‐InsP_7_, [^18^O_2_] 1‐InsP_7_, [^18^O_12_] InsP_6_ concentrations are 4 μM, 4 μM and 20 μM, respectively. The detailed CE‐ESI‐QqQ method is described in the SI.

Figure 3 shows electropherograms of several analysed samples spiked with heavy isotopologues. Clearly, the reference compounds co‐migrate with their light analytes, enabling ready assignment of the different isomers, irrespective of the matrix. For example, we can easily assign the known distinct isomers present in mammalian HCT116 cells (1‐ and 5‐InsP_7_) and would also be able to quantify them with the internal references. In slime mold and plants, an additional isomer could be detected which we have previously tentatively assigned as 4/6‐InsP_7_.^[53]^ Once this new isomer will become accessible as its synthetic isotopologue following the procedures described herein, in‐depth studies into its dynamic regulation will become possible.

## Conclusion

In summary, we introduce a unique ^18^O‐P‐amidite‐based approach for the synthesis of various ^18^O‐labelled phosphorylated products. The utility of the new family of reagents is demonstrated by the scalable synthesis of ^18^O‐labelled nucleotides, such as for example, AMP, ADP, ATP, AP_4_, and PAPS, as well as the challenging magic spot nucleotides. Moreover, synthetically highly demanding ^18^O‐inositolpyrophosphates are accessible. ^18^O_*x*_‐isotopic enrichment ratios of 92–99 % and good yields were obtained consistently, while the labels are introduced in the final steps of the synthesis, which can be a significant advantage. The heavy phosphate labels can be used as internal reference compounds for mass spectrometry applications, as demonstrated herein. Identical migration times allow a straightforward assignment of the isomeric identity of inositol phosphate analytes from different biological matrices, such as mammalian cells, slime mold, and plants. Importantly, our CE‐ESI‐QqQ setup shows less than 1 % phosphate neutral loss across the board of analytes, paving the way for their accurate quantitation. This novel toolbox will enable further innovative applications of ^18^O‐labels in various fields of research involving nature's favourite modification: the phosphate group.

## Conflict of interest

The authors declare no conflict of interest.

## Supporting information

As a service to our authors and readers, this journal provides supporting information supplied by the authors. Such materials are peer reviewed and may be re‐organized for online delivery, but are not copy‐edited or typeset. Technical support issues arising from supporting information (other than missing files) should be addressed to the authors.

Supporting Information
