# Supplementary material for: Stable Isotope Phosphate Labelling of Diverse Metabolites is Enabled by a Family of 18O‐Phosphoramidites
Source: Angew Chem Int Ed Engl. 2021 Nov 23;61(5):e202112457. doi: 10.1002/anie.202112457 (PMC9298905; doi:10.1002/anie.202112457)

Angewandte Chemie
Eine Zeitschrift der Gesellschaft Deutscher Chemiker

Supporting Information

Stable Isotope Phosphate Labelling of Diverse Metabolites is Enabled by a Family of ^{18}O-Phosphoramidites**

*Thomas M. Haas, Stephan Mundinger, Danye Qiu, Nikolaus Jork, Kevin Ritter, Tobias Dürr-Mayer, Alexander Ripp, Adolfo Saiardi, Gabriel Schaaf, and Henning J. Jessen**

anie_202112457_sm_miscellaneous_information.pdf

Table of content

1. General remarks
2. Synthesis of literature known compounds
3. Synthetic procedures of ^{18}O-4-hydroxybenzylic alcohol
4. Synthesis of functionalized ^{18}O-4-hydroxybenzylic alcohols
5. Synthesis of $^{18}O_2$-P-amidites
6. Synthesis of $^{18}O_n$ – phosphate natural products
   - 6.1. Synthesis of $^{18}O_n$ – Nucleotides
   - 6.2. Synthesis of $^{18}O_n$ – Terpenoidphosphates
   - 6.3. Synthesis of $^{18}O_n$ – Polyphosphates
   - 6.4. Synthesis of $^{18}O_n$ – Magic Spot Nucleotides
   - 6.5. Synthesis of $^{18}O_n$ – Inositolpoly and -pyrophosphates
   - 6.6. Synthesis of $^{18}O_n$ – DNA
   - 6.7. Synthesis of $^{18}O_n$ – Phosphoaminoacids
7. CE-MS measurements
8. NMR-spectra
9. HRMS-spectra

Abbreviations

A4P	Adenosine-5’-tetraphosphate
AB	4-Acetoxybenzyl
ADP	Adenosine-5’-diphosphate
AMP	Adenosine-5’-monophosphate
ATP	Adenosine-5’-triphosphate
BSTFA	N,O-Bis(trimethylsilyl)trifluoroacetamide
CH	Cyclohexane
DBU	1,8-Diazabicyclo[5.4.0]undec-7-ene
DCM	Dichloromethane
DIPEA	Diisopropylethylamine
DMF	Dimethylformamide
DMSO	Dimethyl sulfoxide
DMT	Dimethoxytrityl
ETT	5-(Ethylthio)-1*H*-tetrazole
ESI	Electron Spray ionization
FmOH	Fluorenylmethanol
GMP	Guanosine-5’-monophosphate
GTP	Guanosine-5’-triphosphate
HRMS	High resolution mass spectrometry
IPP	Isoprenylpyrophosphate
*m*CPBA	*meta*-Chloroperoxybenzoic acid
min	Minutes
NMR	Nuclear Magnetic Resonance
pGp	Guanosine-3’,5’-bisphosphate
ppGp	Guanosine-3’-phohsphate-5’-diphosphate
ppGpp	Guanosine-3’,5’-bis(diphosphate)
PAPS	Adenosine-3’-phosphate-5’-phosphosulfate
PMB	Para-methoxybenzyl
PPi	Pyrophosphate
RNase T2	Ribonuclease T2
RP-HPLC	Reverse phase high-performance liquid chromatography
rt	Rroom temperature
SAX	Strong ion-exchange chromatography
TBA	Tetrabutylammonium
TBAF	Tetrabutylammonium fluoride
TBHP	*tert*-Butylhydroperoxide
TEAA	Triethylammonium acetate
THF	Tetrahydrofurane
TIPSCl	Triisopropylsilylchloride
Triglyme	Triethylenglycoldimethylether

1. General remarks

Reactions were carried out using flame-dried glassware under an atmosphere of dry Argon and magnetically stirred, unless noted otherwise. Air- and moisture-sensitive liquids and solutions were transferred via syringe or stainless steel canula.

$H_2{}^{18}O$ (>99% purity, 99% isotopic enrichment) was purchased from *Taiyo Nippon Sanso* (respectively from *Cortecnet* as european vendor).

Reagents were purchased from commercial suppliers (Acros, Aldrich, Fluka, TCI) and used without further purification, unless noted otherwise.

Solvents were obtained in analytical grade and used as received for extractions, precipitation and solid washing.

Dry solvents for reactions were purchased in a dry form from Sigma and stored over molecular sieves as well as under the atmosphere of dry N_2.

Deuterated solvents for NMR and reactions were obtained from Armar Chemicals, Switzerland and euriso-top, Germany, in the indicated purity grade and used as received for NMR spectroscopy.

Strong ion-exchange chromatography was performed using an automated Äkta® – system. Q-Sepharose was purchased from Aldrich. Buffer solutions were produced manually using milliQ H_2O.

TBA-salt preparations were performed by either using DowexH^+ followed by TBA(OH) addition or Chelex®100 (preloaded with TBA). In both cases, the TBA salts were obtained after lyophilization.

Commercially available phosphates were transformed into their corresponding TBA-salts as described above.

Ribonuclease T2 from Aspergillus oryzae (50 ku) was purchased as solid from Worthington Biochemical Corporation as lyophilized powder and dissolved in a storage buffer [5.0 ml, glycerol / NaH_2PO_4 (10 mM, pH 6.8) = 1/1]. The stock solution was stored for months at -20°C without loss of function.

Preparative RP-MPLC was performed using an automated Interchim® - system. The AQ-solid phase was purchased from Interchim.

Preparative RP-HPLC was performed using an Azura preparative LC-system with C18-AQ-column and UV-detection.

Lyophilizations were done with Christ Freeze Dryer Alpha 1-4 LDplus and Christ Freeze Dryer Alpha 1-2 LDplus.

Analytical HPLC-MS measurements were performed using a Thermofisher Ultimate 3000 system coupled to MSQplus. C18-AQ-columns were purchased from ProntoSil.

^{1}H-NMR spectra were recorded on Bruker 300 MHz spectrometers, Bruker 400 MHz (with cryoprobe) and Bruker 500 MHz spectrometers in the indicated deuterated solvent. Data are reported as follows: chemical shift (δ, ppm), multiplicity (s, singlet; d, doublet; t, triplet; q, quartet; m, multiplet; br. s, broad signal), coupling constant(s) (*J*, Hz), integration. All signals were referenced to the internal solvent signal as standard (D_2O, δ 4.79; MeCN-d3, δ 1.94, $CDCl_3$, 7.26).
In some phosphate products there is still acetate (mostly as TEA-salt) or acetone present, as complete drying of the products sometimes induce decomposition. These residues were considered for yield determination. After NaClO4 – purification acetone residues were present in the products. These were also considered for yield determination.

^{13}C{^{1}H}-NMR spectra were recorded with ^{1}H-decoupling on Bruker 126 MHz, Bruker 101 MHz (with cryoprobe) spectrometers at 298K in the indicated deuterated solvent.

^{31}P{^{1}H}-NMR spectra and ^{31}P-NMR spectra were recorded with ^{1}H-decoupling or ^{1}H coupling, respectively, on Bruker 202 MHz , 162 MHz (with cryoprobe) and Bruker 122 MHz

spectrometers in the indicated deuterated solvent. All signals were referenced to an internal standard (PPP).

Mass spectra were recorded by C. Warth (Mass spectrometry service of the University of Freiburg) on a Thermo LCQ Advantage [spray voltage: 2.5 – 4.0 kV, spray current: 5 µA, ion transfer tube: 250 (150) °C, evaporation temperature: 50 – 400°C.

2. Syntheses adapted from literature

1,1-dichloro-N,N-diisopropylphosphanamine (21)

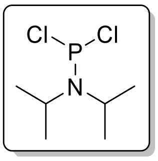

The compound was synthesized according to Ripp et al.[1] Analytical data are in accordance with literature.

1-((9H-fluoren-9-yl)methoxy)-N,N,N',N'-tetraisopropylphosphanediamine (51)

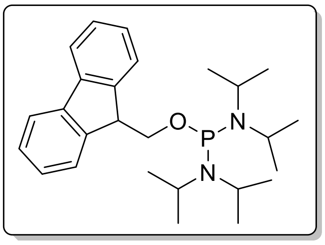

The compound was synthesized according to Hofer et al.[2] Analytical data are in accordance with literature.

2’-Deoxy-5’-O-DMT-thymid-3’-yl)bisdiisopropyl-P-diamidite (SI-1):

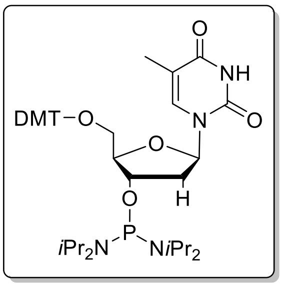

The compound was synthesized according to Leisvuori et al. on a 1.8 mmol scale.[3] Analytical data are in accordance with literature.

3. Synthesis of ^{18}O-4-(hydroxymethyl)phenol

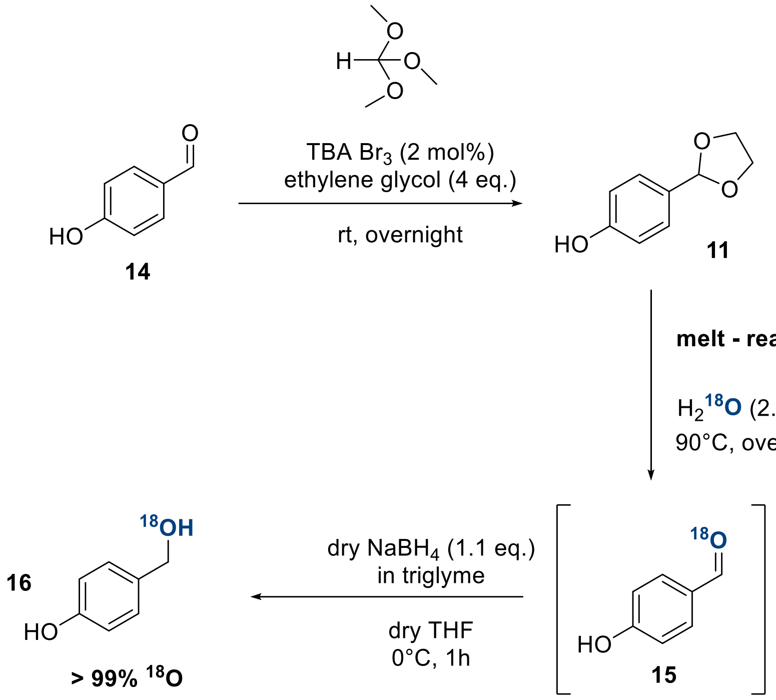

Supporting Figure 1: synthetic route from 4-hydroxybenzaldehyde (**14**) to 18O-4-(hydroxymethyl)phenol (**16**).

4-(1,3-dioxolan-2-yl)phenol (11)

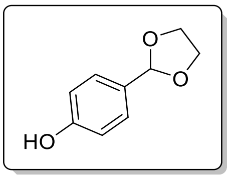

To a solution of 4-hydroxybenzaldehyde (**14**, 10.0 g, 81.9 mmol) and trimethylorthoformate (10.4 g, 10.8 mL, 98.3 mmol, 1.2 eq.) in ethylenglycol (20.3 g, 18.3 mL, 327 mmol, 4.0 eq.) was added tetrabutylammoniumtribromide (1.97 g, 4.09 mmol, 5.0 mol%). The solution was stirred overnight at rt. The solvent was removed under high vacuum and the crude product was filtered over Alox N (Ø = 6 cm, h = 10 cm) with CH:AcOEt [2:1 (750 mL), 1:1 (1350 mL)]. The product containing fractions were combined and evaporated under reduced pressure at 30°C. The product was crystallized from CH:AcOEt (7:1, 500 mL). The product (**11**, 6.81 g, 41.0 mmol, 50%) was isolated as colorless crystals.

Note: The Product is stored in a desiccator over vacuum and P_2O_5 at rt without substantial decomposition for weeks. Under ambient conditions the product will hydrolyze substantially in hours!

^{1}H-NMR (400 MHz, $CDCl_3$, δ/ppm): 7.39 – 7.30 (m, 2H), 6.80 – 6.67 (m, 2H), 5.77 (s, 1H), 5.72 (s, 1H), 4.22 – 4.11 (m, 2H), 4.10 – 4.00 (m, 2H). **^{13}C{^{1}H}-NMR** (101 MHz, $CDCl_3$,

δ/ppm): 156.77, 129.60, 128.29, 115.41, 103.93, 65.35. **HRMS** (ESI) m/z for $C_9H_9O_3$ $[M+H]^+$: calcd. 165.0557, found 165.0558. **Smp.:** 70-72°C.

^{18}O-4-((hydroxyl)methyl)phenol (16)

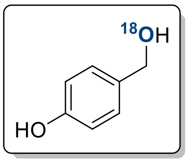

A dry microwave reaction vial with cap was stored overnight in a desiccator over vacuum and P_4O_{10} and filled with 4-(1,3-dioxolan-2-yl)phenol (**11**, 3.32 g, 20.0 mmol). After sealing the vial was evacuated at high vacuum and flooded with argon twice. Afterwards $H_2{}^{18}O$ (1.00 g, 50.0 mmol, 2.5 eq.) was added. The mixture was melted and stirred at 90°C overnight. After cooling down to rt, the resulting solid was dissolved in dry THF (60 mL) and transferred into a dry round bottom flask. Then $NaBH_4$ (2M in triglyme, 11.0 mL, 22.0 mmol, 1.1 eq.) was added in 5 min at 0°C. The reaction mixture was stirred at rt until conversion was complete (ca. 30 min). The reaction was stopped by adding of Na_2SO_4. After filtration and washing with THF, the solvent was removed under reduced pressure. The resulting crude product was purified by flash chromatography (pentane:AcOEt = 1:1). The resulting oil was crystallized from toluene (40 mL) and the product (**16**, 2.16 g, 17.1 mmol, 86%) was isolated as white crystals. The product was stored in the desiccator over high vacuum and P_4O_{10}.

^{1}H-NMR (400 MHz, CD_3CN, δ/ppm): 7.20 – 7.12 (m, 2H), 6.85 (s, 1H), 6.81 – 6.73 (m, 2H), 4.46 (d, J = 5.8 Hz, 1H), 3.02 (t, J = 5.8 Hz, 1H).**^{13}C{^{1}H}-NMR** (101 MHz, CD_3CN, δ/ppm): 157.01, 134.36, 129.47, 115.91, 64.52. **HRMS** (ESI) m/z for $C_7H_7O^{18}O$ $[M+H]^+$: calcd. 125.0494, found 125.0494.

4. Synthesis of functionalized ^{18}O-4-(hydroxymethyl)phenols

^{18}O-4-((hydroxyl)methyl)phenyl acetate (17)

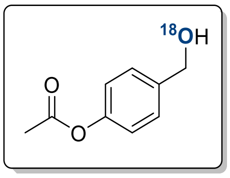

To a solution of ^{18}O-diol **16** (700 mg, 5.55 mmol) in THF (70 mL) was added Et_3N (620 mg, 850 µL, 6.11 mmol, 1.1 eq.) and then cooled to -40°C. Afterwards a solution of AcCl (440 mg, 400 µL, 5.55 mmol, 1.0 eq.) in THF (1.4 mL) was added dropwise by syringe pump (1.0 mL/h) at -40°C. Afterwards the reaction mixture was warmed to rt and stirred until turnover was complete. The solvent was removed under reduced pressure and the crude product was purified by flash chromatography (pentane:AcOEt, 2:1 to 1:1). The product (**17**, 750 mg, 4.48 mmol, 81%) was isolated as colorless gum.

The reaction was performed at 10 mmol scale (referred to product) using a similar procedure. In this case the product was isolated in 71% yield.

^{1}H-NMR (400 MHz, $CDCl_3$, δ/ppm): 7.42 – 7.33 (m, 2H), 7.12 – 7.03 (m, 2H), 4.67 (s, 2H), 2.30 (s, 3H), 1.77 (br. s, 1H). **^{13}C{^{1}H}-NMR** (101 MHz, $CDCl_3$, δ/ppm): 169.72, 150.20, 138.64, 128.21, 121.81, 64.86, 21.25. **HRMS** (ESI) m/z for $C_9H_{14}NO_2{}^{18}O$ $[M+NH_4^+]^+$: calcd. 186.1011, found 186.1019.

^{18}O-(4-methoxyphenyl)methanol-^{18}O (18)

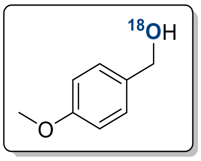

To a mixture of ^{18}O-diol **16** (420 mg, 3.33 mmol) and Cs_2CO_3 (1.30 g, 4.00 mmol, 1.2 eq.) in dry MeCN (15 mL) was added MeI (520 mg, 230 µL, 3.66 mmol, 1.1 eq.) dropwise at rt. The resulting mixture was stirred overnight at 70°C. After complete turnover (TLC-monitoring), the solvent was evaporated under reduced pressure and the crude product was purified by flash chromatography (pentane:AcOEt 3:1 to 2:1). The product (**18**, 340 mg, 2.45 mmol, 73%) was isolated as colorless gum.

The reaction was also performed at 11.9 mmol scale (referred to product) using a similar procedure. In this case the product was isolated in 79% yield.

^{1}H-NMR (400 MHz, $CDCl_3$, δ/ppm): 7.33 – 7.27 (m, 2H), 6.94 – 6.86 (m, 2H), 4.62 (s, 2H), 3.81 (s, 3H), 1.58 (br. s, 1H). **^{13}C{^{1}H}-NMR** (101 MHz, $CDCl_3$, δ/ppm): 159.38, 133.26, 128.80, 114.11, 65.21, 55.45. **HRMS** (ESI) m/z for $C_8H_{14}O^{18}ON$ [M+NH4^{+}]$^{+}$: calcd. 158.1062, found 158.1064.

4-(TIPSO)butanoic acid (SI-2)

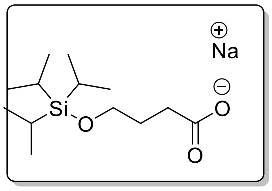

Sodium-4-hydroxybutanoate (3.00 g, 23.8 mmol) was suspended in dry DMF (30.0 mL) and imidazole (4.86 g, 71.4 mmol, 3.0 eq.) was added. Afterwards TIPSCl (4.59 g, 23.8 mmol, 1.0 eq.) was added dropwise and the reaction mixture was stirred at 65°C overnight. The solvent was removed under high vacuum and the crude product was purified by flash chromatography (DCM:MeOH 98:2 to 93:7). The product (**SI-2**, 2.63 g, 9.31 mmol, 39%) was isolated as colorless oil.

^{1}H-NMR (400 MHz, $CDCl_3$, δ/ppm): 3.75 (t, *J* = 6.0 Hz, 2H), 2.50 (t, *J* = 7.3 Hz, 2H), 1.87 (tt, *J* = 7.3, 5.9 Hz, 2H), 1.12 – 1.01 (m, 21H). **^{13}C{^{1}H}-NMR** (101 MHz, $CDCl_3$, δ/ppm): 179.48, 62.88, 31.36, 28.18, 18.43, 12.39. **HRMS** (ESI) m/z for $C_{13}H_{27}O_3Si$ [M-H]$^{-}$: calcd. 259.1735, found 259.1736.

^{18}O-4-(hydroxylmethyl)phenyl 4-(TIPSO)butanoate (19)

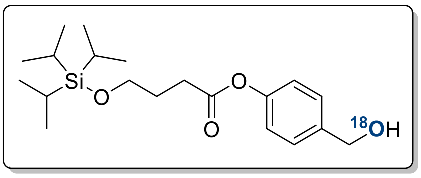

Sodium-4-((triisopropylsilyl)oxy)butanoate (**SI-2**, 600 mg, 2.13 mmol), ^{18}O-diol **16** (300 mg, 2.38 mmol, 1.1 eq.) and DMAP (29.0 mg, 246 µmol, 15 mol%) were dissolved in dry DMF (10.0 mL). Subsequently EDC (590 mg, 3.09 mmol, 1.5 eq.) was added to the solution and the mixture was stirred overnight at rt. Afterwards the solvent was removed under high vacuum and the crude product was purified by flash chromatography (pentane:AcOEt 4:1 to 2:1). The product (**19**, 330 mg, 890 µmol, 42%) was isolated as colorless gum.

^{1}H-NMR (400 MHz, $CDCl_3$, δ/ppm): 7.43 – 7.31 (m, 2H), 7.13 – 7.00 (m, 2H), 4.68 (s, 2H), 3.80 (t, *J* = 6.0 Hz, 2H), 2.70 (t, *J* = 7.2 Hz, 2H), 1.97 (tt, *J* = 7.3, 6.0 Hz, 2H), 1.70 – 1.64 (m, 1H), 1.13 – 1.00 (m, 21H). **^{13}C{^{1}H}-NMR** (101 MHz, $CDCl_3$, δ/ppm): 172.47, 150.33, 138.48, 128.20, 121.84, 64.94, 62.18, 30.89, 28.15, 18.16, 12.11. **HRMS** (ESI) m/z for $C_{20}H_{35}O_3{}^{18}OSi$ $[M+H]^+$: calcd. 369.2342, found 369.2358.

Synthesis of ^{18}O-4-((hydroxyl)methyl)phenyl 5-(Fmoc-amino)pentanoate (^{18}O-bigFM-alcohol) (20)

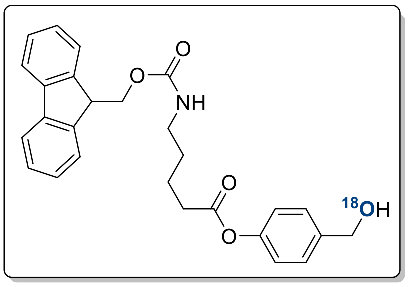

Fmoc-valeric acid (500 mg, 1.47 mmol), TBTU (570 mg, 1.76 mmol, 1.2 eq.) and DIPEA (1.14 g, 1.51 mL, 8.82 mmol, 6.0 eq.) were dissolved in dry DMF (10 mL) and stirred for 15 min at rt. Afterwards this solution was added dropwise to a solution of ^{18}O-diol **16** (183 mg, 1.47 mmol, 1.0 eq.) in dry DMF (5.0 mL) via syringe pump (8.0 mL/h). The reaction mixture was stirred overnight. Subsequently, the solvent was removed under high vacuum and the crude product was purified by flash chromatography (DCM:AcOEt 5:1 to 1:1). The product (**20**, 334 mg, 750 µmol, 51%) was isolated as white solid.
Rotameric signals are partially visible in ^{1}H-NMR.

^{1}H-NMR (400 MHz, $CDCl_3$, δ/ppm): 7.77 (dt, *J* = 7.5, 1.0 Hz, 2H), 7.63 – 7.55 (m, 2H), 7.44 – 7.35 (m, 4H), 7.31 (td, *J* = 7.5, 1.2 Hz, 2H), 7.12 – 7.03 (m, 2H), 4.83 (s, 1H), 4.69 (d, *J* = 5.5 Hz, 2H), 4.41 (d, *J* = 6.9 Hz, 2H), 4.21 (t, *J* = 6.9 Hz, 1H), 3.26 (q, *J* = 6.6 Hz, 2H), 2.60 (t, *J* = 7.3 Hz, 2H), 1.79 (p, *J* = 7.3 Hz, 2H), 1.74 – 1.59 (m, 3H). **^{13}C{^{1}H}-NMR** (101 MHz, $CDCl_3$, δ/ppm): 172.10, 156.58, 150.19, 144.12, 141.47, 138.64, 128.22, 127.81, 127.18, 125.17, 121.79, 120.11, 66.70, 64.90, 47.44, 40.71, 33.96, 29.52, 22.05. **HRMS** (ESI) m/z for $C_{27}H_{31}O_4N_2{}^{18}O$ $[M+NH_4]^+$: calcd. 465.2270, found 465.2279.

5. Synthesis of $^{18}O_2$-P-amidites

$^{18}O_2$–AB-P-amidite 27: $^{18}O_2$ – Bis(4-acetoxybenzyl)diisopropyl-P-amidite

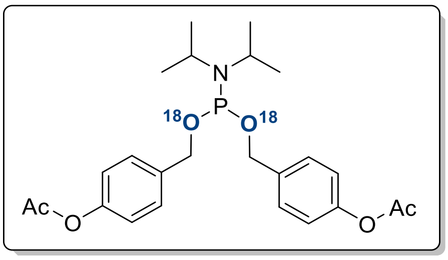

^{18}O-alcohol **17** (750 mg, 4.46 mmol, 2.0 eq.) was coevaporated with dry MeCN (2 x 5.0 mL) and dissolved in dry THF (10 mL). Subsequently, DIPEA (660 mg, 880 µL, 5.13 mmol, 2.3 eq.) was added followed by addition of (*i*Pr)$_2$N-PCl$_2$ (**21**, 450 mg, 410 µL, 2.23 mmol). The reaction was stirred for 3 h. After complete turnover (^{31}P-NMR), Et_2O (10.0 mL) was added. The precipitate was filtered off and washed with Et_2O. The filtrate was concentrated under reduced pressure and the resulting crude product was purified by flash chromatography (SiO_2 was deactivated with Et_3N, pentane:AcOEt 7:1 to 4:1). The product (**27**, 610 mg, 1.13 mmol, 59%) was isolated as colorless gum.

The reaction was performed at 7.1 mmol scale (referred to product) using a similar procedure. In this case the product was isolated in 69% yield.

^{1}H-NMR (400 MHz, $CDCl_3$, δ/ppm): 7.58 – 7.31 (m, 4H), 7.08 – 7.01 (m, 3H), 4.75 (dd, *J* = 12.6, 8.1 Hz, 2H), 4.67 (dd, *J* = 12.7, 8.6 Hz, 2H), 3.77 – 3.60 (m, 2H), 2.29 (s, 5H), 1.20 (d, *J* = 6.9 Hz, 10H). **^{13}C{^{1}H}-NMR** (101 MHz, $CDCl_3$, δ/ppm): 169.68, 149.95, 137.26 (d, *J* = 7.6 Hz), 128.15, 121.49, 64.93 (d, *J* = 18.4 Hz), 43.26 (d, *J* = 12.4 Hz), 24.80 (d, *J* = 7.2 Hz), 21.29. **^{31}P{^{1}H}-NMR** (162 MHz, $CDCl_3$, δ/ppm): 147.85. **HRMS** (ESI) m/z for $C_{24}H_{33}NO_4{}^{18}O_2P$ $[M+H]^+$: calcd. 466.2125, found 466.2154.

$^{18}O_2$–PMB-P-amidite 28: $^{18}O_2$-Bis(4-methoxybenzyl)diisopropylphosphoramidite

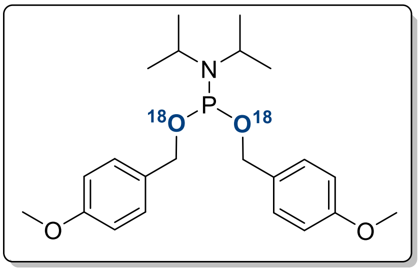

^{18}O-alcohol **18** (330 mg, 2.35 mmol, 2.0 eq.), Et_3N (400 mg, 540 µL, 3.89 mmol, 3.3eq.) were dissolved in Et_2O (5.0 mL). The resulting solution was added to a solution of (*i*Pr)$_2$N-PCl$_2$ (**21**,

240 mg, 220 µL, 1.18 mmol) in dry Et_2O (5.0 mL) at 0 °C via syringe pump (10 mL/h). The reaction mixture was stirred for 3 h at rt before the precipitate filtered off and washed with Et_2O. The filtrate was concentrated under reduced pressure and the crude product was purified by flash chromatography (SiO_2 was deactivated with Et_3N, pentane:Et_3N 60:1 to 20:1) The product (**28**, 350 mg, 850 µmol, 72%) was isolated as colorless gum.

The reaction was also performed at 3.50 mmol scale (referred to product) using a similar procedure. In this case the product was isolated in 60% yield.

^{1}H-NMR (400 MHz, $CDCl_3$, δ/ppm): 7.30 – 7.24 (m, 5H), 6.90 – 6.83 (m, 4H), 4.69 (dd, *J* = 12.2, 8.3 Hz, 2H), 4.61 (dd, *J* = 12.1, 8.8 Hz, 2H), 3.80 (s, 5H), 3.68 (dh, *J* = 10.0, 6.8 Hz, 2H), 1.20 (d, *J* = 6.8 Hz, 9H). **^{13}C{^{1}H}-NMR** (101 MHz, $CDCl_3$, δ/ppm): 159.04, 131.88 (d, *J* = 7.7 Hz), 128.72, 113.78, 65.22 (d, *J* = 18.2 Hz), 55.41, 43.19 (d, *J* = 12.3 Hz), 24.78 (d, *J* = 7.2 Hz). **HRMS** (ESI) m/z for $C_{22}H_{33}O_2{}^{18}O_2NP$ $[M+H]^+$: calcd. 410.2227, found 410.2245.

$^{18}O_2$–TIPS-P-amidite 31:

((((diisopropylamino)phosphanediyl)bis(oxy-*^{18}O*))bis(methylene))bis(4,1-phenylene) bis(4-((triisopropylsilyl)oxy)butanoate)

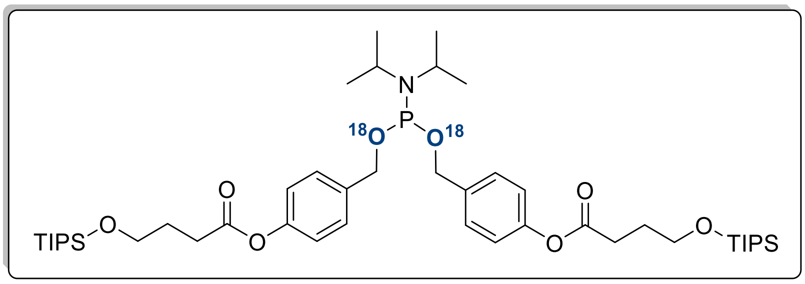

^{18}O-alcohol **19** (400 mg, 1.09 mmol, 2.0 eq.) was coevaporated with dry MeCN (2 x 5.0 mL) and then dissolved in dry THF (8.0 mL). DIPEA (160 mg, 215 µL, 1.24 mmol, 2.3 eq.) and (*i*Pr)$_2$N-PCl_2 (**21**, 110 mg, 100 µL, 540 µmol) were added and the solution was stirred for 5h at rt. After turnover was complete (^{31}P-NMR-monitoring), Et_2O (16 mL) was added the precipitate was filtered off. The filtrate was concentrated under reduced pressure (max. 30°C). The crude product was purified by flash chromatography (SiO_2 was deactivated with Et_3N, heptane:AcOEt:Et_3N 14:6:0.3). The product (**31**, 303 mg, 35.0 µmol, 65%) was isolated as colorless gum.

^{1}H-NMR (400 MHz, $CDCl_3$, δ/ppm): 7.40 – 7.30 (m, 4H), 7.11 – 6.99 (m, 4H), 4.75 (dd, *J* = 12.7, 8.1 Hz, 2H), 4.67 (dd, *J* = 12.6, 8.5 Hz, 2H), 3.80 (t, *J* = 6.0 Hz, 4H), 3.76 – 3.61 (m,

2H), 2.69z (t, J = 7.3 Hz, 4H), 1.97 (ddt, J = 7.8, 7.3, 6.0 Hz, 4H), 1.20 (d, J = 6.8 Hz, 12H), 1.15 – 0.97 (m, 43H). **^{13}C{^{1}H}-NMR** (101 MHz, $CDCl_3$, δ/ppm): 172.44, 150.04, 137.11 (d, J = 7.6 Hz), 128.14, 121.49, 64.97 (d, J = 18.4 Hz), 62.21, 43.26 (d, J = 12.3 Hz), 30.91, 28.19, 24.80 (d, J = 7.2 Hz), 18.17, 12.11. **^{31}P{^{1}H}-NMR** (162 MHz, $CDCl_3$, δ/ppm): 147.79. **HRMS** (ESI) m/z for $C_{46}H_{81}O_6{}^{18}O_2NPSi_2$ $[M+H]^+$: calcd.866.5318, found 866.5345.

$^{18}O_2$–bigFM-P-amidite 32:

((((diisopropylamino)phosphanediyl)bis(oxy-18O))bis(methylene))bis(4,1-phenylene) bis(5-(Fmoc-amino)pentanoate)

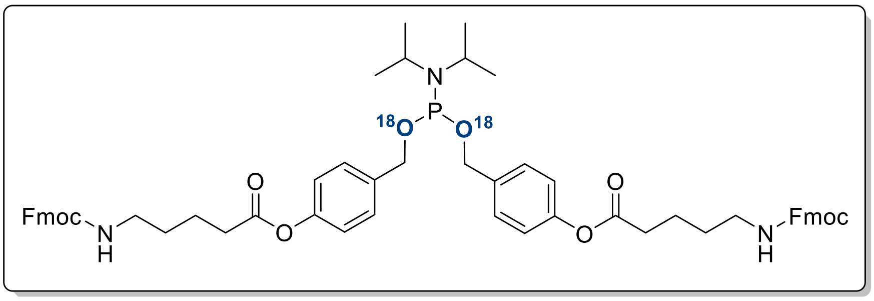

^{18}O-alcohol **20** (235 mg, 525 µmol, 2.0 eq.) was coevaporated with dry MeCN (2 x 5.0 mL) and then dissolved in dry THF (5.0 mL). DIPEA (80.0 mg, 105 µL, 604 µmol, 2.3 eq.) and (iPr)$_2$-N-PCl_2 (**21**, 53.0 mg, 48.0 µL, 262 µmol) were added and the mixture was stirred for 3 h at rt. After completion (^{31}P-NMR-monitoring), Et_2O (10 mL) was added and the precipitate was filtered off. The filtrate was concentrated under reduced pressure (max. 30°C). The crude product was purified by flash chromatography (SiO_2 was deactivated with Et_3N, pentane:AcOEt 9:1 to 4:1). The product (**32**, 174 mg, 170 µmol, 65%) was isolated as colorless gum.

^{1}H-NMR (400 MHz, $CDCl_3$, δ/ppm): 7.76 (dt, J = 7.5, 1.0 Hz, 4H), 7.59 (dq, J = 7.4, 0.9 Hz, 4H), 7.44 – 7.28 (m, 12H), 7.10 – 7.00 (m, 4H), 4.83 (br.s, 2H), 4.79 – 4.62 (m, 4H), 4.41 (d, J = 6.9 Hz, 4H), 4.21 (t, J = 6.9 Hz, 2H), 3.69 (dp, J = 10.0, 6.8 Hz, 2H), 3.25 (q, J = 6.6 Hz, 4H), 2.59 (t, J = 7.2 Hz, 4H), 1.78 (p, J = 7.4 Hz, 4H), 1.65 (q, J = 7.3 Hz, 4H), 1.55 (s, 2H), 1.20 (d, J = 6.8 Hz, 12H). **^{13}C{^{1}H}-NMR** (101 MHz, $CDCl_3$, δ/ppm): 172.09, 156.57, 149.92, 144.13, 141.48, 137.25 (d, J = 7.6 Hz), 128.17, 127.81, 127.18, 125.18, 121.45, 120.11, 66.70, 64.94 (d, J = 18.3 Hz), 47.45, 43.27 (d, J = 12.4 Hz), 40.72, 33.98, 32.08, 29.85, 29.81, 29.51, 24.80 (d, J = 7.2 Hz), 22.84, 22.07, 14.27. **^{31}P{^{1}H}-NMR** (162 MHz, $CDCl_3$, δ/ppm): 147.88. **HRMS** (ESI) m/z for $C_{60}H_{67}O_8{}^{18}O_2N_3P$ $[M+H]^+$: calcd. 1024.4643, found 1024.4648.

$^{18}O_2$-AB-P-diamidite 29: 4-(((bis(diisopropylamino)phosphaneyl)oxy)methyl)phenyl acetate

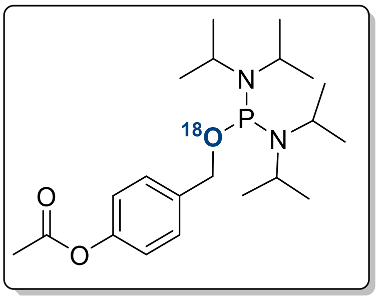

DIPEA (222 mg, 290 µL, 1.71 mmol, 1.2 eq.) and ^{18}O-alcohol **17** (240 mg, 1.43 mmol) were dissolved in dry DCM (3.0 mL). This mixture was added dropwise to a solution of ($i$$Pr_2N)_2$-PCl (**21**, 460 mg, 1.71 mmol, 1.2 eq.) in dry DCM (4.0 mL), that had been precooled to -78°C. Afterwards, the cooling bath was removed and the solution was stirred at rt for 1.5 h. The solution was evaporated under reduced pressure (max. 30°C) and the crude product was purified by flash chromatography (SiO_2 was deactivated with Et_3N, heptane:AcOEt:Et_3N 40:2:1 to 40:8:4). The product (**29**, 250 mg, 628 µmol, 44%) was isolated as colorless gum.

^{1}H-NMR (400 MHz, $CDCl_3$, δ/ppm): 7.41 – 7.32 (m, 2H), 7.08 – 7.00 (m, 2H), 4.63 (d, J = 7.2 Hz, 1H), 3.57 (dp, J = 10.8, 6.8 Hz, 4H), 2.29 (s, 3H), 1.19 (d, J = 2.5 Hz, 12H), 1.17 (d, J = 2.6 Hz, 12H). **^{13}C{^{1}H}-NMR** (101 MHz, $CDCl_3$, δ/ppm): 169.76, 149.64, 138.33 (d, J = 10.5 Hz), 127.91, 121.30, 65.70 (d, J = 23.3 Hz), 44.63 (d, J = 12.3 Hz), 24.78 (d, J = 8.0 Hz), 24.00 (d, J = 5.8 Hz), 21.30. **^{31}P{^{1}H}-NMR** (162 MHz, $CDCl_3$, δ/ppm): 123.36. **HRMS** (ESI) m/z for $C_{21}H_{38}O_2{}^{18}ON_2P$ $[M+H]^+$: calcd. 399.2657, found 399.2674.

$^{18}O_2$-AB-PMB-P-amidite 30:

4-(((diisopropylamino)((4-methoxybenzyl)oxy-^{18}*O*)phosphaneyl)oxy)methyl)phenyl acetate

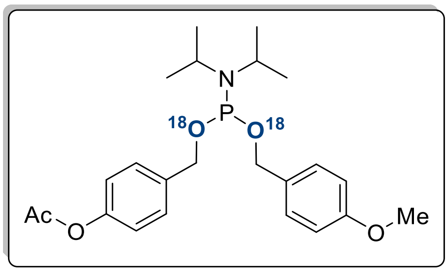

^{18}O-PMB-alcohol **18** (75.0 mg, 535 µmol) was coevaporated with dry MeCN (5.0 mL) and then dissolved in dry THF (3.0 mL). Afterwards ^{18}O-AB-Diamidite **29** (213 mg, 535 µmol, 1.0 eq.) and ETT (69.6 mg, 535 µmol, 1.0 eq.) were added and the reaction mixture was stirred for 1 h at rt. After complete turnover (^{31}P-NMR-monitoring), Et_2O (6.0 mL) was added, and the

precipitate was filtered off. The solvent was removed under reduced pressure (max. 30°C). The crude product was purified by flash chromatography (SiO_2 was deactivated with Et_3N, heptane:AcOEt:Et_3N 40:2:1 to 40:4:2). The product (**30**, 111 mg, 254 mmol, 47%) was isolated as colorless gum.

^{1}H-NMR (400 MHz, $CDCl_3$, δ/ppm): 7.37 – 7.31 (m, 2H), 7.30 – 7.26 (m, 2H), 7.08 – 7.01 (m, 2H), 6.90 – 6.84 (m, 2H), 4.83 – 4.53 (m, 4H), 3.80 (s, 3H), 3.68 (dh, *J* = 10.0, 6.8 Hz, 2H), 2.29 (s, 3H), 1.20 (d, *J* = 6.8 Hz, 12H). **^{13}C{^{1}H}-NMR** (101 MHz, $CDCl_3$, δ/ppm): 169.69, 159.08, 149.91, 137.37 (d, *J* = 7.7 Hz), 131.77 (d, *J* = 7.4 Hz), 128.73, 128.14, 121.44, 113.81, 65.29 (d, *J* = 18.5 Hz), 64.87 (d, *J* = 18.2 Hz), 55.42, 43.22 (d, *J* = 12.6 Hz), 24.83 (d, *J* = 3.6 Hz), 24.76 (d, *J* = 3.6 Hz), 21.29. **^{31}P{^{1}H}-NMR** (162 MHz, $CDCl_3$, δ/ppm): 147.43. **HRMS** (ESI) m/z for $C_{23}H_{33}O_3{}^{18}O_2NP$ $[M+H]^+$: calcd. 438.2103, found 438.2195.

^{18}O-DNA-precursor P-amidite (33):

4-((((((2R,3S,5R)-2-((bis(4-methoxyphenyl)(phenyl)methoxy)methyl)-5-(5-methyl-2,4-dioxo-3,4-dihydropyrimidin-1(2H)-yl)tetrahydrofuran-3-yl)oxy)(diisopropylamino)phosphaneyl)oxy)methyl)phenyl acetate

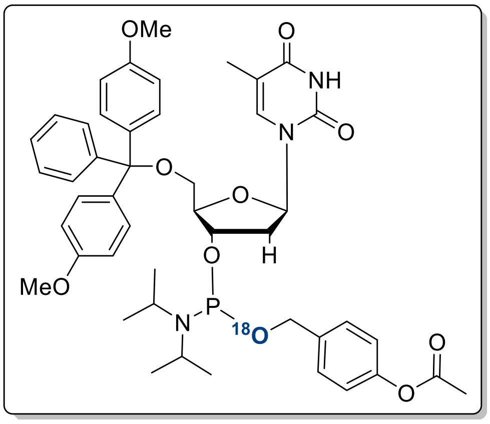

Diamidite **SI-1** (800.0 mg, 1.03 mmol, 1.05 eq.) was coevaporated with dry MeCN (5.0 mL) and dissolved in dry MeCN (5.0 mL). ^{18}O-AB-alcohol **17** (164.9 mg, 980 µmol) and ETT (115.9 mg, 980 µmol, 1.0 eq.) were added. The mixture was stirred for 45 min at room temperature. When turnover was complete, the solution was evaporated under reduced pressure (max. 30°C). The crude product was purified by flash chromatography (SiO_2 was deactivated with Et_3N, pentane:AcOEt:Et_3N 2:1:0.01 to 1:2:0.01). The product (**33**, 548.0 mg, 651 µmol, 66%) was isolated as colorless gum.

The compound was also synthesized from unlabeled ^{16}O-AB-alcohol. This reaction gave a similar result. NMR-data of the ^{16}O-product (**SI-3**) was indistinguishable. Notably, ^{1}H and ^{13}C-NMR data are complex due to the presence of a diastereomeric mixture.

^{1}H-NMR (400 MHz, $CDCl_3$, δ/ppm): 8.12 (s, 1H), 7.66 – 7.55 (m, 1H), 7.42 – 7.37 (m, 2H), 7.36 – 7.32 (m, 1H), 7.30 – 7.19 (m, 8H), 7.10 – 7.02 (m, 1H), 6.97 (d, *J* = 8.5 Hz, 1H), 6.86 – 6.76 (m, 3H), 6.43 – 6.34 (m, 1H), 4.74 – 4.58 (m, 2H), 4.57 – 4.48 (m, 1H), 4.20 – 4.12 (m, 1H), 3.78 (s, 3H), 3.78 – 3.76 (m, 3H), 3.67 – 3.53 (m, 2H), 3.50 – 3.41 (m, 1H), 3.35 – 3.26 (m, 1H), 2.54 – 2.44 (m, 1H), 2.34 – 2.21 (m, 4H), 1.45 – 1.38 (m, 3H), 1.20 – 1.02 (m, 12H). **^{13}C{^{1}H}-NMR** (101 MHz, $CDCl_3$, δ/ppm): 169.67, 169.63, 169.57, 163.60 (d, *J* = 1.8 Hz), 158.86, 150.25, 150.21, 150.08, 150.02, 144.49, 144.43, 138.65, 136.90, 136.82, 136.75, 135.87, 135.85, 135.58, 135.56, 135.49, 135.46, 130.30, 130.26, 128.33, 128.22, 128.19, 128.12, 127.27, 121.84, 121.62, 121.52, 113.39, 111.25, 111.22, 87.05, 85.97 (d, *J* = 4.0 Hz), 85.63 (d, *J* = 5.9 Hz), 84.97, 84.87, 73.80 (d, *J* = 17.5 Hz), 73.59 (d, *J* = 17.1 Hz), 65.14, 65.03, 64.96, 64.93, 64.85, 63.45, 63.35, 55.38 (d, *J* = 1.5 Hz), 43.41 (d, *J* = 7.8 Hz), 43.29 (d, *J* = 7.8 Hz), 40.36 – 40.25 (m), 29.85, 24.77, 24.75, 24.70, 24.64, 21.27, 11.86. **^{31}P{^{1}H}-NMR** (162 MHz, $CDCl_3$, δ/ppm): 148.56, 148.26.

*^{18}O-isotopologue (**33**):*

HRMS (ESI) m/z for $C_{46}H_{55}O_{9}{}^{18}ON_{3}P$ $[M+H]^+$: calcd. 842.3662, found 842.3675.

*^{16}O-isotopologue (**SI-3**):*

HRMS (ESI) m/z for $C_{46}H_{55}O_{10}N_{3}NaP$ $[M+Na]^+$: calcd. 862.3439, found 862.3441.

6. Synthesis of $^{18}O_n$ – phosphate natural products

6.1. Synthesis of $^{18}O_n$ – Nucleotides

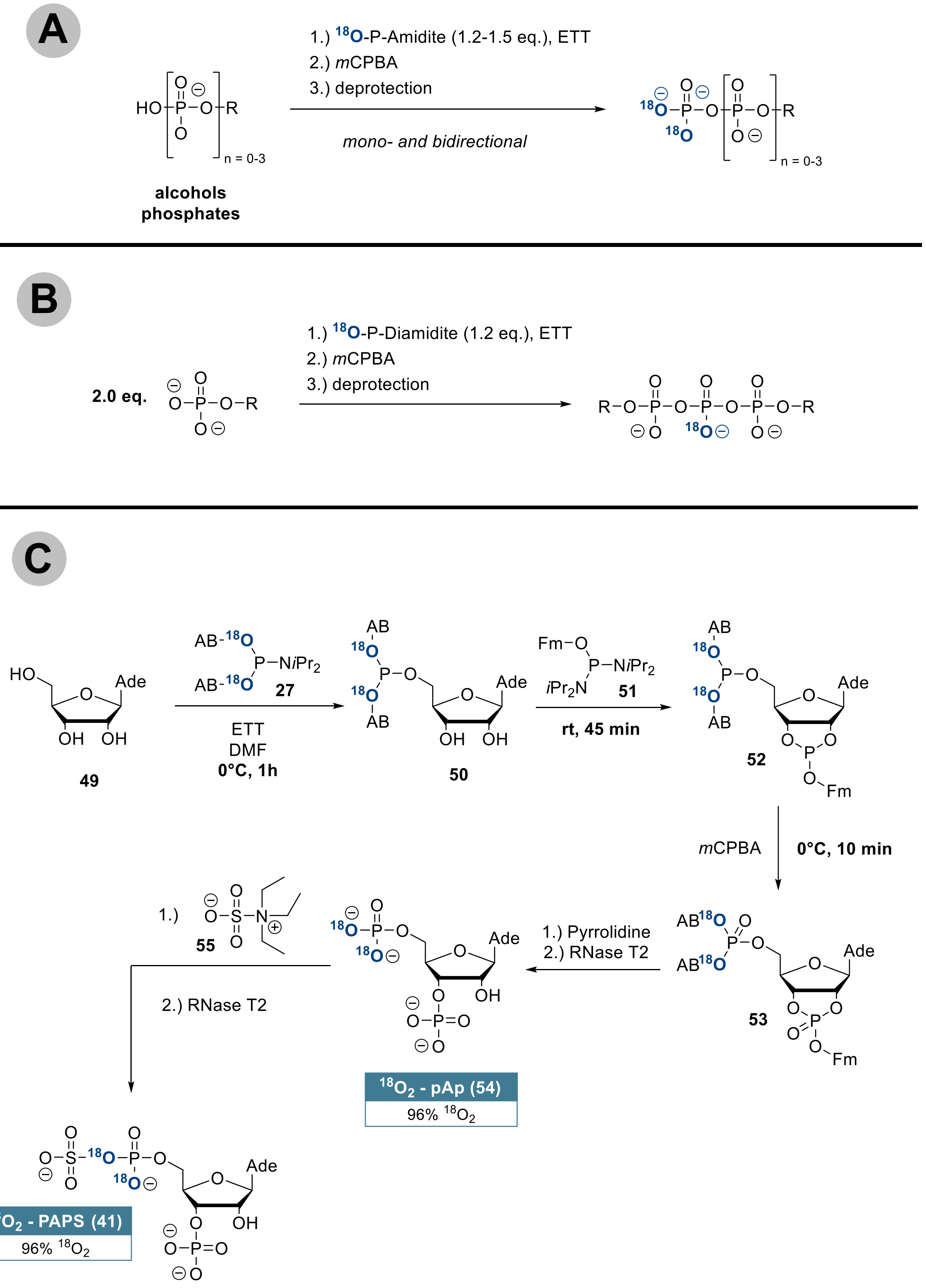

Supporting Figure 2: A: General synthetic strategy towards $^{18}O_2$ – nucleotides, terpenoidphosphates and polyphosphates. **B:** Homologative dimerization towards dinucleotides. **C:** Synthetic route towards selectively 5'-$^{18}O_2$-labeled PAPS (**41**).

$^{18}O_2$-Adenosine-5'-monophosphate ($^{18}O_2$-AMP, 34)

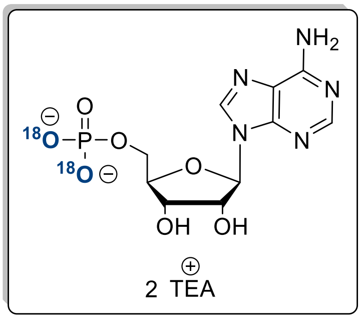

2',3'-isopropylideneadenosine (23.1 mg, 75.0 µmol) and ETT (29.4 mg, 226 µmol, 3.0 eq.) were coevaporated with dry MeCN (2 x 5.0 mL) and dissolved in dry DMF (2.0 mL). Then $^{18}O_2$-PMP-P-amidite **28** (39.5 mg, 98.0 µmol, 1.3 eq.) was added and the mixture was stirred for 1 h at rt. Subsequently, the reaction mixture was cooled to 0°C and *m*CPBA (77%, 31.2 mg, 140 µmol, 1.9 eq.) was added. The solution was stirred for 15 min at 0°C and the solvent was removed under high vacuum. The resulting oil was dissolved in DCM (1.5 mL). Then, TFA (85.9 mg, 58.1µL, 750 µmol, 10.0 eq.) was added and the solution was stirred for 3 h (reaction monitoring by ^{31}P-NMR). Precipitation was induced by the addition of Et_2O (40 mL). The precipitate was separated by centrifugation, washed with Et_2O (40 mL) and dried under high vacuum. The resulting gum was dissolved in H_2O (1.5 mL) the solution was acidified with aq. HCl (5 M) to pH 1.5. The solution was incubated at 37°C overnight. Subsequently, the crude product was purified by automated RP-MPLC (Interchim, C18-AQ, H_2O/MeCN/TEAA-buffer [10 mM]). The product containing fractions were lyophilized and the product (**34**, 38.5 mg, 58.7 µmol, 78%) was isolated as white solid and TEA-salt.
The reaction was performed at 1.98 mmol scale (= 1.19 g of $^{18}O_2$-ATP x 4 Na^+) using a similar procedure. In this case the product was isolated in 81% yield.

^{1}H-NMR (400 MHz, D_2O, δ/ppm): 8.52 (s, 1H), 8.26 (s, 1H), 6.14 (d, J = 5.9 Hz, 1H), 4.77 – 4.74 (m, 1H), 4.50 (dd, J = 5.1, 3.5 Hz, 1H), 4.40 – 4.36 (m, 1H), 4.12 – 4.08 (m, 2H), 3.21 (q, J = 7.2 Hz, 15H), 1.28 (td, J = 7.3, 0.3 Hz, 23H). **^{13}C{^{1}H}-NMR** (101 MHz, D_2O, δ/ppm): 155.59, 152.79, 149.08, 139.90, 118.62, 86.86, 84.24 (d, J = 8.9 Hz), 74.35, 70.49, 64.14 (d, J = 5.0 Hz), 46.65, 8.19. **^{31}P{^{1}H}-NMR** (162 MHz, D_2O, δ/ppm): 1.07. **HRMS** (ESI) m/z for $C_{10}H_{13}O_5{}^{18}O_2N_5$ $[M+H]^-$: calcd. 350.0643, found 350.0657.

$^{18}O_2$-Guanosine-5'-monophosphate ($^{18}O_2$-GMP, 6)

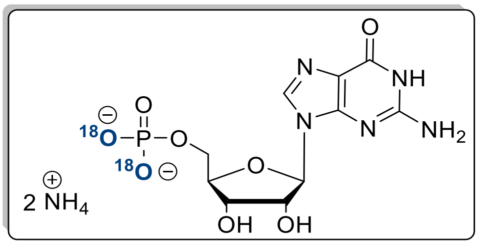

2',3'-isopropylideneguanosine (24.4 mg, 75.0 µmol) and ETT (29.4 mg, 226 µmol, 3.0 eq.) were coevaporated with dry MeCN (2 x 5.0 mL) and dissolved in a mixture of dry DMSO (0.4 mL) and dry DMF (1.6 mL). Then $^{18}O_2$-PMB-P-amidite **28** (39.5 mg, 98.0 µmol, 1.3 eq.) was added and the mixture was stirred for 1 h at rt. Afterwards the solution was cooled to 0°C and TBHP (5.5 M in decanes, 27.4 µL, 151 µmol, 2.0 eq.) was added. After stirring for 1 h at rt, the solvent was removed under high vacuum. The residue was dissolved in DCM (4.0 mL) and TFA (85.9 mg, 58.1 µL, 750 µmol, 10.0 eq.) was added. The reaction mixture was stirred for 3 h (reaction monitoring by ^{31}P-NMR). Subsequently precipitation was induced by the addition of Et_2O (40 mL). The precipitate was separated by centrifugation, washed with Et_2O (40 mL) and dried under high vacuum. The crude product was dissolved in H_2O (2.0 mL) and acidified with to pH=1.5 using aq. HCl (5 M). The solution was incubated at 37°C overnight. The crude product was purified by automated SAX (Äkta pure, Q-sepharose, NH_4HCO_3-buffer). The product containing fractions were lyophilized and the product (**6**, 25.5 mg, 64.2 µmol, 86%) was isolated as white solid and NH_4-salt.

^{1}H-NMR (400 MHz, D_2O, δ/ppm): 8.16 (s, 1H), 5.88 (d, J = 5.8 Hz, 1H), 4.74 – 4.70 (m, 1H), 4.45 (dd, J = 5.2, 3.6 Hz, 1H), 4.29 (qd, J = 3.6, 1.3 Hz, 1H), 4.09 – 3.88 (m, 2H). **^{13}C{^{1}H}-NMR** (101 MHz, D_2O, δ/ppm): 160.27, 153.98, 151.60, 137.57, 116.04, 86.67, 84.44 (d, J = 8.7 Hz), 73.99, 70.58, 63.45 (d, J = 4.6 Hz). **^{31}P{^{1}H}-NMR** (162 MHz, D_2O, δ/ppm): 3.62. **HRMS** (ESI) m/z for $C_{10}H_{13}O_6{}^{18}O_2N_5P$ $[M-H]^-$: calcd. 366.0592, found 366.0608.

β-$^{18}O_2$-Adenosine-5'-diphosphate (β-$^{18}O_2$-ADP, 35)

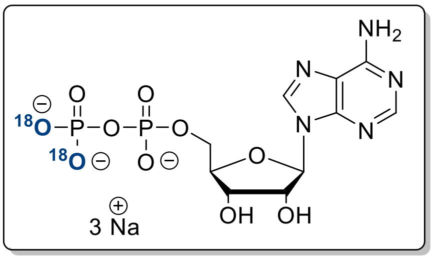

AMP x 2.0 TBA (74.2 mg, 90.0 µmol) and ETT (34.9 mg, 269 µmol, 3.0 eq.) were dissolved in dry DMF (2.0 mL) and $^{18}O_2$-AB-P-amidite (**27**, 50.0 mg, 108.0 µmol, 1.2 eq.) was added. The solution was stirred for 20 min at rt. Afterwards the reaction mixture was cooled to 0°C and *m*CPBA (77%, 33.4 mg, 152 µmol, 1.7 eq.) was added. The resulting solution was stirred for 15 min at 0°C before Et_2O (40 mL) was added. The resulting precipitate was separated by centrifugation, washed with Et_2O (40 mL) and dried under high vacuum. The resulting solid was dissolved in dry DMSO (2.0 mL) and pyrrolidine (400 µL) was added. The solution was stirred overnight at rt. Afterwards, the crude product was precipitated by the addition of Et_2O (40 mL). The precipitate was separated by centrifugation, washed with Et_2O (40 mL) and dried under high vacuum. The crude product was purified by automated SAX (Äkta pure, Q-Sepharose, $NaClO_4$-buffer). The product containing fractions (ca. 100 mM) were lyophilized. The resulting solid was washed with acetone (2 x 40 mL), separated by centrifugation and dried under high vacuum. The product (**35**, 40.8 mg, 82.1 µmol, 91%) was isolated as white solid and Na-salt.

^{1}H-NMR (400 MHz, D_2O, δ/ppm): 8.54 (s, 1H), 8.25 (s, 1H), 6.15 (d, *J* = 5.2 Hz, 1H), 4.77 (t, *J* = 5.2 Hz, 1H), 4.64 (ddd, *J* = 5.2, 4.4, 0.5 Hz, 1H), 4.42 – 4.36 (m, 1H), 4.28 (ddd, *J* = 11.8, 6.4, 3.0 Hz, 1H), 4.21 (ddd, *J* = 11.8, 4.7, 3.1 Hz, 1H). **^{13}C{^{1}H}-NMR** (101 MHz, D_2O, δ/ppm): 155.62, 152.82, 148.99, 139.93, 118.63, 87.00, 83.77 (d, *J* = 9.0 Hz), 74.30, 69.83, 64.32 (d, *J* = 5.2 Hz). **^{31}P{^{1}H}-NMR** (162 MHz, D_2O, δ/ppm): **HRMS** (ESI) m/z for $C_{10}H_{14}O_8{}^{18}O_2N_5P_2$ $[M-H]^-$: calcd. 430.0306, found 430.0318.

γ-$^{18}O_2$-Adenosine-5'-triphosphate (γ-$^{18}O_2$-ATP, 36)

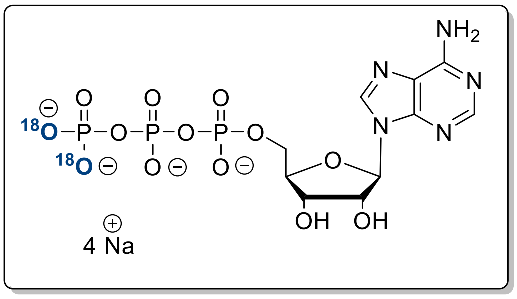

ADP x 1.55 TBA (60.1 mg, 75.0 µmol) and ETT (29.4 mg, 226 µmol, 3.0 eq.) were dissolved in dry DMF (2.0 mL). Then $^{18}O_2$-AB-P-amidite **27** (42.0 mg, 90.0 µmol, 1.2 eq.) was added and the solution was stirred for 15 min at rt. Subsequently, the mixture was cooled to 0°C and *m*CPBA (77%, 31.2 mg, 140 µmol, 1.9 eq.) was added. The solution was stirred for 15 min at 0°C. Afterwards, precipitation was induced by the addition of Et_2O (40 mL). The precipitate

was separated by centrifugation, washed with Et_2O (40 mL) and dried under high vacuum. Then the resulting solid was dissolved in dry DMSO (2.0 mL). Pyrrolidine (400 µL) was added and the mixture was stirred overnight. The crude product was precipitated by the addition of Et_2O (40 mL). The precipitate was separated by centrifugation, washed with Et_2O (40 mL) and dried under high vacuum. Subsequently, the crude product was purified by automated SAX (Äkta pure, Q-Sepharose, $NaClO_4$ – buffer). The product containing fractions (ca. 100 mM) were lyophilized. The resulting solid was washed with acetone (2 x 20.0 mL), separated by centrifugation and dried under high vacuum. The product (**36**, 25.4 mg, 42.4 µmol, 56%) was isolated as white solid and Na-salt.

The reaction was performed at 1.98 mmol scale (= 1.19 g of ATP x 4 Na^+) using a similar procedure. In this case the product was isolated in 81% yield.

^{1}H-NMR (400 MHz, D_2O, δ/ppm): 8.56 (s, 1H), 8.26 (s, 1H), 6.15 (d, *J* = 6.0 Hz, 1H), 4.87 – 4.81 (m, 1H), 4.65 (dd, *J* = 5.1, 3.5 Hz, 1H), 4.40 (q, *J* = 2.9 Hz, 1H), 4.31 (dd, *J* = 11.8, 2.7 Hz, 1H), 4.20 (dd, *J* = 11.8, 2.9 Hz, 1H). **^{13}C{^{1}H}-NMR** (101 MHz, D_2O, δ/ppm): 155.67, 152.85, 149.21, 139.97, 118.63, 86.51, 84.08 (d, *J* = 8.9 Hz), 74.16, 70.21, 64.98 (d, *J* = 5.7 Hz). **^{31}P{^{1}H}-NMR** (162 MHz, D_2O, δ/ppm): -5.89 (d, *J* = 20.0 Hz), -11.06 (d, *J* = 19.3 Hz), -21.77. **HRMS** (ESI) m/z for $C_{10}H_{15}O_{11}{}^{18}O_2N_5P_3$ $[M-H]^-$: calcd. 509.9970, found 509.9975.

γ-^{18}O$_2$-Guanosine-5'-triphosphate (γ-^{18}O$_2$-GTP, 37)

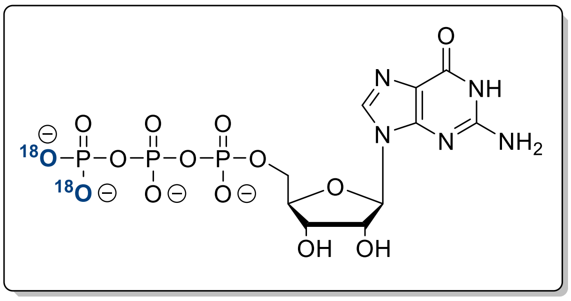

GDP x 1.95 TBA (68.1 mg, 75.0 µmol) and ETT (29.1 mg, 224 µmol, 3.0 eq.) were dissolved in dry DMF (2.0 mL). Then $^{18}O_2$-AB-P-amidite **27** (52.0 mg, 112 µmol, 1.5 eq.) was added and the mixture was stirred for 20 min at rt. Afterwards, the reaction mixture was cooled to 0°C and *m*CPBA (77%, 31.2 mg, 140 µmol, 1.9 eq.) was added and stirred for 15 min at 0°C. Then, precipitation was induced by addition of Et_2O (40 mL). The precipitate was separated by centrifugation, washed with Et_2O (40 mL) and dried under high vacuum. The resulting solid was dissolved in dry DMSO (2.0 mL). Pyrrolidine (400 µL) was added and the solution was stirred overnight. Subsequently, the crude product was precipitated by the addition of Et_2O (40

mL). The precipitate was separated by centrifugation, washed with Et_2O (40 mL) and dried under high vacuum. The crude product was purified by automated SAX (Äkta pure, Q-Sepharose, $NaClO_4$ – buffer). The product containing fractions (ca 100 mM) were lyophilized. The resulting solid was washed with acetone (2 x 20 mL), separated by centrifugation, and dried under high vacuum. The product (**37**, 25.3 mg, 41.1 µmol, 55%) was isolated as white solid and Na-salt.

^{1}H-NMR (400 MHz, D_2O, δ/ppm): 8.15 (s, 1H), 5.93 (d, *J* = 6.3 Hz, 1H), 4.87 – 4.81 (m, 1H), 4.63 (dd, *J* = 5.2, 3.3 Hz, 1H), 4.36 (qd, *J* = 3.3, 2.1 Hz, 1H), 4.29 (ddd, *J* = 11.7, 6.7, 3.2 Hz, 1H), 4.20 (ddd, *J* = 11.6, 5.0, 3.4 Hz, 1H). **^{13}C{^{1}H}-NMR** (101 MHz, D_2O, δ/ppm): 159.35, 154.19, 151.89, 137.75, 116.30, 86.53, 83.98 (d, *J* = 8.9 Hz), 73.48, 70.22, 65.04 (d, *J* = 5.5 Hz). **^{31}P{^{1}H}-NMR** (162 MHz, D_2O, δ/ppm): -6.18, -11.15 (d, *J* = 20.0 Hz), -21.96 (t, *J* = 20.0 Hz). **HRMS** (ESI) m/z for $C_{10}H_{15}O_{12}{}^{18}O_2\,N_5P_3$ [M-H]$^-$: calcd. 525.9919, found 525.9917.

γ-$^{18}O_2$-Uridine-5'-triphosphate (γ-$^{18}O_2$-UTP, 38)

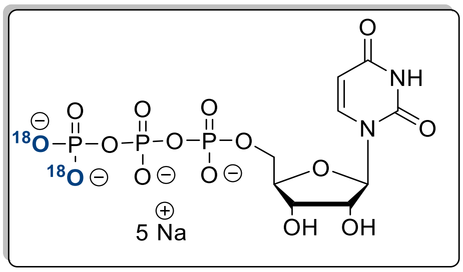

UDP x 1.62 TBA (59.2 mg, 75.0 µmol) and ETT (29.1 mg, 224 µmol, 3.0 eq.) were dissolved in dry DMF (2.0 mL). Then $^{18}O_2$-AB-P-amidite **27** (52.0 mg, 112 µmol, 1.5 eq.) was added and the mixture was stirred for 20 min at rt. Afterwards, the solution was cooled to 0°C and *m*CPBA (77%, 31.2 mg, 140 µmol, 1.9 eq.) was added. The solution was stirred for 15 min at 0°C. Subsequently, precipitation was induced by the addition of Et_2O (40 mL). The precipitate was separated by centrifugation, washed with Et_2O (40 mL) and dried under high vacuum. Afterwards, the solid was dissolved in dry DMSO (2.0 mL). Then, pyrrolidine (400 µL) was added and the solution was stirred overnight. The crude product was precipitated by the addition of Et_2O (40 mL). The precipitate was separated by centrifugation, washed with Et_2O (40 mL) and dried under high vacuum. Subsequently, the crude product was purified by automated SAX (Äkta pure, Q-Sepharose, $NaClO_4$ – buffer). The product containing fractions (ca. 100 mM) were lyophilized. The resulting solid was washed with acetone (2 x 20 mL), separated by centrifugation and dried under high vacuum. The product (**38**, 26.9 mg, 46.7 µmol, 62%) was isolated as white solid and Na-salt.

^{1}H-NMR (400 MHz, D_2O, δ/ppm): 7.95 (d, *J* = 8.0 Hz, 1H), 6.02 (d, *J* = 5.2 Hz, 1H), 5.95 (d, *J* = 8.0 Hz, 1H), 4.48 (dd, *J* = 5.3, 4.2 Hz, 1H), 4.41 (t, *J* = 5.3 Hz, 1H), 4.32 – 4.20 (m, 3H). **^{13}C{^{1}H}-NMR** (101 MHz, D_2O, δ/ppm): 141.33, 102.78, 88.20, 83.24, 83.15, 73.69, 69.42, 64.77, 64.72. **^{31}P{^{1}H}-NMR** (162 MHz, D_2O, δ/ppm): -5.99 (d, *J* = 20.1 Hz), -11.18 (d, *J* = 19.6 Hz), -21.92 (t, *J* = 19.8 Hz). **HRMS** (ESI) m/z for $C_9H_{14}O_{13}{}^{18}O_2N_2P_3$ [M-H]$^-$: calcd. 486.9697, found 486.9705.

δ-$^{18}O_2$-Adenosine-5'-tetraphosphate (δ-$^{18}O_2$-Ap4, 39)

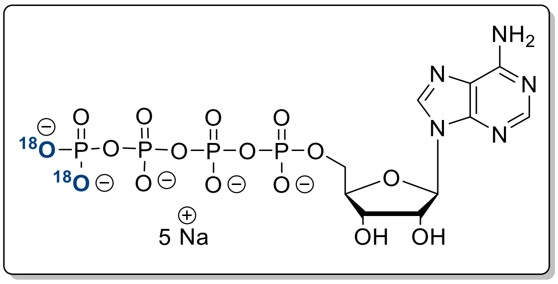

ATP x 2.59 TBA (84.9 mg, 75.0 µmol) and ETT (29.4 mg, 226 µmol, 3.0 eq.) were dissolved in dry DMF (2.0 mL). Afterwards, $^{18}O_2$-PMB-P-amidite **39** (42.0 mg, 90.0 µmol, 1.2 eq.) was added and the mixture was stirred for 15 min at rt. The solution was cooled to 0°C and *m*CPBA (77%, 31.2 mg, 140 µmol, 1.9 eq.) was added. The mixture was stirred for 15 min at 0°C. Precipitation was induced by the addition of Et_2O. The precipitate was separated by centrifugation, washed with Et_2O (40 mL) and dried under high vacuum. Afterwards, the precipitate was dissolved in dry DMSO (2.0 mL). Then pyrrolidine (400 µL) was added and the resulting solution was stirred overnight. The crude product was precipitated by the addition of Et_2O (40 mL). The precipitate was separated by centrifugation, washed with Et_2O (40 mL) and dried under high vacuum. Subsequently, the crude product was purified by automated SAX (Äkta pure, Q-Sepharose, $NaClO_4$ – buffer). The product containing fractions (ca. 100 mM) were lyophilized. The resulting solid was washed with acetone (2 x 20 mL), separated by centrifugation and dried under high vacuum. The product (**39**, 28.9 mg, 41.2 µmol, 55%) was isolated as white solid and Na-salt.

^{1}H-NMR (400 MHz, D_2O, δ/ppm): 8.57 (s, 1H), 8.27 (s, 1H), 6.14 (d, *J* = 6.7 Hz, 1H), 4.89 – 4.83 (m, 1H), 4.67 (dd, *J* = 5.2, 2.6 Hz, 1H), 4.42 (ddt, *J* = 2.8, 2.8, 2.8 Hz, 1H), 4.31 (ddd, *J* = 11.6, 6.1, 2.6 Hz, 1H), 4.21 – 4.14 (m, 1H). **^{13}C{^{1}H}-NMR** (101 MHz, D_2O, δ/ppm): 155.70, 152.88, 149.35, 139.95, 118.63, 86.31, 84.61 (d, *J* = 9.3 Hz), 74.16, 70.54, 65.42 (d, *J* = 5.6 Hz). **^{31}P{^{1}H}-NMR** (162 MHz, D_2O, δ/ppm): -5.71 (d, *J* = 19.0 Hz), -11.31 (d,

J = 19.1 Hz), -21.55 (dd, *J* = 19.1, 15.9 Hz), -22.45 (dd, *J* = 19.0, 15.7 Hz). **HRMS** (ESI) m/z for $C_{10}H_{16}O_{14}{}^{18}O_2N_5P_4$ $[M-H]^-$: calcd. 589.9633, found 589.9637.

^{18}O-Diadenosinetriphosphate (β-^{18}O-Ap3A, 40)

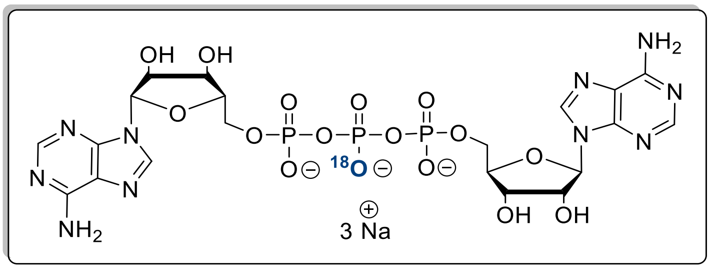

AMP x 2.00 TBA (124 mg, 150 µmol, 2.0 eq.) and ETT (68.1 mg, 524 µmol, 3.5 eq.) were dissolved in dry DMF (2.0 mL). Then, ^{18}O-P-diamidite **29** (44.7 mg, 112 µmol, 1.5 eq.) was added and the mixture was stirred for 15 min at rt. Afterwards, the solution was cooled to 0°C and *m*CPBA (77%, 73.5 mg, 329 µmol, 2.2 eq.) was added. The solution was stirred for 15 min at 0°C before precipitation was induced by the addition of Et_2O:pentane (5:1, 40 mL). The precipitate was separated by centrifugation, washed with Et_2O (40 mL) and dried under high vacuum. Afterwards the precipitate was dissolved in dry DMSO (2.0 mL). Then, pyrrolidine (400 µL) was added and the solution was stirred for 1 h. The crude product was precipitated by the addition of Et_2O (40 mL). The precipitate was separated by centrifugation, washed with Et_2O (40 mL) and dried under high vacuum. Subsequently, the crude product was purified by automated SAX (Äkta pure, Q-Sepharose, $NaClO_4$ – buffer). The product containing fractions (ca. 100 mM) were lyophilized. The resulting solid was washed with acetone (2 x 20 mL), separated by centrifugation and dried under high vacuum. The product (**40**, 31.7 mg, 38.5 µmol, 51%) was isolated as white solid and Na-salt.

^{1}H-NMR (400 MHz, D_2O, δ/ppm): 8.29 (s, 2H), 8.10 (s, 2H), 6.00 (d, *J* = 4.6 Hz, 1H), 4.59 (t, *J* = 4.8 Hz, 2H), 4.49 (t, *J* = 4.8 Hz, 2H), 4.37 – 4.25 (m, 6H). **^{13}C{^{1}H}-NMR** (101 MHz, D_2O, δ/ppm): 154.93, 152.59, 148.17, 139.04, 117.86, 87.20, 83.03 (d, *J* = 9.4 Hz), 74.86, 69.59, 64.61 (d, *J* = 5.5 Hz). **^{31}P{^{1}H}-NMR** (162 MHz, D_2O, δ/ppm): -11.59 (d, *J* = 19.5 Hz), -23.07 (t, *J* = 19.5 Hz). **HRMS** (ESI) m/z for $C_{20}H_{26}O_{15}{}^{18}ON_{10}P_3$ $[M-H]^-$: calcd. 757.0789, found 757.0816.

5'-$^{18}O_2$-Adenosine-3'-5'-bisphosphate (5'-$^{18}O_2$-pAp, 53)

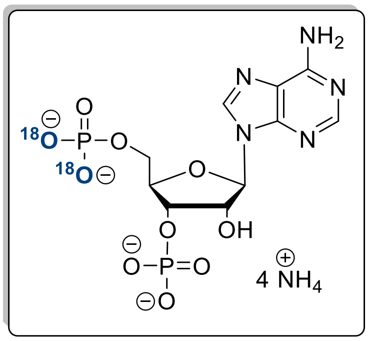

Adenosine (20.0 mg, 75.0 µmol) and ETT (48.7 mg, 375 µmol, 5.0 eq.) were coevaporated with dry MeCN (2 x 5.0 mL) and then dissolved in dry DMF (1.5 mL). The mixture was cooled to 0°C. Subsequently, a solution of $^{18}O_2$-AB-P-amidite **27** (60.9 mg, 131 µmol, 1.75 eq.) in dry DMF (1.0 mL, 0 °C) was added dropwise. The resulting solution was stirred for 1h at 0°C. Afterwards Fm-Diamidite **51** (41.5 mg, 97.3 µmol, 1.3 eq.) was added and the mixture was stirred for 45 min at rt. The solution was cooled to 0°C and *m*CPBA (77%, 55.2 mg, 247 µmol, 3.3 eq.) was added. The solution was stirred for 15 min at 0°C. Subsequently, pyrrolidine (700 µL) was added and the solution was stirred overnight at rt. Precipitation was induced by the addition of Et_2O (40 mL). The precipitate was separated by centrifugation, washed with Et_2O (40 mL) and dried under high vacuum. The residue was dissolved in H_2O (5.0 mL) and Rnase T2 (40 µL) was added. The solution was incubated overnight at 37 °C and then directly applied to automated SAX-purification (Äkta pure, Q-Sepharose, NH_4HCO_3 – buffer). The product containing fractions (ca. 200-300 mM) were lyophilized. The product (**53**, 16.5 mg, 33.1 µmol, 44%) was isolated as white solid and NH_4-salt.

^{1}H-NMR (400 MHz, D_2O, δ/ppm): 8.53 (s, 1H), 8.28 (s, 1H), 6.19 (d, *J* = 5.4 Hz, 1H), 4.88 – 4.82 (m, 2H), 4.59 (p, *J* = 2.6 Hz, 1H), 4.18 (dd, *J* = 5.0, 2.9 Hz, 2H). **^{13}C{^{1}H}-NMR** (101 MHz, D_2O, δ/ppm): 154.24, 150.96, 148.92, 140.32, 118.57, 86.83, 83.31 (dd, *J* = 8.7, 3.7 Hz), 73.80 (d, *J* = 4.9 Hz), 64.22 (d, *J* = 4.8 Hz). **^{31}P{^{1}H}-NMR** (162 MHz, D_2O, δ/ppm): 0.19, 0.05. **HRMS** (ESI) m/z for $C_{10}H_{14}O_8{}^{18}O_2N_5P_2$ $[M-H]^-$: calcd. 430.0306, found 430.0308.

Adenosine-3'-phosphat-5'-α-$^{18}O_2$-phosphosulfate (5'-$^{18}O_2$-PAPS, 41)

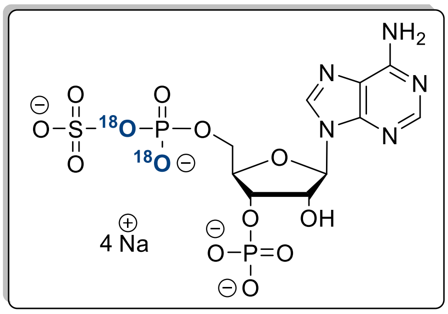

pAp x 4.8 TBA (22.0 mg, 14 µmol) was dissolved in a dry mixture of DMF:pyridine (14:1, 1.0 mL). Subsequently, triethylammoniumsulfate (13.0 mg, 30.5 µmol, 2.2 eq.) was added and the solution was stirred for 45 min. Afterwards, the product was precipitated by the addition of Et_2O (20 mL). The precipitate was separated by centrifugation, washed with Et_2O (20 mL) and dried under high vacuum. The crude product was dissolved in H_2O (4.0 mL) and Rnase T2 (40 µL) was added. The solution was incubated overnight at rt and directly applied to automated SAX-purification (Äkta pure, Q-Sepharose, $NaClO_4$ – buffer). The product containing fractions (ca. 100 mM) were lyophilized. The resulting solid was washed with acetone (2 x 20 mL), separated by centrifugation, and dried under high vacuum. The product (**41**, 2.10 mg, 4.05 µmol, 29%) was isolated as white solid and Na-salt.

^{1}H-NMR (400 MHz, D_2O, δ/ppm): 8.56 (s, 1H), 8.27 (s, 1H), 6.19 (d, *J* = 7.1 Hz, 1H), 4.59 (dt, *J* = 5.0, 2.4 Hz, 2H), 4.34 – 4.19 (m, 3H). **^{13}C{^{1}H}-NMR** (101 MHz, D_2O, δ/ppm): 155.67, 152.87, 149.41, 139.98, 118.60, 86.47, 84.06 (dd, *J* = 3.8 Hz), 74.52 (d, *J* = 3.4 Hz), 73.77 (d, *J* = 4.7 Hz), 66.19 (d, *J* = 5.7 Hz). **^{31}P{^{1}H}-NMR** (162 MHz, D_2O, δ/ppm): 3.79, -10.61. **HRMS** (ESI) m/z for $C_{10}H_{14}O_{11}{}^{18}O_2N_5P_2S$ $[M-H]^-$: calcd. 509.9874, found 509.9896.

6.2. Synthesis of $^{18}O_n$ – Polyphosphates

$^{18}O_4$-Tetraphosphate ($^{18}O_4$–P_4, 42)

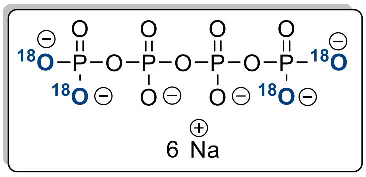

PPi x 1.90 TBA (47.6 mg, 75.0 µmol) and ETT (39.0 mg, 300 µmol, 4.0 eq.) were dissolved in dry DMF (2.0 mL). Then $^{18}O_2$-PMB-P-amidite **27** (87.2 mg, 188 µmol, 2.5 eq.) was added and the mixture was stirred for 15 min at rt. Afterwards, the solution was cooled to 0°C and *m*CPBA (77%, 55.3 mg, 247 µmol, 3.3 eq.) was added. The reaction mixture was stirred for 15 min at 0°C. Subsequently, precipitation was induced by addition of Et_2O (40 mL). The precipitate was separated by centrifugation, washed with Et_2O (40 mL) and dried under high vacuum. Afterwards the precipitate was dissolved in dry DMSO (2.0 mL). Pyrrolidine (400 µL) was added and the solution was stirred overnight. The crude product was precipitated by the addition of Et_2O (40 mL). The precipitate was separated by centrifugation, washed with Et_2O (40 mL) and dried under high vacuum. Subsequently, the crude product was purified by automated SAX (Äkta pure, Q-Sepharose, $NaClO_4$ – buffer). The product containing fractions (ca. 100 mM) were lyophilized. The resulting solid was washed with acetone (2 x 20 mL), separated by centrifugation and dried under high vacuum. The product (**42**, 11.6 mg, 24.3 µmol, 32%) was isolated as white solid and Na-salt.

$^{31}P\{^1H\}$-NMR (162 MHz, D_2O, δ/ppm): -5.26 – -6.70 (m, 2P), -20.50 – -21.71 (m, 2P). **HRMS** (ESI) m/z for $H_5O_9{}^{18}O_4P_4$ $[M-H]^-$: calcd.344.8856, found 344.8863.

6.3. Synthesis of $^{18}O_n$ – Terpenoidphosphates

Isoprenylphosphate (SI-4)

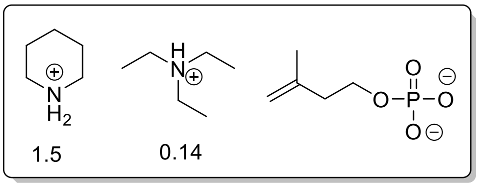

Isoprenol (130 mg, 151 µL, 1.51 mmol), and ETT (491 mg, 3.78 mmol, 2.5 eq.) were dissolved in DMF (3.0 ml). $(FmO)_2P\text{-}N\textit{i}Pr_2$ (1.02 g, 1.97 mmol, 1.3 eq.) was added as solution in DMF (2.0 ml) and the resulting mixture was stirred for 1 h at rt. The solution was cooled to 0 °C and *m*CPBA (77%, 724 mg, 2.95 mmol, 1.5 eq.) was added. The solution was stirred for 15 min at 0 °C and piperidine (10 vol%) was added. The solution was stirred for 40 min at rt. The solvent was removed under reduced pressure. The crude product was purified by RP-MPLC [C18-AQ, dryload on celite, elution with H_2O/MeCN/ TEAA (10 mM)]. The product containing fractions were identified by NMR or HPLC and the product was isolated after lyophilization. The product (175 mg, 573 µmol, 38%) was isolated as white solid.

^{1}H-NMR (400 MHz, D_2O, δ/ppm): 4.89 – 4.86 (m, 1H), 4.84 – 4.82 (m, 1H), 3.99 (td, *J* = 6.7, 6.6 Hz, 2H), 3.19 – 3.13 (m, 7H), 3.12 – 3.04 (m, 1H), 2.38 (d, *J* = 2549.5 Hz, 1H), 1.85 – 1.71 (m, 10H), 1.70 – 1.63 (m, 3H), 1.27 (t, *J* = 7.3 Hz, 1H). **^{13}C{^{1}H}-NMR** (101 MHz, D_2O, δ/ppm): 143.62, 111.52, 63.71 (d, *J* = 5.2 Hz), 44.51, 37.92 (d, *J* = 7.2 Hz), 27.12, 22.18, 21.55, 21.45, 14.47. **^{31}P{^{1}H}-NMR** (162 MHz, D_2O, δ/ppm): 0.49. **HRMS** (ESI) m/z for $C_5H_{10}O_4P$ $[M\text{-}H]^-$: calcd. 165.0322, found 165.0323.

β-$^{18}O_2$-Isoprenyl-diphosphate (β-$^{18}O_2$-IPP, 43)

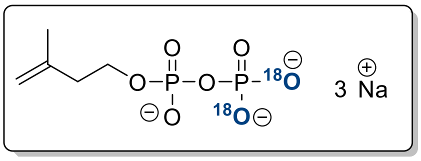

Isoprenylphosphat x 1.25 TBA (**SI-4**, 34.8 mg, 75.0 µmol) and ETT (29.1 mg, 224 µmol, 3.0 eq.) were dissolved in dry DMF (2.0 mL). Afterwards $^{18}O_2$-P-amidite **31** (97.0 mg, 112.0 µmol, 1.5 eq.) was added and the mixture was stirred for 15 min at rt. Subsequently, the reaction mixture was cooled to -20°C before *m*CPBA (77%, 31.2 mg, 140 µmol, 1.9 eq.) was added. The solution was stirred for 15 min at -20°C. The mixture was warmed to rt before THF

(2.0 mL) and TBAF (1 M in THF, 1.12 mL, 1.12 mmol, 15.0 eq.) were added. The solution was stirred overnight and the crude product was precipitated by the addition of Et_2O (40 mL). The precipitate was separated by centrifugation and washed with Et_2O (40 mL) and dried under high vacuum. Subsequently, the crude product was purified by automated SAX (Äkta pure, Q-Sepharose, $NaClO_4$ – buffer). The product containing fractions were lyophilized. The resulting solid was washed with acetone (2 x 20.0 mL), separated by centrifugation and dried under high vacuum. The product (**43**, 17.8 mg, 57.1 µmol, 76%) was isolated as white solid and Na-salt.

^{1}H-NMR (400 MHz, D_2O, δ/ppm): 4.92 – 4.82 (m, 2H), 4.06 (q, *J* = 6.7 Hz, 2H), 2.43 – 2.37 (m, 2H), 1.79 (t, *J* = 0.5 Hz, 3H). **^{13}C{^{1}H}-NMR** (101 MHz, D_2O, δ/ppm): 144.00, 111.41, 63.97 (d, *J* = 5.5 Hz), 37.88 (d, *J* = 7.7 Hz), 21.71. **^{31}P{^{1}H}-NMR** (162 MHz, D_2O, δ/ppm): -6.00 (d, *J* = 21.9 Hz), -10.16 (d, *J* = 21.8 Hz). **HRMS** (ESI) m/z for $C_5H_{11}O_5{}^{18}O_2P_2$ $[M-H]^-$: calcd. 249.0070, found 249.0076.

6.4. Synthesis of $^{18}O_n$ – Magic Spot Nucleotides

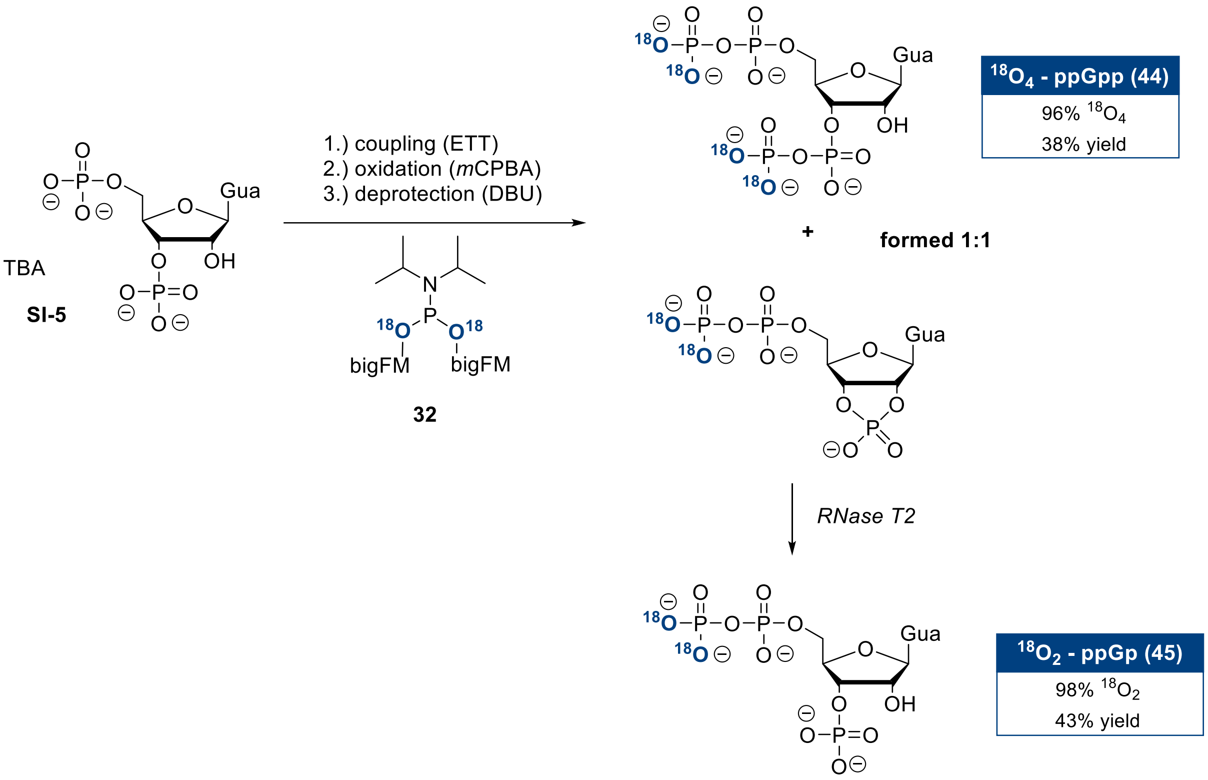

Supporting Figure 3: Synthetic route towards ^{18}O – Magic Spot Nucleotides.

Guanosine-3',5'-bisphosphate (pGp, SI-5)

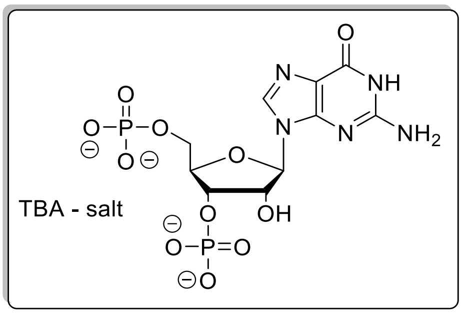

The compound was synthesized according to Haas et al.[4] Analytical data are in accordance with literature.

Guanosine-3'-5'-bis(β-$^{18}O_2$-diphosphate) ($^{18}O_4$ – ppGpp, 44)

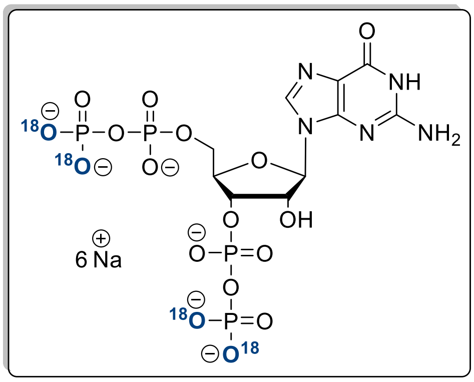

pGp x 1.60 TBA (**SI-5**, 39.0 mg, 47.0 µmol) and ETT (30.7 mg, 236 µmol, 5.0 eq.) were dissolved in dry DMF (2.5 mL). $^{18}O_2$-BigFM-P-Amidite **32** (127 mg, 118 µmol, 2.5 eq.) was added and the mixture was stirred for 15 min at rt. Afterwards the solution was cooled to 0°C and *m*CPBA (77%, 38.3 mg, 171 µmol, 3.6 eq.) was added. The solution was stirred for 15 min at 0°C before DBU (500 µL) was added. The mixture was stirred for 45 min at rt. Subsequently, the crude product was precipitated by the addition of Et_2O (40 mL). The precipitate was separated by centrifugation, washed with Et_2O (20 mL) and dried under high vacuum. The resulting crude product was purified by automated SAX (Äkta pure, Q-Sepharose, $NaClO_4$-buffer). The product containing fractions (ca. 100 mM) were lyophilized. The resulting solid was washed with acetone (2 x 20 mL), separated by centrifugation and dried under high vacuum. The product (**44**, 13.2 mg, 17.8 µmol, 38%) was isolated as white solid and Na-salt. The fractions containing the side-product guanosine-2',3'-cyclophosphate-5'-triphosphate are combined and used for the synthesis of ppGp (**45**, see subsequent procedure).

^{1}H-NMR (400 MHz, D_2O, δ/ppm): 8.16 (s, 1H), 6.00 (d, *J* = 6.3 Hz, 1H), 4.99 – 4.93 (m, 1H), 4.93 – 4.84 (m, 1H), 4.60 – 4.48 (m, 1H), 4.31 – 4.15 (m, 3H). **^{13}C{^{1}H}-NMR** (101 MHz, D_2O, δ/ppm): 159.70 (dd, *J* = 10.4, 8.0 Hz), 154.44, 151.93, 137.83, 116.37, 86.78, 83.39 (dd, *J* = 10.2, 8.8 Hz), 74.79 (d, *J* = 4.2 Hz), 73.10 (d, *J* = 3.5 Hz), 65.03 (d, *J* = 5.0 Hz) 54.93, 152.59, 148.17, 139.04, 117.86, 87.20, 83.03 (d, *J* = 9.4 Hz), 74.86, 69.59, 64.61 (d, *J* = 5.5 Hz). **^{31}P{^{1}H}-NMR** (162 MHz, D_2O, δ/ppm): -5.57 (d, *J* = 22.2 Hz), -5.84 (d, *J* = 21.4 Hz), -10.44 (d, *J* = 21.2 Hz), -10.70 (d, *J* = 22.1 Hz). **HRMS** (ESI) m/z for $C_{10}H_{16}O_{13}{}^{18}O_4N_5P_4$ $[M-H]^-$: calcd. 609.9667, found 609.9681.

Guanosine-3'-phosphate-5'-β-$^{18}O_2$-diphosphate ($^{18}O_2$-ppGp, 45)

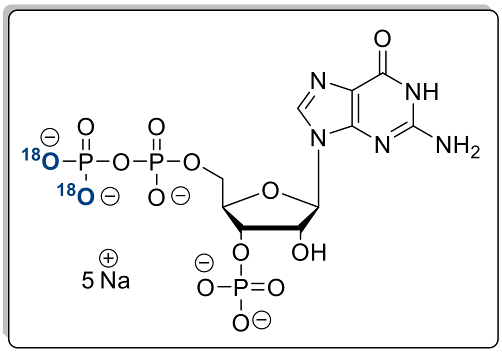

The fractions containing the side-product guanosine-2',3'-cyclophosphate-5'-triphosphate from the ppGpp-purification (see above) are combined and lyophilized. The resulting solid was washed with acetone (2 x 20 mL), separated by centrifugation and dried under high vacuum. Subsequently it was dissolved in H_2O (2 mL) and Rnase T2 (30 µl) was added. The pH was adjusted to 5.5 by the addition of aq. HCl and the resulting solution was incubated overnight at 37 °C. The resulting solution was directly applied to automated SAX-purification. (Äkta pure, Q-Sepharose, $NaClO_4$ – buffer). The product containing fractions (ca. 100 mM) were lyophilized. The resulting solid was washed with acetone (2 x 20 mL), separated by centrifugation and dried under high vacuum. The product (**45**, 12.8 mg, 20.1 µmol, 43%) was isolated as white solid and Na-salt.

^{1}H-NMR (400 MHz, D_2O, δ/ppm): 8.18 (s, 1H), 5.97 (d, *J* = 7.1 Hz, 1H), 4.83 (dd, *J* = 7.1, 5.0 Hz, 1H), 4.75 (ddd, *J* = 7.3, 5.0, 2.4 Hz, 1H), 4.52 (dtd, *J* = 1.9, 1.9, 1.9 Hz, 1H), 4.21 (dd, *J* = 5.2, 3.7 Hz, 2H). **^{13}C{^{1}H}-NMR** (101 MHz, D_2O, δ/ppm): 159.33, 154.19, 152.01, 137.81, 116.26, 86.50, 84.05 (dd, *J* = 9.1, 5.4 Hz), 73.64 (d, *J* = 3.1 Hz), 73.53 (d, *J* = 4.5 Hz), 65.28 (d, *J* = 5.3 Hz). **^{31}P{^{1}H}-NMR** (162 MHz, D_2O, δ/ppm): 4.03, -5.99 (d, *J* = 21.3 Hz), -10.45 (d, *J* = 21.8 Hz). **HRMS** (ESI) m/z for $C_{10}H_{16}O_{12}{}^{18}O_2\ N_5P_3$ $[M\text{-}H]^+$: calcd. 525.9919, found 525.9938.

6.5. Synthesis of $^{18}O_n$ – Inositolpoly and -pyrophosphates

6.5.1. Syntheses applying (hydroxymethyl)phenol-based ^{18}O-P-amidites

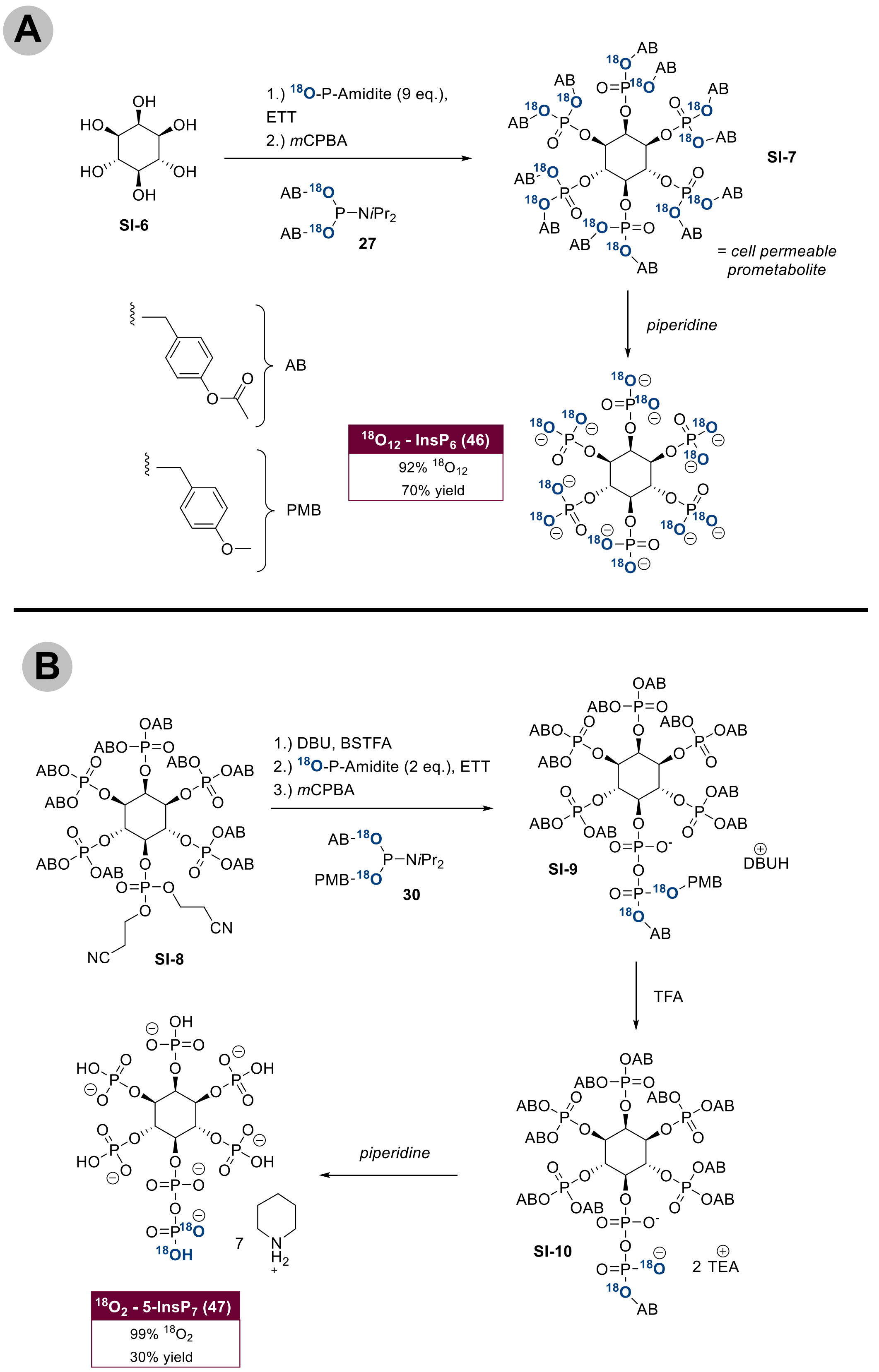

Supporting Figure 4: Synthetic access towards $^{18}O_{12}$ – $InsP_6$ (Figure A) and $^{18}O_2$-5-$InsP_7$ using ^{18}O-P-amidites.

AB-protected $^{18}O_{12}$ – myp-Inositolhexakisphosphate (AB_{12}-$^{18}O_{12}$-$InsP_6$, SI-7)

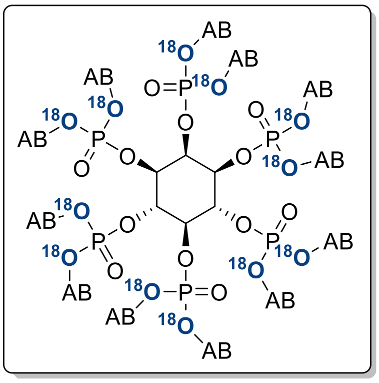

myo-Inositol (**SI-6**, 4.00 mg, 21.5 µmol, 1.0 eq) and $^{18}O_2$-P-amidite **27** (93.0 mg, 200 µmol, 9.0 eq) were dissolved in DMF (500 µL). ETT (25.8 mg, 200 µmol, 9.0 eq) was added and the resulting mixture was stirred at rt. The reaction progress was followed by ^{31}P-NMR. After completion of the reaction (2 h), the mixture was cooled to 0°C and *m*CPBA (70%, 49.3 mg, 200 µmol, 9.0 eq) was added. The mixture was stirred for 15 min at rt. The mixture was diluted with EtOAc (10 mL) and washed with H_2O (3 × 10 mL) and brine (10 mL). The solution was dried under $MgSO_4$ and the solvent was removed under reduced pressure. The crude product was purified by automated MPLC (Interchim-system, C18aq-column, H_2O/MeCN). The product (**SI-7**, 37 mg, 15.0 µmol, 70%) was isolated as colorless oil.

^{1}H-NMR (400 MHz, $CDCl_3$, δ/ppm): 7.33 – 7.25 (m, 8H), 7.22 – 7.13 (m, 16H), 6.99 – 6.87 (m, 24H), 5.59 (d, *J* = 9.0 Hz, 1H), 5.23 – 4.86 (m, 26H), 4.37 (t, *J* = 9.6 Hz, 2H), 4.12 (q, *J* = 7.2 Hz, 1H), 2.29 – 2.20 (m, 36H). **^{31}P{^{1}H}-NMR** (162 MHz, $CDCl_3$, δ/ppm): -0.77 (s, 1P), -0.87 (s, 2P), -1.81 (s, 2P), -2.75 (s, 1P). **HRMS** (ESI) m/z for $C_{114}H_{116}O_{36}{}^{18}O_{12}P_6$ $[M+H_2]^{2+}$: calcd. 1231.2780 found 1231.2774.

$^{18}O_{12}$ – myo-Inositolhexakisphosphate ($^{18}O_{12}$-IP6, 46)

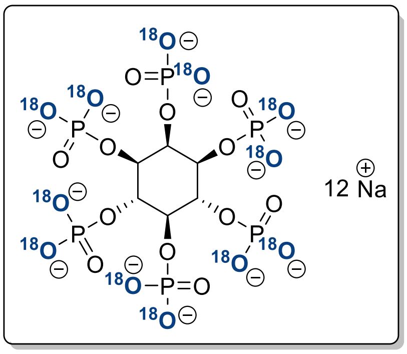

AB-protected hexakisphosphate **SI-7** (28.0 mg, 11.5 µmol) was dissolved in DMF (2.0 mL) and piperidine (1.0 mL) was added. The resulting mixture was stirred at rt, and the reaction progress was followed by ^{31}P-NMR. After completion of the reaction (3 h), the product was precipitated with Et_2O (100 mL). The precipitate was centrifuged and the collected solid was redissolved in MeOH (0.5 mL). Reprecipitation with $NaClO_4$ – solution (0.5 M in acetone, 40 mL) was induced. The precipitate was separated by centrifugation and washed with acetone. The product was isolated as white solid (**46**, 10.0 mg, 10.6 µmol, 93%).

^{1}H-NMR (400 MHz, D_2O, δ/ppm): δ 4.94 – 4.85 (m, 1H), 4.52 – 4.38 (m, 2H), 4.21 – 3.99 (m, 3H). **^{31}P{^{1}H}-NMR** (162 MHz, D_2O, δ/ppm): 1.95 (s, 1P), 1.03 (s, 2P), 0.72 (s, 3P). **HRMS** (ESI) m/z for $C_6H_{17}O_{12}{}^{18}O_{12}P_6$ [M-H]$^-$: calcd. 682.9050, found 682.9058.

(AB$_{10}$ - *β*-CE$_2$)-myo-Inositolhexakisphosphate SI-8

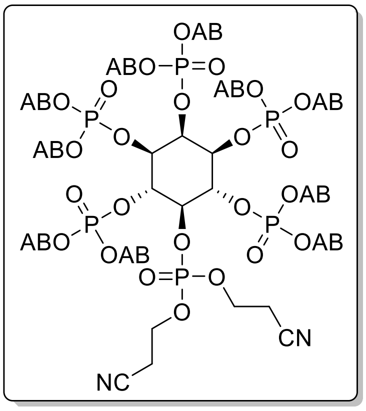

The compound was synthesized according to Pavlovic et al.[5] Analytical data are in accordance with literature.

^{18}O- 5-Diphospho-inositol pentakisphosphate derivative SI-9

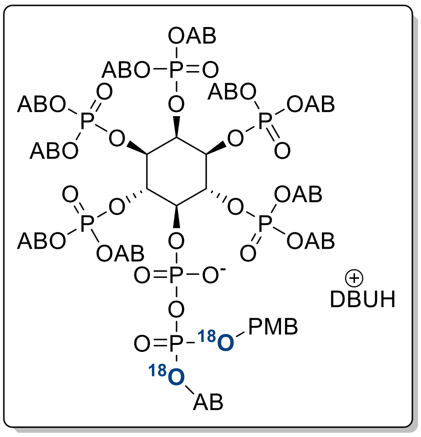

Hexakisphosphate **SI-8** (50.0 mg, 22.3 µmol, 1.0 eq.) was co-evaporated with MeCN (1.0 mL) and then dissolved in MeCN (2.0 mL). DBU (13.3 µL, 13.5 mg, 89.0 µmol, 4.0 eq.) and 2 min afterwards, BSTFA (23.8 µL, 22.9 mg, 89.0 µmol, 4.0 eq.) were added. Turnover was monitored by TLC (EtOAc:MeOH, 9:1). After 5 minutes TFA (6.8 µL, 10 mg, 89.0 µmol, 4.0 eq.) in MeOH (20 µL) was added and the solvent was immediately removed under reduced pressure. The residual was dissolved in MeCN (2 mL) and $^{18}O_2$-AB-PMB-P-amidite **30** (19.5 mg, 44.5 µmol. 2.0 eq.) was added. An ETT-solution in THF (1M, 44.5 µL, 44.5 µmol, 2.0 eq.) was added and the reaction mixture was stirred for 30 minutes. Subsequently, *m*CPBA (70%, 10.2 mg, 44.5 µmol, 2.0 eq.) was added at 0°C. The mixture was stirred for 10 min at rt. The product was precipitated with Et2O (40 mL), redissolved in DCM (2 mL) and reprecipitated twice with Et_2O (45 mL). The precipitate was dried under high vacuum. The product (**SI-9**, 30.0 mg, 14.7 µmol, 66 %) was isolated as DBU-salt and colorless solid.

^{1}H-NMR (400 MHz $CDCl_3$, δ/ppm): 11.42 (s, $DBUH^+$), 7.48 – 7.08 (m, 24H), 7.03 – 6.85 (m, 22H), 6.73 (d, J = 8.6 Hz, 2H), 5.41 – 4.89 (m, 30H), 3.73 (s, 2H), 3.34 – 3.28 (m, $DBUH^+$), 3.24 (t, J = 5.9 Hz, $DBUH^+$), 2.65 (s, $DBUH^+$), 2.32 – 2.24 (m, 33H), 1.91 – 1.73 (m, $DBUH^+$), 1.58 (d, J = 12.2 Hz, $DBUH^+$).**^{13}C{^{1}H}-NMR** (101 MHz, $CDCl_3$, δ/ppm): 169.14, 166.10, 151.57 – 149.76 (m), 129.75, 129.63 (d, J = 1.8 Hz), 129.30 (d, J = 1.8 Hz), 129.25, 129.16, 128.87, 121.62, 121.58, 121.53, 121.49, 121.42, 113.68, 69.43 – 68.89 (m), 55.19, 54.15, 48.47, 47.32, 38.04, 32.19, 28.90, 26.74, 23.88, 21.12, 19.31. **^{31}P{^{1}H}-NMR** (162 MHz, $CDCl_3$, δ/ppm): -0.83 (s, 2P), -1.88 (s, 2P), -3.13 (s, 1P), -10.94 (d, J = 14.5 Hz, 1P), -12.48 (d, J = 14.7 Hz, 1P). **HRMS** (ESI) m/z for $C_{112}{}^{13}CH_{114}O_{48}{}^{18}O_2P_7$ $[M-H]^-$: calcd. 2492.4551, found 2492.4551.

^{18}O- 5-Diphospho-inositol pentakisphosphate derivative SI-10

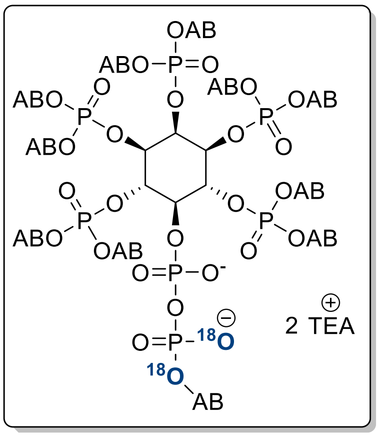

Protected diphosphoinositolpentakisphosphate SI-9 (21 mg, 7.9 µmol) was dissolved in DCM (2.0 mL), TFA (6.1 µL, 9.0 mg, 79 µmol, 10 eq.) was added and the reaction mixture was stirred for 15 min. Most volatiles were removed under high vacuum. The crude product was dissolved in MeCN and directly purified automated MPLC (Interchim-system C18aq-column, MeCN/H_2O/TEAA-buffer (5 mM) to avoid decomposition. The product (**SI-10**, 18 mg, 7.0 µmol, 88%) was isolated as TEAA-salt and colorless solid.

^{1}H NMR (400 MHz, $CDCl_3$, δ/ppm): 7.40 – 7.12 (m, 22H), 6.98 – 6.82 (m, 22H), 5.40 – 4.73 (m, 26H), 2.83 (q, *J* = 7.3 Hz, TEA^+), 2.29 - 2.20 (m, 33H), 1.05 (t, *J* = 7.3 Hz, TEA^+). **^{13}C{^{1}H}-NMR** (101 MHz, $CDCl_3$, δ/ppm): 174.66, 169.57, 169.41, 169.34, 169.32, 169.29, 150.71, 150.65, 150.54, 149.85, 137.00, 136.91, 133.52, 133.45, 129.50, 129.40, 129.36, 128.73, 121.74, 121.68, 121.61, 121.27, 77.36, 75.73, 74.05, 69.48, 69.43, 69.36, 69.29, 69.23, 69.10, 66.83, 66.78, 45.23, 21.24, 8.56. **^{31}P{^{1}H}-NMR** (162 MHz, $CDCl_3$, δ/ppm): -0.12 – -3.68 (m), -10.27 (d, *J* = 15.2 Hz), -11.95 (d, *J* = 15.1 Hz). **HRMS:** (ESI) m/z for $[M]^{2-}$ calcd. $C_{104}{}^{13}CH_{114}O_{47}{}^{18}O_2P_7$: 1185.7009, found 1185.7004

β-$^{18}O_2$- 5-Diphospho-inositol pentakisphosphate (β-$^{18}O_2$-5-IP7, 47)

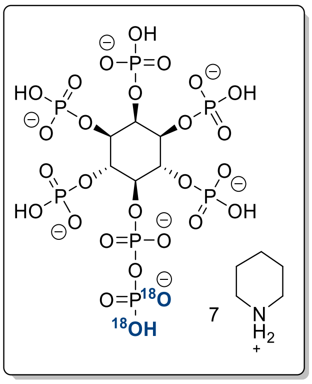

Protected diphosphoinositolpentakisphosphate SI-10 (9.0 mg, 3.5 µmol, 1.0 eq.) was dissolved in DMF (1.0 mL), piperidine (500 µL) was added and the reaction mixture was stirred for 2.5 h. The product was precipitated with Et_2O (14 mL), separated by centrifugation and washed with Et_2O (14 mL). The solid was dried under high vacuum for 1 h. The product (**47**, 1.83 mg, 1.47 mmol, 51%) was isolated as piperidinium salt and colorless solid.
The purity of the 5-IP7 was determined by CE-MS to >98%.

^{1}H NMR (400 MHz, D_2O, δ/ppm, one inositol-H is hidden under HDO-signal): 4.47 (q, *J* = 9.5 Hz, 2H), 4.28 (q, *J* = 9.5 Hz, 1H), 4.23 – 4.11 (m, 2H), 3.24 – 3.00 (m, PIP^+), 1.90 – 1.72 (m, PIP^+), 1.66 (q, *J* = 5.7 Hz, PIP^+). **^{31}P NMR** (162 MHz, D_2O, δ/ppm): 0.50 (s, 2P), 0.40 (s,

2P), -0.65 (s, 1P), -10.78 (d, *J* = 20.4 Hz, 1P), -11.09 (d, *J* = 19.8 Hz, 1P). **HRMS:** (ESI) m/z for $[M]^{2-}$ calcd. $C_6H_{17}O_{25}{}^{18}O_2P_7$: 370.9108, found 370.9112

6.5.1. Synthesis of $^{18}O_2$ – 1-InsP$_7$ using (Bn^{18}O)$_2$-PNiPr$_2$

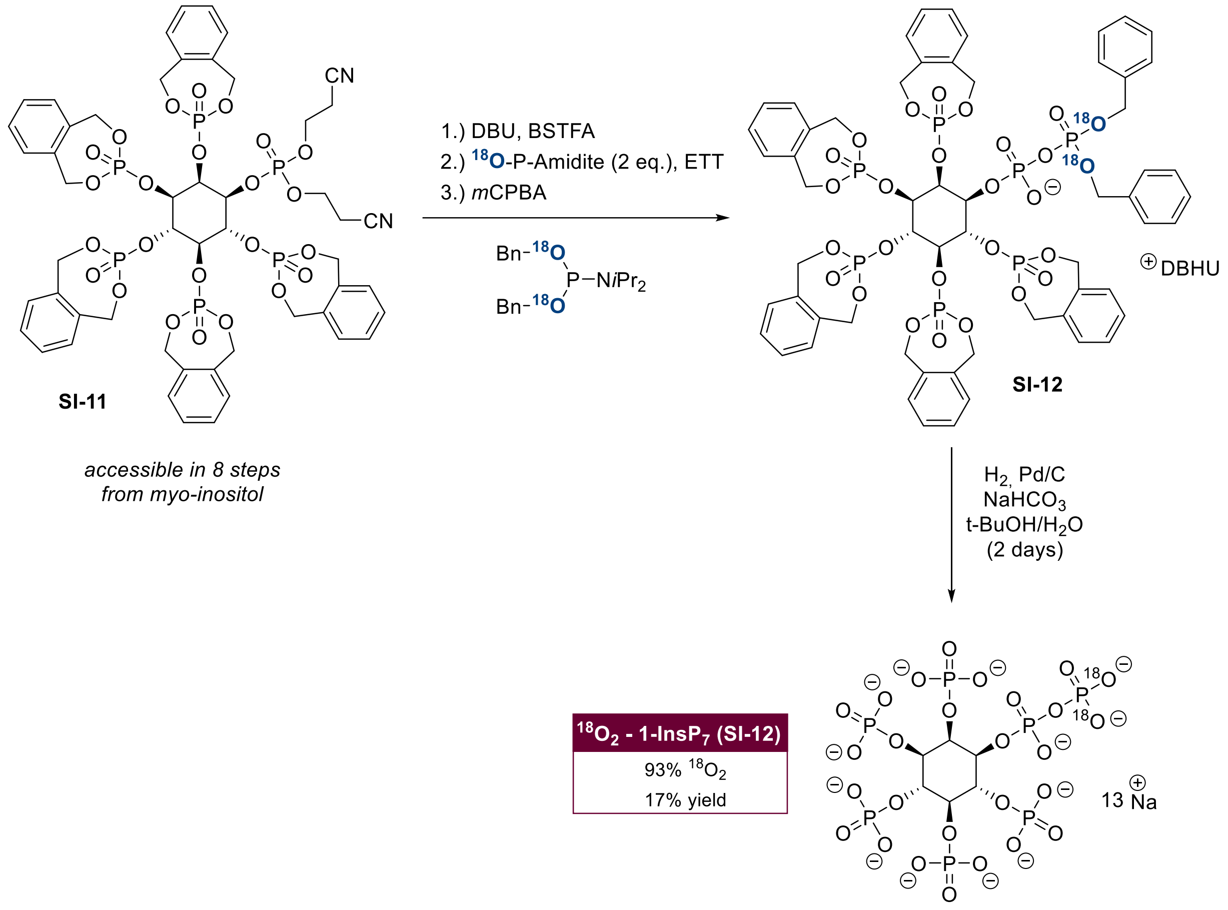

Supporting Figure 5: Synthesis of $^{18}O_2$-1-InsP$_7$ using known ^{18}O-Bn-P-amidites.

$^{18}O_2$-Dibenzyl diisopropylphosphoramidite (SI-14)

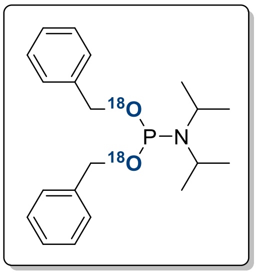

The compound was synthesized according to Hofer et al.[6] Analytical data are in accordance with literature.

1-β −CE/*o*-xylene-protected InsP6 (SI-11)

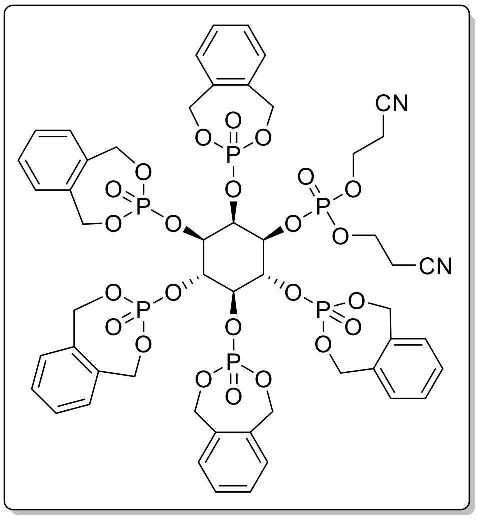

1-βCE/*o*-xylene-protected $InsP_6$ **SI-11** was synthesized in 8 steps from myo-inositol according to Capolicchio et al.[7] Analytical data are in accordance with literature.

Protected β-$^{18}O_2$- 1-Diphospho-inositol pentakisphosphate SI-12

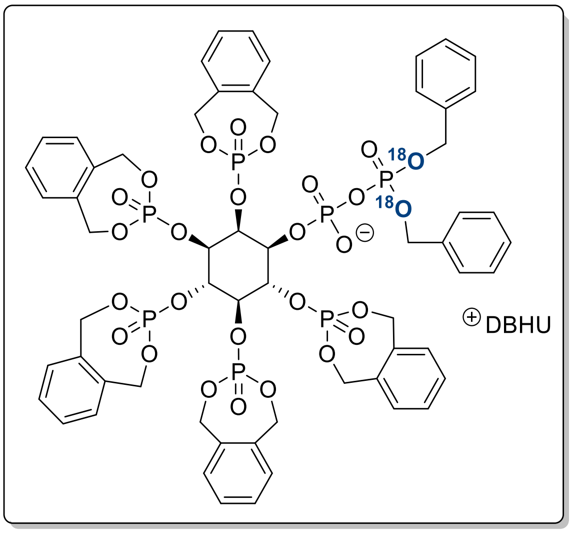

1-βCE/*o*-xylene-protected $InsP_6$ (**SI-11**, 80 mg, 56 µmol, 1.0 eq.) was dissolved in MeCN (2.0 mL). DBU (34 µl, 35 mg, 227 µmol, 4.0 eq.) and BSTFA (61 µl, 58 mg, 227 µmol, 4.0 eq.) were added and the solution stirred for 10 min at room temp. After completion of the deprotection, a solution of TFA (18 µl, 26 mg, 227 µmol, 4.0 eq.) in MeOH (60 µl) was added and stirred for 10 min. The mixture was evaporated to dryness, ETT (15 mg, 113 µmol, 2.0 eq.) added and coevaporated using MeCN (2.0 mL). The residue was taken up in MeCN (2.0 mL). $^{18}O_2$-P-amidite **SI-14** (40 mg, 113 µmol, 2.0 eq.) was dissolved in MeCN (2 mL) and then added to the reaction mixture. It was stirred for 15 min. At 0°C, *m*CPBA (≤ 77%, 25 mg, 113 µmol, 2.0 eq.) was added and stirred for 10 min. The product was precipitated using ice-

cold Et_2O (40 mL) and washed with ice-cold Et_2O (40 mL). Drying *in vacuo* gave the product (**SI-12**, 1.2 eq. DBU, 75 mg, 46 µmol, 83%) as white solid.

^{1}H-NMR (400 MHz, MeOD-d_4, δ/ppm): 1.20 (m, DBU), 1.25-1.42 (m, DBU), 1.70-1.84 (m, DBU), 2.03 (m, DBU), 2.68 (m, DBU), 3.36 (m, DBU), 3.51 (m, DBU), 3.55 (t, DBU), 3.61 (m, DBU), 4.92-6.03 (m, 30 H), 7.10-7.53 (m, 30 H). **^{31}P{^{1}H}-NMR** (162 MHz, MeOD-d_4, δ/ppm): -3.27, -3.39, -3.49, -4.09, -4.20, -11.45 (d, J = 15.9 Hz), -13.45 (d, J = 14.6 Hz). **HRMS** (ESI) m/z for $C_{60}H_{60}O_{25}{}^{18}O_2P_7$ $[M-H]^+$: calcd: 1433.1576, found: 1433.1561.

β-$^{18}O_2$- 1-Diphospho-inositol pentakisphosphate (β-$^{18}O_2$-1-InsP$_7$, SI-13)

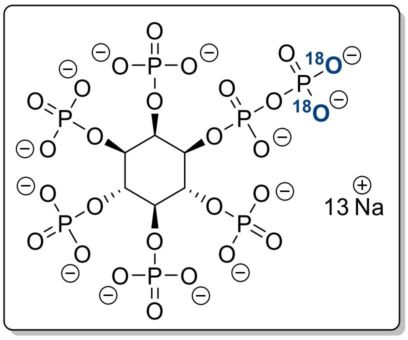

o-Xylene/benzyl-protected 1-P(^{18}O)P-InsP$_5$ (**SI-12**, 68 mg, 42.1 µmol) and $NaHCO_3$ (46 mg, 547 µmol, 13.0 eq.) were dissolved in *t*-BuOH and water (4:1, 4.5 mL) and Pd/C (672 mg, 632 µmol, 15.0 eq.) added. The reaction mixture was stirred 1.5 days under H_2 (35 bar) in a stainless-steel autoclave. Water (4.5 mL) was added, and the hydrogenation continued for 3.5 hours. The catalyst was removed by centrifugation, washed with water and the combined aqueous layers were freeze-dried. The residue was dissolved in water (5.0 mL) and precipitated using ice-cooled acetone (45 mL). The precipitated oil was washed with ice-cooled acetone (45 mL) and dried *in vacuo* to give a white solid. The crude product was purified by automated SAX (Q Sepharose, $NaClO_4$ - buffer, 200 – 300 mM). Product containing fractions were combined and precipitated at –20°C using a $NaClO_4$ solution (0.5 M in acetone). The precipitate was washed with cold acetone. Drying *in vacuo* gave the product (**SI-13**, 11.5 mg, 11.2 µmol, 27%) as white solid.

CE-MS analysis of the product revealed substantial amounts (>20%) of InsP$_6$ present in the sample.

$^{31}P\{^{1}H\}$-NMR (162 MHz, D_2O, δ/ppm): 3.33-4.85 (m, 5 P), –4.92 to –3.31 (br. s, 1 P) and -10.41 to –7.98 (br. s, 1 P). **HRMS** (ESI) m/z for $C_6H_{17}O_{25}{}^{18}O_2P_7$ [M-H_2]$^-$: calcd: 370.9108, found: 370.9108.

6.6. Synthesis of $^{18}O_n$ – DNA

Automated synthesis

The oligonucleotides were synthesized on a H-8 custom LNA, DNA/RNA automatic synthesizer from K&A Laborgeräte at 1 µmol scale employing the standard solid phase β-cyanoethyl-phosphoramidite chemistry in trityl-on mode. The DNA phosphoramidites (DMT-dA(bz), ^{16}O-P-amidite **SI-3**, ^{18}O-P-amidite **33**) were diluted to 100 mM with dry acetonitrile and synthesis occurred from the 3' towards the 5' end of the oligonucleotides on packed solid phase columns.

Deprotection and purification

The 34 µmol/g controlled pore glass (CPG) solid support was treated with conc. aq. ammonia/methylamine (1:1, 1.0 mL) for 2 h at 55°C. Afterwards milliQ H_2O (10 mL) was added and the CPG was removed by centrifugation and washed with milliQ H_2O (2 x 2.0 mL). The combined supernatants are syringe-filtered and lyophilized. The crude oligonucleotides are purified by preparative RP-HPLC. Product containing fractions were lyophilized.

The DMT group was cleaved by making a 2 wt% DNA-solution pH 4.0 (200 mM) NaOAc/HOAc-buffer (pH 4.0, 200 mM), containing 200 mM NaCl. This solution was shaken for 2 h at 50 °C. The product was precipitated by 2-propanol (-20°C, 10 volumes). The precipitate was separated by centrifugation and washed with EtOH (-20°C, 70%, 2.0mL). The residue was dissolved in milliQ H_2O (1.5 mL) and lyophilized. The purity of the obtained oligonucleotides were confirmed with analytical HPLC and CE-MS.

Synthesis of 5’-ATATATAT

P-Amidites applied for SPS:

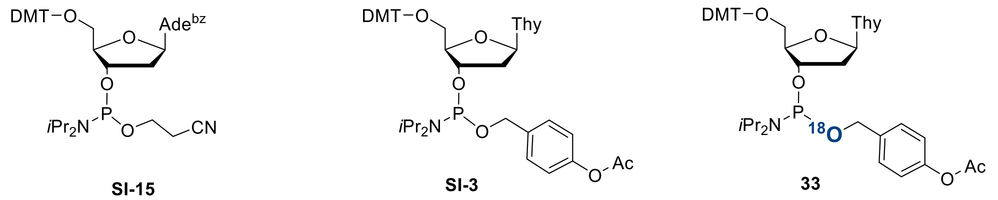

SI-15 **SI-3** **33**

DMT-5'-ATATATAT :

SI-16

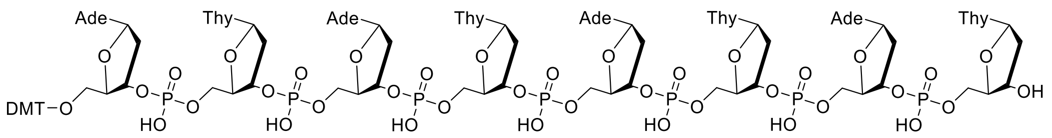

$^{18}O_3$ - DMT - 5' - ATATATAT :

SI-17

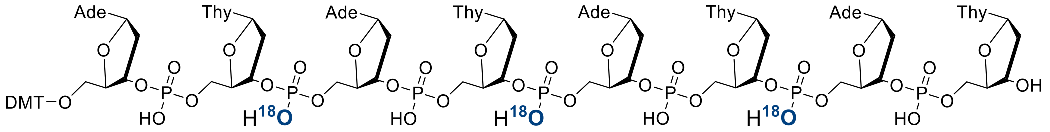

Supporting Figure 6: Synthesis of 5’-ATATATAT (**SI-18**) and its $^{18}O_3$ – isotopologue (**48**). The applied P-amidites are shown above.

The oligonucleotide sequence was synthesized according to the procedure described above. AB-protected P-amidites **33** and **SI-3** were applied for the incorporation of T. The synthesis was performed with ^{18}O labeled **48** as well as unlabeled **SI-18** in order to compare both oligonucleotide isotopologues. The yield was determined by UV-absorbance (NanoDrop®). 5’-ATATATAT (124 µg, 48.3 nmol, 4.8%) and $^{18}O_3$-5’ATATATAT (243 µg, 94.6 nmol, 9.5%) were both isolated as colorless oils after DMT-deprotection.

*DMT-5’-ATATATAT (**SI-15**):*
HRMS (ESI) m/z for $C_{101}H_{116}N_{28}O_{48}P_7$ $[M-H_3]^{3-}$: calcd. 902.1903, found: 902.1965.

*$^{18}O_3$-DMT-5’-ATATATAT (**SI-16**):*
HRMS (ESI) m/z for $C_{101}H_{117}N_{28}O_{45}{}^{18}O_3P_7$ $[M-H_2]^{2-}$: calcd.: 1356.7955, found: 1356.7931.

*5’-ATATATAT (**SI-18**):*
HRMS (ESI) m/z for $C_{80}H_{101}N_{28}O_{46}P_7$ $[M-H_2]^{2-}$: calcd.: 1202.2221, found: 1202.2227

*$^{18}O_3$-5’-ATATATAT (**48**):*

HRMS (ESI) m/z for $C_{80}H_{101}N_{28}O_{43}{}^{18}O_3P_7$ $[M-H_2]^{2-}$: calcd.: 1205.2285, found: 1205.2301.

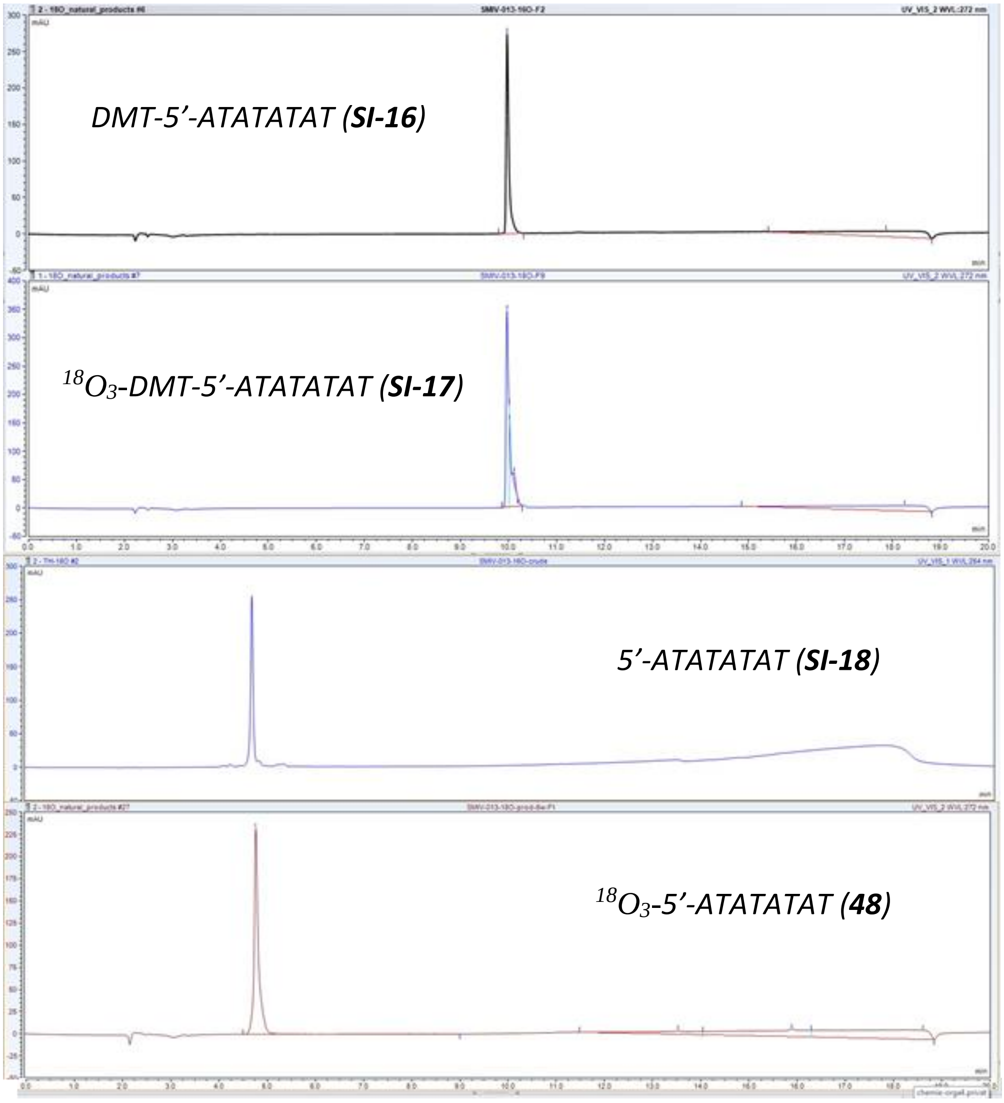

Supporting Figure 7: HPLC (C18-AQ column, H_2O/MeCN/TEAA-buffer [20 mM]) analyses of DMT-protected and deprotected 5’-ATATATAT isotopologues **SI-18** and **48**.

7. CE-MS measurements

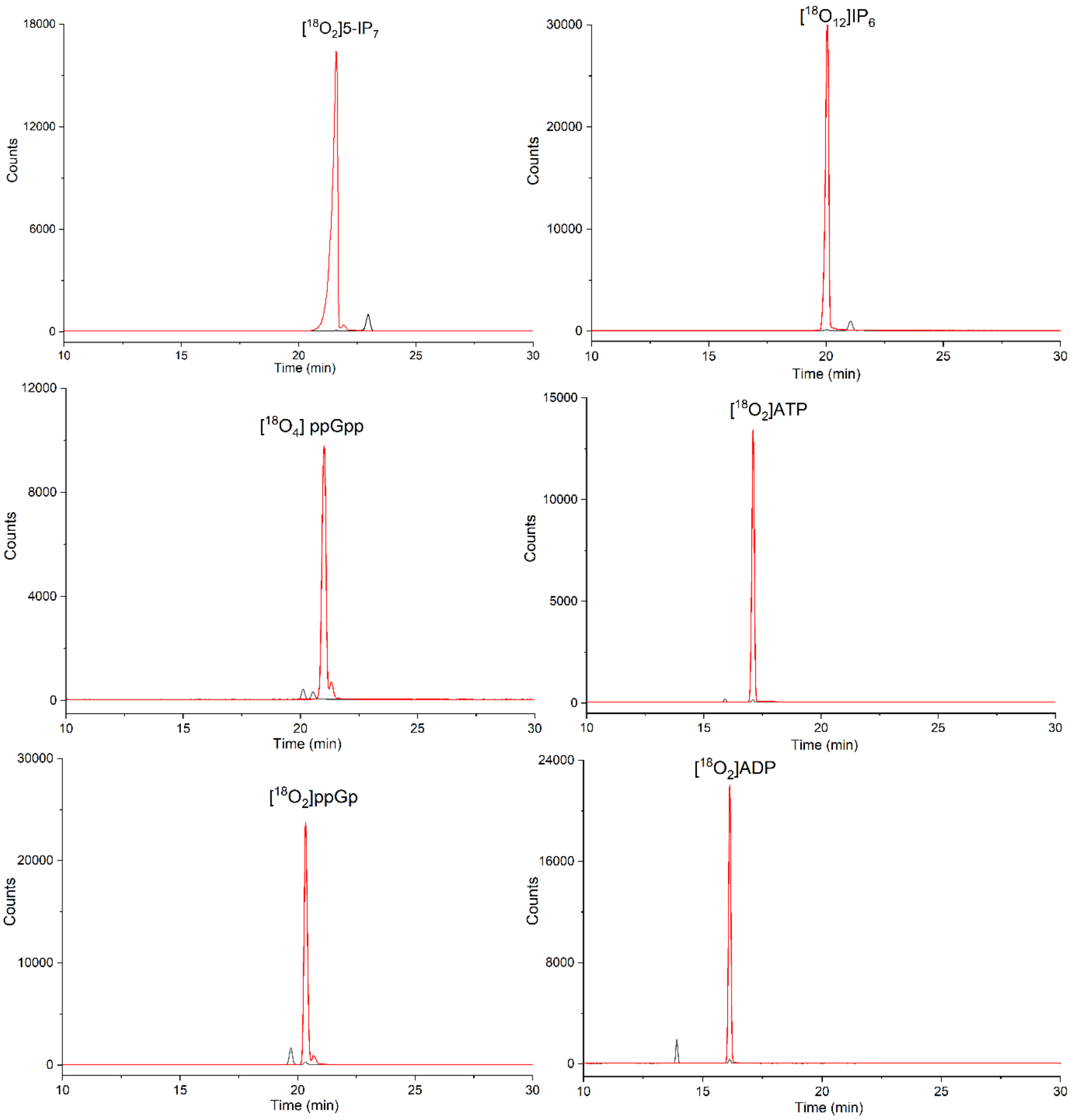

Supplementary Figure 11: Minimal neutral loss of phosphorylated analytes in CE-ESI-QqQ. Electropherogram in red is the corresponding analytes and the black electropherogram for tracking related in-source fragment. The degree of phosphate neutral loss in [$^{18}O_2$]5-IP_7, [$^{18}O_{12}$]IP_6, [$^{18}O_4$]ppGpp, [$^{18}O_2$]ATP, [$^{18}O_2$]ppGp and [$^{18}O_2$]ADP is 0.1%, 0.3%, 0.2%, 0.8%, 0.9% and 1.3%, respectively. BGE for [$^{18}O_{12}$]IP_6, [$^{18}O_2$]ATP, [$^{18}O_2$]ppGp and [$^{18}O_2$]ADP: 35 mM ammonium acetate titrated with ammonium hydroxide to pH 9.7, BGE for [$^{18}O_2$]5-IP_7 and [$^{18}O_4$]ppGpp: 40 mM ammonium acetate titrated with ammonium hydroxide to pH 9.0; Concentration of solutes: 20 µM for [$^{18}O_2$]5-IP_7 and 40 µM for the others. Other conditions is same as in CE-ESI-QqQ method description.

CE-ESI-QqQ was performed on an Agilent 7100 CE System coupled with a triple quadrupole tandem mass spectrometry Agilent 6495c system, equipped with an Agilent Jet Stream (AJS) electrospray ionization (ESI) source. CE-MS sheath liquid coaxial interface was adopted, with an isocratic LC pump constantly delivering the sheath-liquid (via a splitter set with a ratio of 1:100). Agilent MassHunter Workstation (Version 10.1) was employed to control the entire system, data acquisition and analysis. All experiments were performed on a bare fused silica capillary with a length of 100 cm (50 µm internal diameter and 365 µm outer diameter). 40 mM ammonium acetate titrated by ammonia solution to pH 9.0 was used as background eletrolyte (BGE). Samples were injected by applying 100 mbar pressure for 10 s, corresponding to 1% of the total capillary volume (20 nL).

The sheath liquid is a mixture of water-isopropanol (1/1, v/v), which was introduced at 10 µL/min. The MS source parameters settings: nebulizer pressure was 8 psi, gas temperature was 150 °C and with a flow of 11 L/min, sheath gas temperature was 175 °C and with a flow at 8 L/min, the capillary voltage was -2000 V with nozzle voltage 2000V. Negative high-pressure RF and low-pressure RF (Ion Funnel parameters) was 70V and 40V, respectively. Mass spectrometer parameters for MRM transitions are shown below, which were identified by MassHunter Optimizer software.

MSM parameter settings for IP_7 and IP_6 measurements:

Compound Name	Precursor Ion	Product Ion	dwell	Frag (V)	CE (V)	Cell Acc (V)	Polarity
$[^{18}O_2]IP_7$	370.9	319.9 [a]	80	166	10	3	Negative
$[^{18}O_2]IP_7$	370.9	83	80	166	57	4	Negative
IP_7	368.9	319.9 [a]	80	166	10	3	Negative
IP_7	368.9	79	80	166	57	4	Negative
$[^{18}O_{12}]IP_6$	340.9	495	80	166	17	4	Negative
$[^{18}O_{12}]IP_6$	340.9	83 [a]	80	166	60	4	Negative
IP_6	328.9	481	80	166	10	4	Negative
IP_6	328.9	79 [a]	80	166	60	4	Negative

[a] MRM transition with the highest response for each compound (used for quantitation)

MSM parameter settings for evaluating in-source fragmentation:

$[^{18}O_2]$5-IP_7

Compound Name	Precursor Ion	Product Ion	dwell	Frag (V)	CE (V)	Cell Acc (V)	Polarity
$[^{18}O_2]IP_7$	370.9	319.9[a]	100	166	10	3	Negative
$[^{18}O_2]IP_7$	370.9	83	100	166	57	4	Negative
$[^{18}O_2]IP_7$-$HPO[^{18}O]_2$	328.9	481	100	166	17	4	Negative
$[^{18}O_2]IP_7$-$HPO[^{18}O]_2$	328.9	79 [a]	100	166	60	4	Negative

$[^{18}O_{12}]IP_6$

Compound Name	Precursor Ion	Product Ion	dwell	Frag (V)	CE (V)	Cell Acc (V)	Polarity
$[^{18}O_{12}]IP_6$	340.9	495	100	166	17	4	Negative
$[^{18}O_{12}]IP_6$	340.9	83 [a]	100	166	57	4	Negative
$[^{18}O_{12}]IP_6$- $HPO[^{18}O]_2$	299	515 [a]	100	166	10	1	Negative
$[^{18}O_{12}]IP_6$- $HPO[^{18}O]_2$	299	83	100	166	14	1	Negative

[$^{18}O_4$]ppGpp

Compound Name	Precursor Ion	Product Ion	dwell	Frag (V)	CE (V)	Cell (V)	Acc	Polarity
[$^{18}O_4$]ppGpp	610	507.9 [a]	100	166	25	3		Negative
[$^{18}O_4$]ppGpp	610	162.8	100	166	45	4		Negative
[$^{18}O_4$]ppGpp- HPO[^{18}O]$_2$	526	428.1	100	166	21	3		Negative
[$^{18}O_4$]ppGpp- HPO[^{18}O]$_2$	526	424.1 [a]	100	166	21	3		Negative
[$^{18}O_4$]ppGpp- HPO[^{18}O]	526	158.8	100	166	41	5		Negative
[$^{18}O_4$]ppGpp- HPO[^{18}O]$_2$	526	162.8	100	166	41	5		Negative

[$^{18}O_2$]ATP

Compound Name	Precursor Ion	Product Ion	dwell	Frag (V)	CE (V)	Cell (V)	Acc	Polarity
[$^{18}O_2$]ATP	510	407.9	100	166	21	4		Negative
[$^{18}O_2$]ATP	510	162.9 [a]	100	166	37	4		Negative
[$^{18}O_2$]ATP- HPO[^{18}O]$_2$	426	328	100	166	17	4		Negative
[$^{18}O_2$]ATP- HPO[^{18}O]$_2$	426	158.9 [a]	100	166	21	4		Negative

[$^{18}O_2$]ppGp

Compound Name	Precursor Ion	Product Ion	dwell	Frag (V)	CE (V)	Cell (V)	Acc	Polarity
[$^{18}O_2$]ppGp	526	424 [a]	100	166	21	1		Negative
[$^{18}O_2$]ppGp	526	162.9	100	166	37	4		Negative
[$^{18}O_2$]ppGp- HPO[^{18}O]$_2$	442	344 [a]	100	166	17	4		Negative
[$^{18}O_2$]ppGp- HPO[^{18}O]$_2$	442	158.8	100	166	29	4		Negative

[$^{18}O_2$]ADP

Compound Name	Precursor Ion	Product Ion	dwell	Frag (V)	CE (V)	Cell (V)	Acc	Polarity
[$^{18}O_2$]ADP	430	328	100	166	17	4		Negative
[$^{18}O_2$]ADP	430	83[a]	100	166	60	1		Negative
[$^{18}O_2$]ADP- HPO[^{18}O]$_2$	346.1	97	100	166	25	4		Negative
[$^{18}O_2$]ADP- HPO[^{18}O]$_2$	346.1	78.9 [a]	100	166	46	4		Negative

The biological IP extracts are identical samples which were studied in our previous publication (*Nat. Commun.* **2020**, *11*, 6035).

8. Supporting references

[1] A. Ripp, J. Singh, H. J. Jessen, *Curr. Protoc. Nucleic Acid Chem.* **2020**, *81*, e108.

[2] A. Hofer, E. Marques, N. Kieliger, S.-K. N. Gatter, S. Jordi, E. Ferrari, M. Hofmann, T. B. Fitzpatrick, M. O. Hottiger, H. J. Jessen, *Org. Lett.* **2016**, *18*, 13, 3222-3225.

[3] A. Leisvuori, H. Lönnberg, M. Ora, *Eur. J. Org. Chem.* **2014**, 5816-5826.

[4] T. M. Haas, P. Ebensperger, V. B. eisenbeis, C. Nopper, T. Dürr, N. Jork, N. Steck, C. Jessen-Trefzer, H. J. Jessen, *Chem. Commun.* **2019**, *55*, 5399-5342.

[5] I. Pavlovic, D. T. Thakor, L. Bigler, M. S. C. Wilson, D. Laha, G. Schaaf, A. Saiardi and H. J. Jessen, *Angew. Chemie - Int. Ed.* **2015**, *54*, 9622–9626.

[6] A. Hofer, G. S. Cremosnik, A. C. Müller, R. Giambruno, C. Trefzer, G. Superti-Furga, K. L. Bennett, H. J. Jessen, *Chem. Eur. J.* **2015**, *21*, 10116-10122.

[7] S. Capolicchio, D. T. Thakor, A. Linden, H. J. Jessen, *Angew. Chem. Int. Ed.* **2013**, *52*, 6912-6916.

Compound 11: 4-(1,3-dioxolan-2-yl)phenol, ^{1}H - NMR ($CDCl_3$, 400 MHz)

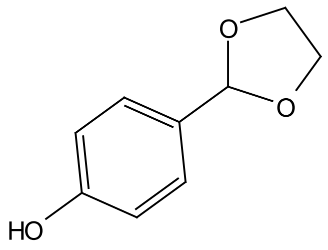

11

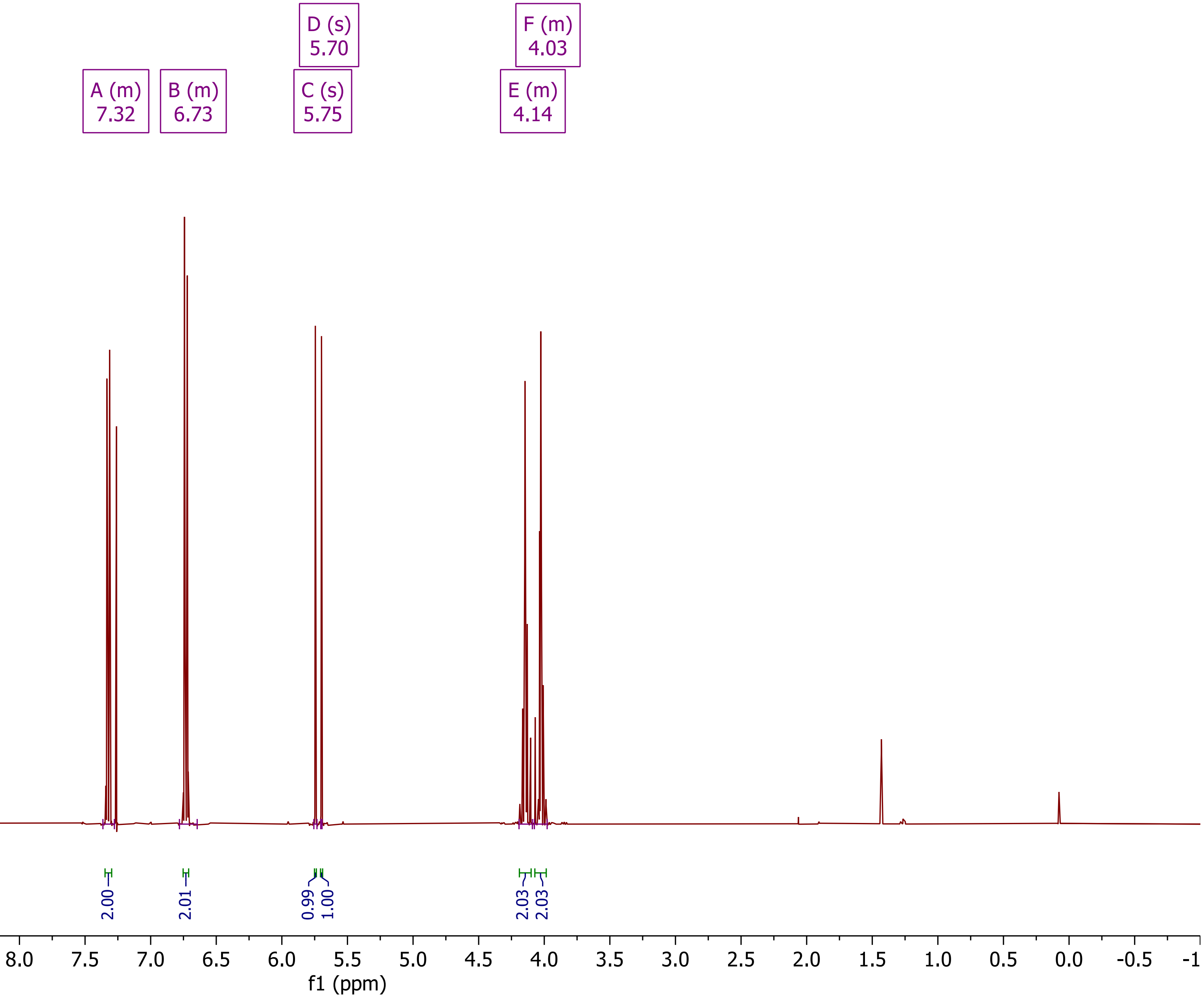

Compound 11: 4-(1,3-dioxolan-2-yl)phenol, $^{13}C\{^1H\}$ - NMR ($CDCl_3$, 101 MHz)

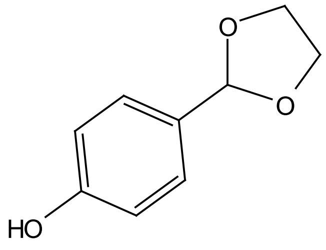

11

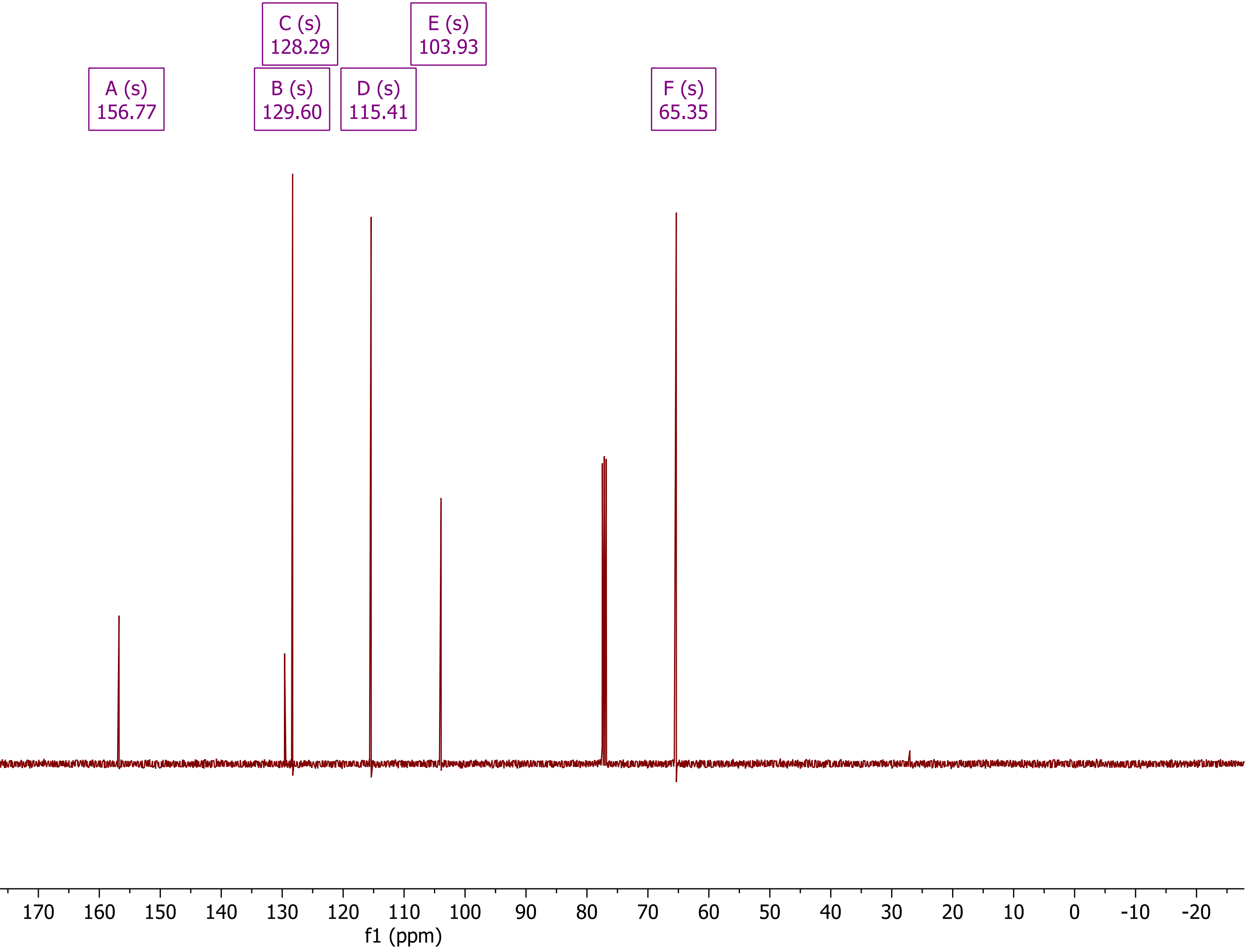

Compound 16: 4-((hydroxyl)methyl)phenol, ^{1}H - NMR (CD_3CN, 400 MHz)

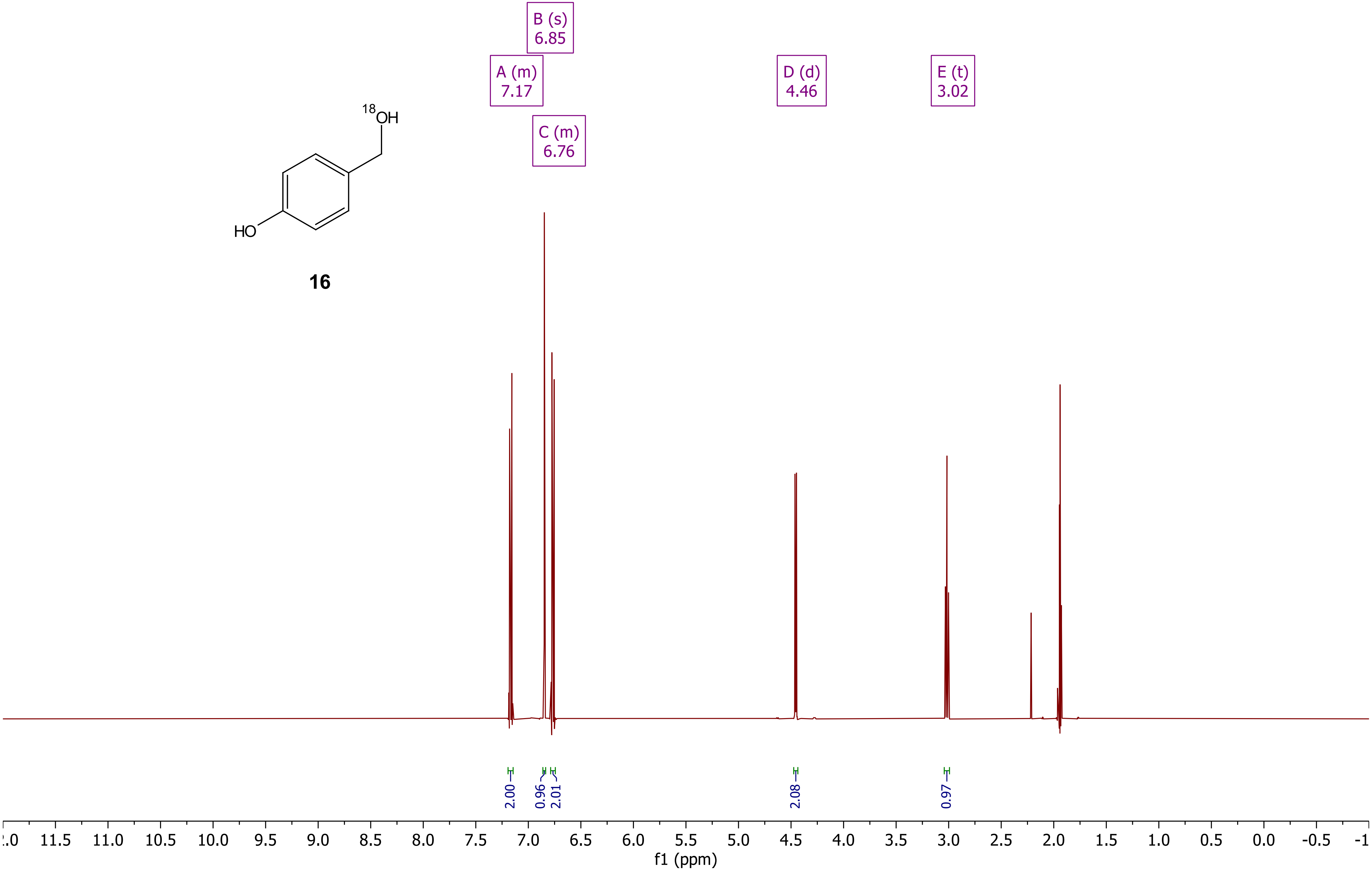

Compound 16: 4-((hydroxyl)methyl)phenol, $^{13}C\{^{1}H\}$ - NMR (CD_3CN, 101 MHz)

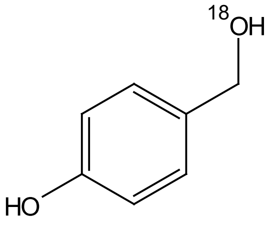

16

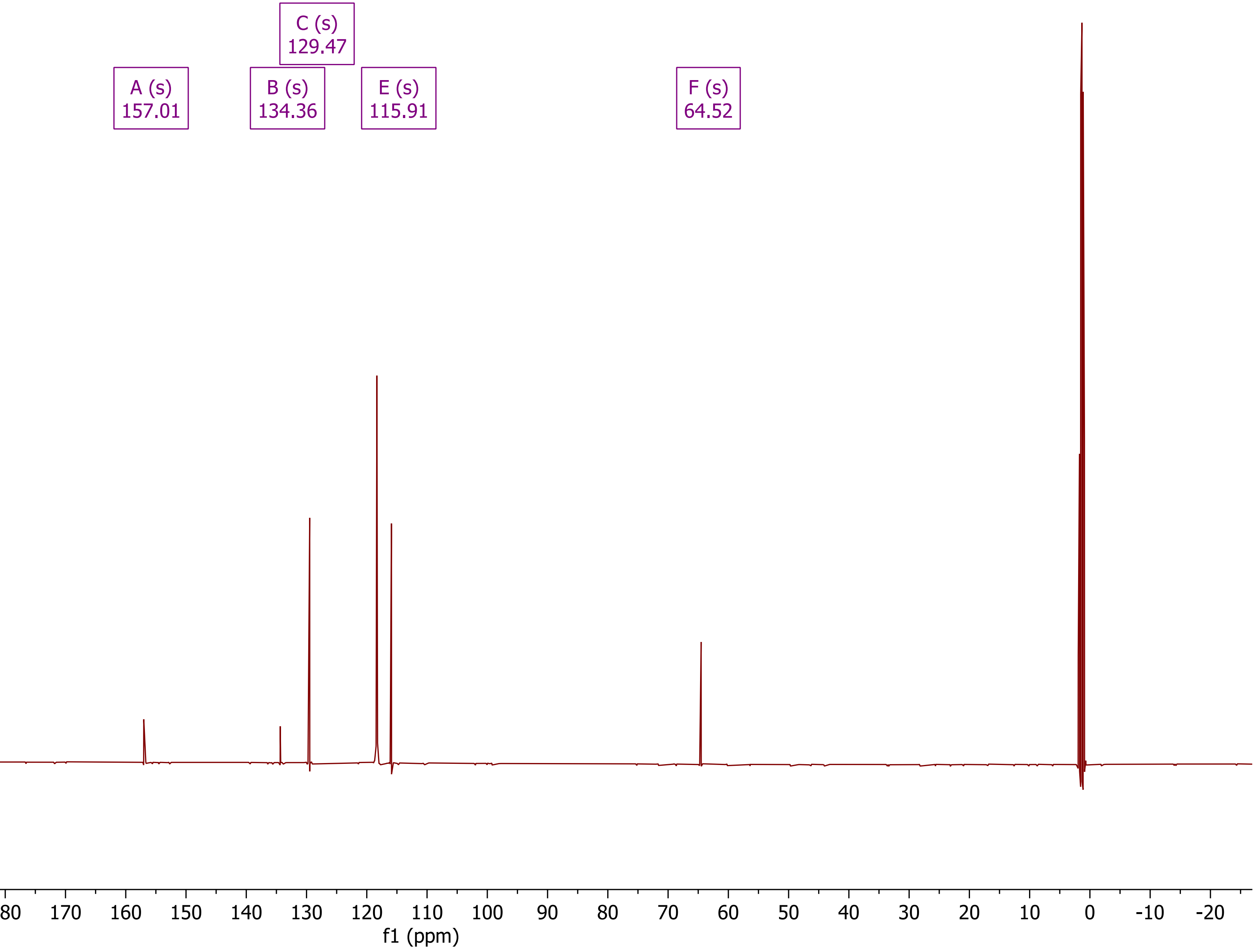

Compound 17:* ^{18}O-AB-alcohol, *^{1}H - NMR ($CDCl_3$, 400 MHz)

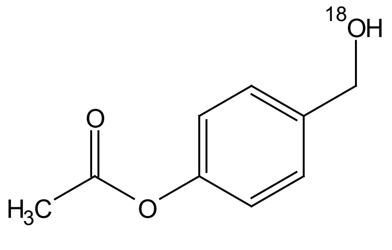

17

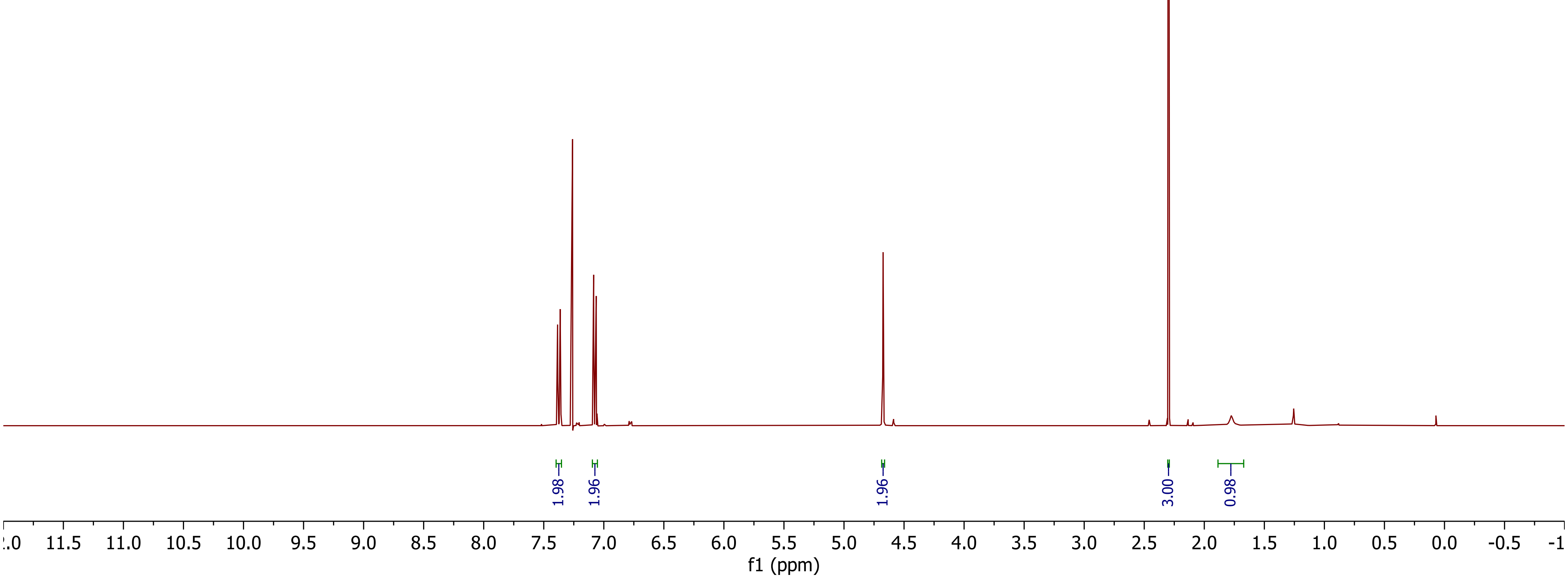

Compound 17:* ^{18}O-AB-alcohol, *^{13}C{^{1}H} - NMR (CDCl$_3$, 400 MHz)

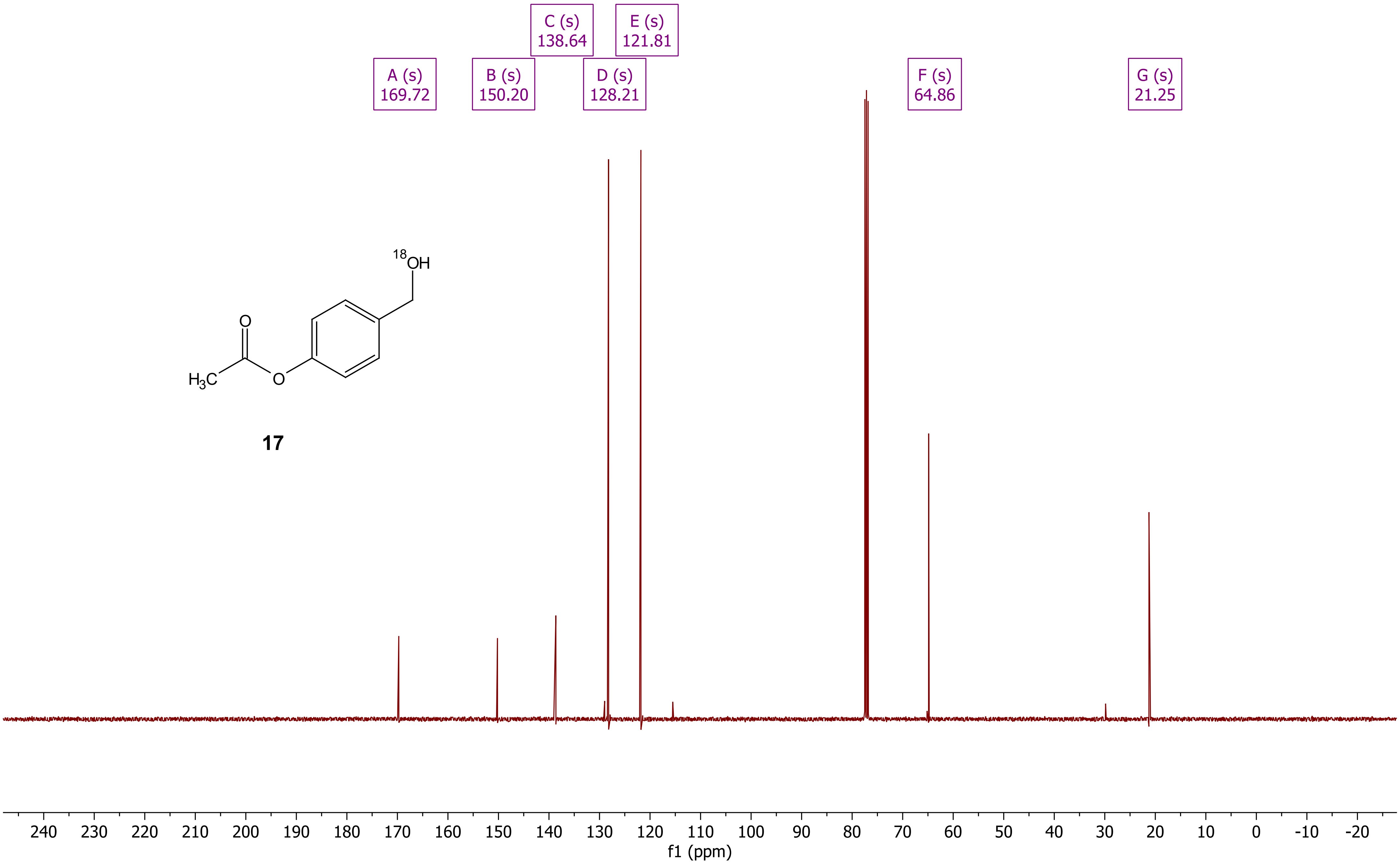

17

Compound 18:* ^{18}O-PMB-alcohol, *^{1}H - NMR ($CDCl_3$, 400 MHz)

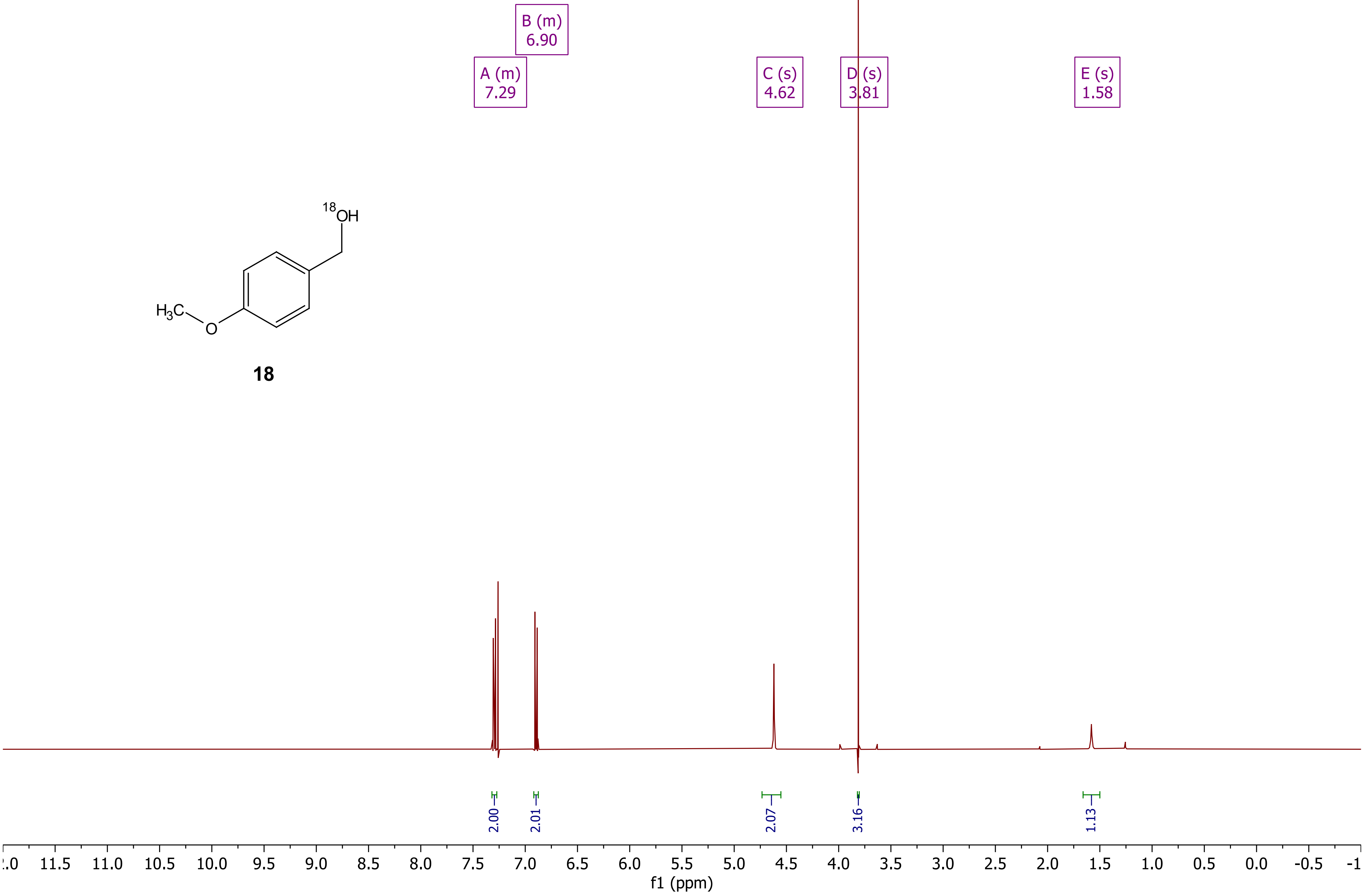

Compound 18: **^{18}O-PMB-alcohol,** ***$^{13}C\{^1H\}$ - NMR ($CDCl_3$, 101 MHz)***

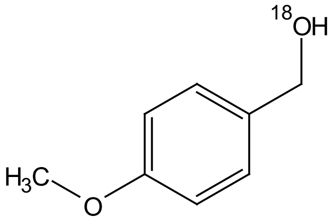

18

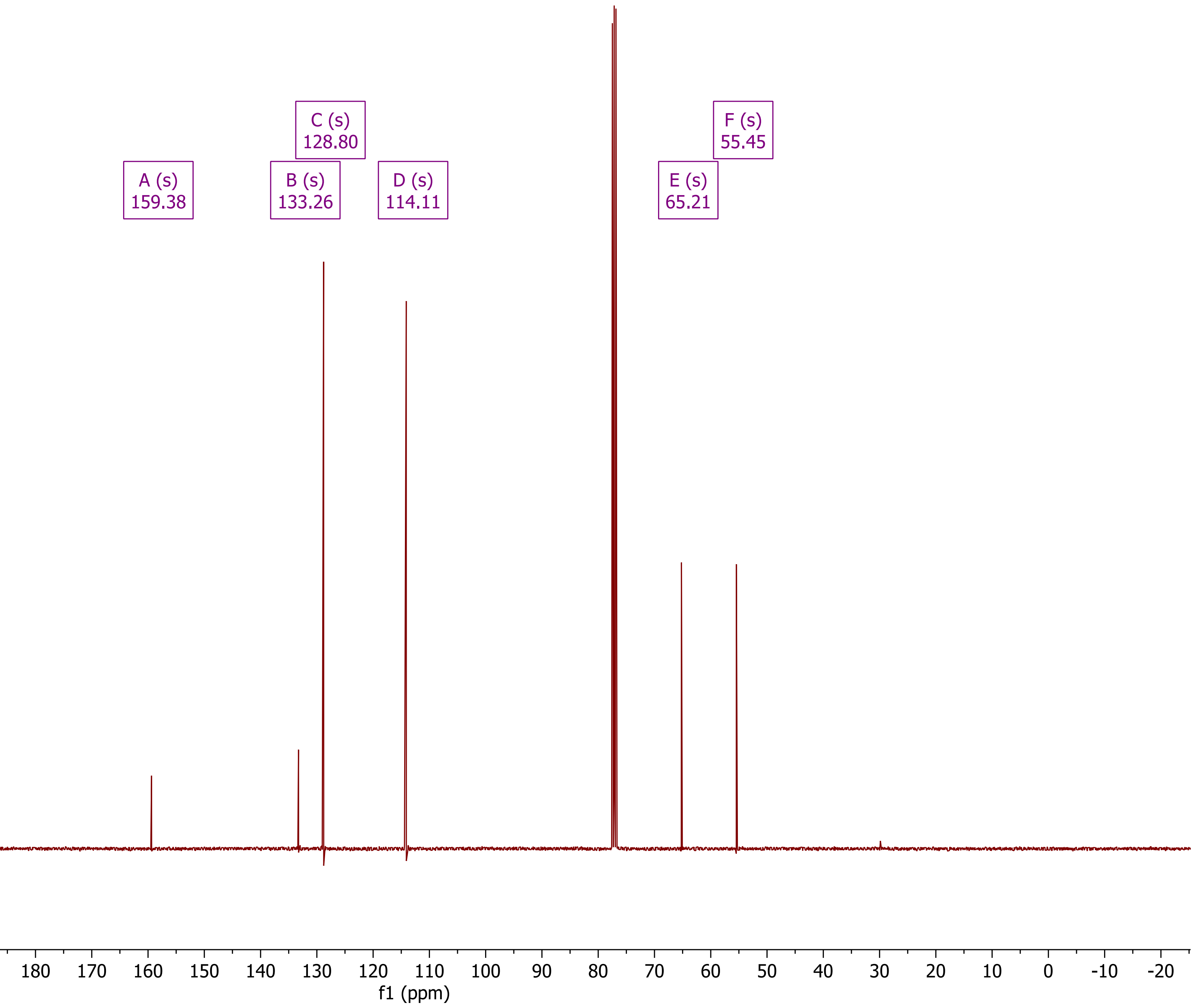

Compound SI-2:* 4-TIPS-butanoic acid, *¹H - NMR (CDCl$_3$, 400 MHz)

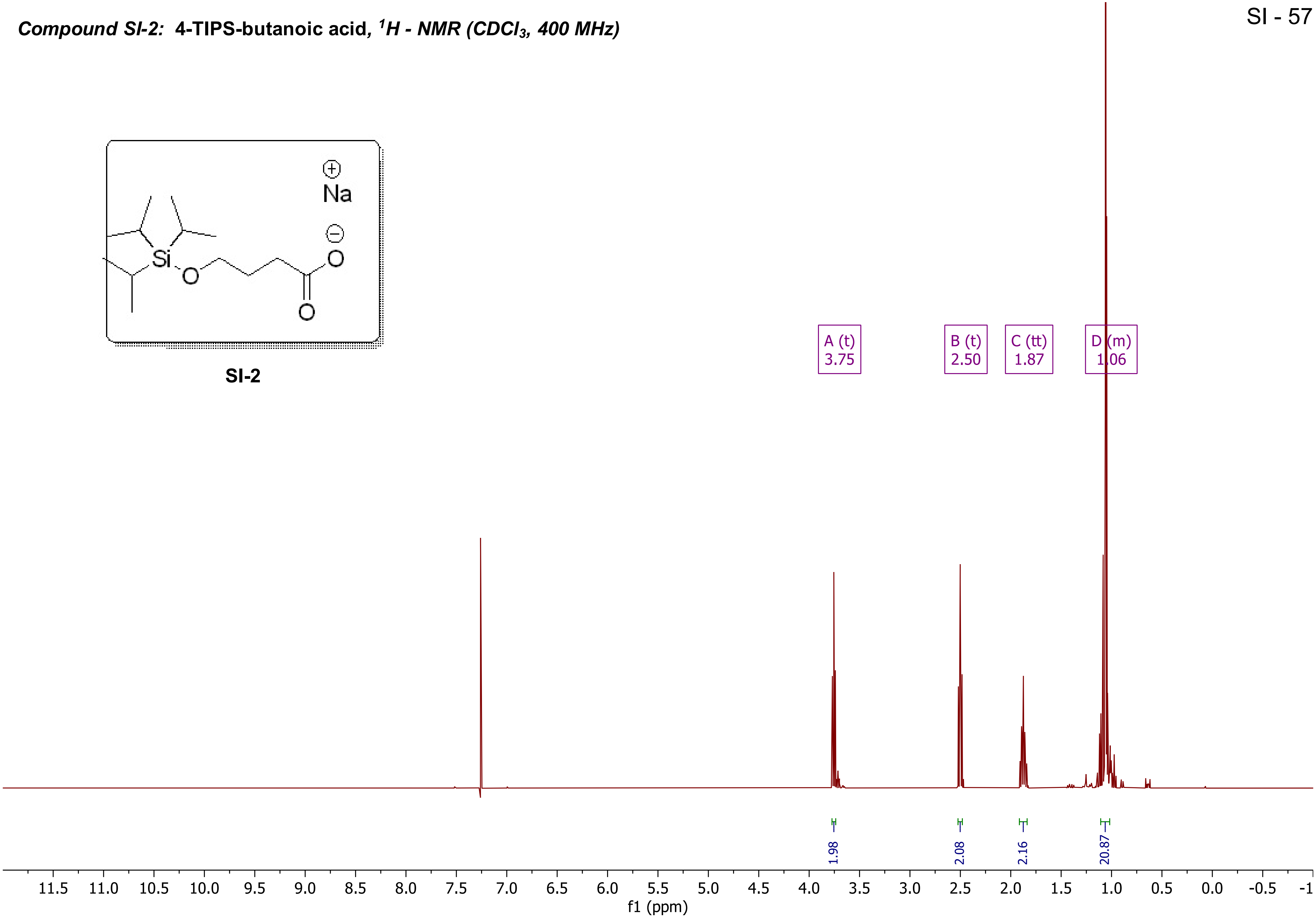

Compound SI-2: **4-TIPS-butanoic acid,** ***$^{13}C\{^1H\}$ - NMR ($CDCl_3$, 400 MHz)***

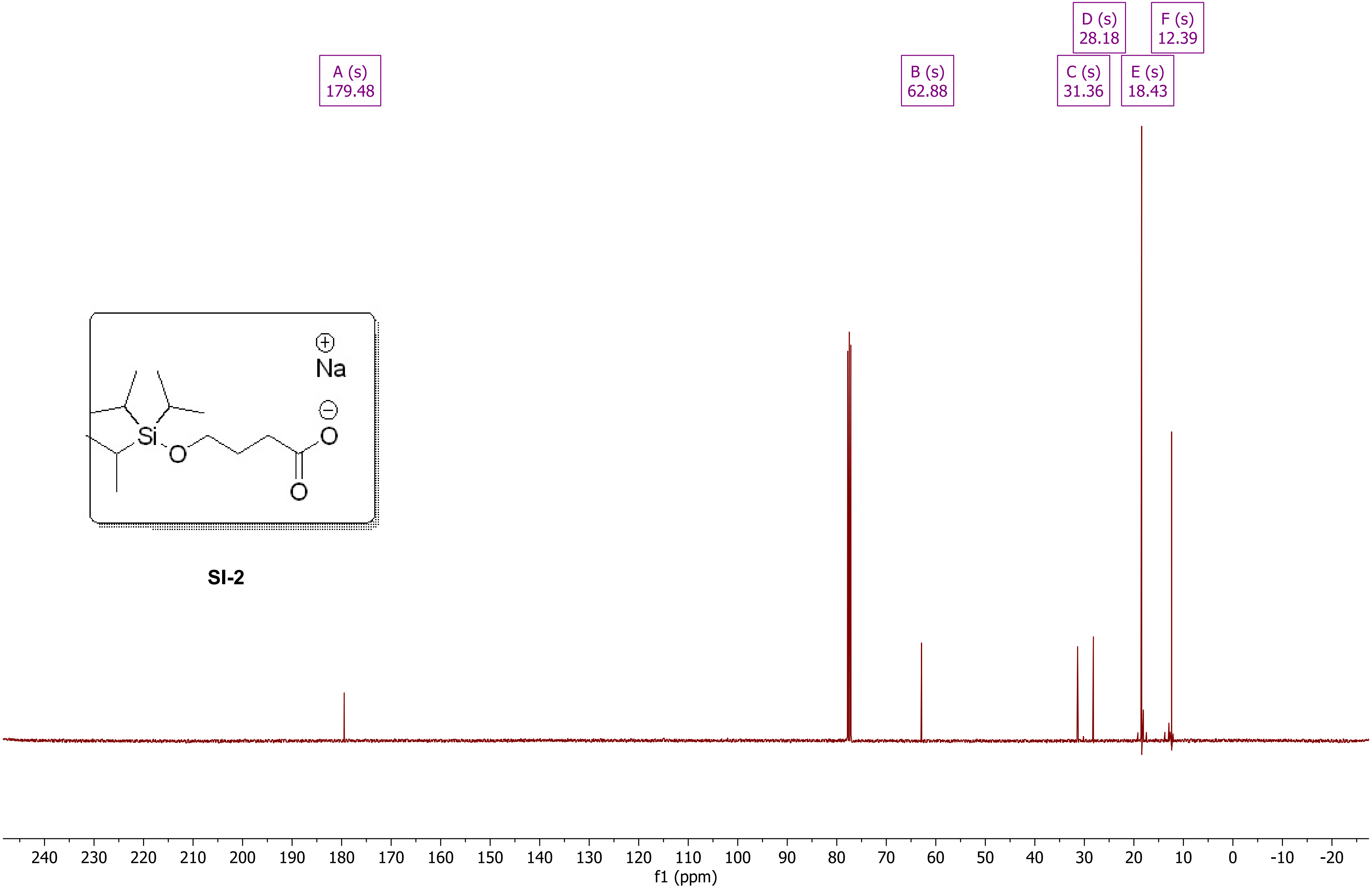

Compound 19:* 4-((hydroxyl)methyl)phenyl 4-(TIPSO)butanoate, *^{1}H - NMR ($CDCl_3$, 400 MHz)

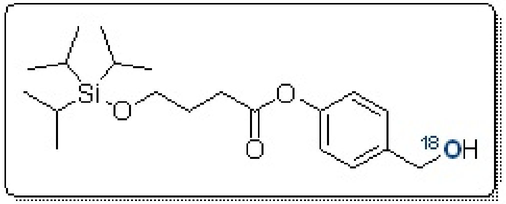

19

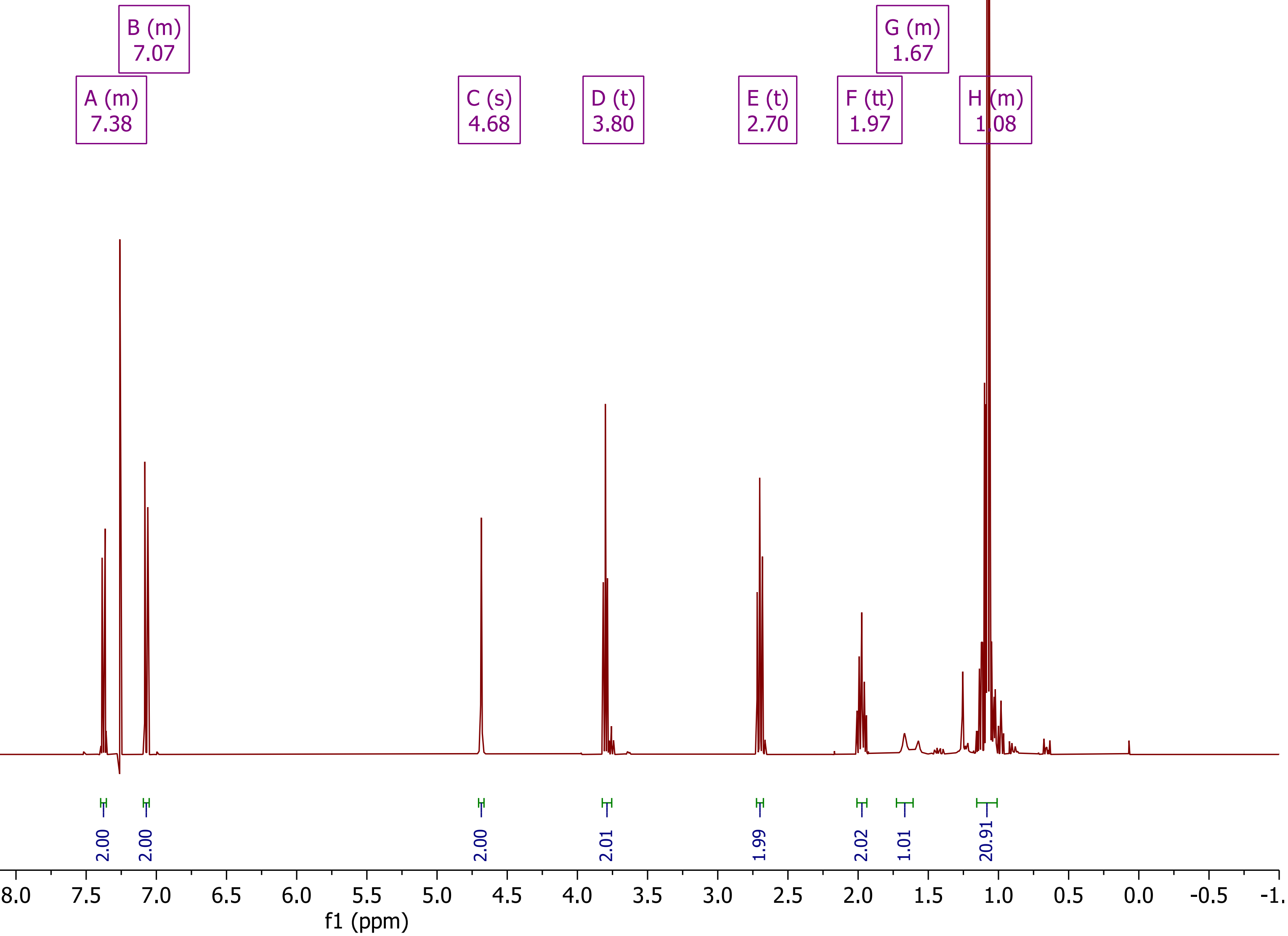

Compound 19:* 4-((hydroxyl)methyl)phenyl 4-(TIPSO)butanoate, *$^{13}C\{^1H\}$ - NMR ($CDCl_3$, 101 MHz)

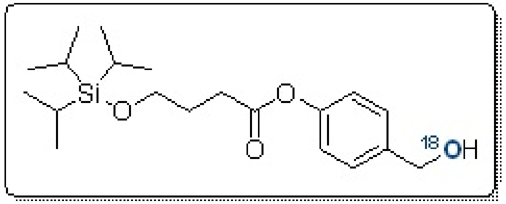

19

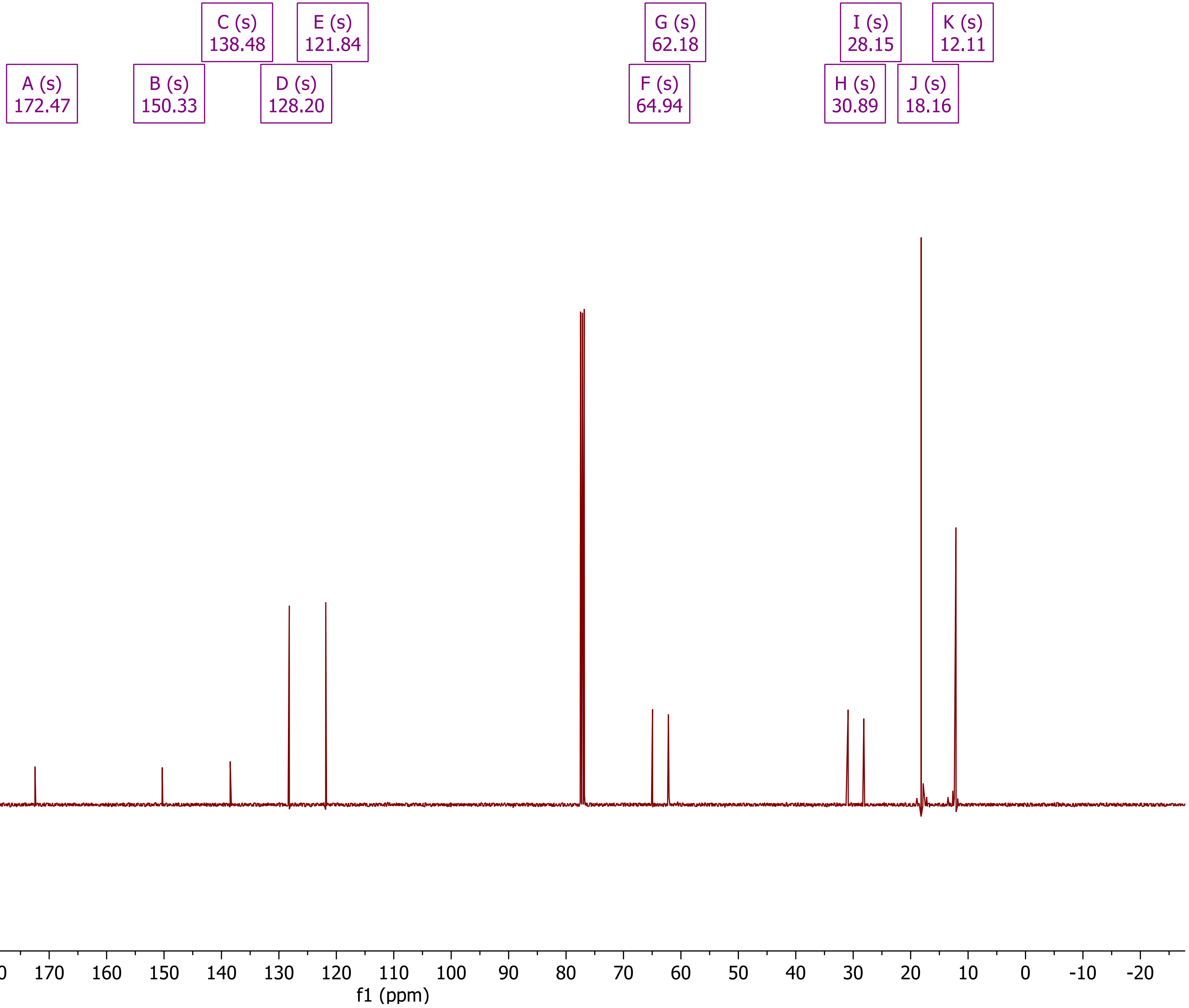

Compound 20: **^{18}O-BigFM-alcohol,** ***^{1}H - NMR ($CDCl_3$, 400 MHz)***

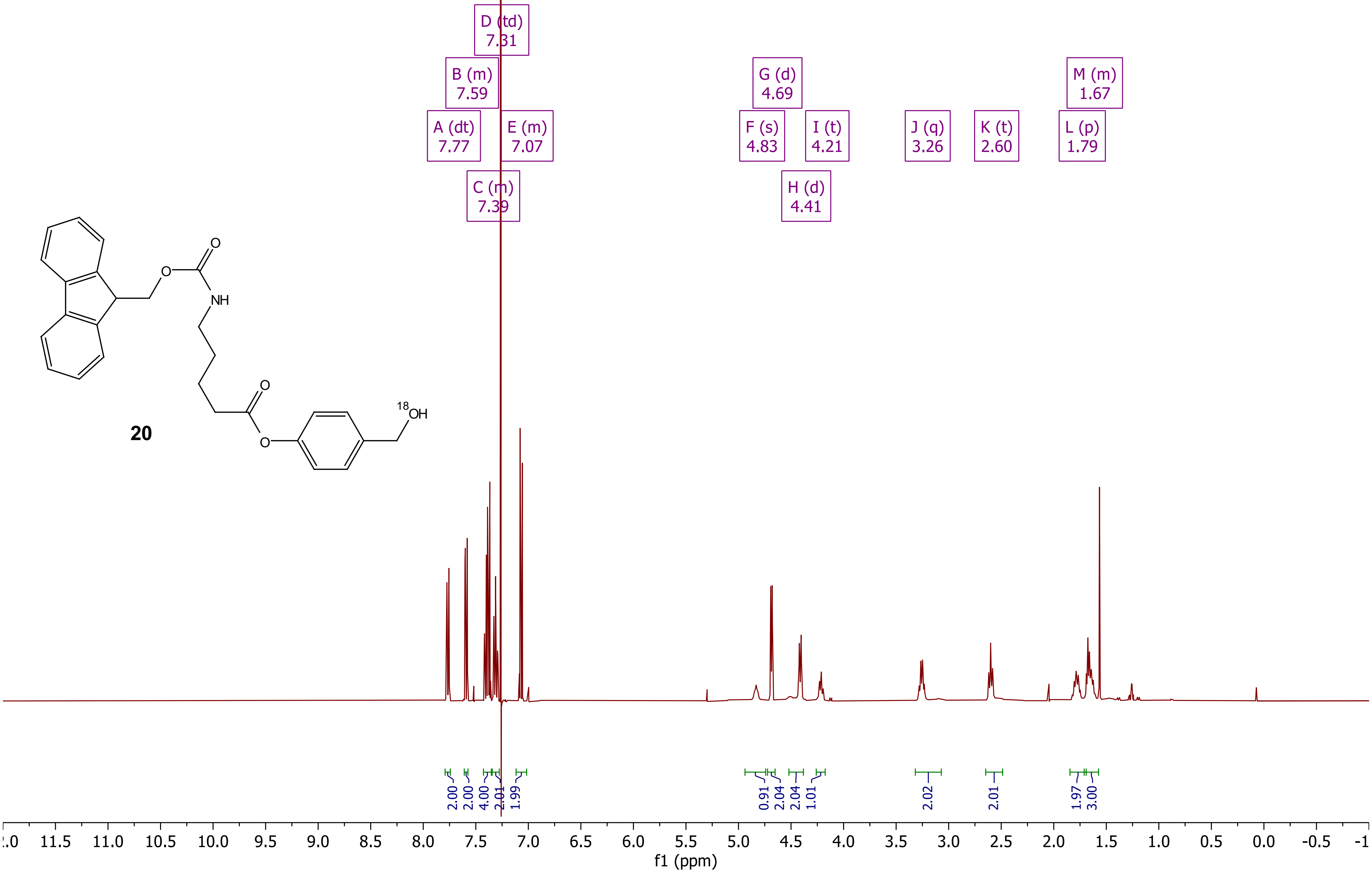

Compound 20: ^{18}**O-BigFM-alcohol,** $^{13}C\{^1H\}$ ***- NMR (CDCl$_3$, 101 MHz)***

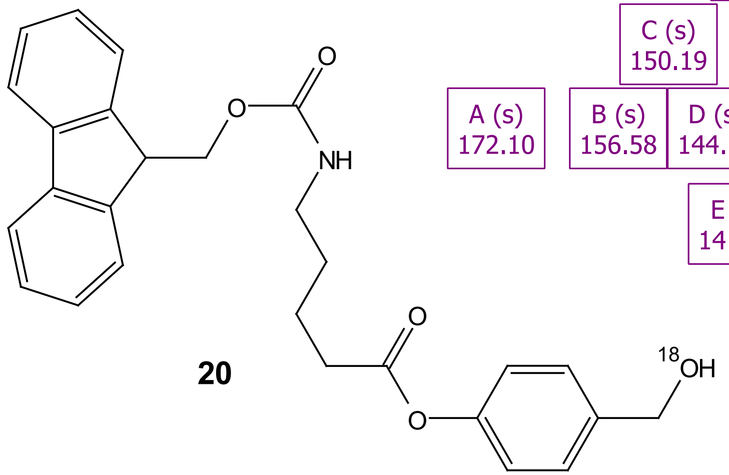

20

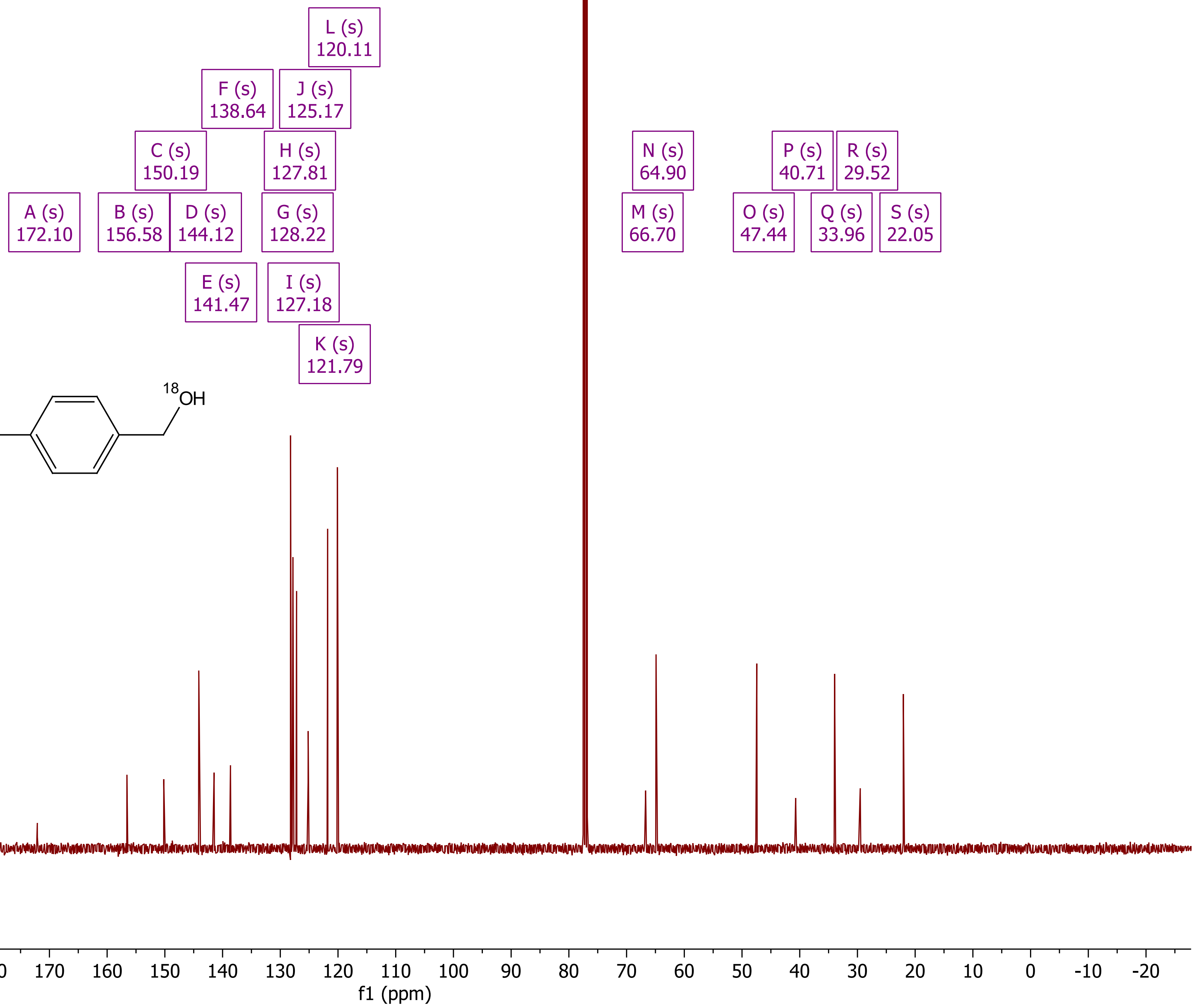

Compound 27:* (^{18}OAB)$_2$ P-N*i*Pr$_2$, ^{1}*H - NMR ($CDCl_3$, 400 MHz)

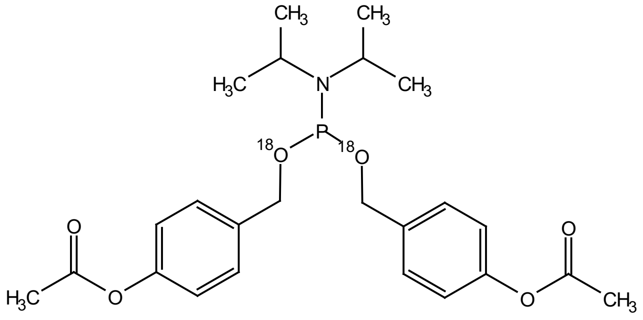

27

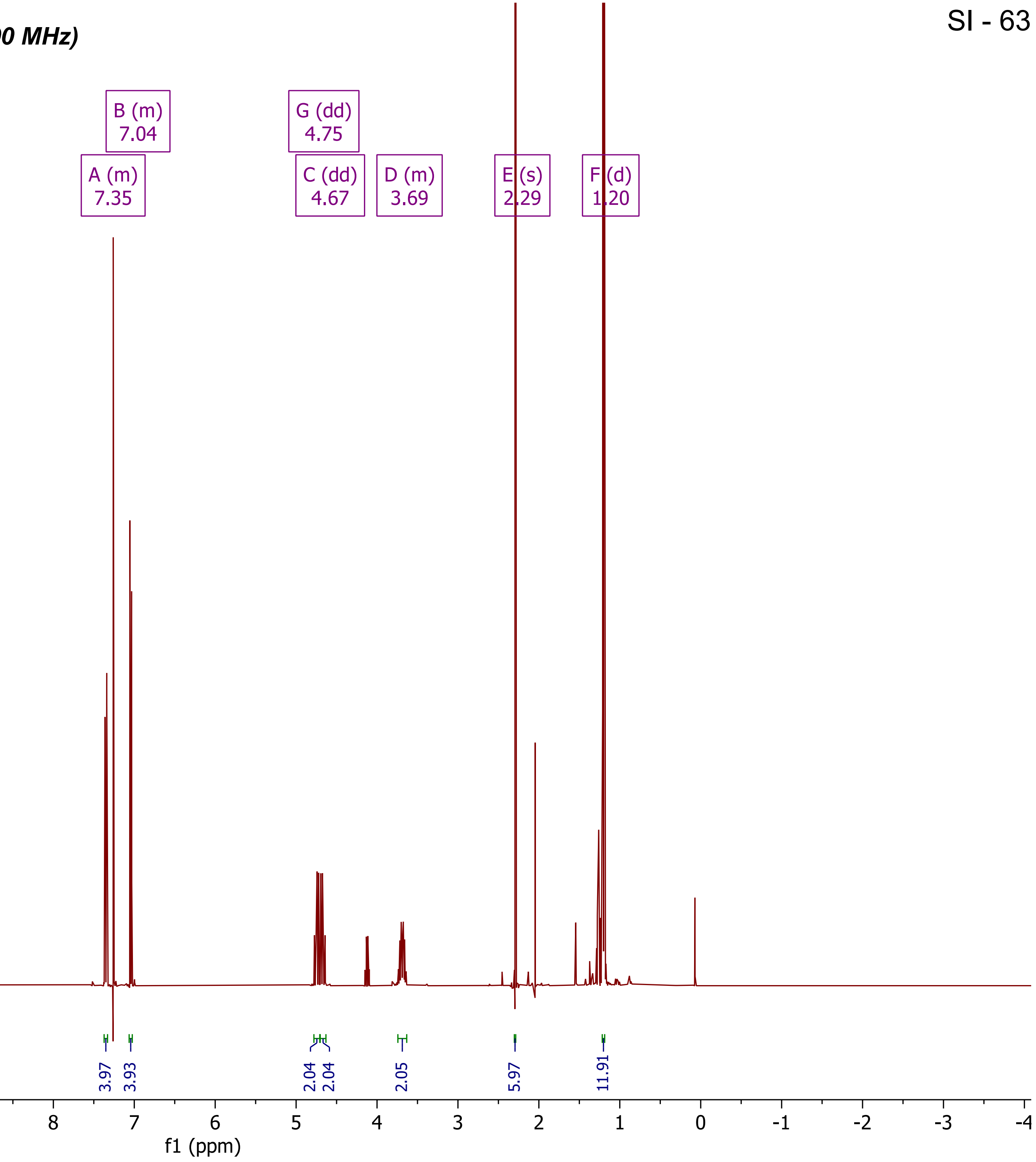

Compound 27: (^{18}OAB)$_2$ P-NiPr$_2$, ^{31}P{^{1}H} - NMR (CDCl$_3$, 162 MHz)

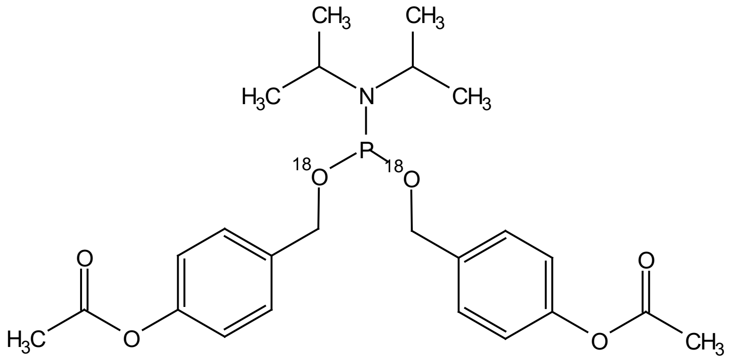

27

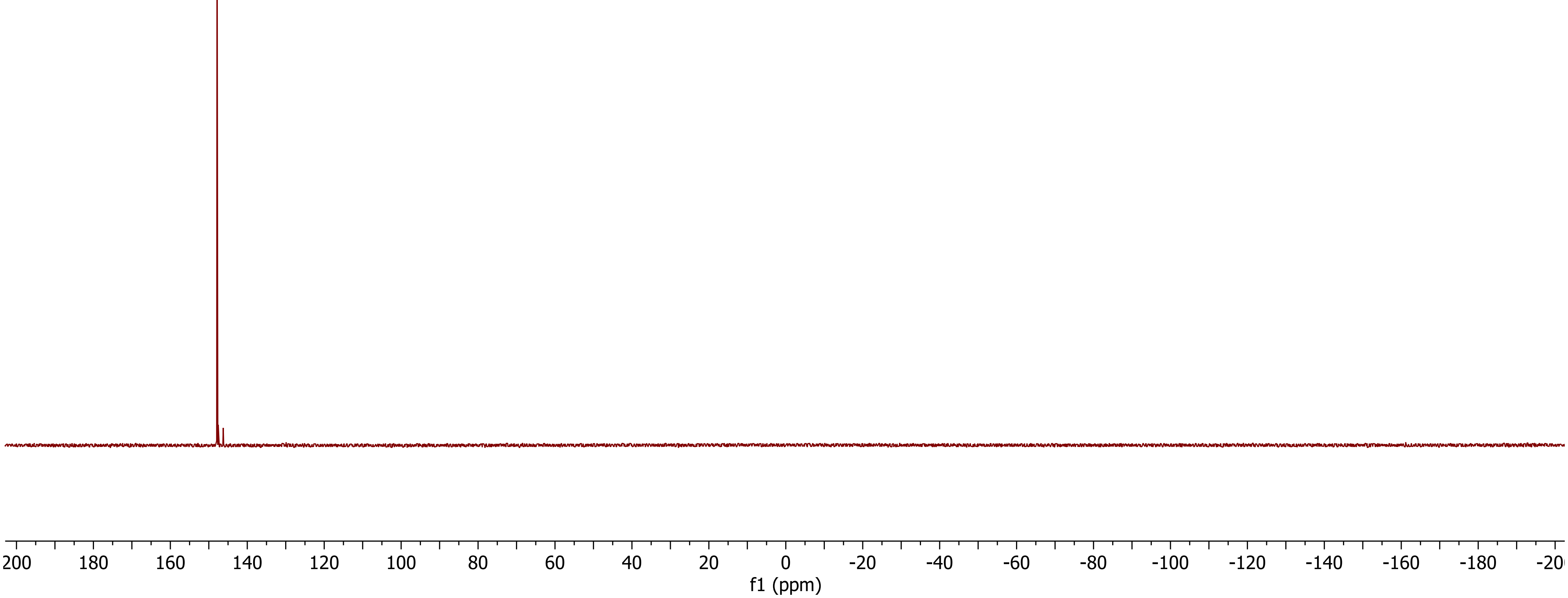

Compound 27:* (^{18}OAB)$_2$ P-N*i*Pr$_2$, *^{13}C{^{1}H} - NMR (CDCl$_3$, 101 MHz)

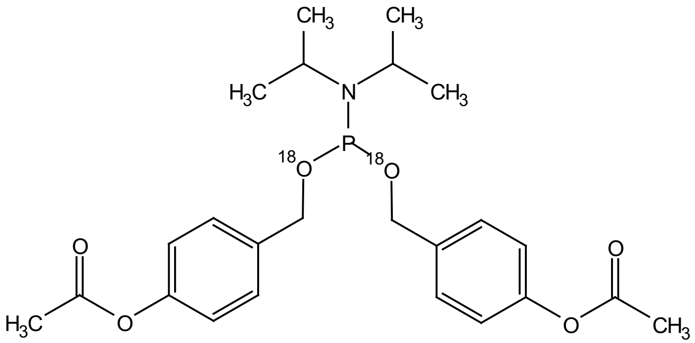

27

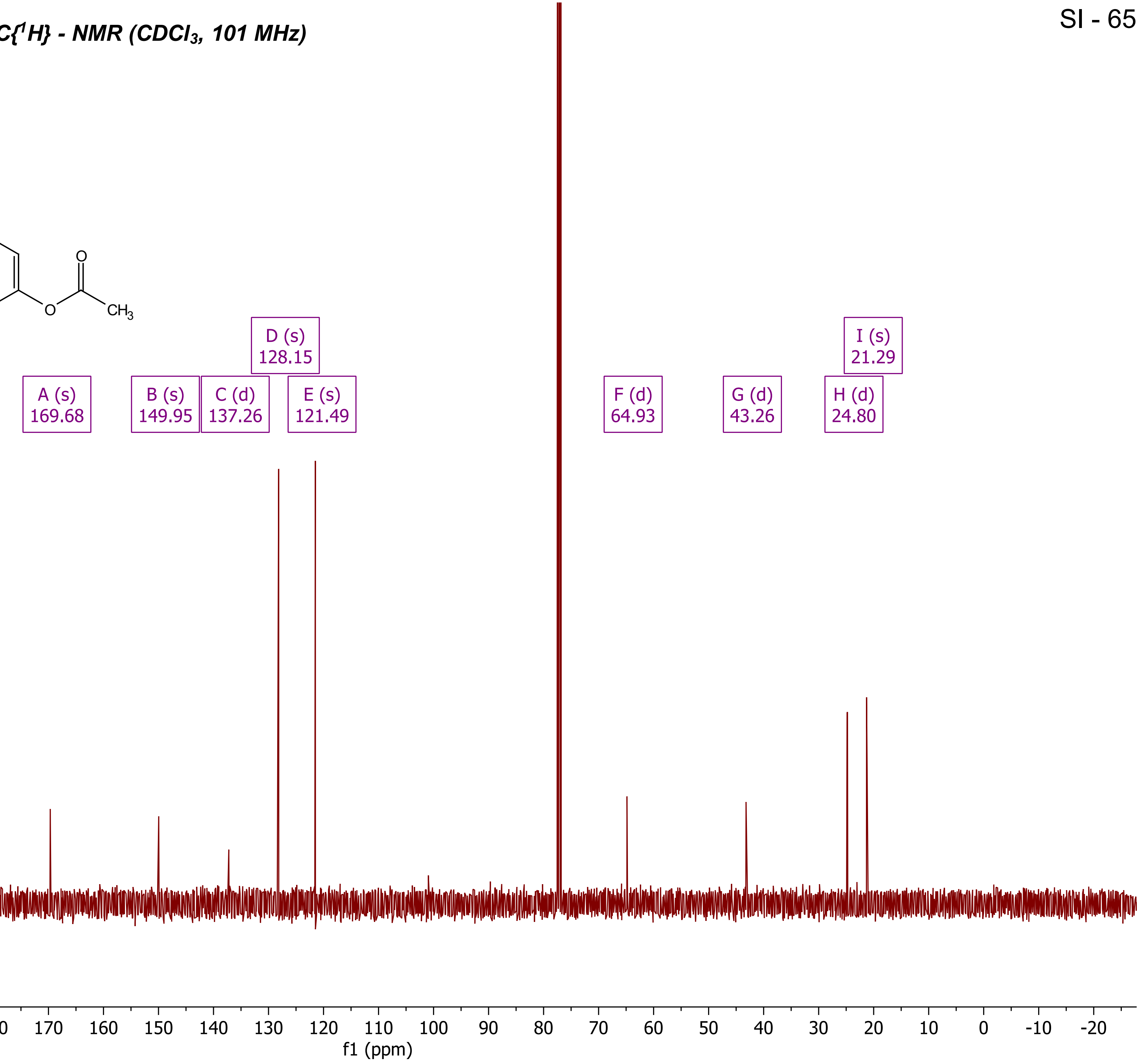

Compound 28:* (^{18}O-PMB)$_2$ P-N*i*Pr$_2$, *^{1}H - NMR (CDCl$_3$, 400 MHz)

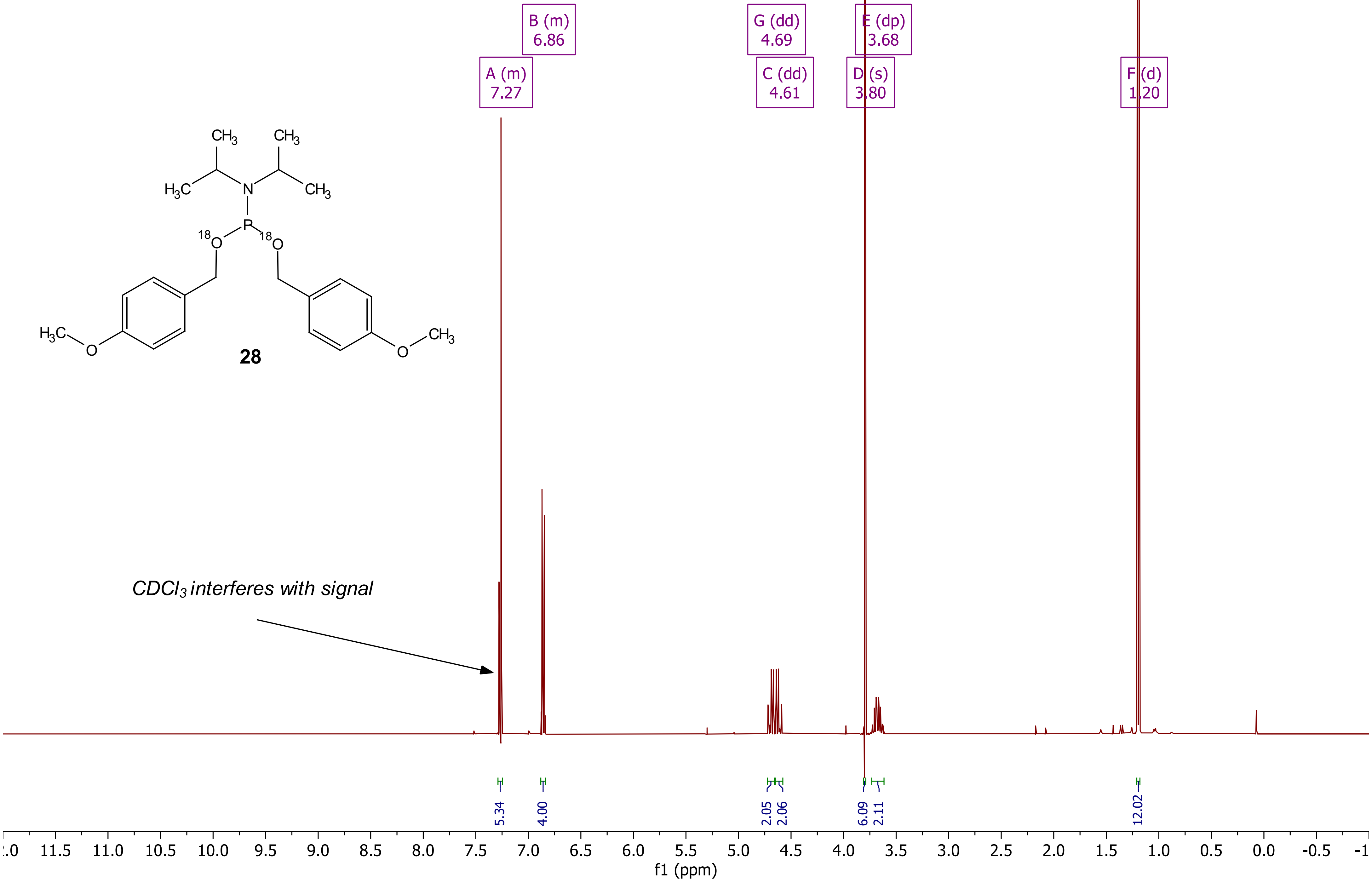

Compound 28:* (^{18}OPMB)$_2$ P-N*i*Pr$_2$, *^{31}P{^{1}H} - NMR (CDCl$_3$, 162 MHz)

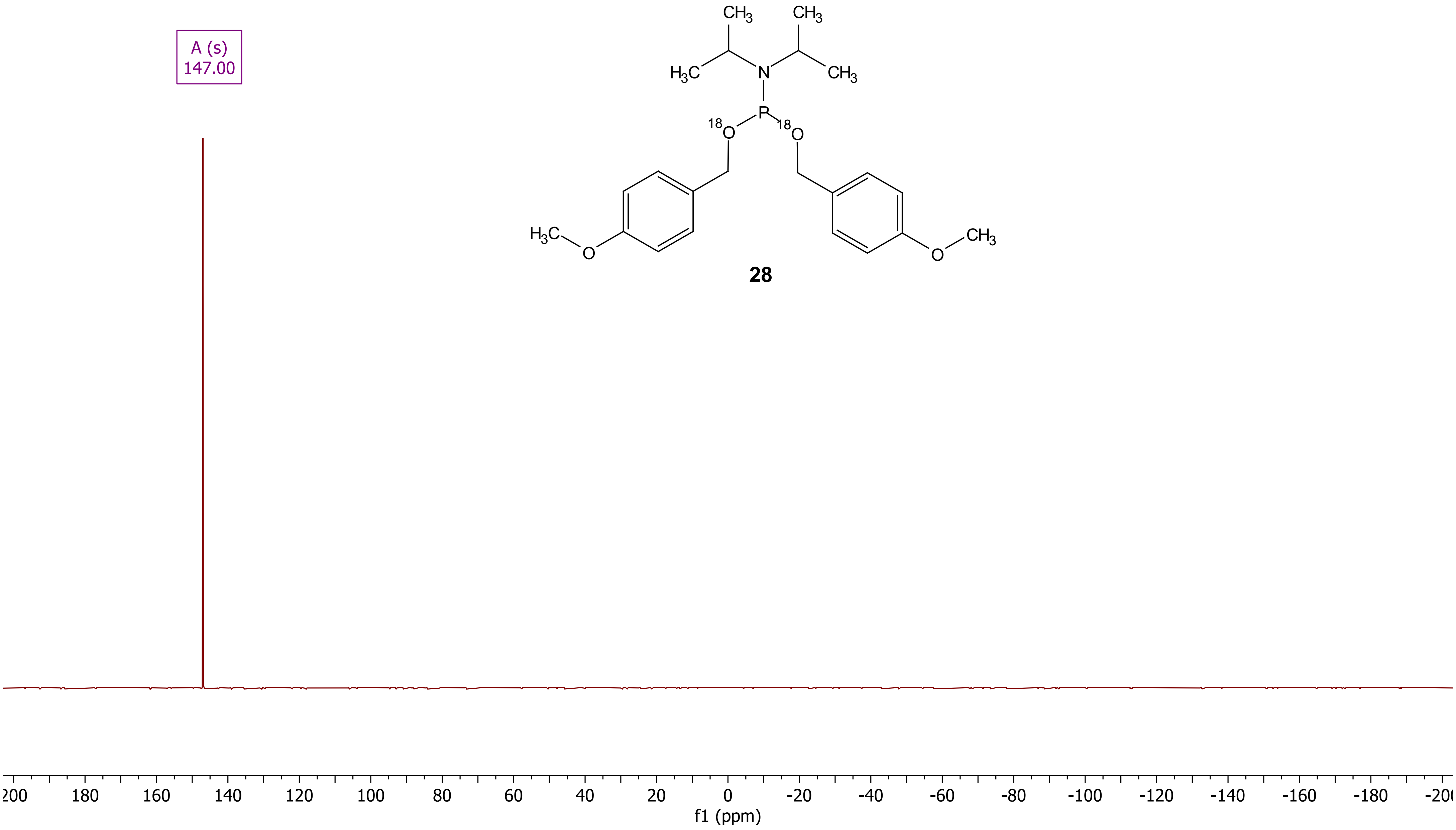

Compound 28:* (^{18}OPMB)$_2$ P-N*i*Pr$_2$, *^{13}C{^{1}H} - NMR (CDCl$_3$, 101 MHz)

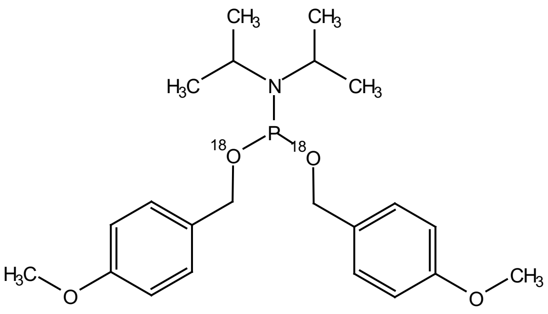

28

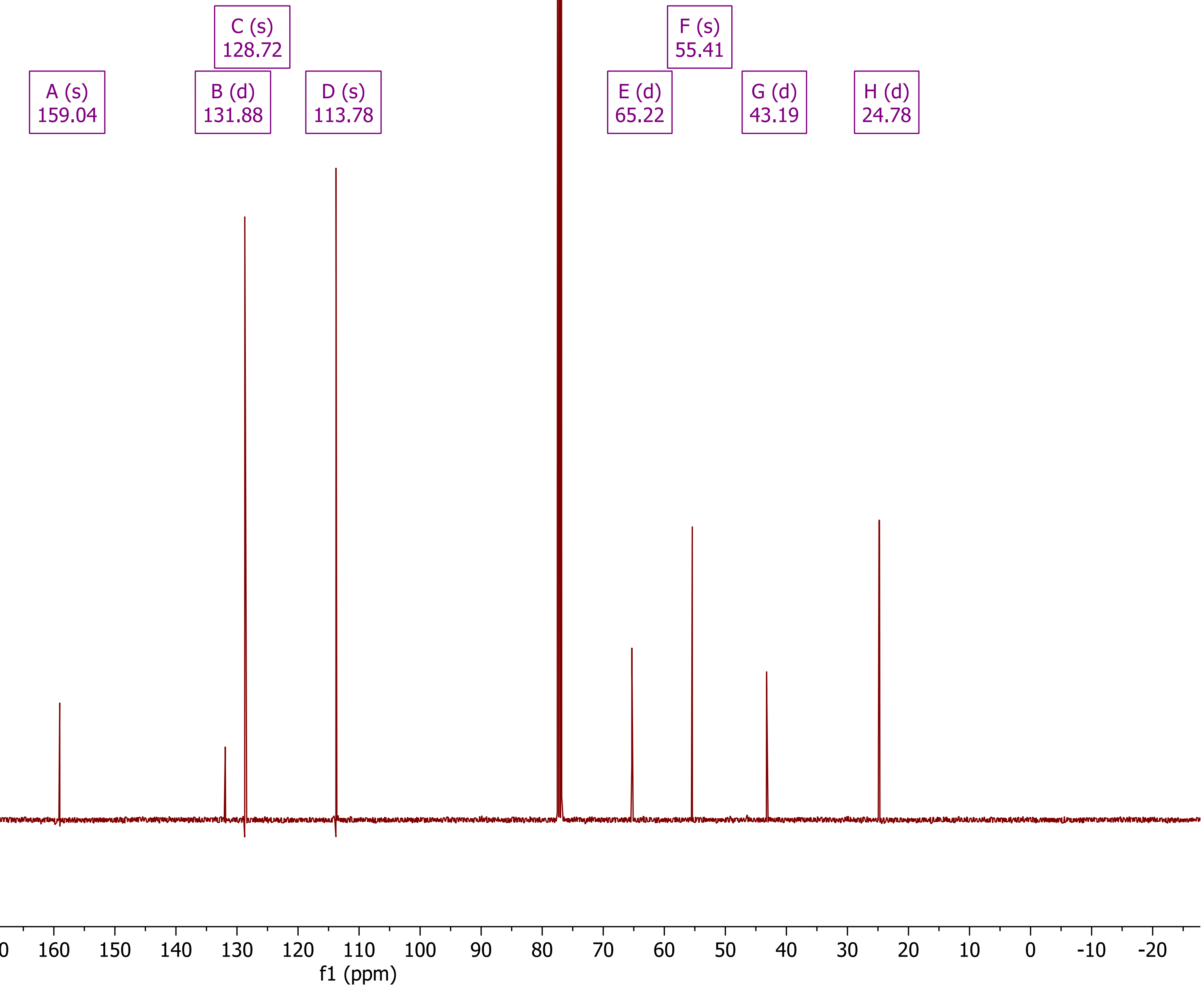

Compound 31:* ((((diisopropylamino)phosphanediyl)bis(oxy-^{18}O))bis(methylene))bis(4,1-phenylene) bis(4-TIPSO)butanoate), *^{1}H - NMR ($CDCl_3$, 400 MHz)

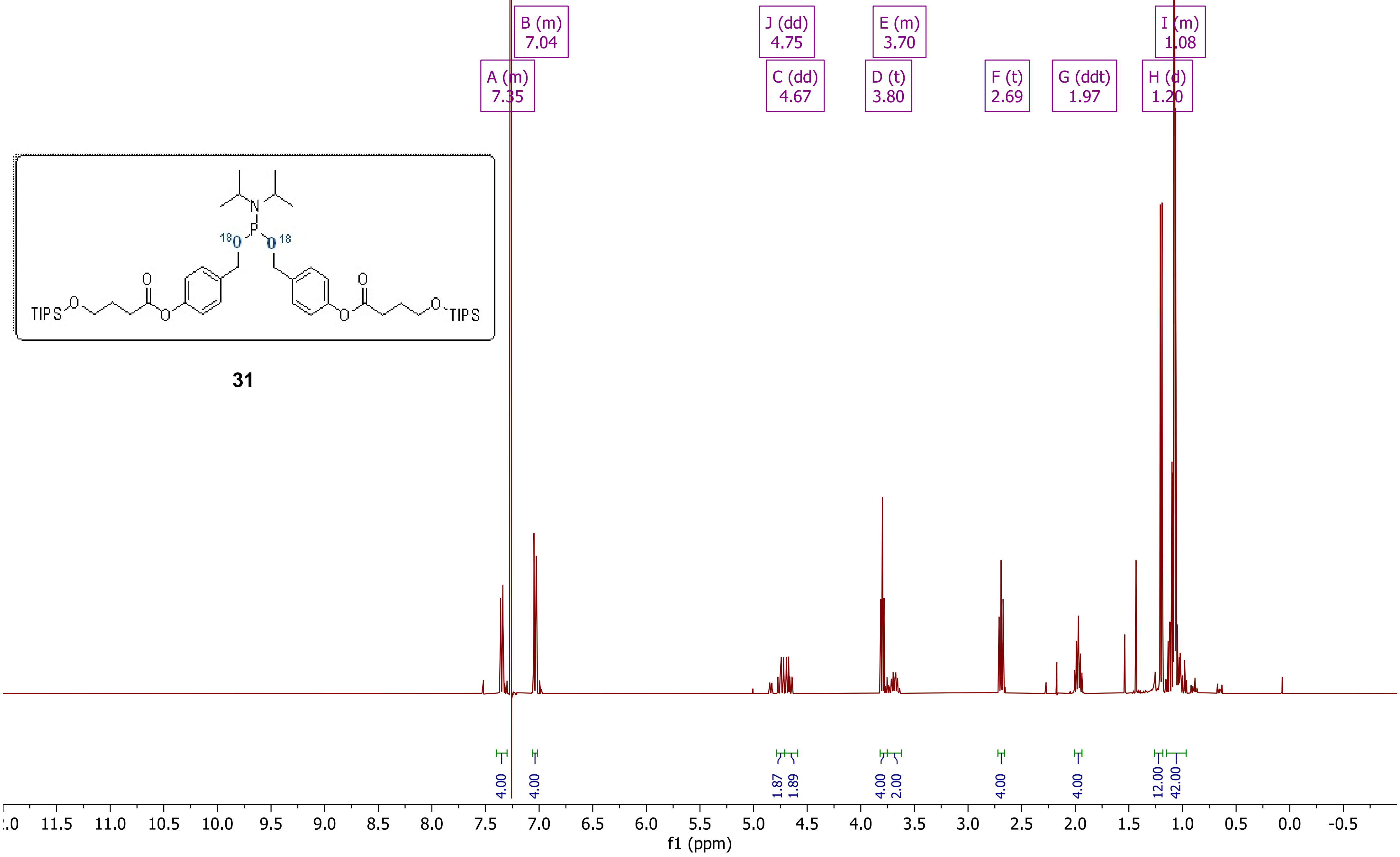

Compound 31:* ((((diisopropylamino)phosphanediyl)bis(oxy-^{18}O))bis(methylene))bis(4,1-phenylene) bis(4-TIPSO)butanoate), $^{13}C\{^{1}H\}$ - *NMR ($CDCl_3$, 101 MHz)

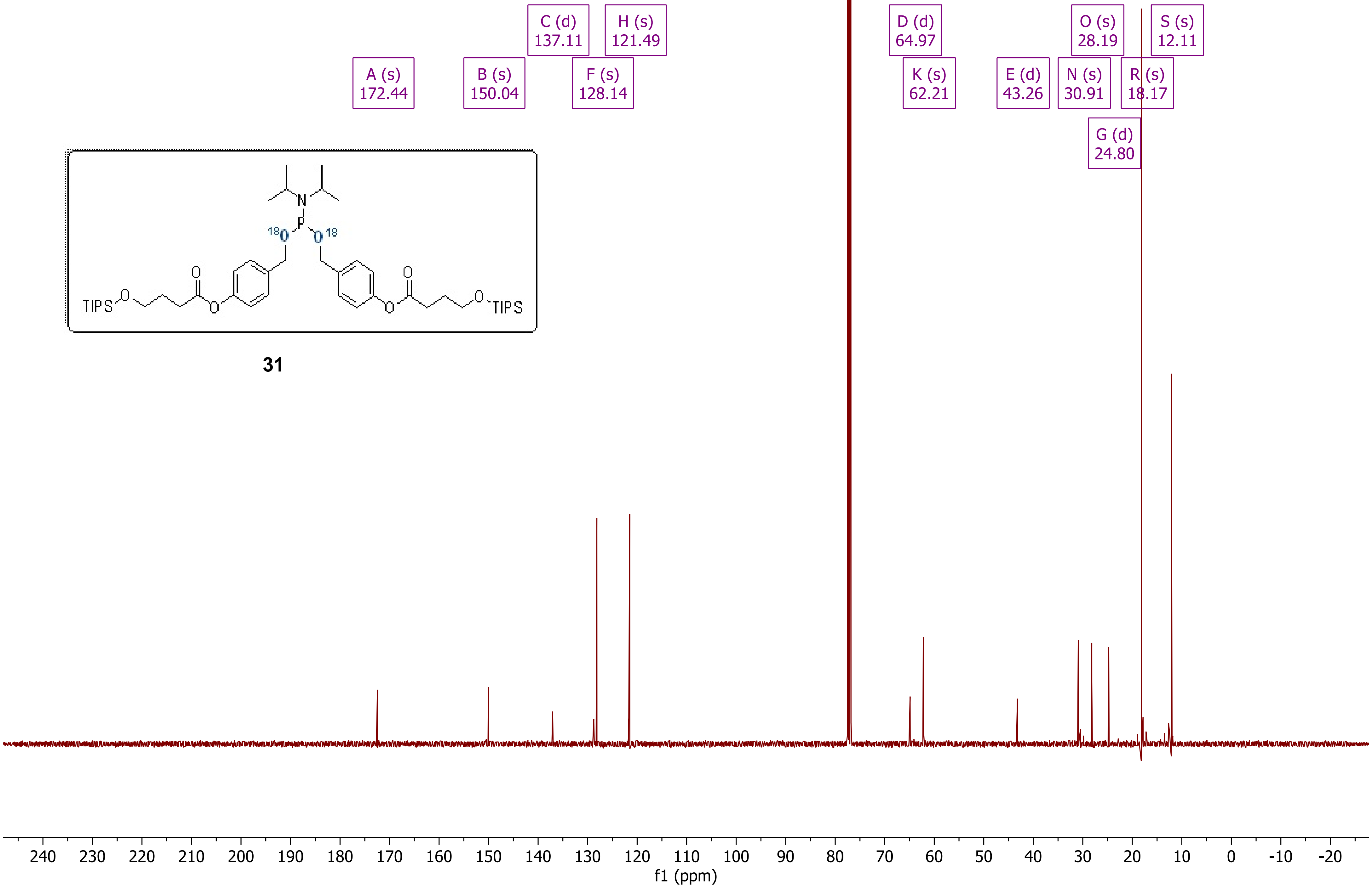

31

Compound 31:* ((((diisopropylamino)phosphanediyl)bis(oxy-^{18}O))bis(methylene))bis(4,1-phenylene) bis(4-TIPSO)butanoate), *^{31}P{^{1}H} - NMR (CDCl$_3$, 162 MHz)

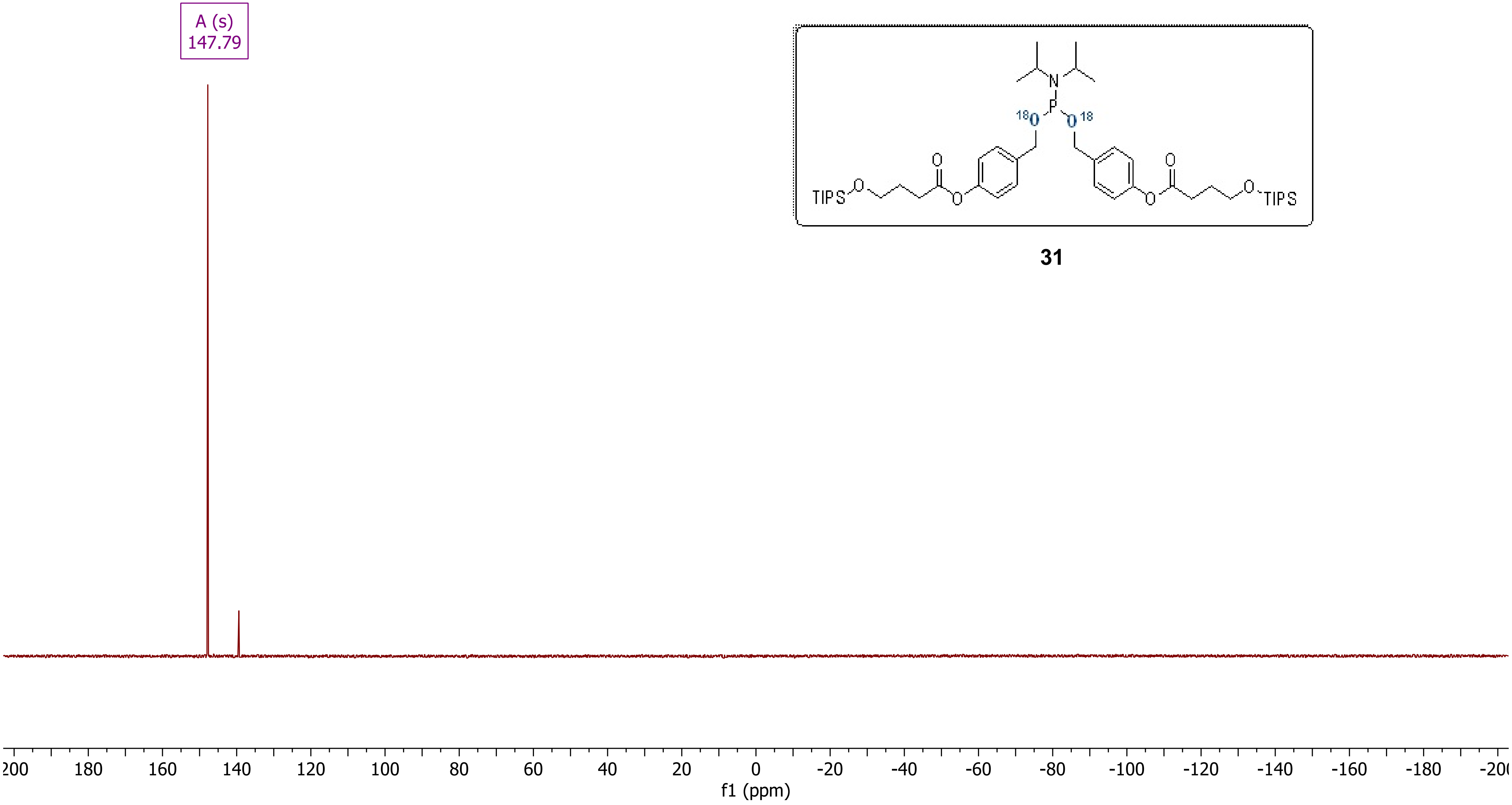

31

Compound 32: **((((diisopropylamino)phosphanediyl)bis(oxy-18O))bis(methylene))bis(4,1-phenylene) bis(5-((Fmoc)amino)pentanoate)**, ***^{1}H - NMR (CDCl$_3$, 400 MHz)***

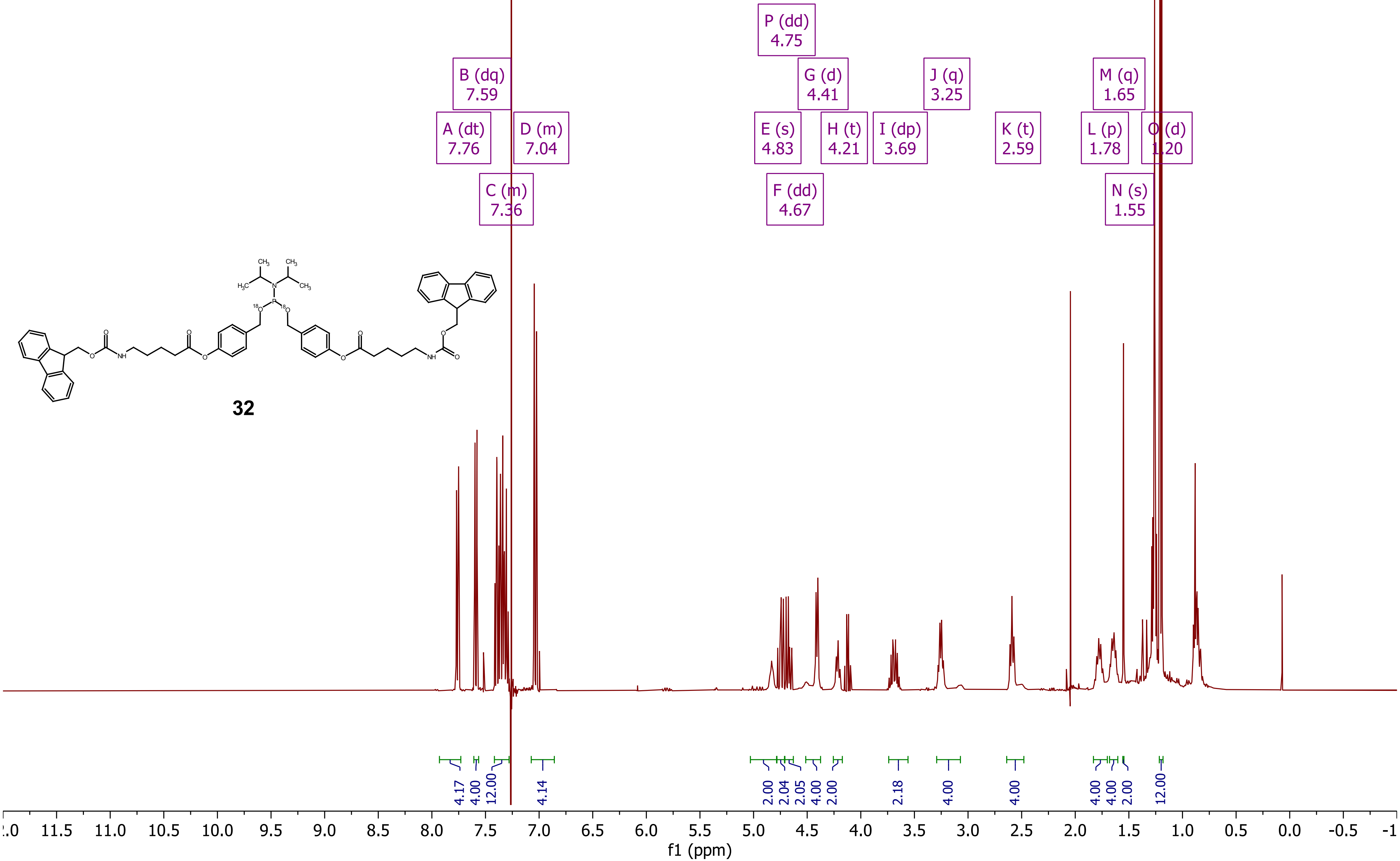

Compound 32: **((((diisopropylamino)phosphanediyl)bis(oxy-18O))bis(methylene))bis(4,1-phenylene) bis(5-((Fmoc)amino)pentanoate),**
***$^{13}C\{^{1}H\}$* - NMR ($CDCl_3$, 101 MHz)**

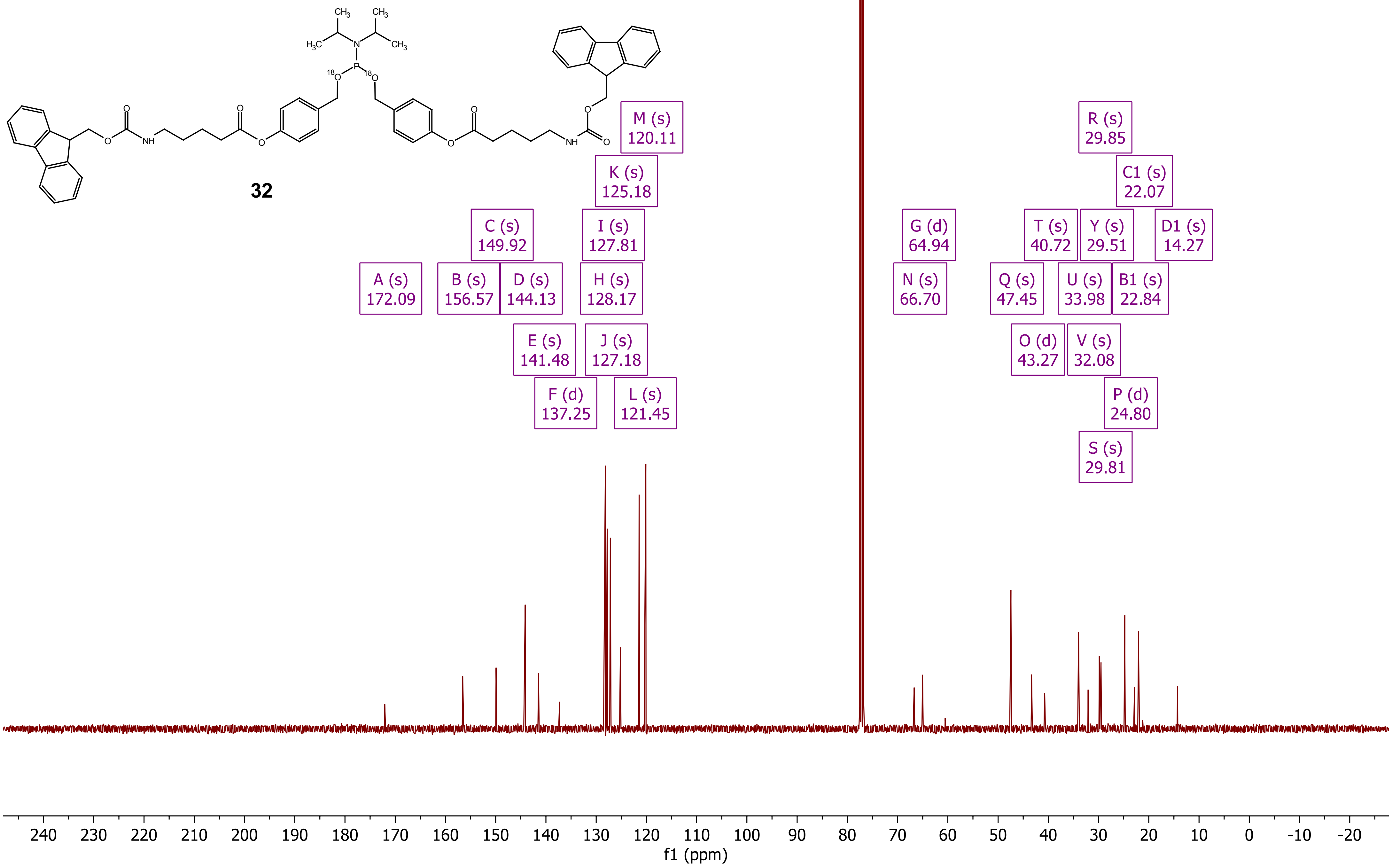

Compound 32:* ((((diisopropylamino)phosphanediyl)bis(oxy-18O))bis(methylene))bis(4,1-phenylene) bis(5-((Fmoc)amino)pentanoate), *$^{31}P\{^{1}H\}$ - NMR ($CDCl_3$, 162 MHz)

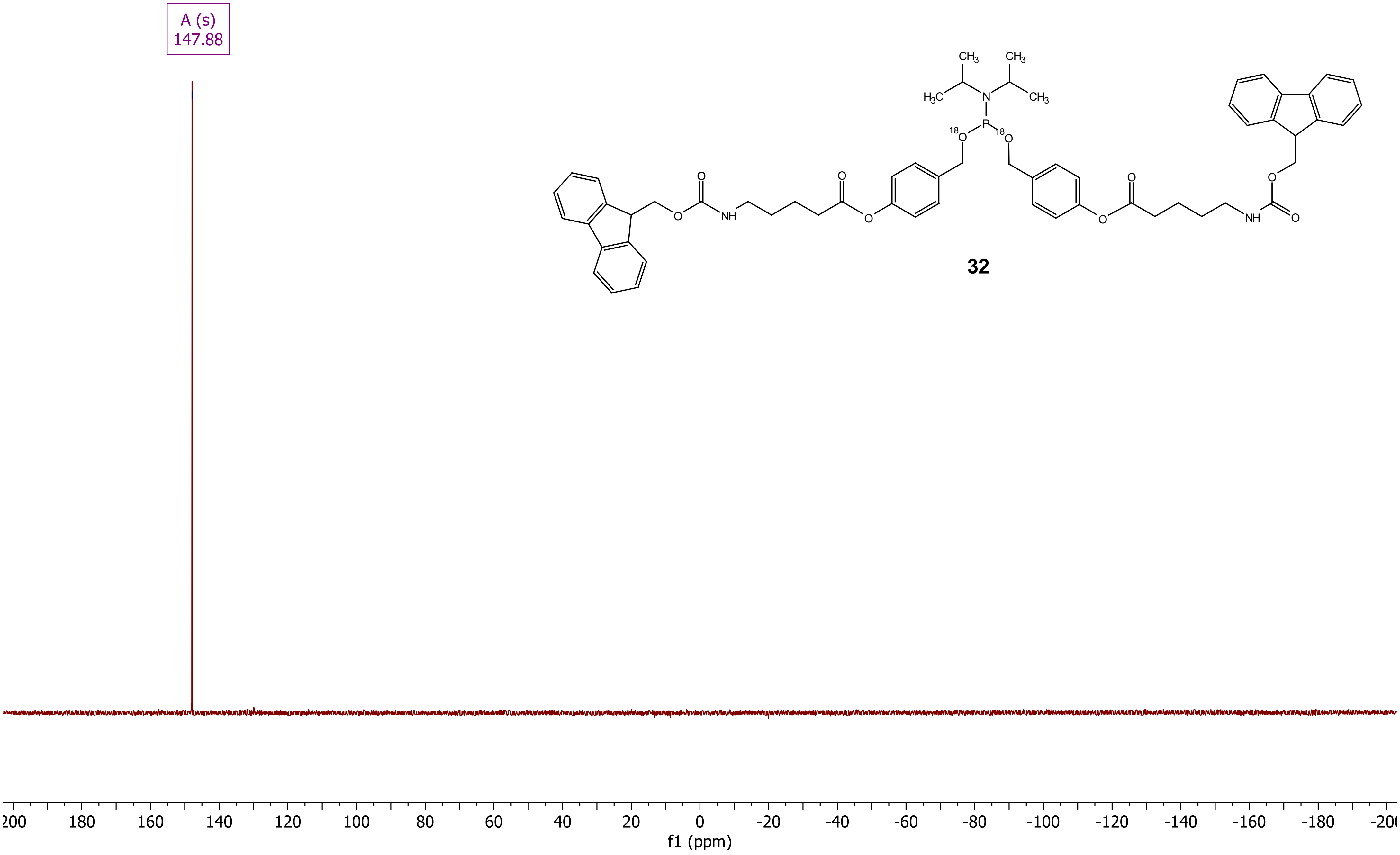

Compound 29:* (^{18}OAB)P-(N*i*Pr$_2$)$_2$, *^{1}H - NMR (CDCl$_3$, 400 MHz)

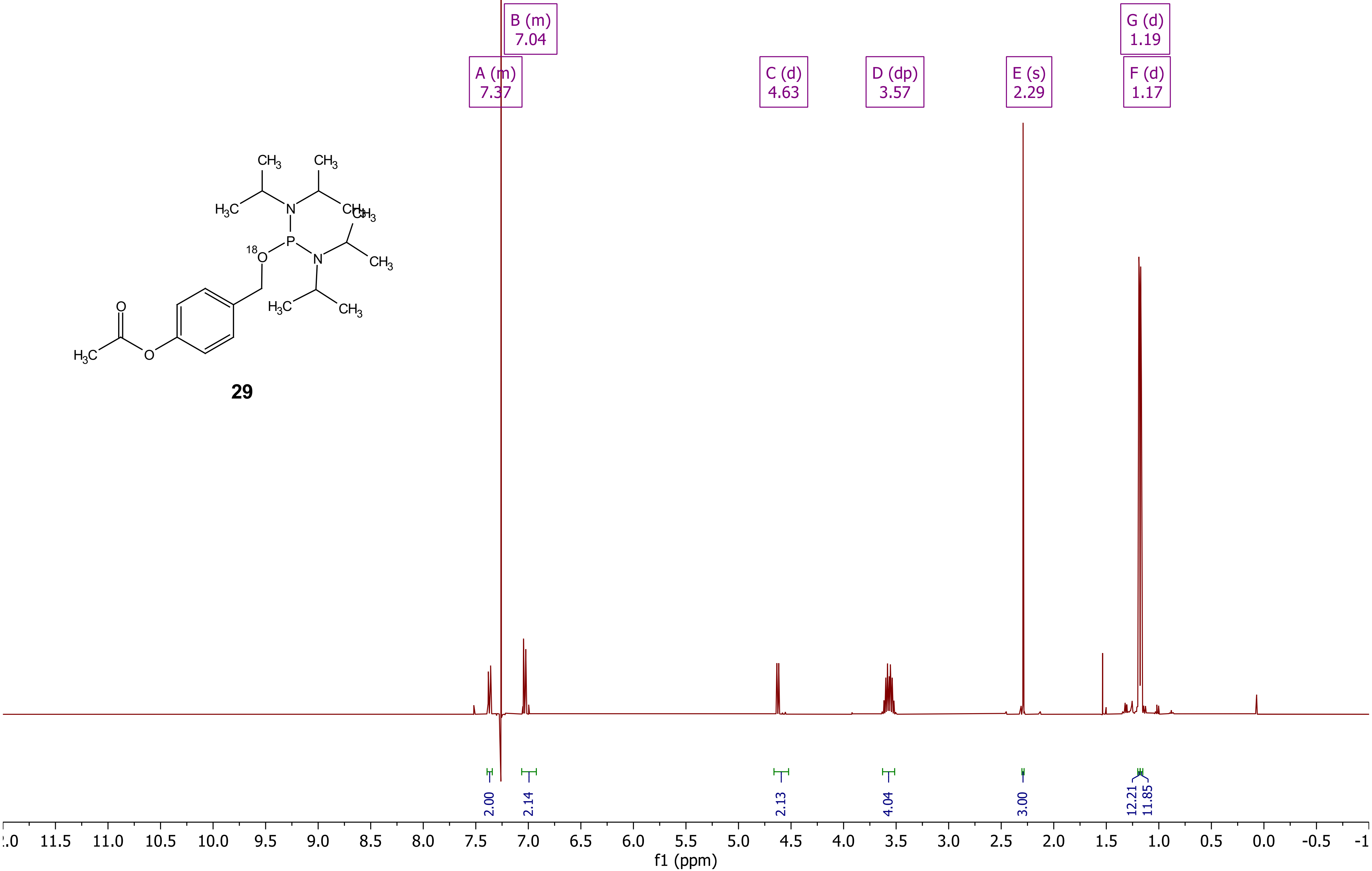

Compound 29:* ([18]OAB)P-(N*i*Pr$_2$)$_2$, *^{31}P{^{1}H} - NMR (CDCl$_3$, 162 MHz)

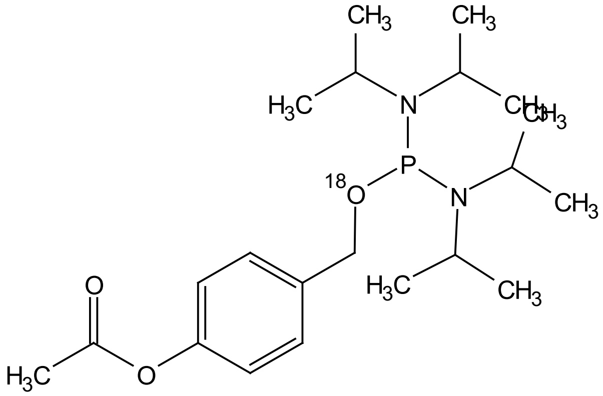

29

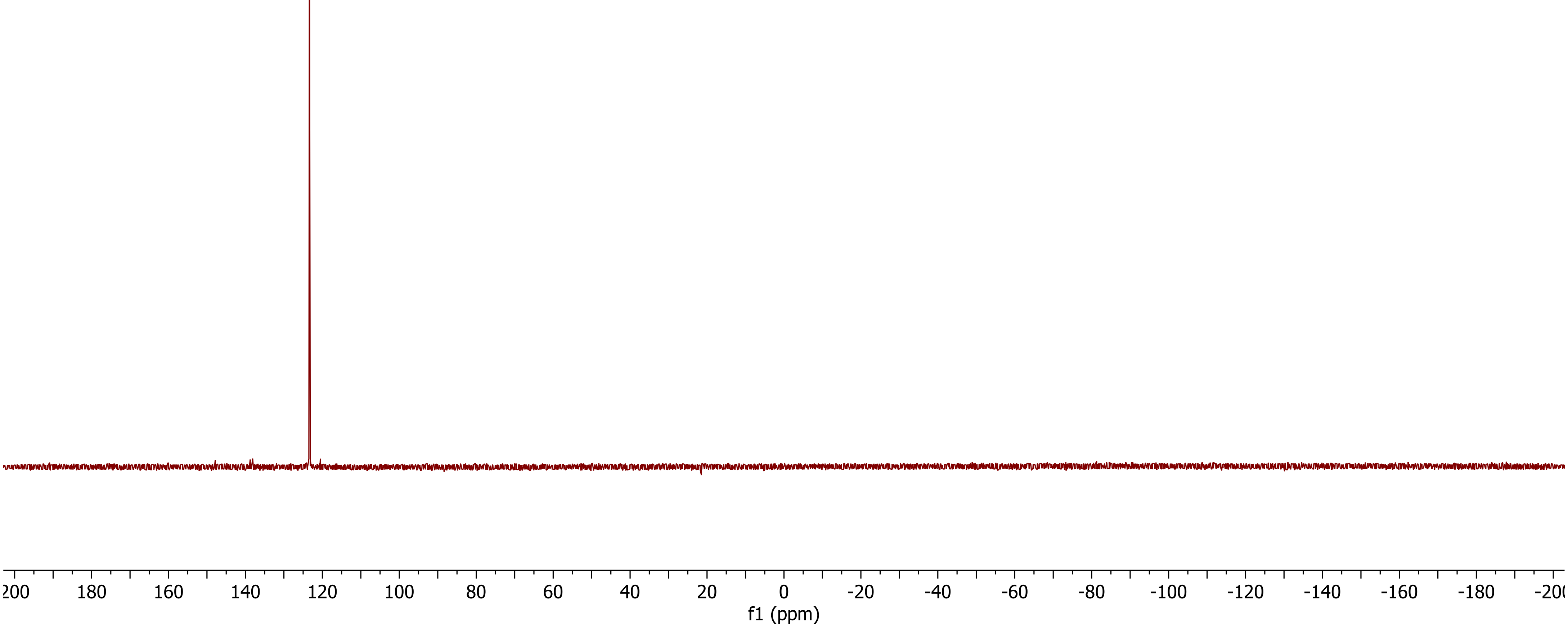

Compound 29:* (^{18}OAB)P-(N*i*Pr$_2$)$_2$, *^{13}C{^{1}H} - NMR (CDCl$_3$, 101 MHz)

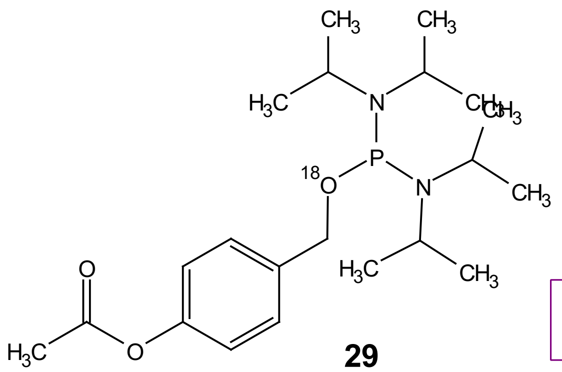

29

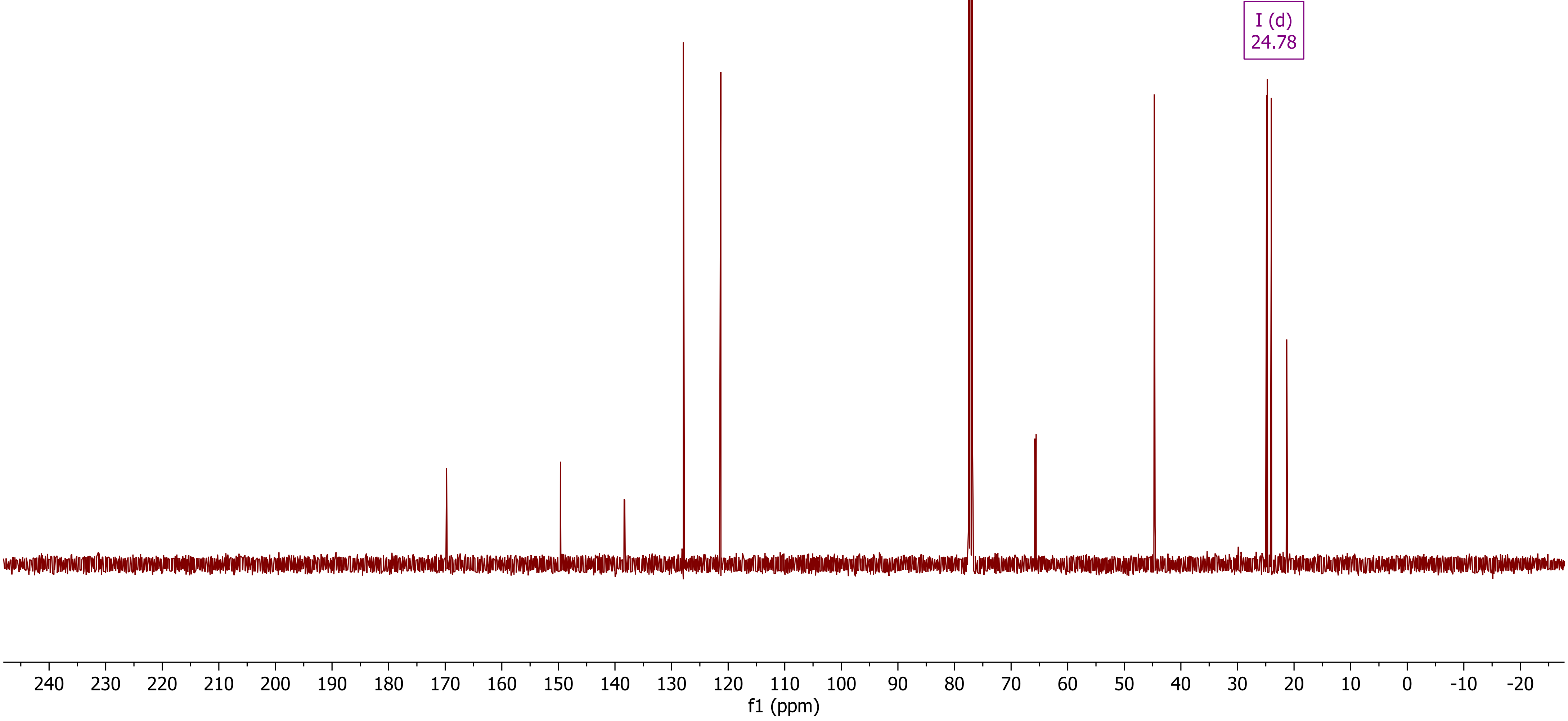

Compound 30:* (^{18}OAB)(^{18}OPMB)P-N*i*Pr$_2$, ^{1}*H - NMR (CDCl$_3$, 400 MHz)

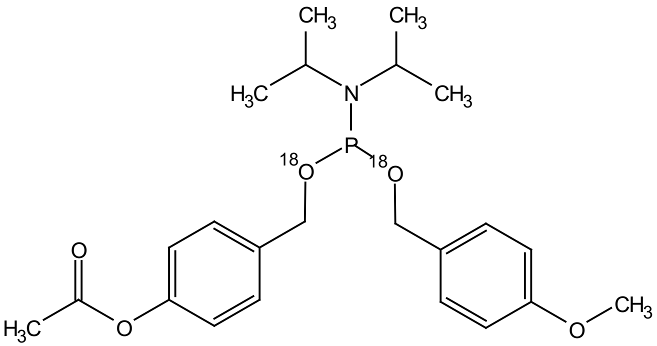

30

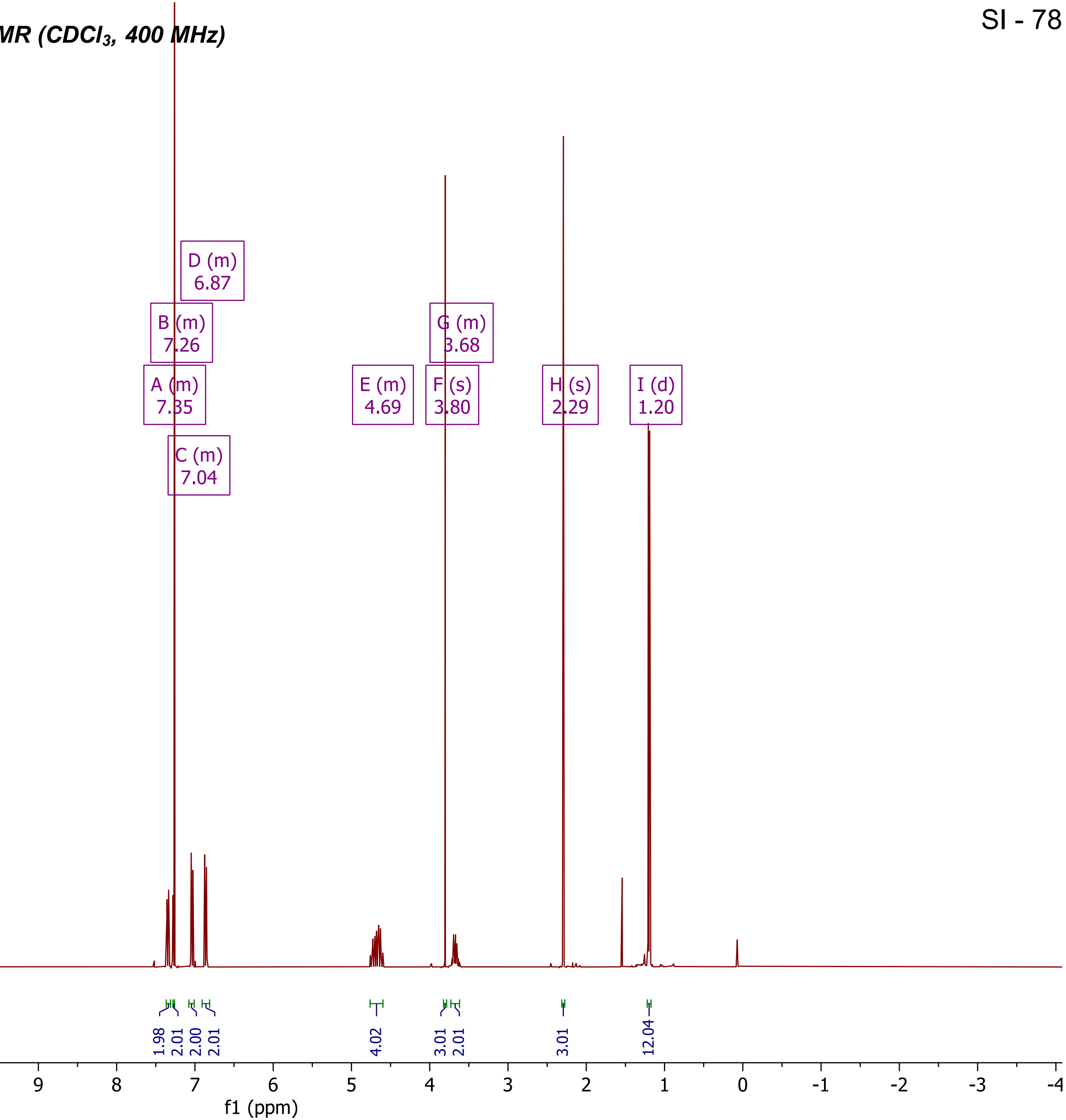

Compound 30:* (^{18}OAB)(^{18}OPMB)P-N*i*Pr$_2$, *^{31}P{^{1}H} - NMR ($CDCl_3$, 162 MHz)

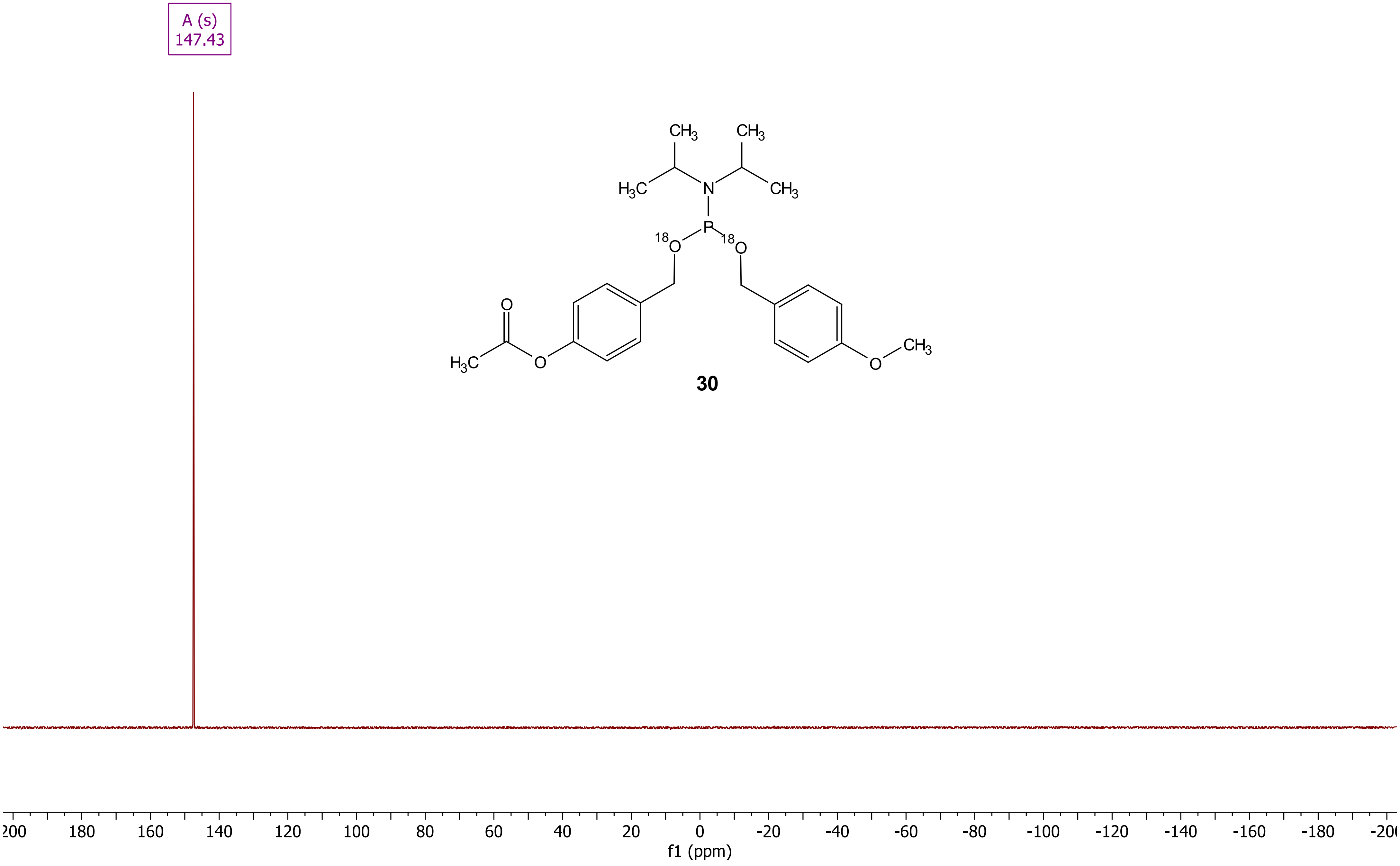

Compound 30:* (^{18}OAB)(^{18}OPMB)P-N*i*Pr$_2$, *^{13}C{^{1}H} - NMR (CDCl$_3$, 101 MHz)

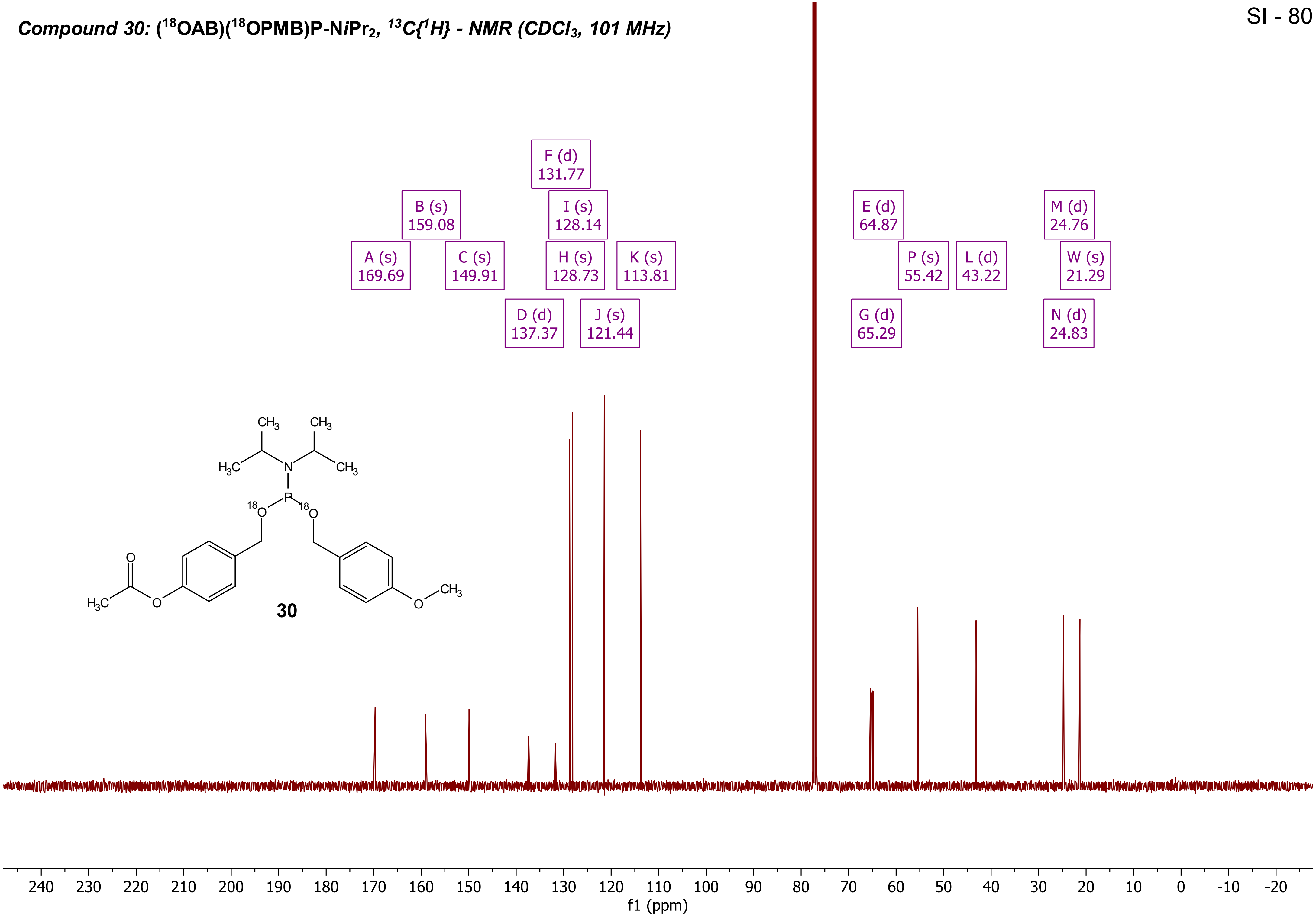

Compound 33: ^{18}O-DNA-precursor P-amidite, ^{1}H - NMR ($CDCl_3$, 400 MHz)

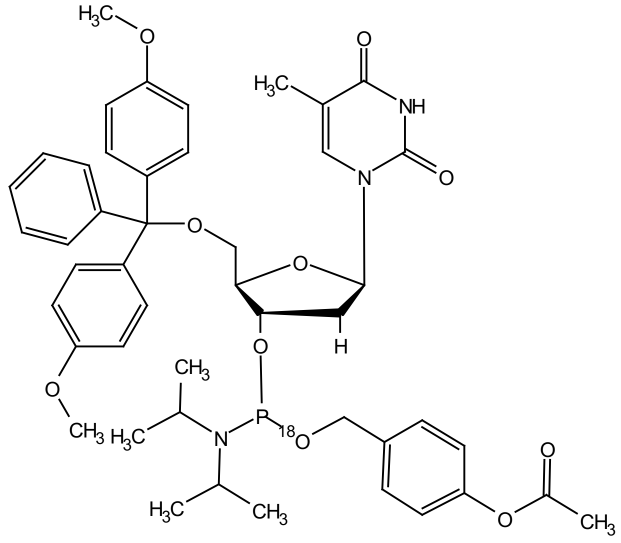

33

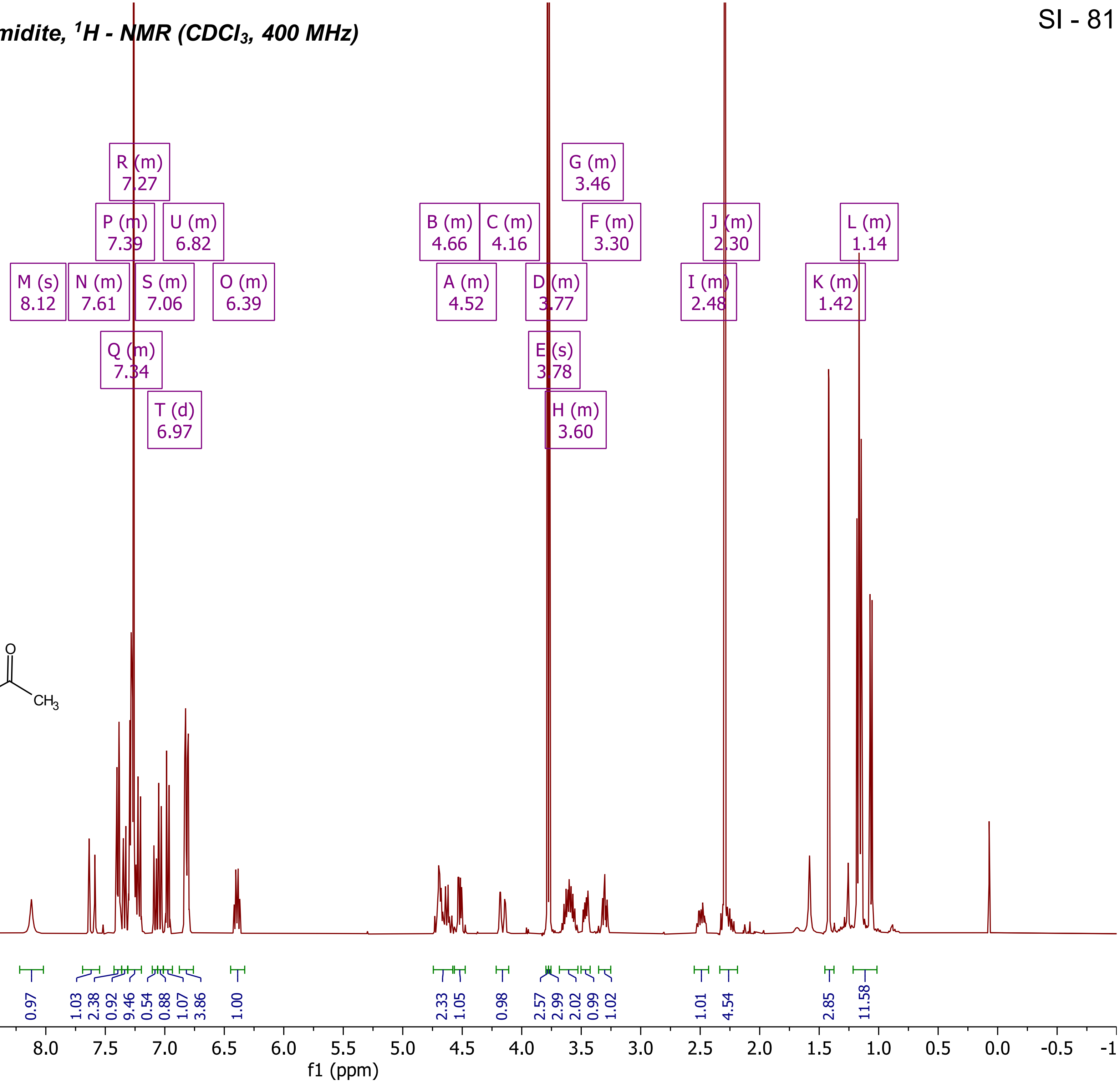

Compound 33: ¹⁸O-DNA-precursor P-amidite, $^{13}C\{^{1}H\}$ - NMR ($CDCl_3$, 101 MHz)

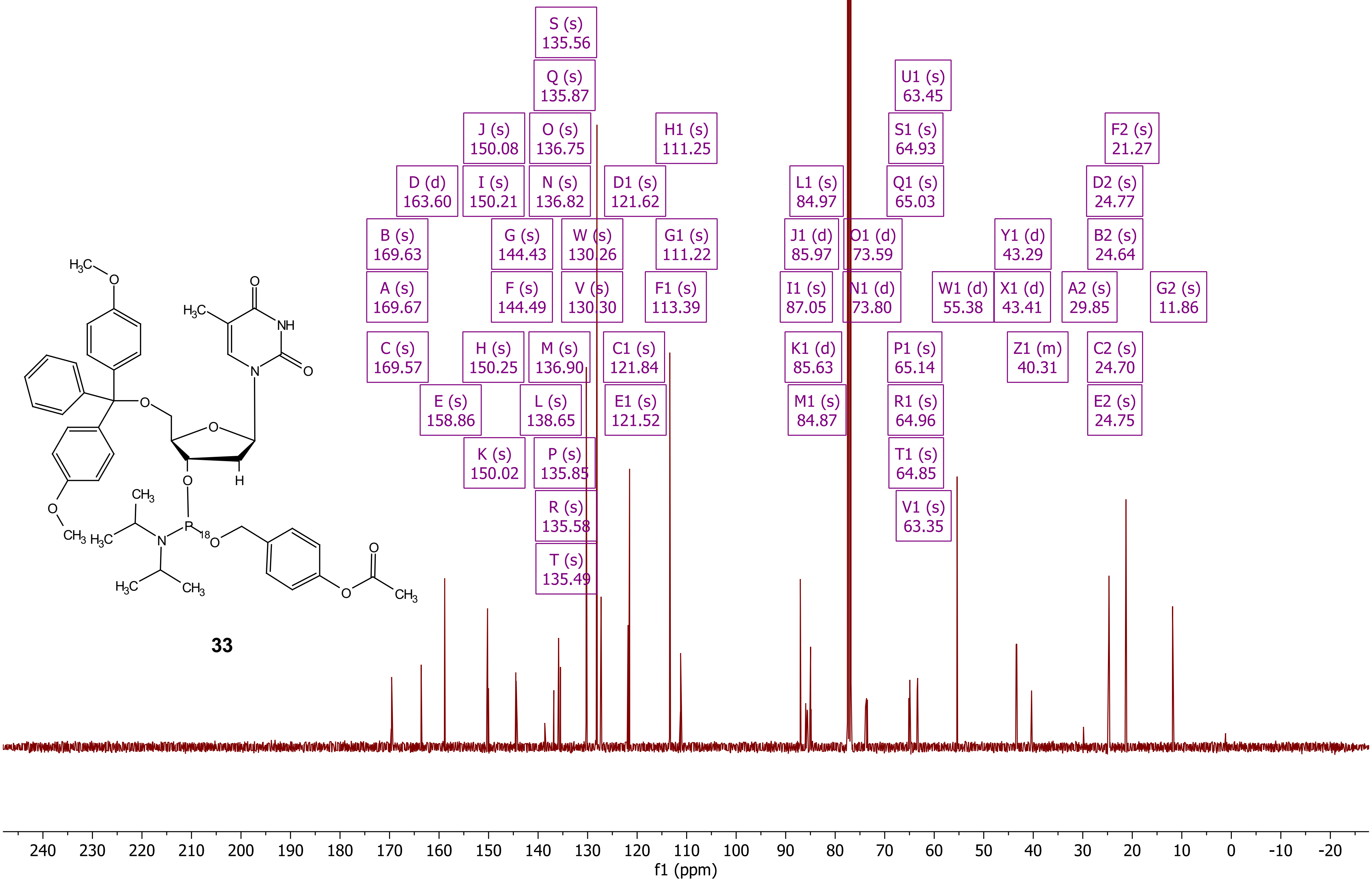

Compound 33: ^{18}O-DNA-precursor P-amidite , ^{31}P{^{1}H} - NMR ($CDCl_3$, 162 MHz)

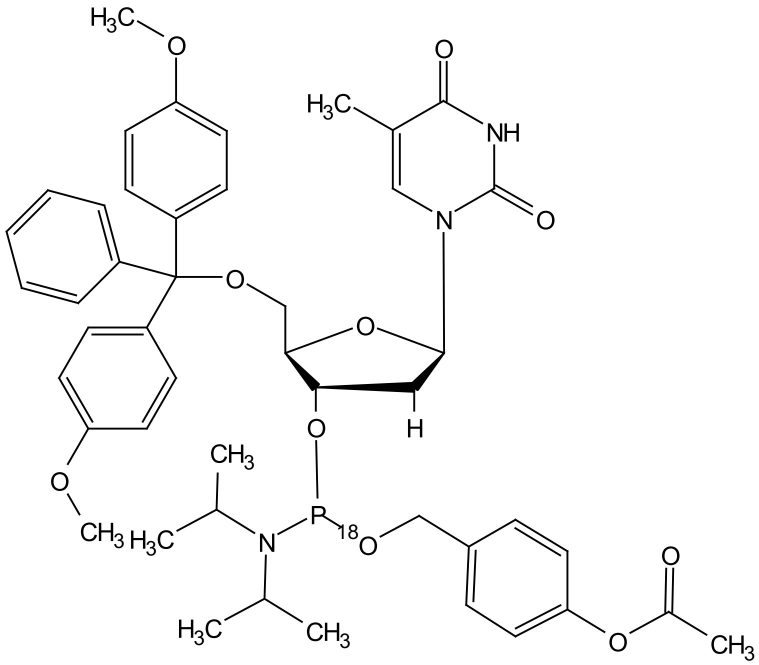

33

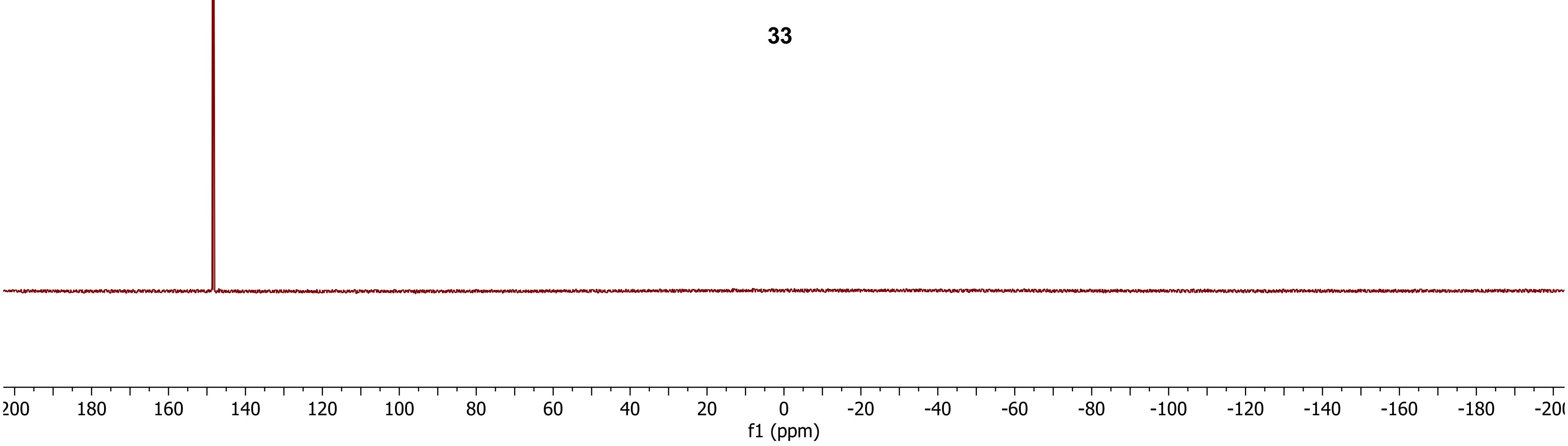

Compound 34: $^{18}O_2$ - AMP, ^1H - NMR (D_2O, 400 MHz)

34

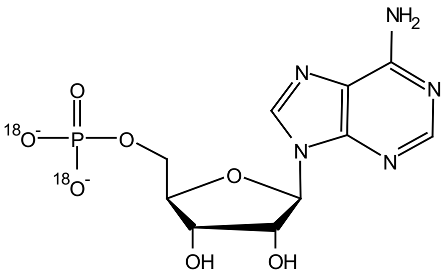

TEA - salt

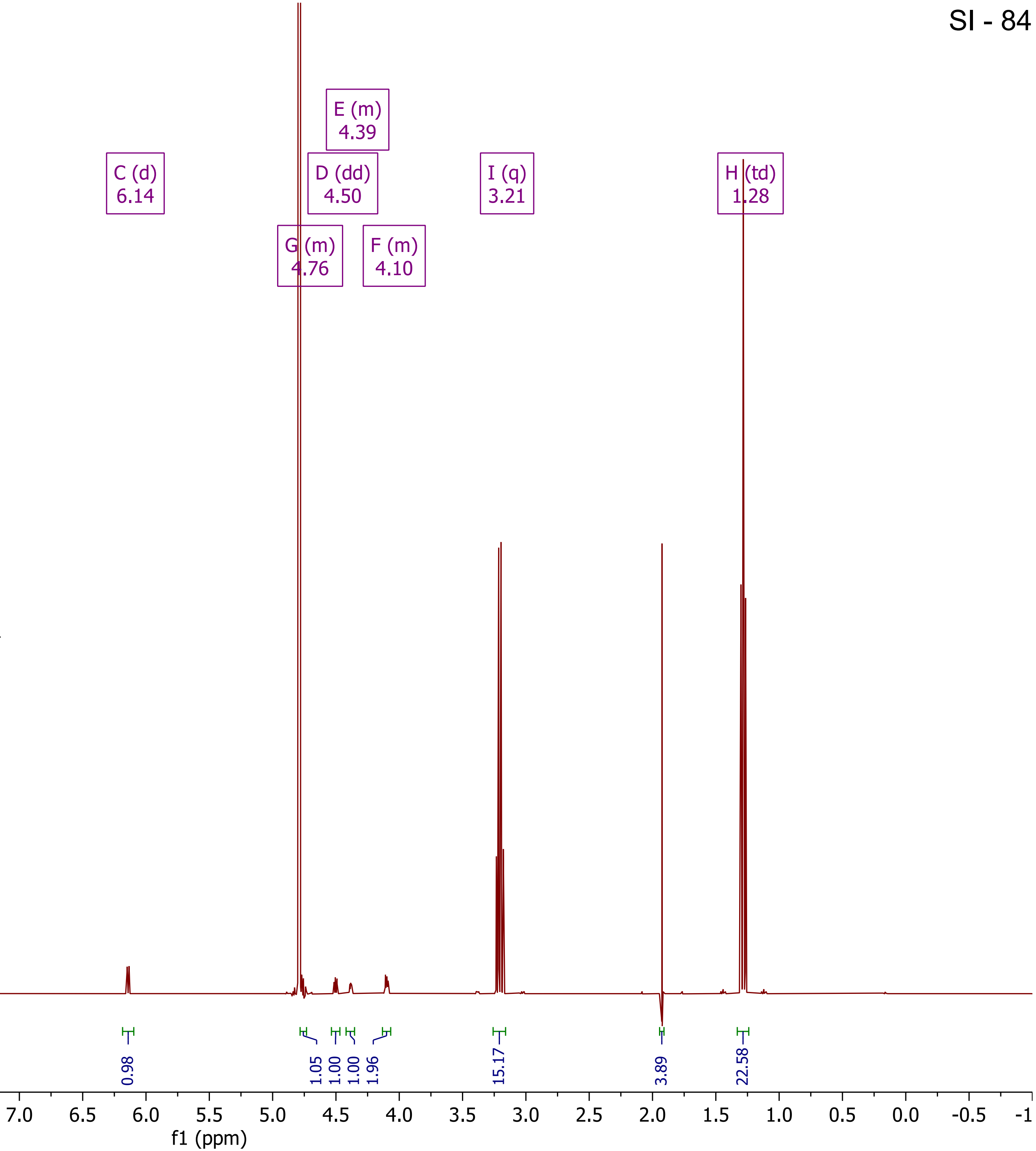

Compound 34: $^{18}O_2$ - AMP, $^{31}P\{^1H\}$ - NMR (D_2O, 162 MHz)

34

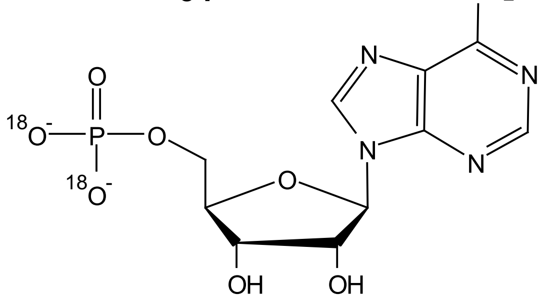

TEA - salt

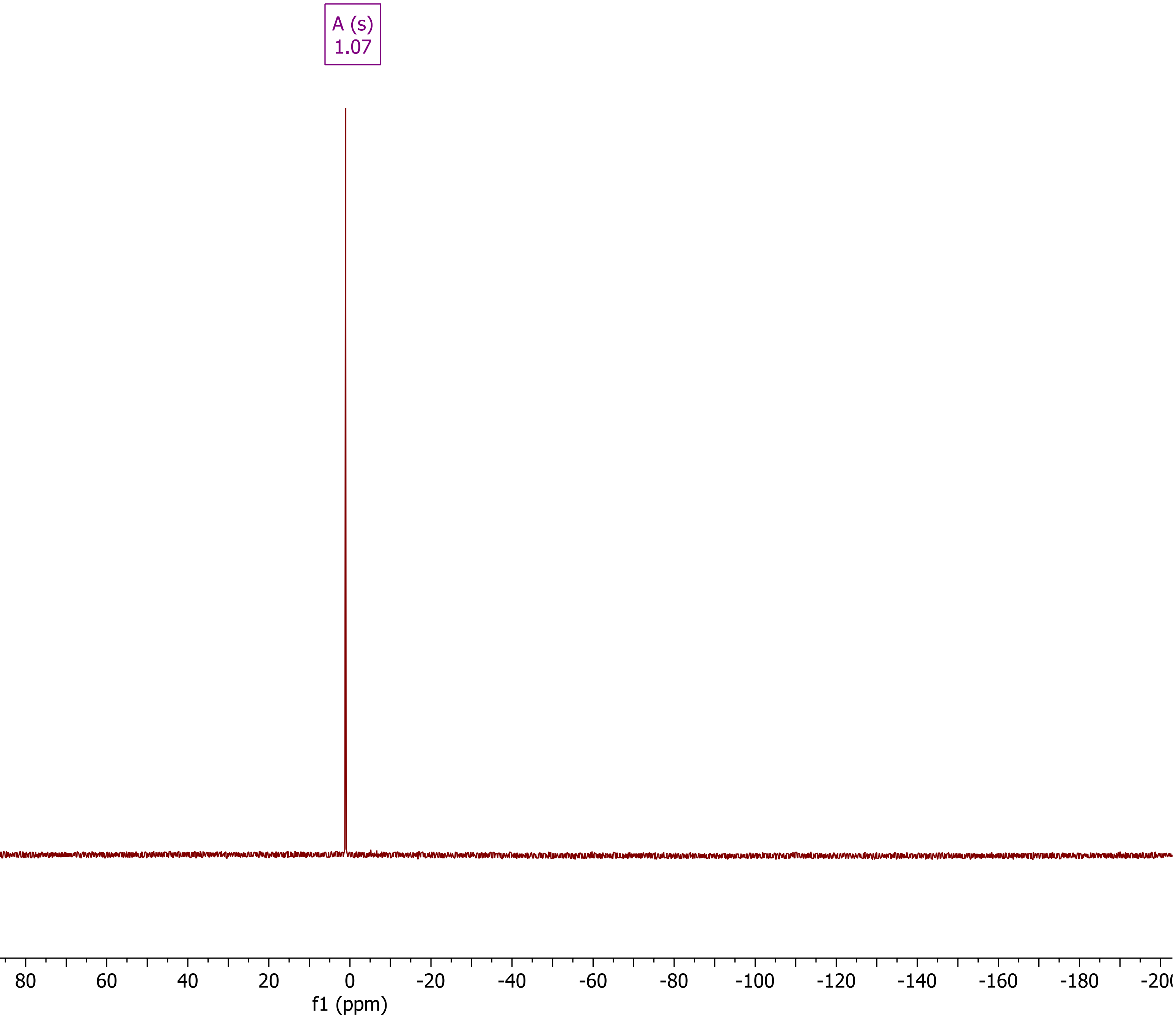

Compound 34: $^{18}O_2$ - AMP, $^{13}C\{^1H\}$ - NMR (D_2O, 101 MHz)

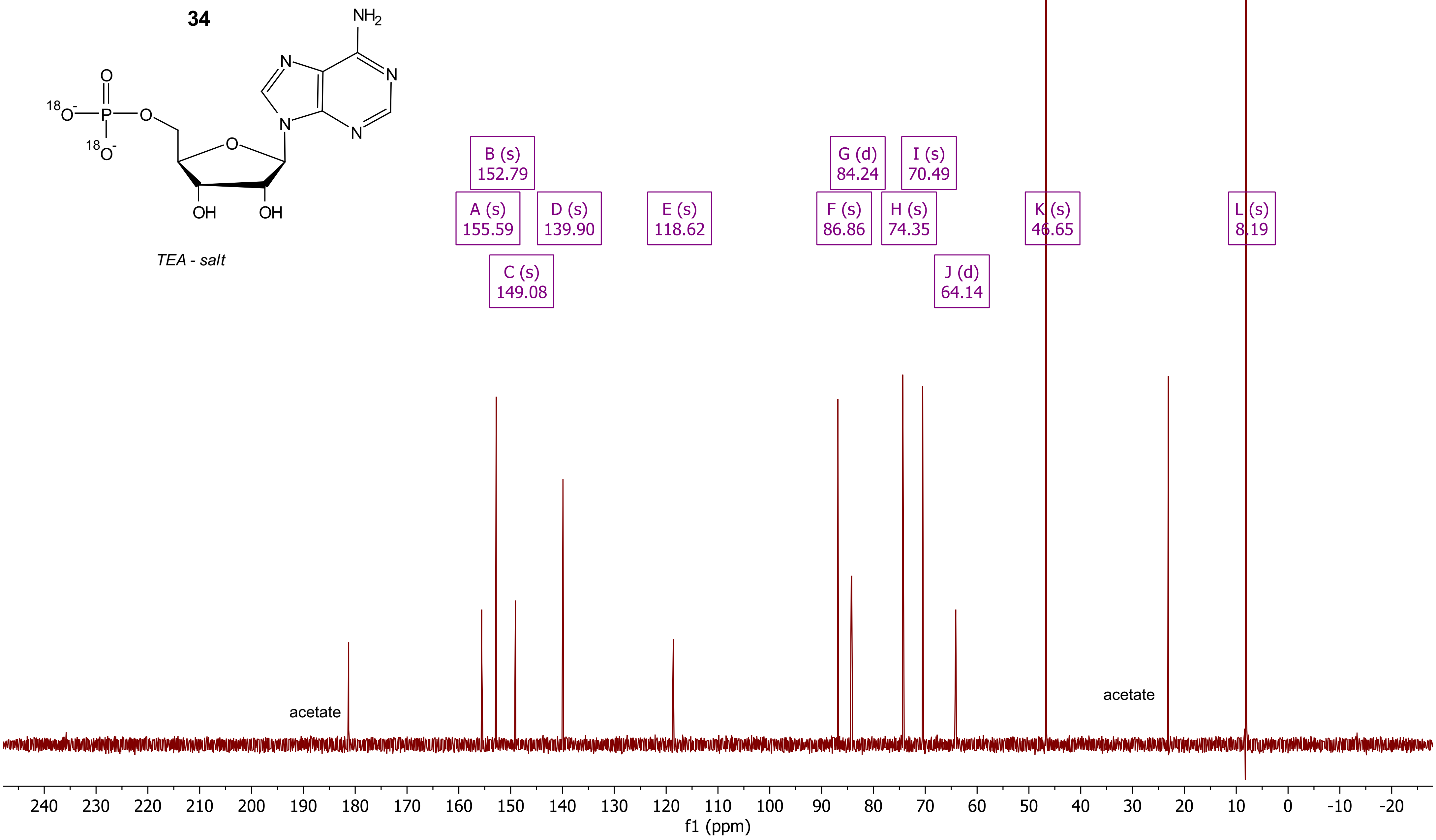

Compound 6: $^{18}O_2$ - GMP, ^1H - NMR (D_2O, 400 MHz)

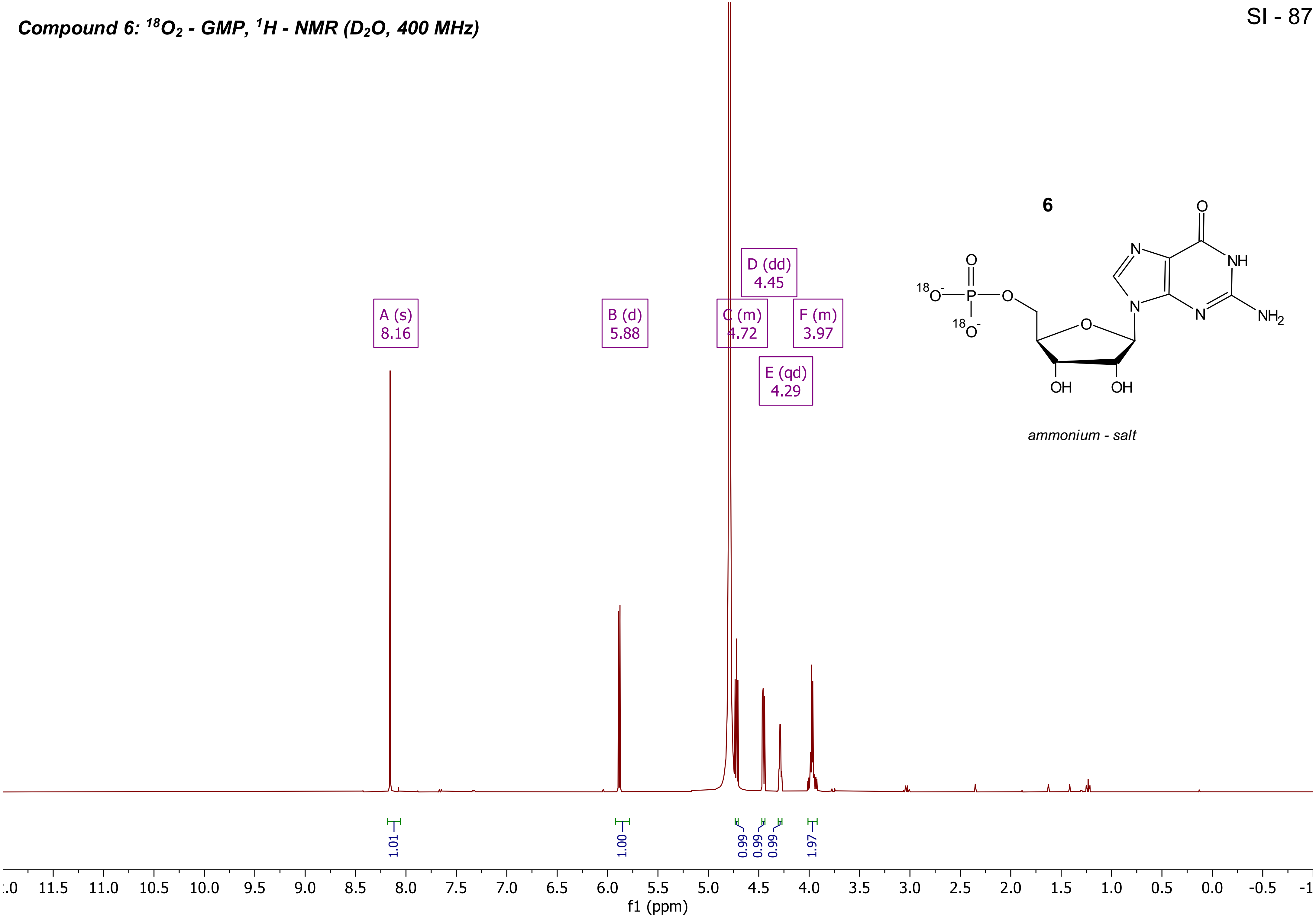

Compound 6: $^{18}O_2$ - GMP, $^{31}P\{^1H\}$ - NMR (D_2O, 162 MHz)

6

ammonium - salt

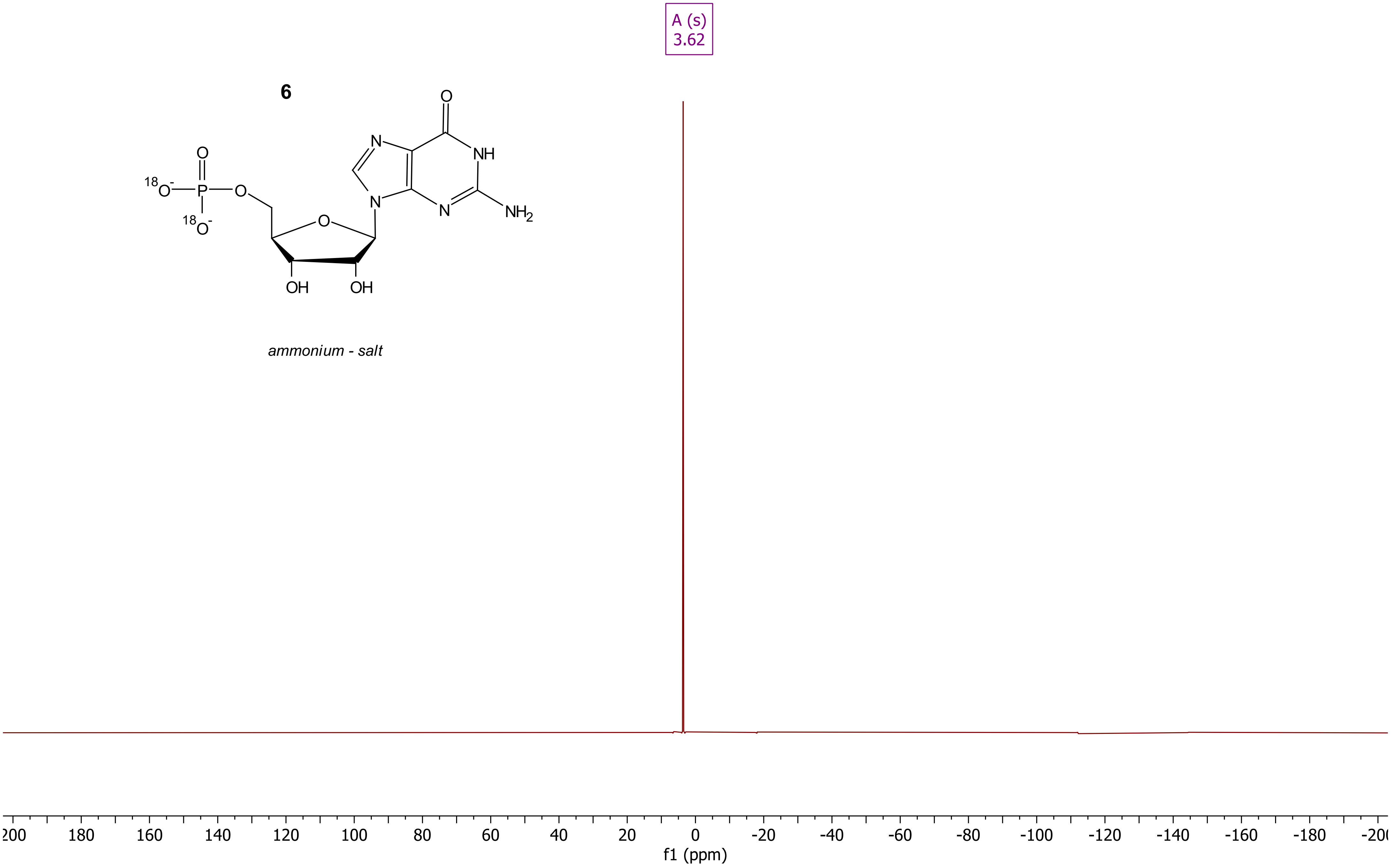

Compound 6: $^{18}O_2$ - GMP, $^{13}C\{^{1}H\}$ - NMR (D_2O, 101 MHz)

6

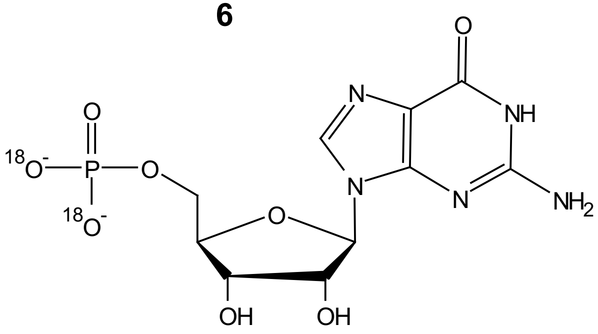

ammonium - salt

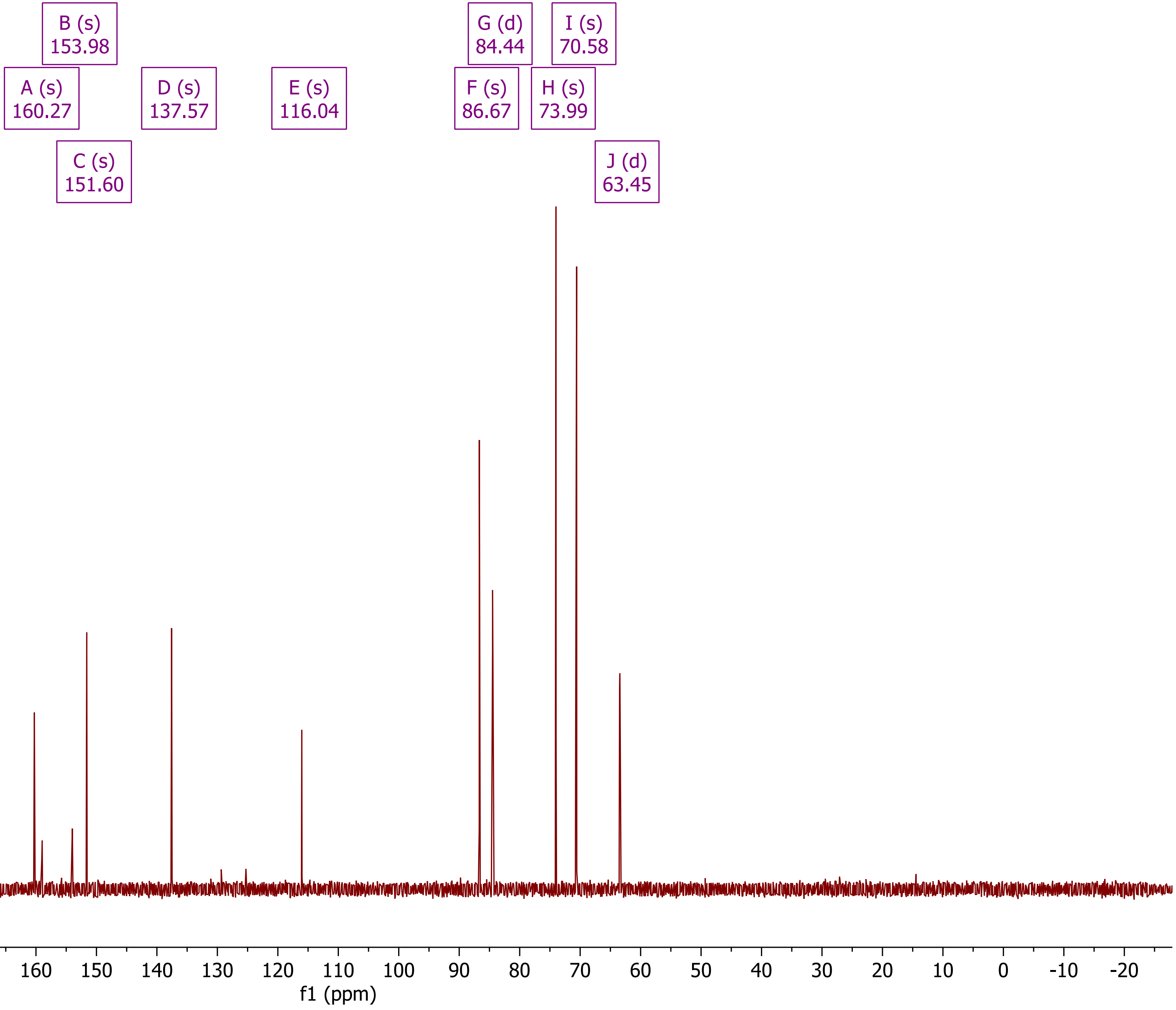

Compound 35: $^{18}O_2$ - ADP, ^{1}H - NMR (D_2O, 400 MHz)

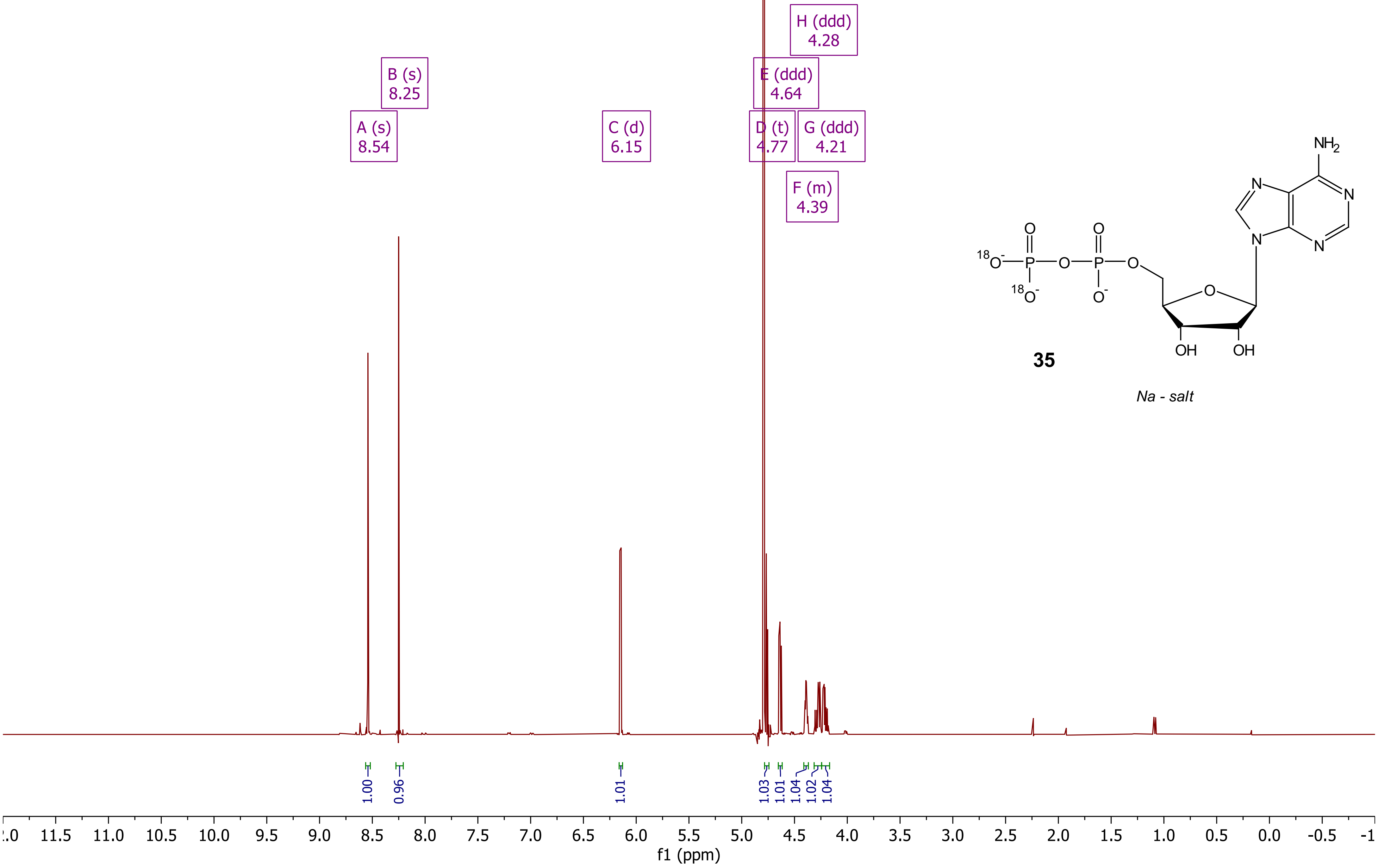

Compound 35: $^{18}O_2$ - ADP, $^{31}P\{^1H\}$ - NMR (D_2O, 162 MHz)

35

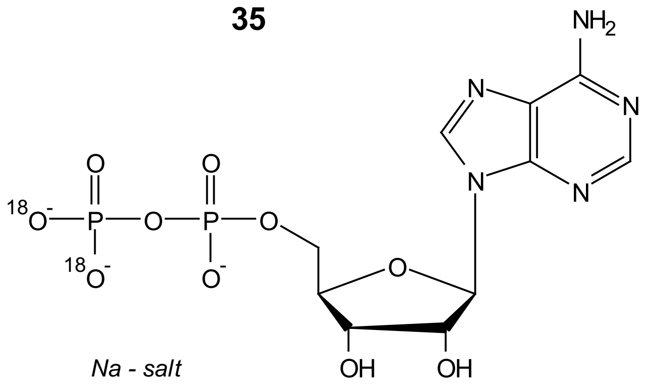

Na - salt

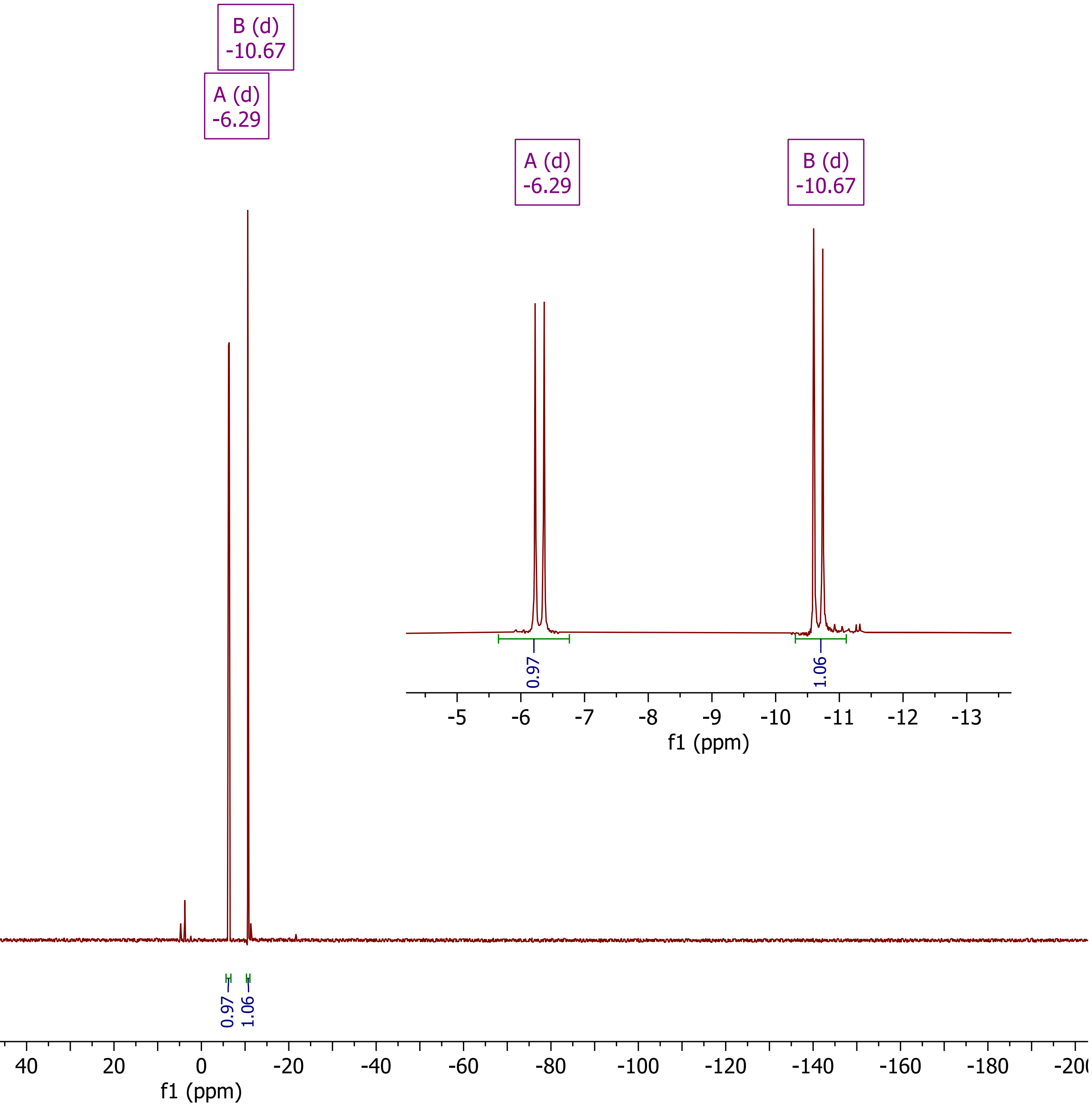

Compound 35: $^{18}O_2$ - ADP, $^{13}C\{^1H\}$ - NMR (D_2O, 101 MHz)

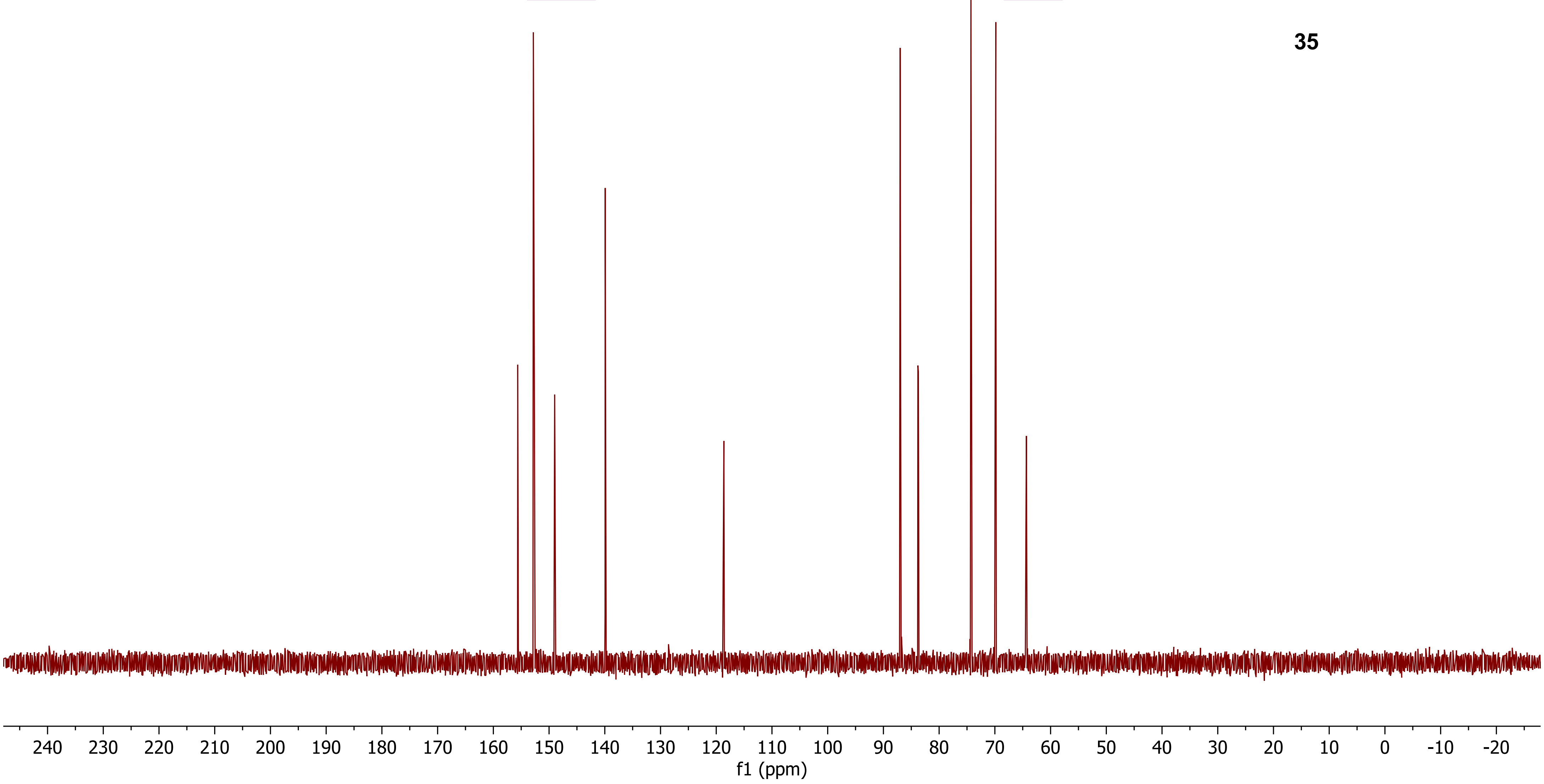

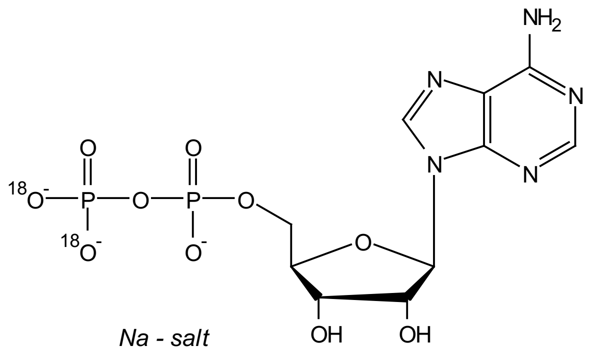

35

Compound 36: $^{18}O_2$ - ATP, ^{1}H - NMR (D_2O, 400 MHz)

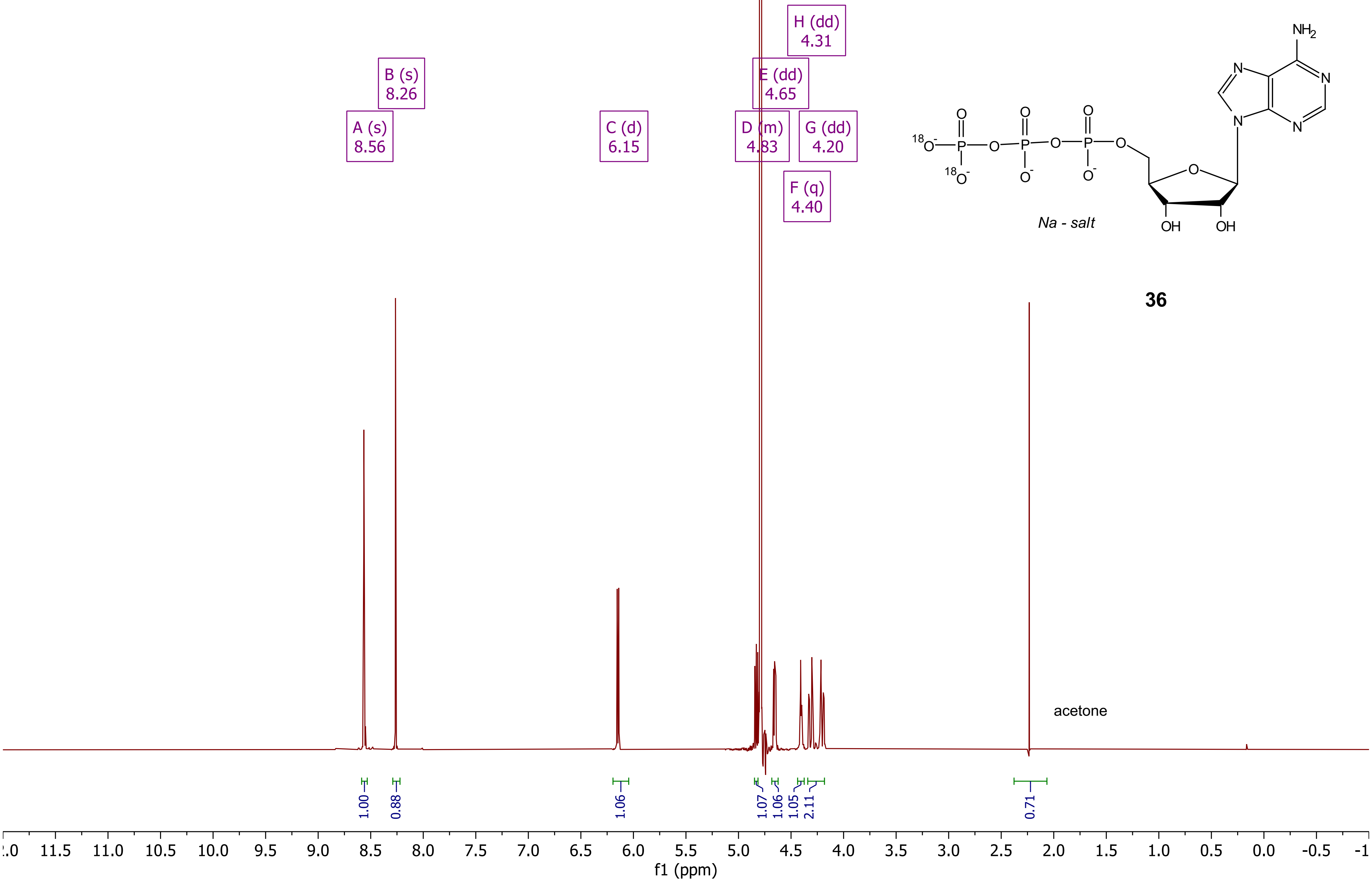

Compound 36: $^{18}O_2$ - ATP, $^{31}P\{^1H\}$ - NMR (D_2O, 162 MHz)

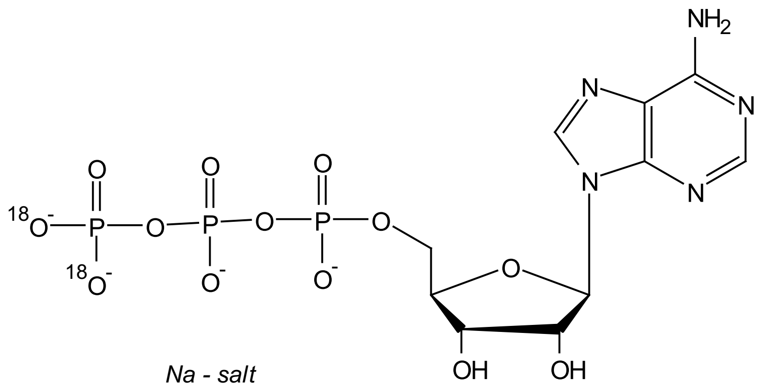

Na - salt

36

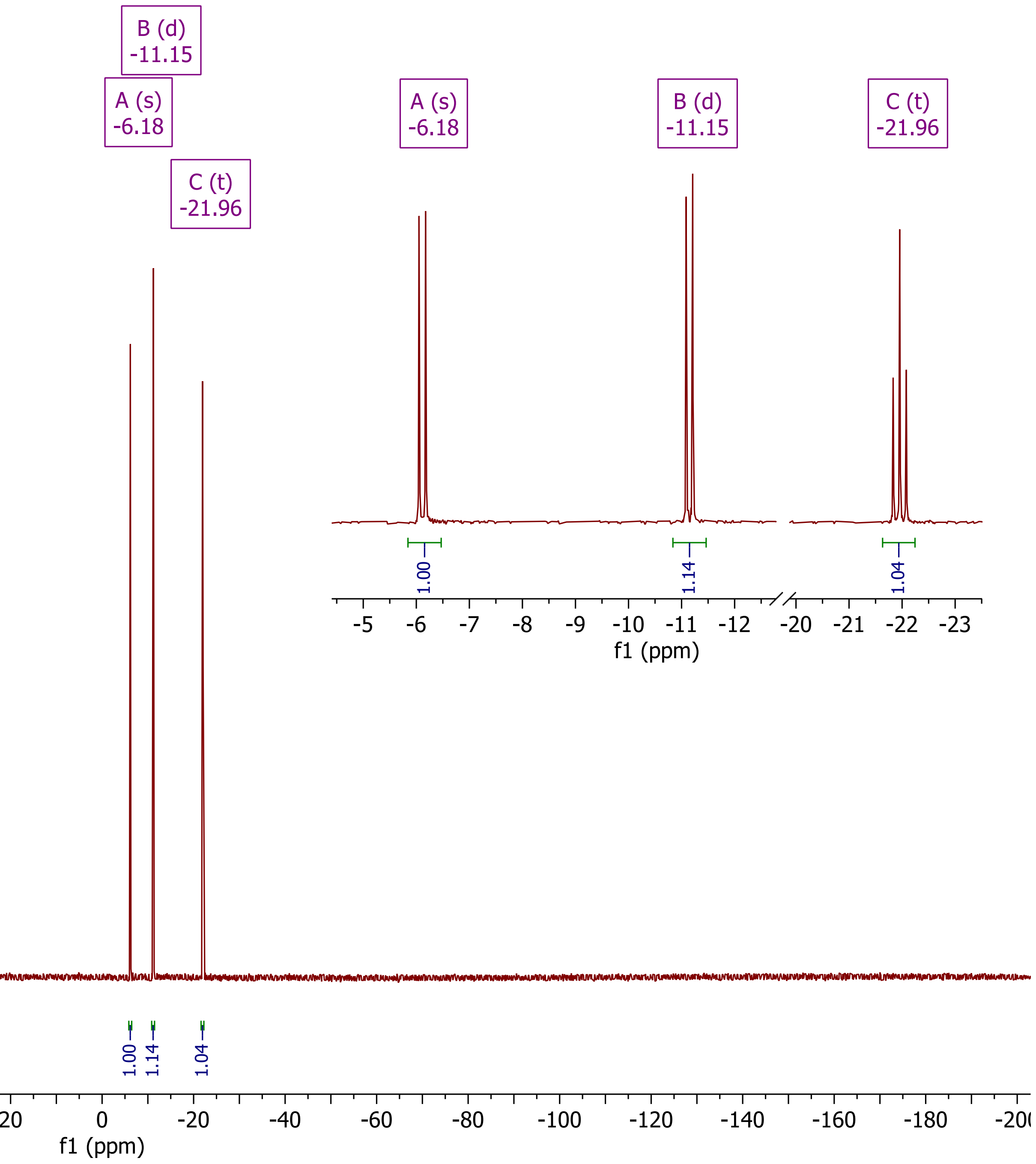

Compound 36: $^{18}O_2$ - ATP, $^{13}C\{^1H\}$ - NMR (D_2O, 101 MHz))

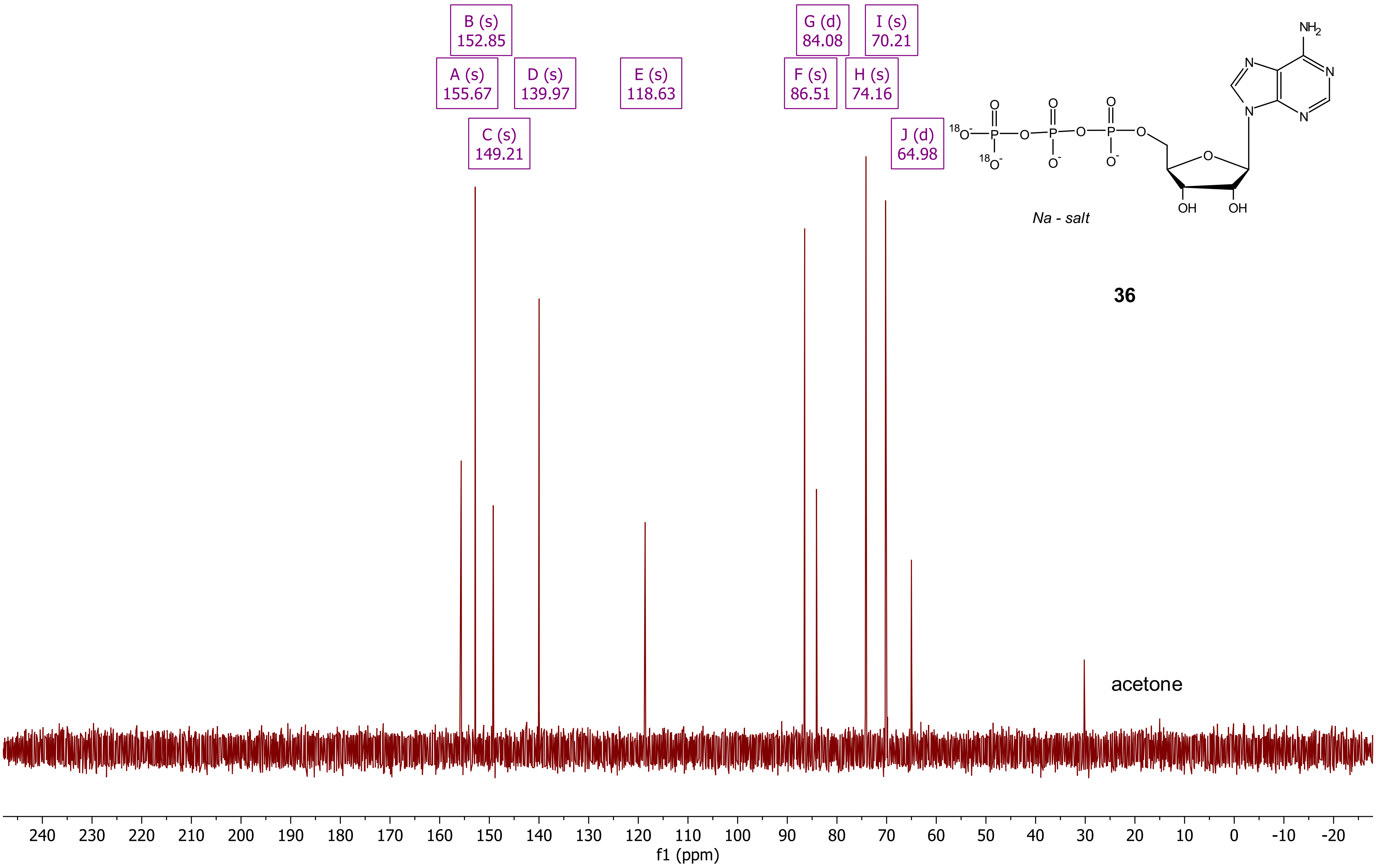

36

Compound 37: $^{18}O_2$ - GTP, ^1H - NMR (D_2O, 400 MHz)

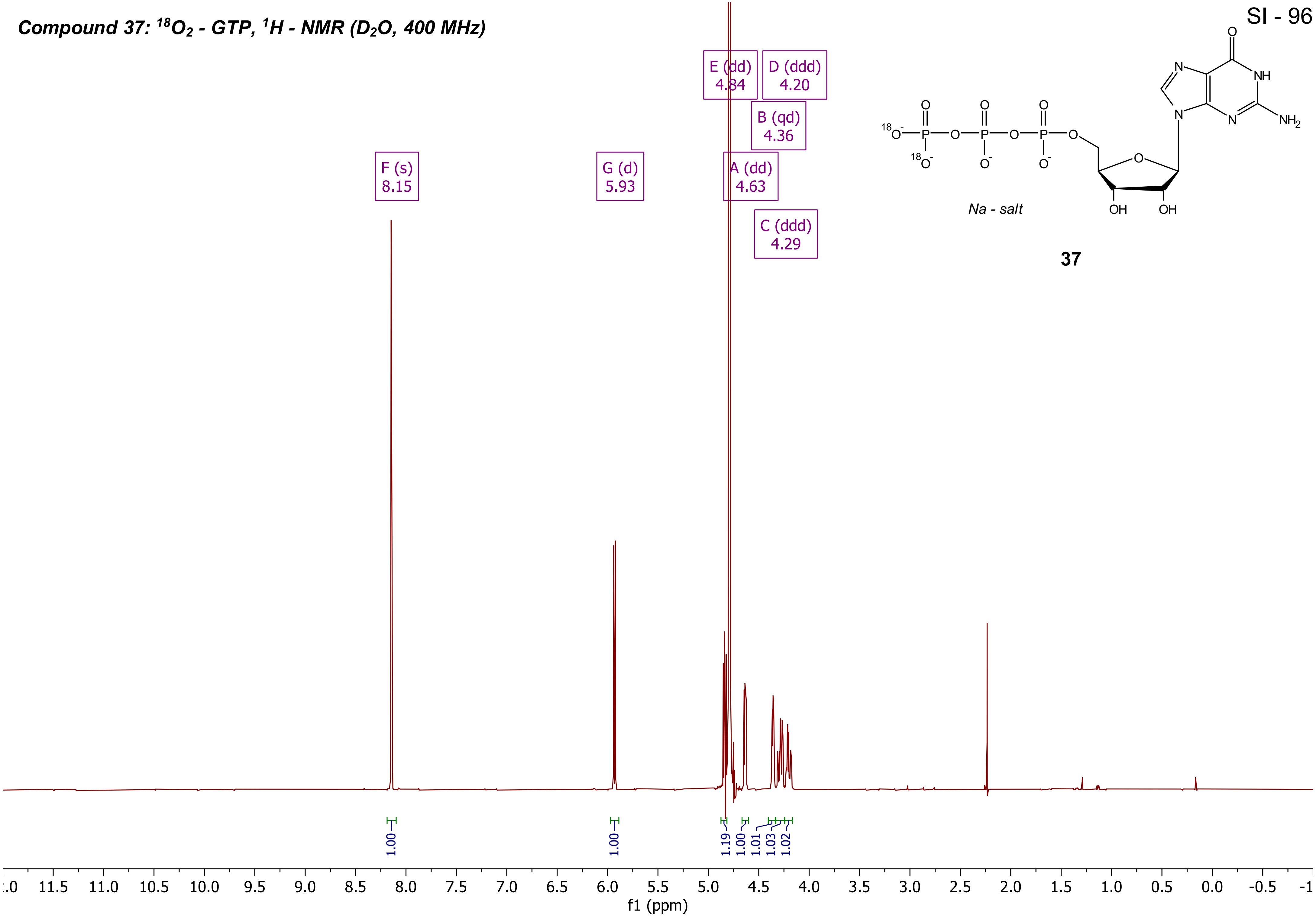

Compound 37: $^{18}O_2$ - GTP, $^{31}P\{^1H\}$ - NMR (D_2O, 162 MHz)

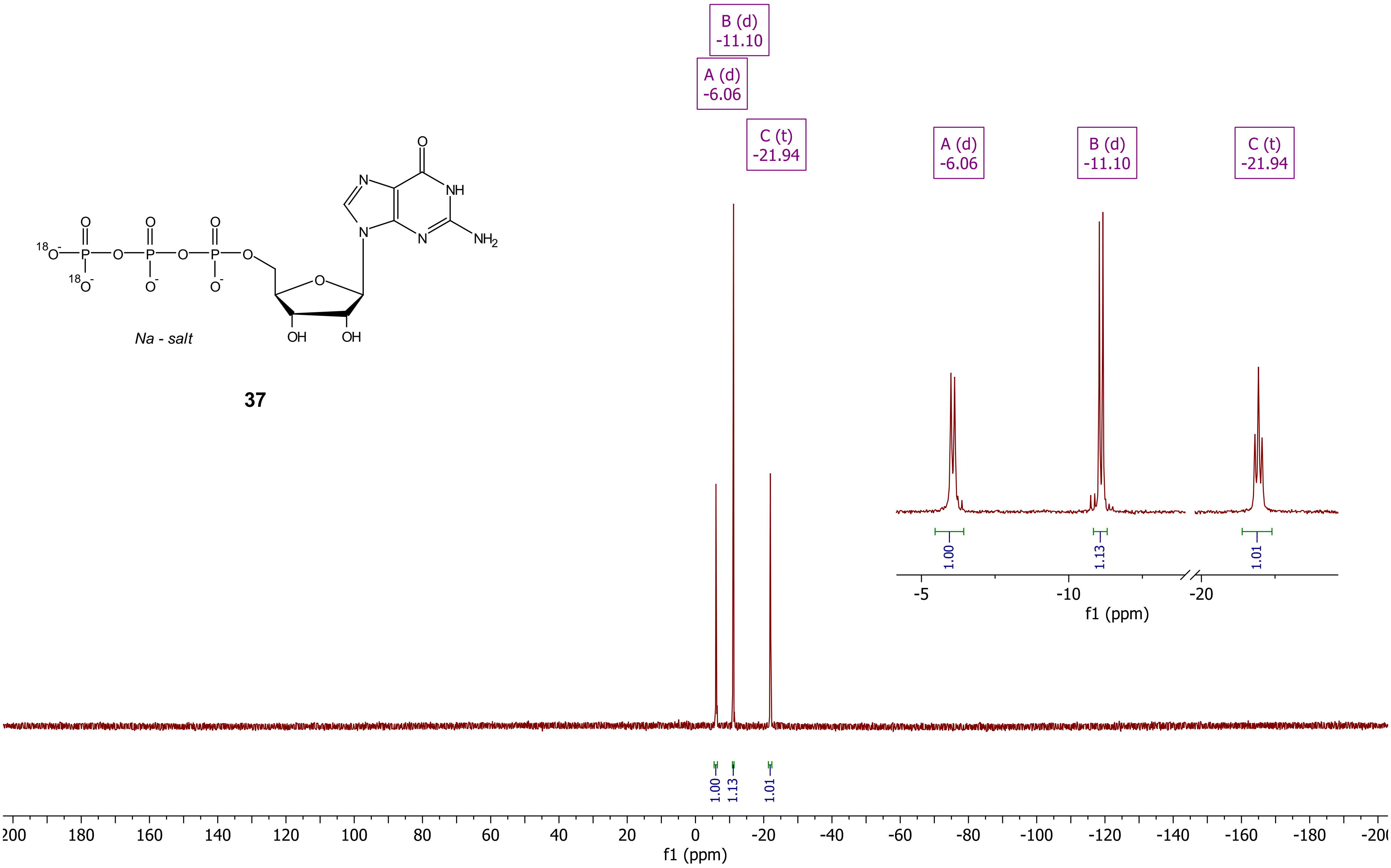

37

Compound 37: $^{18}O_2$ - GTP, $^{13}C\{^1H\}$ - NMR (D_2O, 101 MHz)

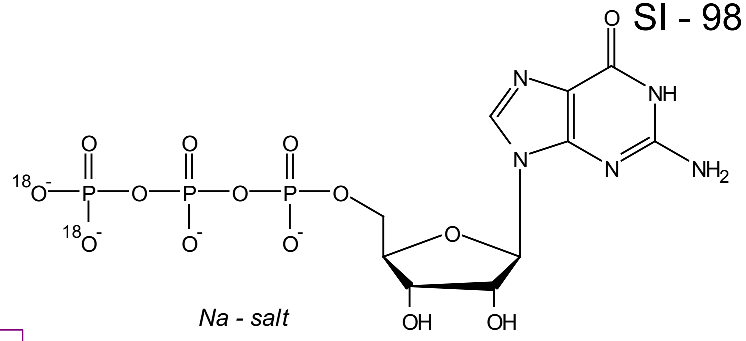

37

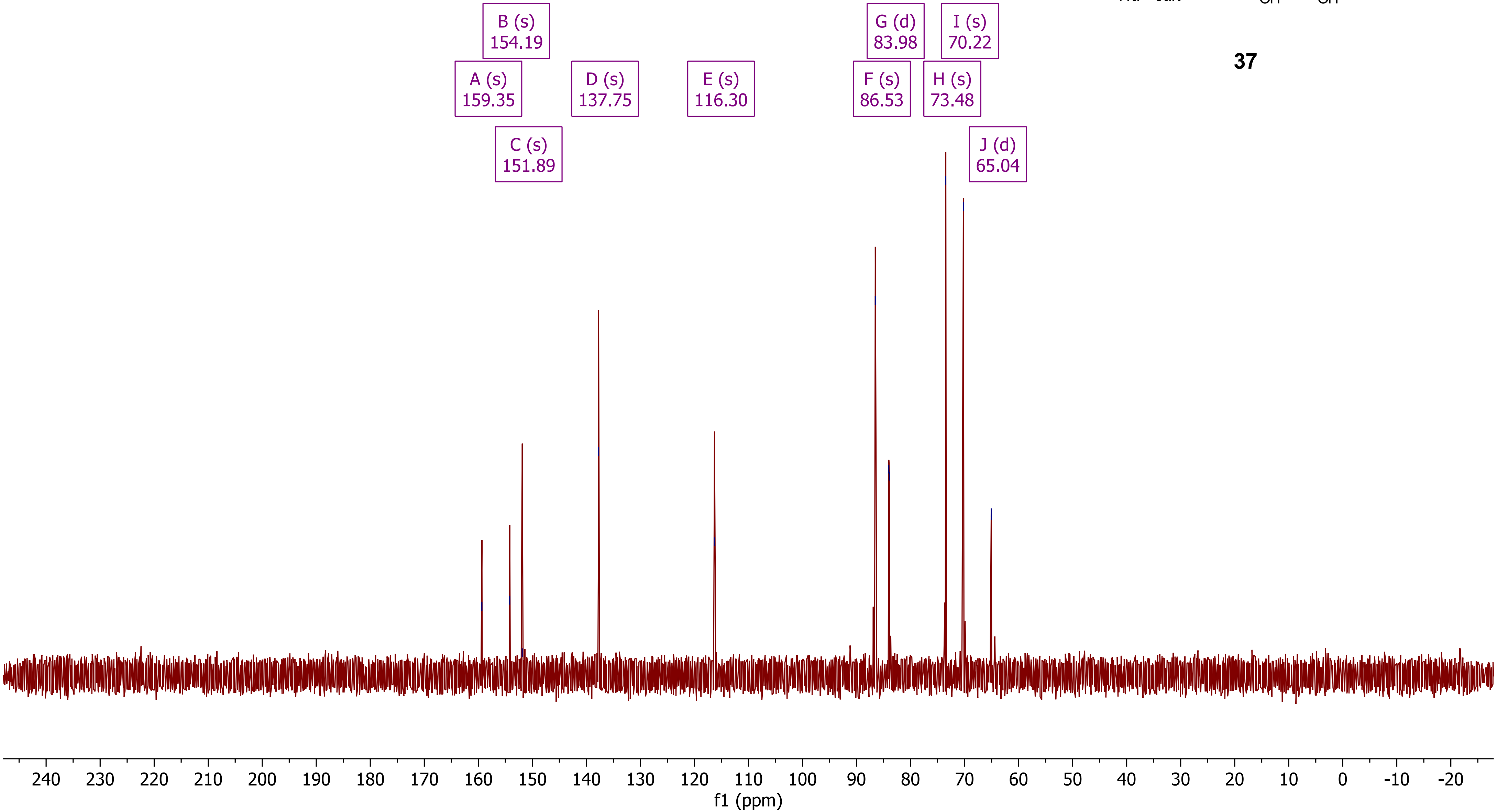

Compound 38: $^{18}O_2$ - UTP, ^{1}H - NMR (D_2O, 400 MHz)

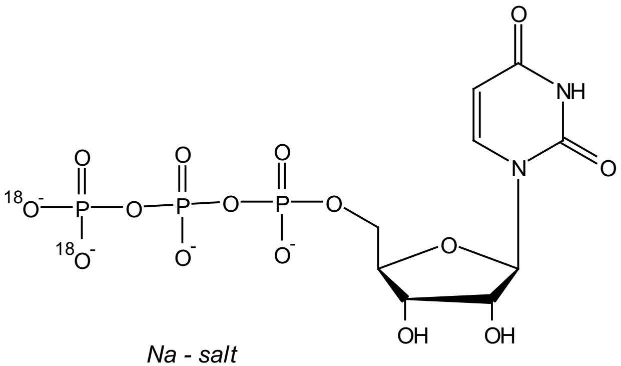

Na - salt

38

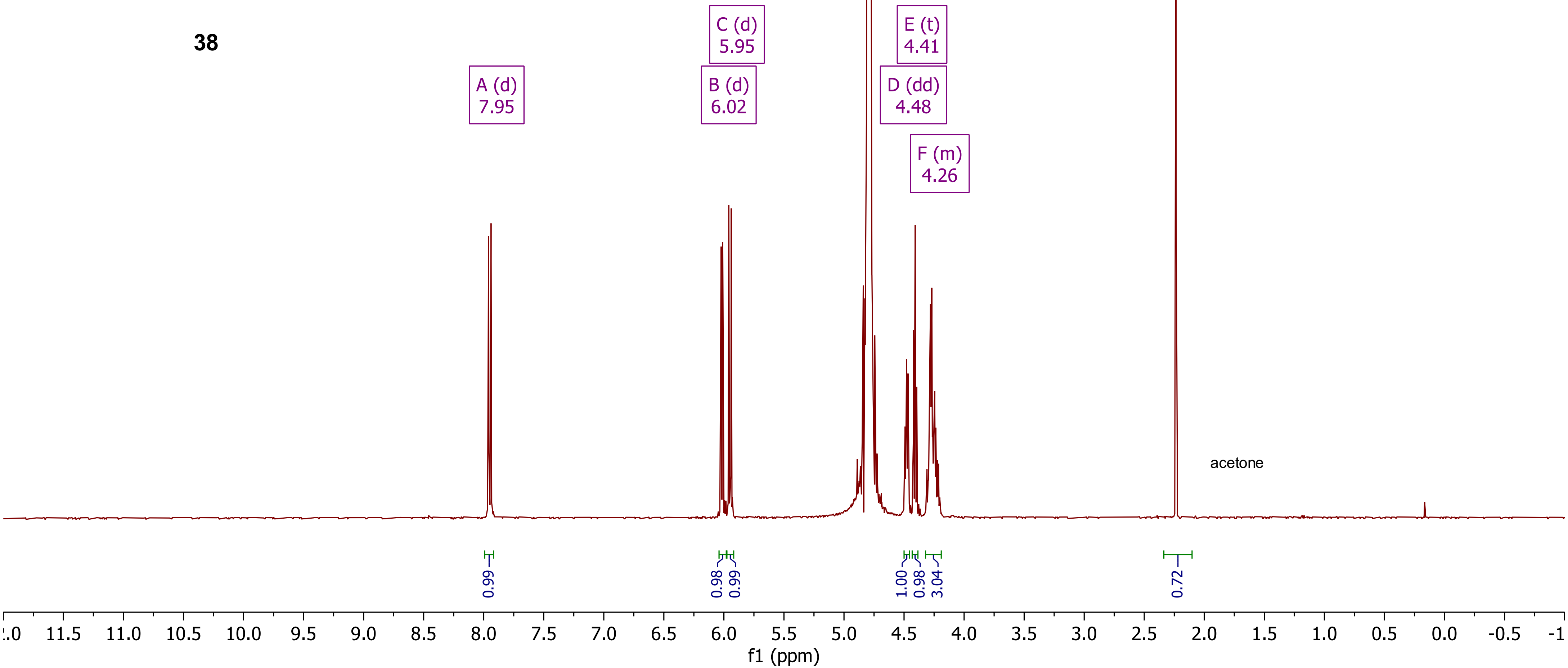

Compound 38: $^{18}O_2$ - UTP, $^{31}P\{^1H\}$ - NMR (D_2O, 162 MHz)

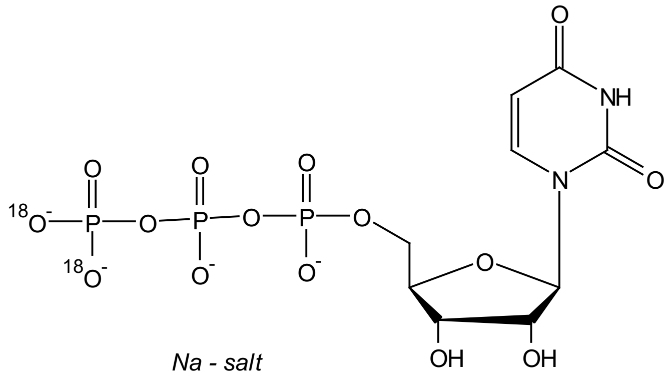

38

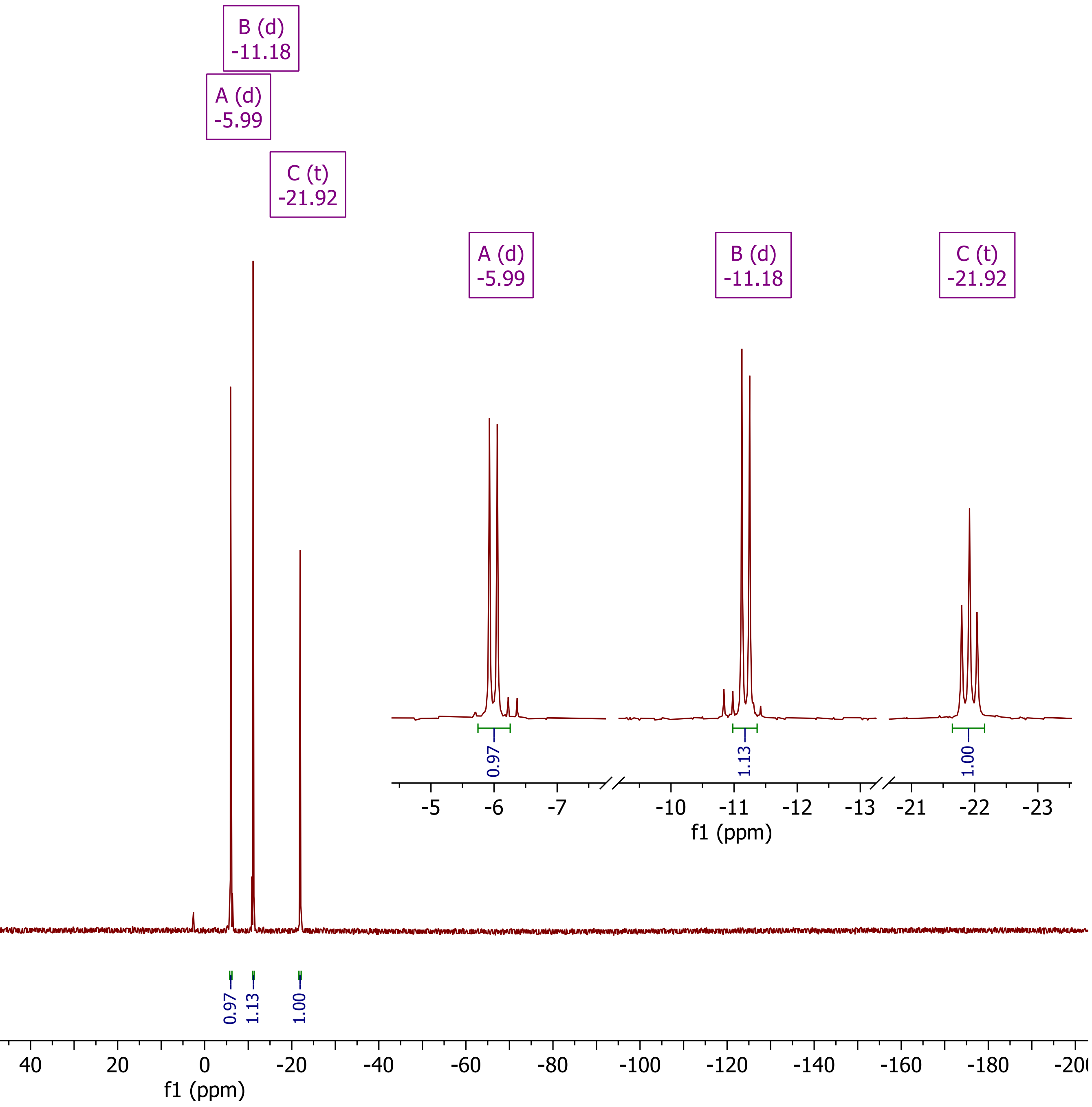

Compound 38: $^{18}O_2$ - UTP, $^{13}C\{^1H\}$ - NMR (D_2O, 101 MHz)

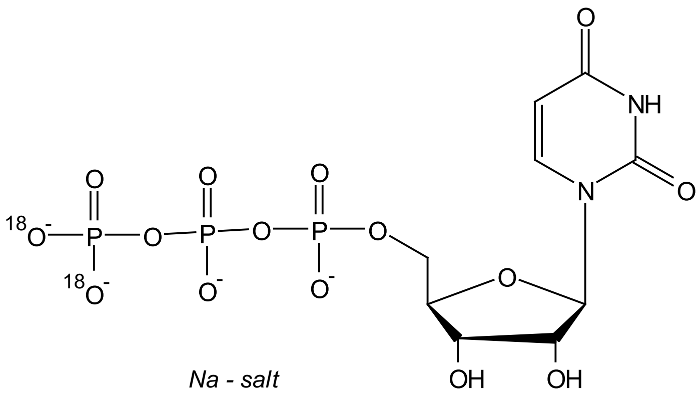

Na - salt

38

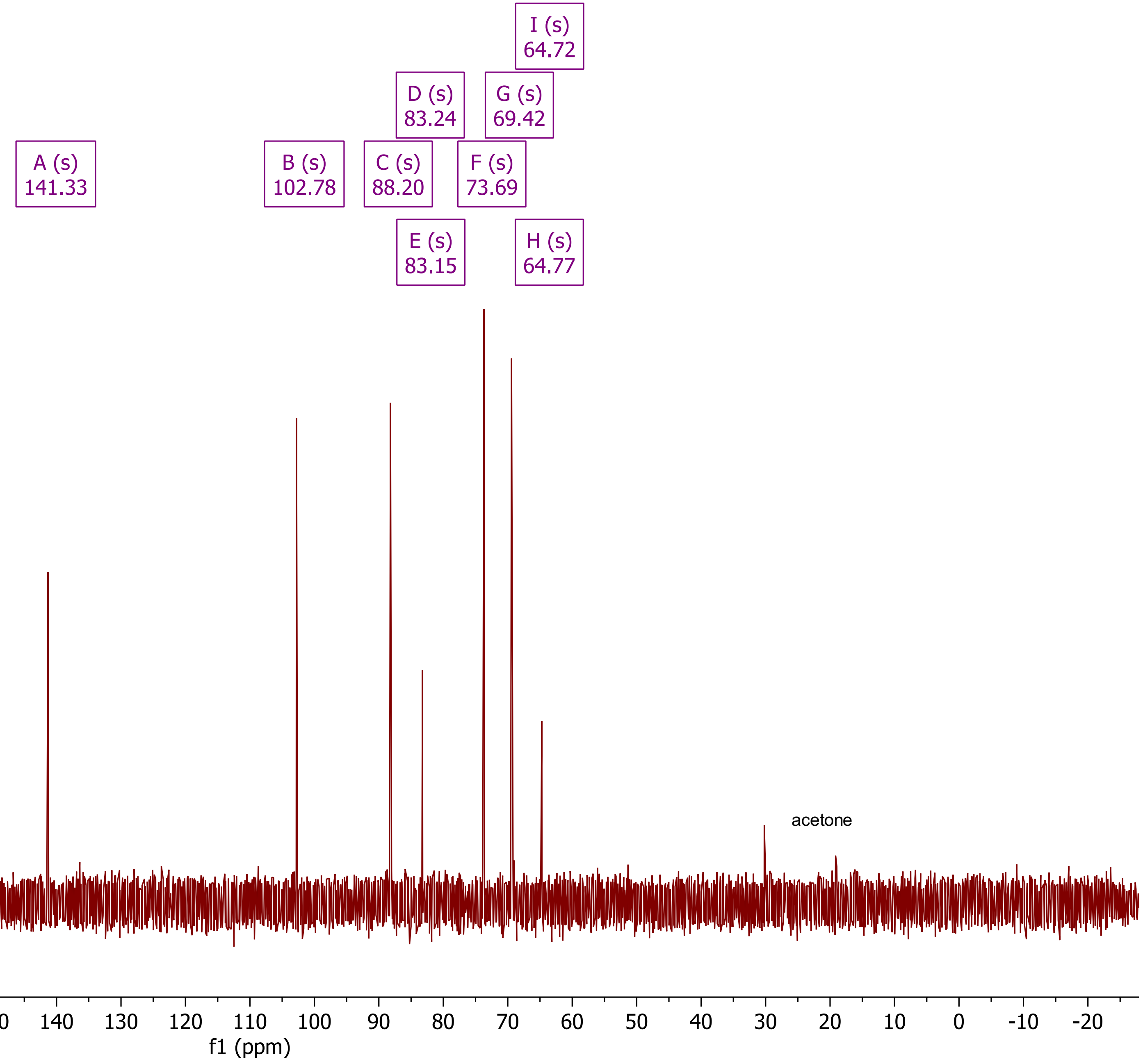

Compound 39: $^{18}O_2$ - A4P, ^{1}H - NMR (D_2O, 400 MHz)

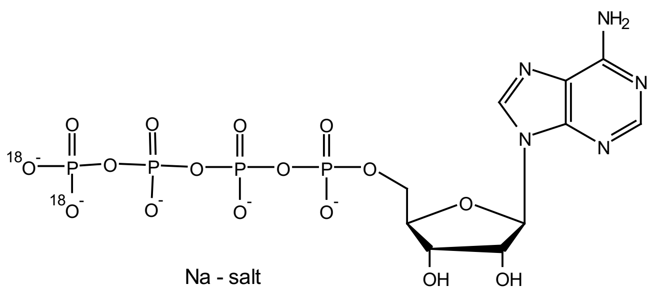

39

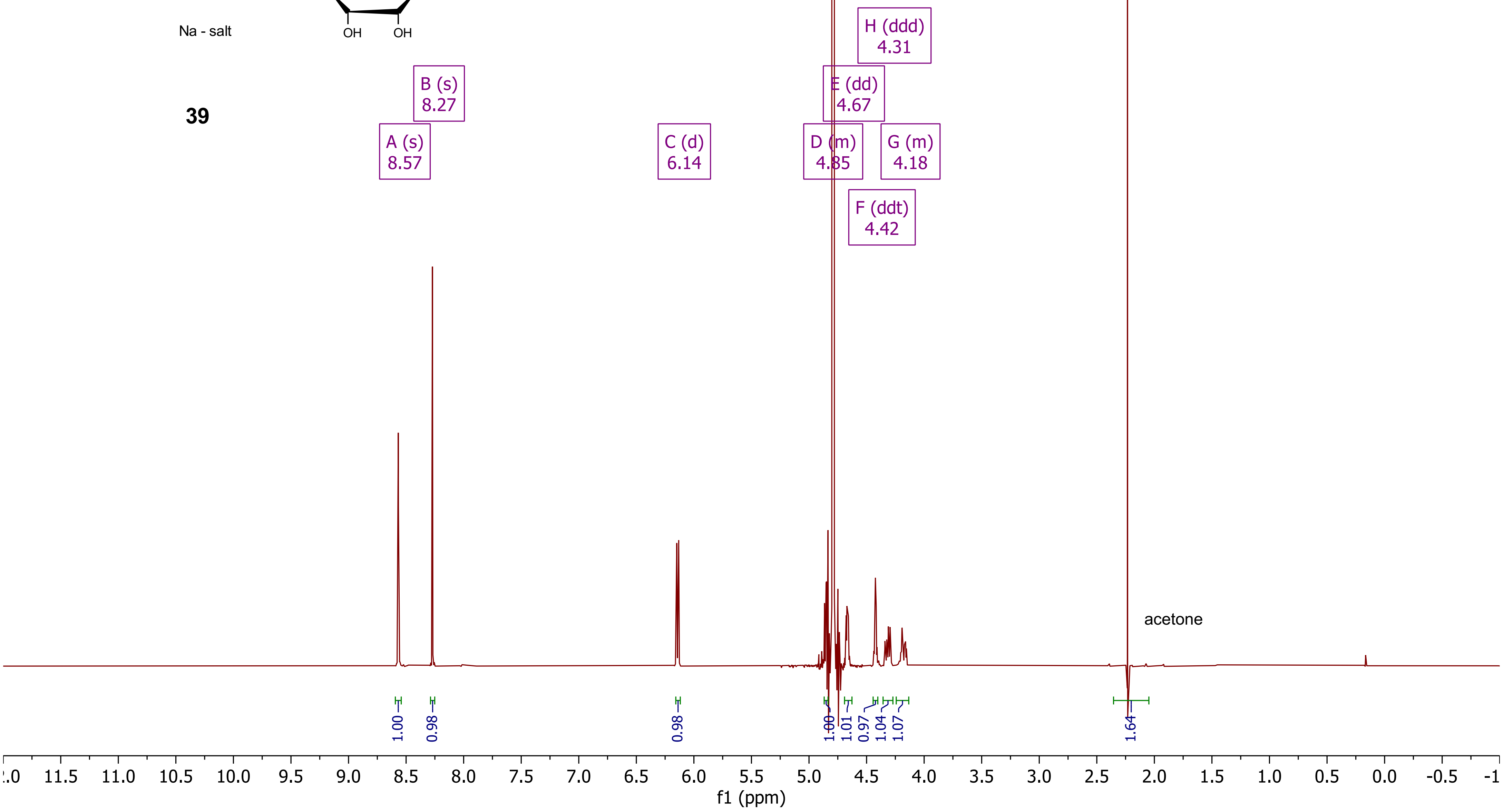

Compound 39: $^{18}O_2$ - A4P, $^{31}P\{^1H\}$ - NMR (D_2O, 162 MHz)

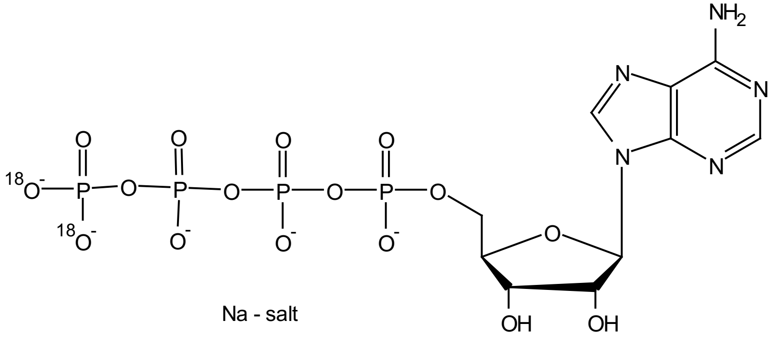

39

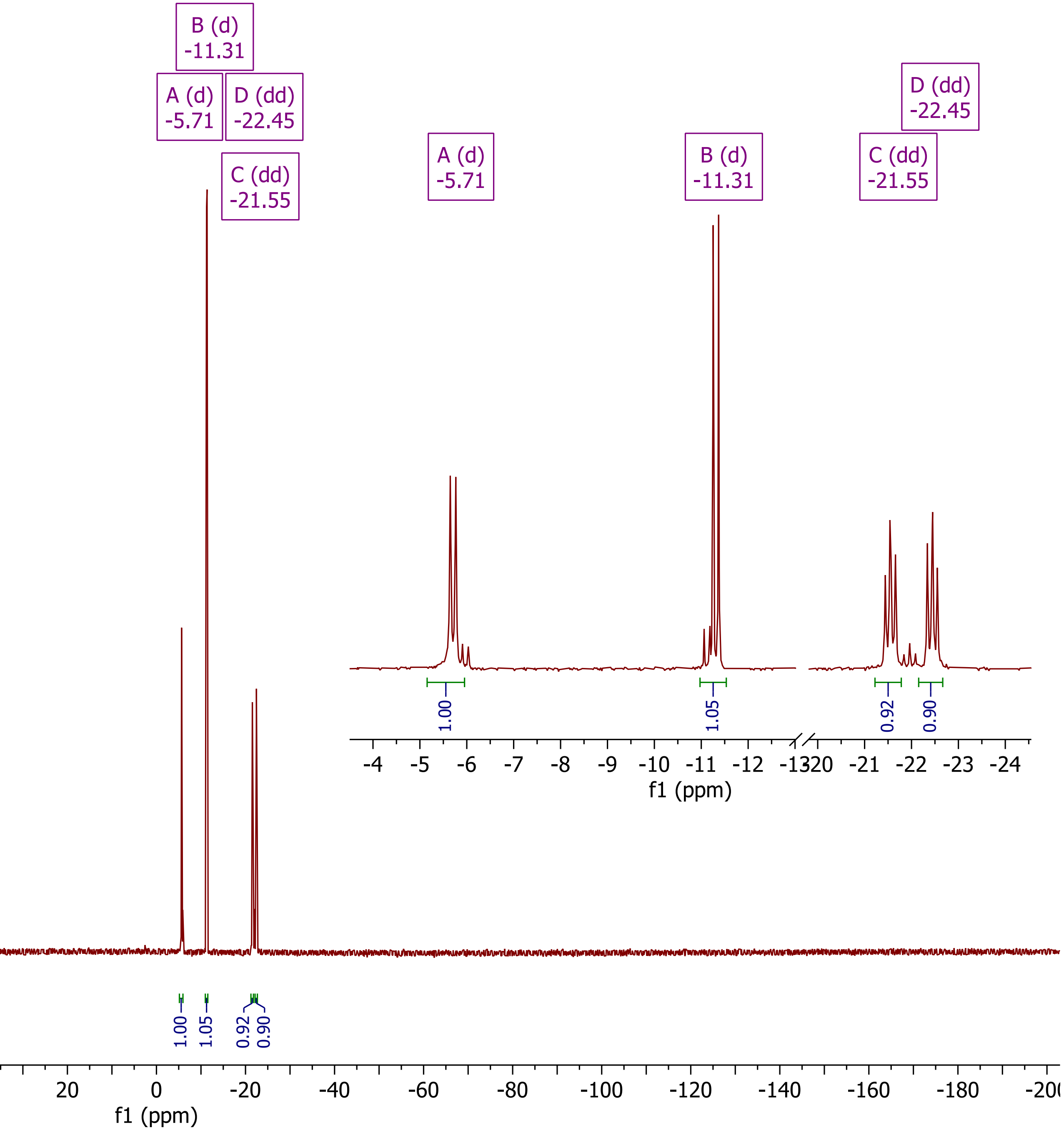

Compound 39: $^{18}O_2$ - A4P, $^{13}C\{^1H\}$ - NMR (D_2O, 101 MHz)

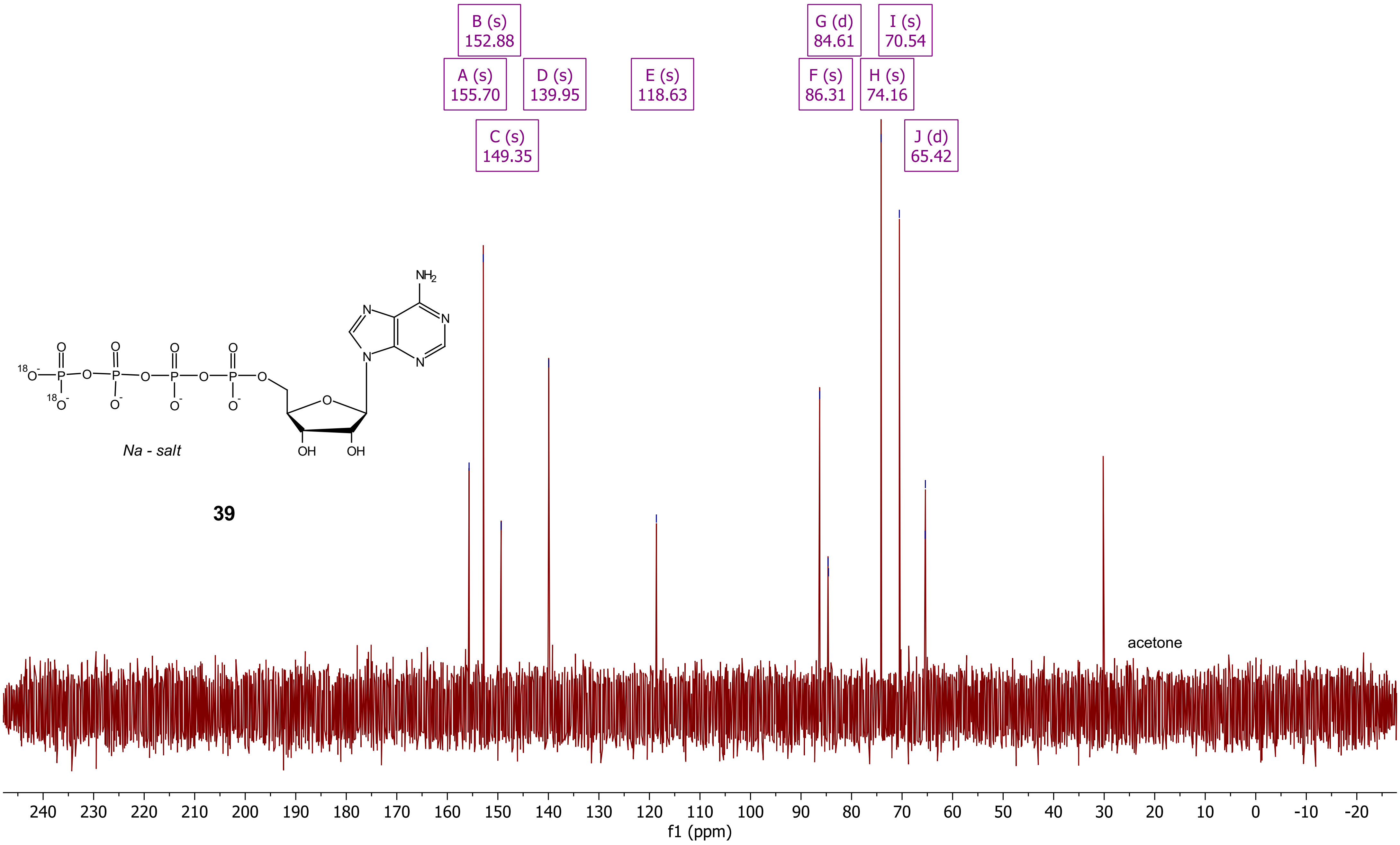

Compound 40:* ^{18}O-Ap3A, ^{1}*H - NMR (D$_2$O, 400 MHz)

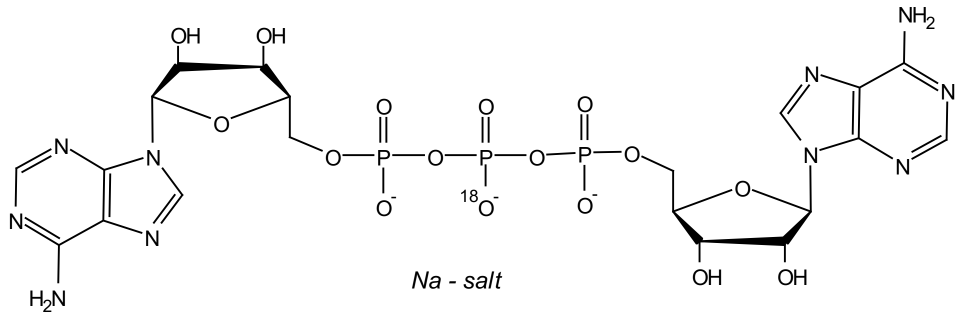

40

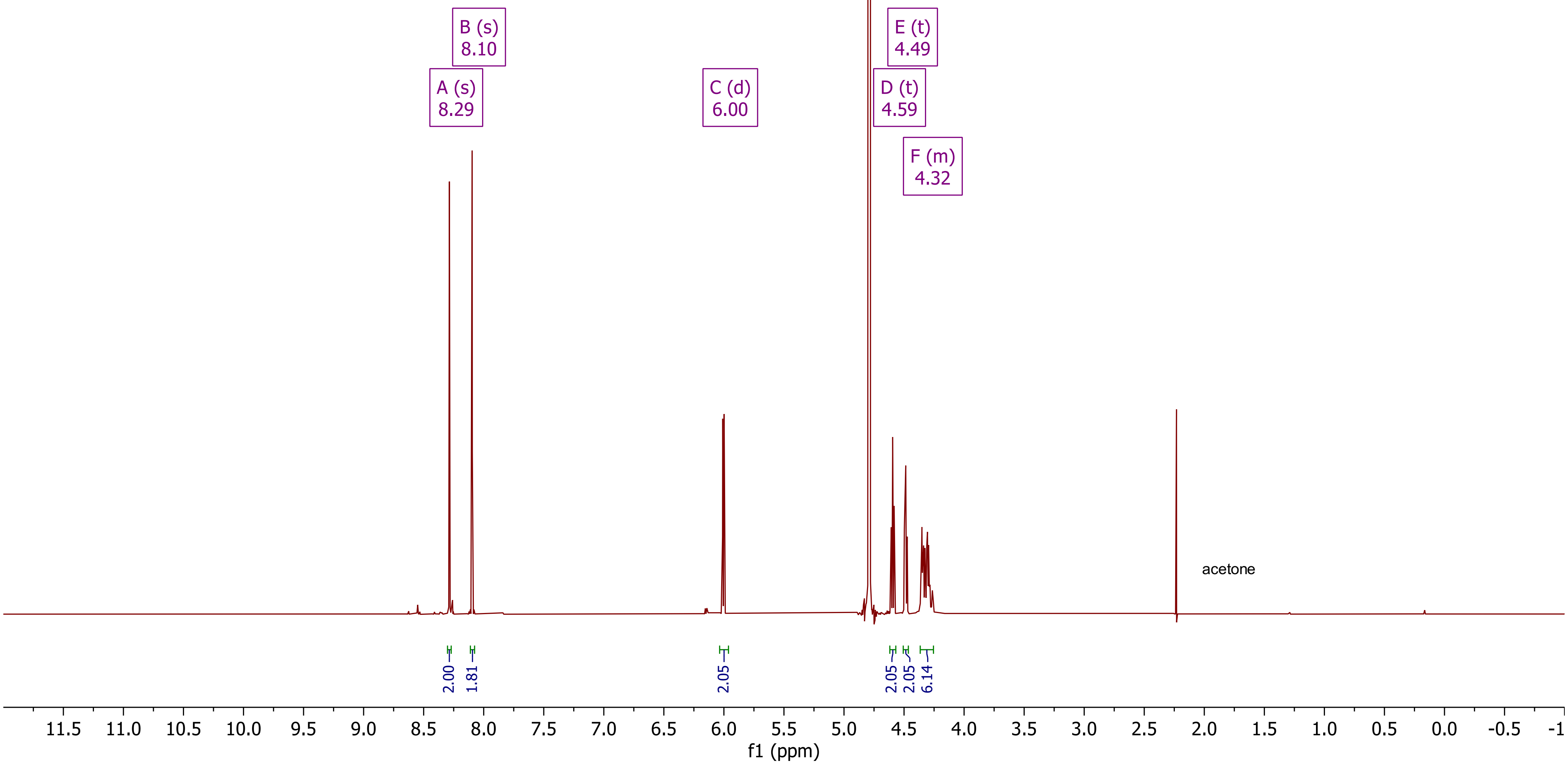

Compound 40: ^{18}O - Ap3A, ^{31}P{^{1}H} - NMR (D_2O, 162 MHz)

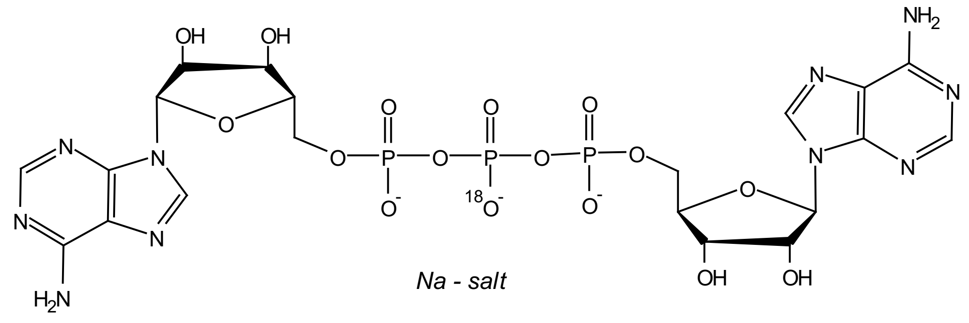

40

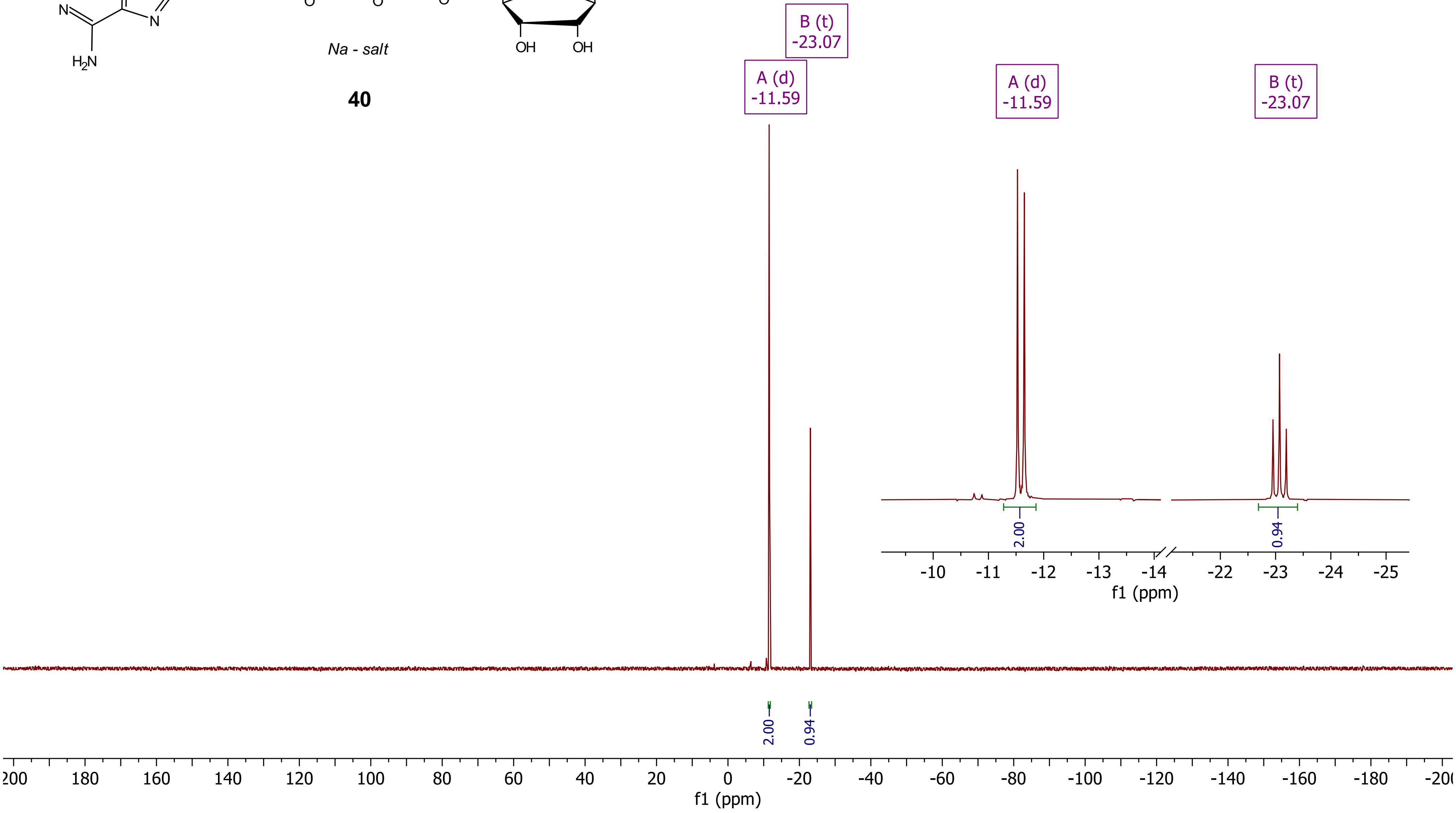

Compound 40: ^{18}O - Ap3A, ^{13}C{^{1}H} - NMR (D$_2$O, 101 MHz)

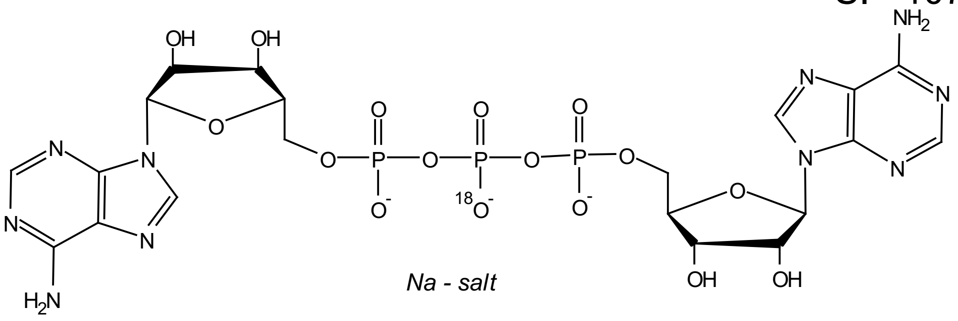

40

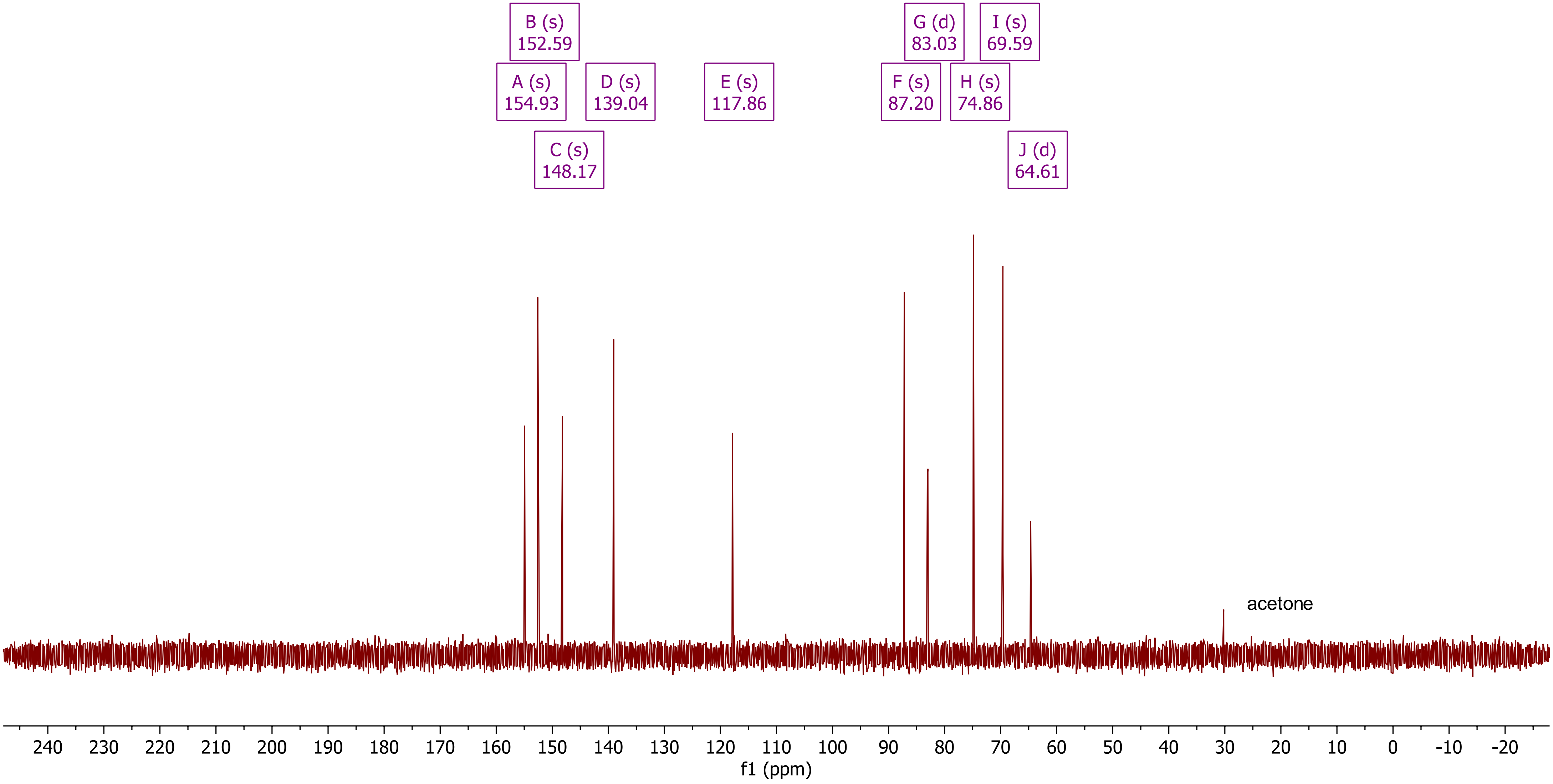

Compound 54: $^{18}O_2$ - pAp, ^{1}H - NMR (D_2O, 400 MHz)

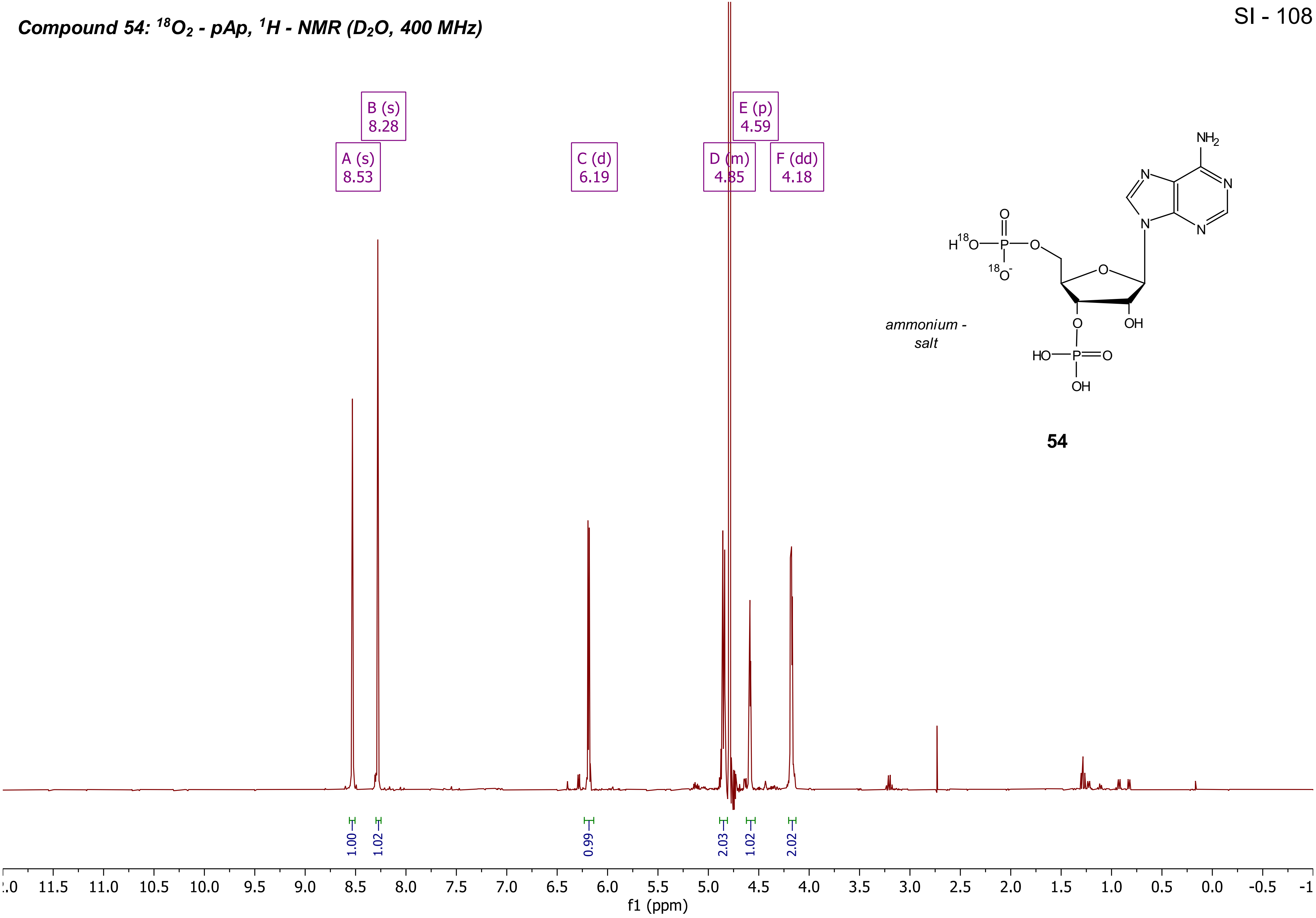

Compound 54: $^{18}O_2$ - pAp, $^{31}P\{^1H\}$ - NMR (D_2O, 162 MHz)

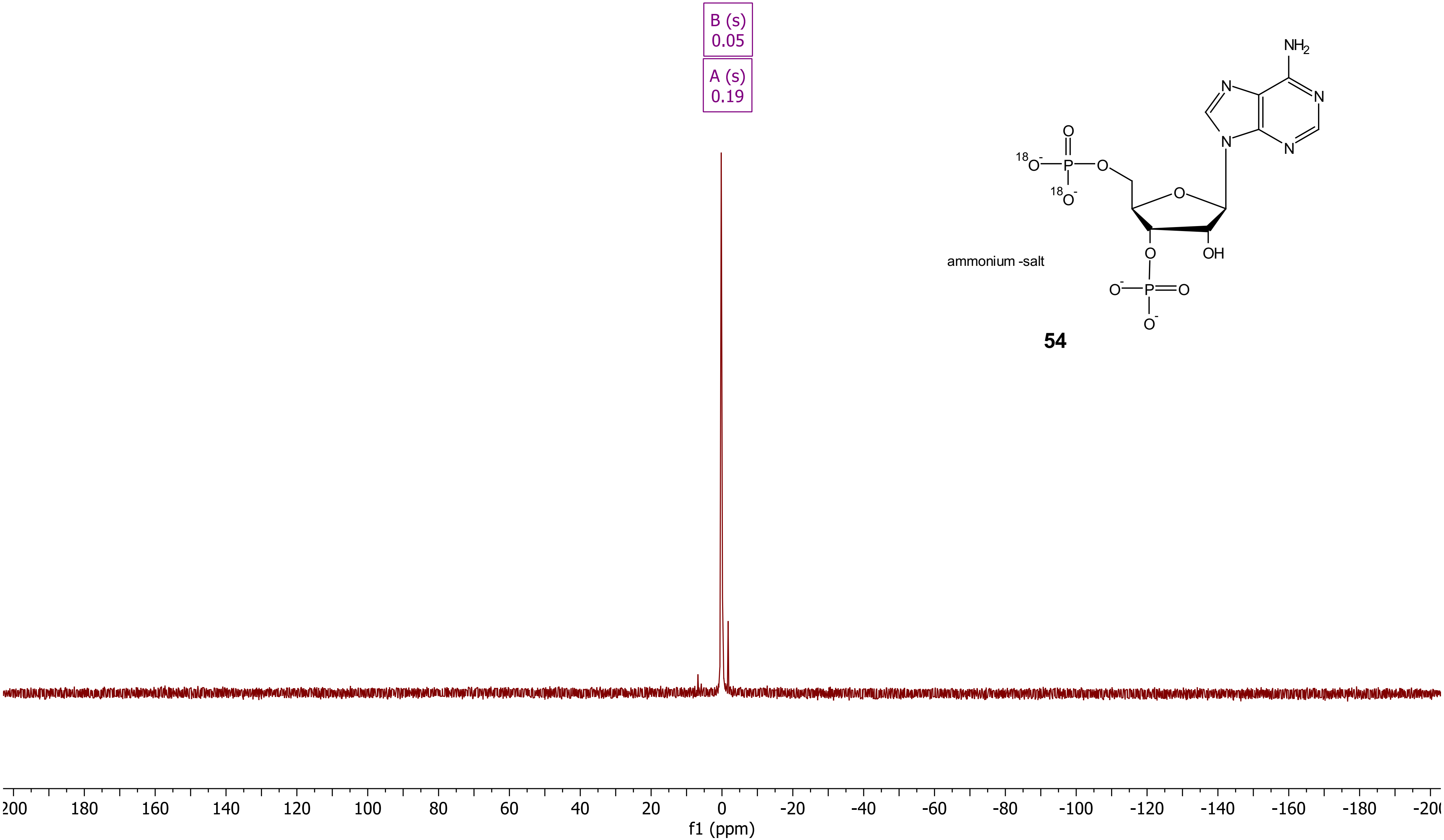

Compound 54: $^{18}O_2$ - pAp, $^{13}C\{^1H\}$ - NMR (D_2O, 101 MHz)

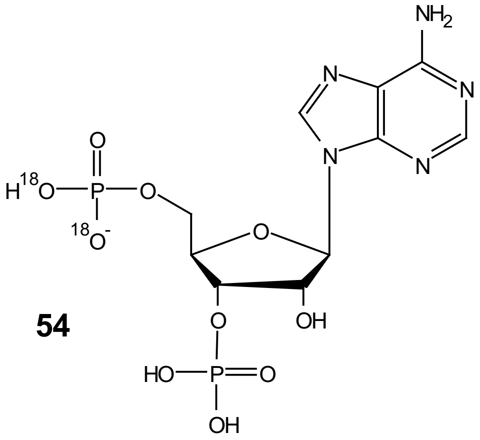

54

ammonium - salt

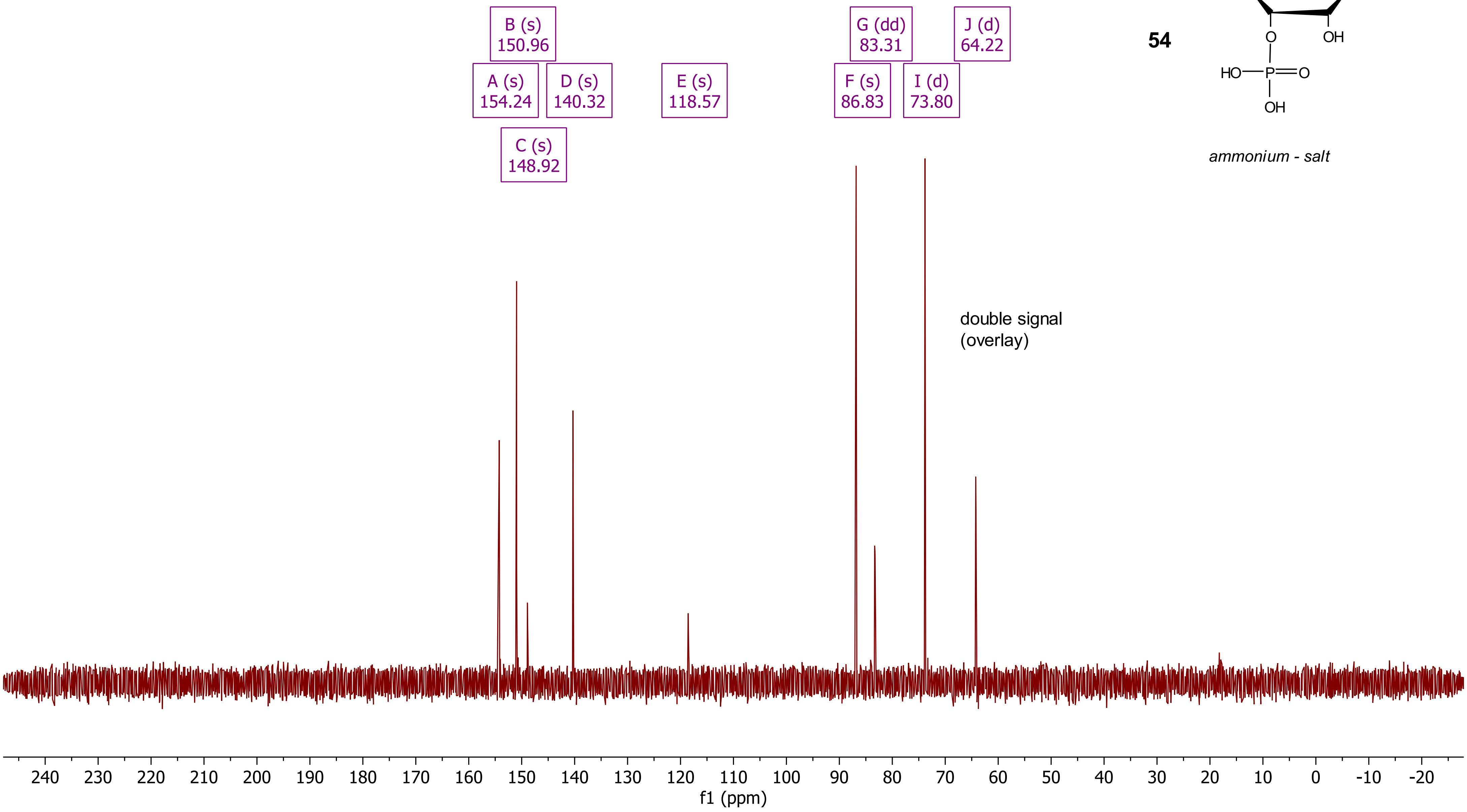

Compound 41: $^{18}O_2$ - PAPS, ^1H - NMR (D_2O, 400 MHz)

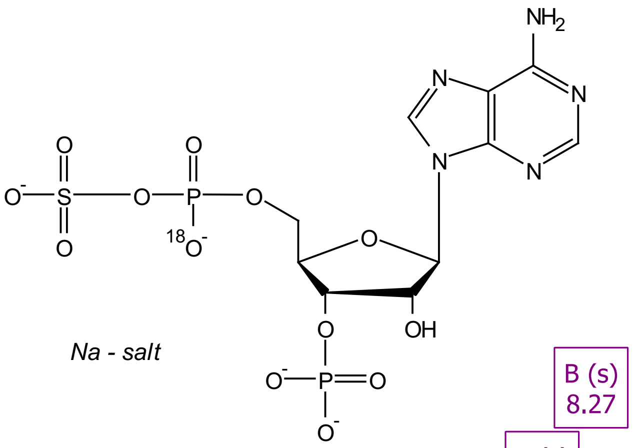

41

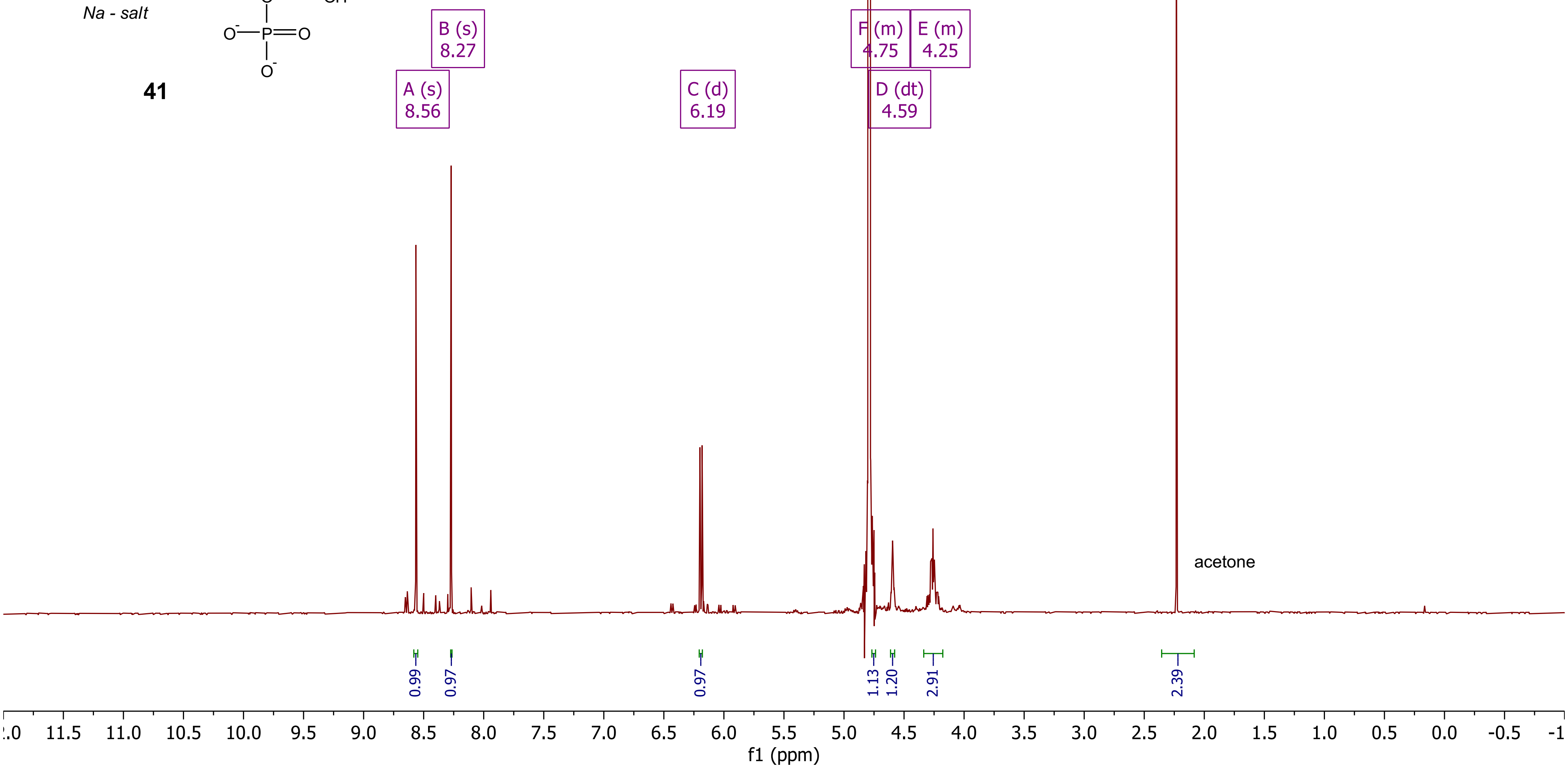

Compound 41: $^{18}O_2$ - PAPS, $^{31}P\{^1H\}$ - NMR (D_2O, 162 MHz)

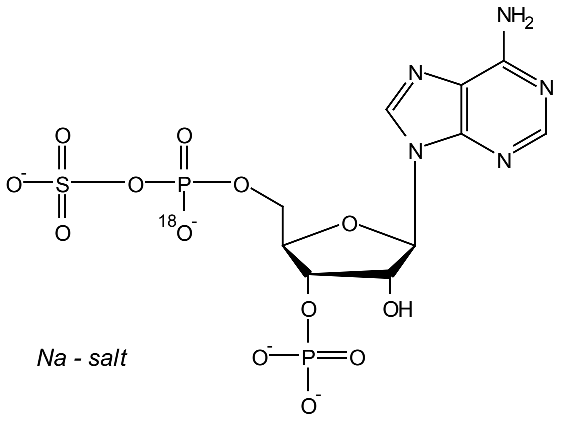

41

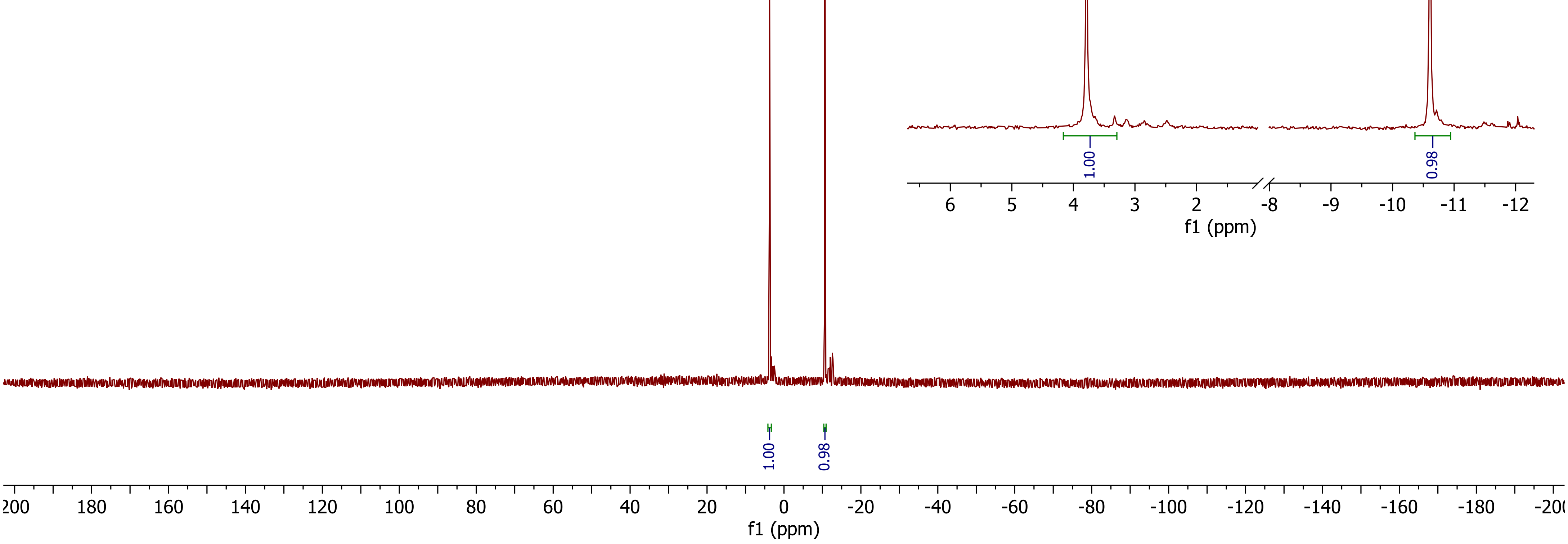

Compound 41: $^{18}O_2$ - PAPS, $^{13}C\{^1H\}$ - NMR (D_2O, 101 MHz)

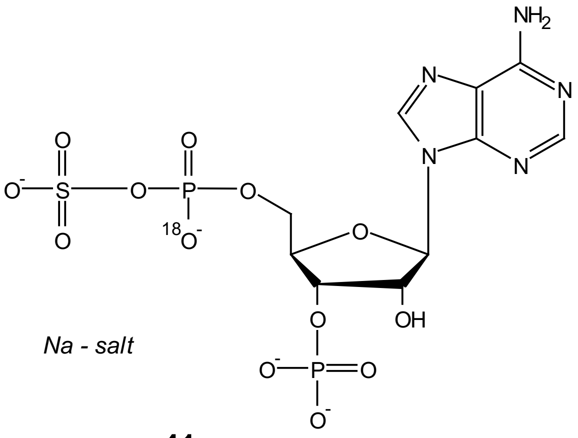

Na - salt

41

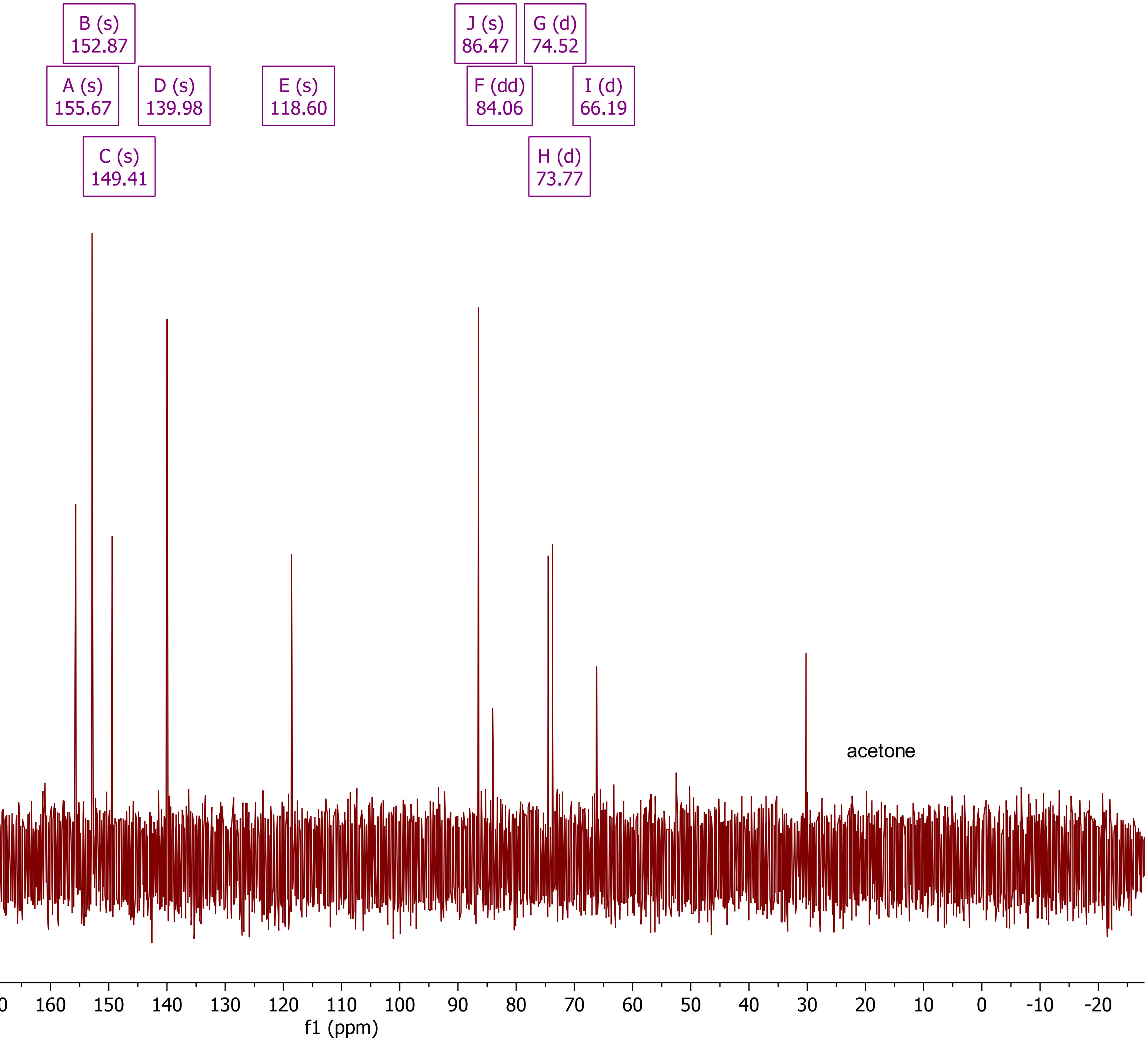

Compound 42: $^{18}O_4$ - P4, $^{31}P\{^1H\}$ - NMR (D_2O, 162 MHz)

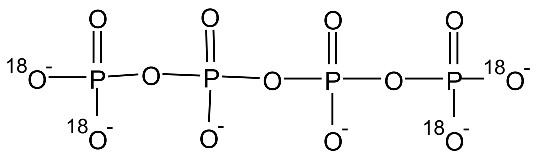

Na - salt

42

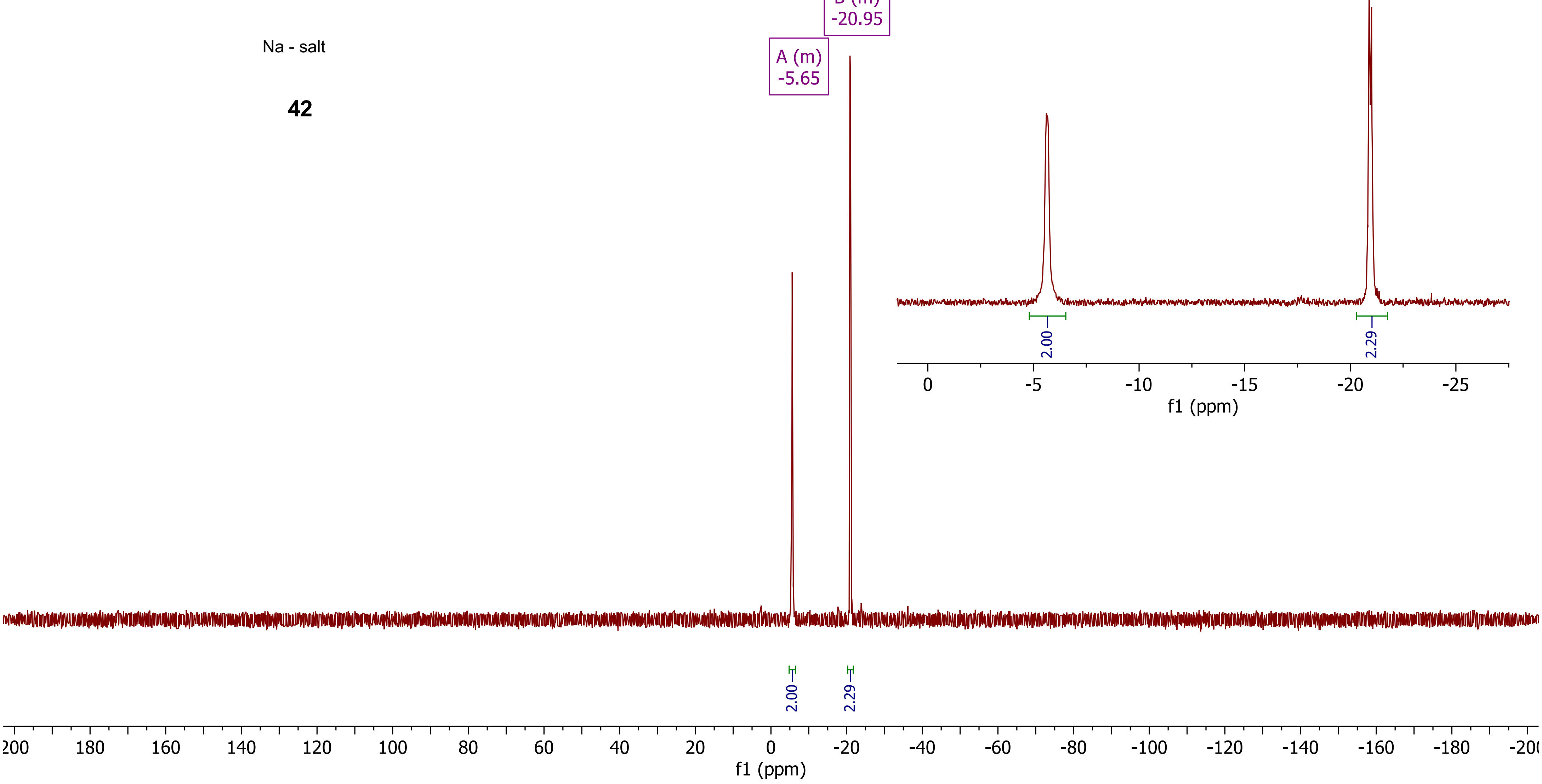

Compound SI-4: Isoprenylphosphate, ^1H - NMR (D_2O, 400 [illegible]

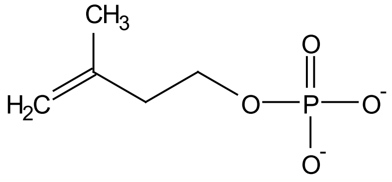

1.5 piperidinium - salt
0.14 TEAA - salt

SI-4

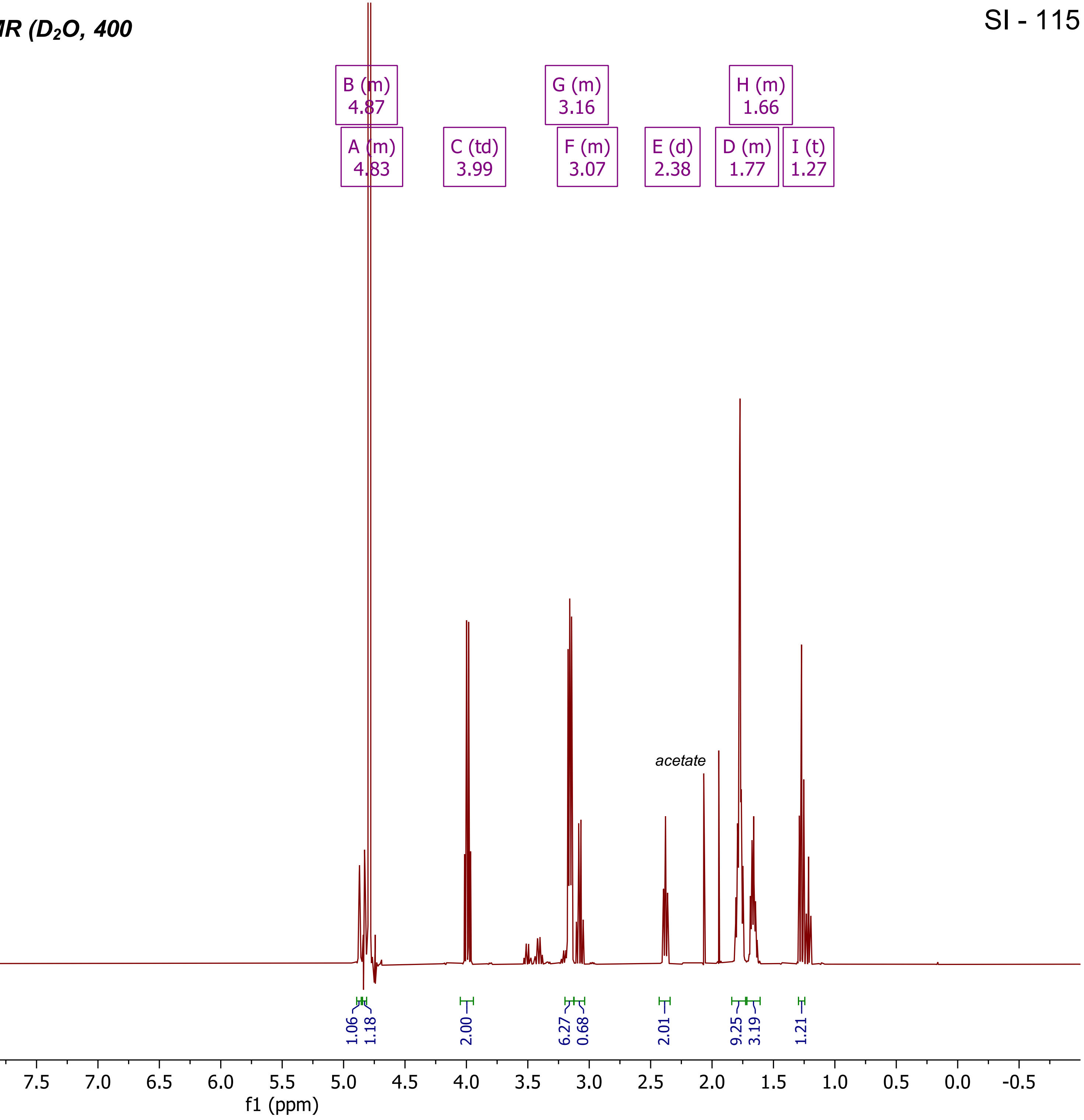

Compound SI-4: Isoprenylphosphate, $^{31}P\{^{1}H\}$ - NMR (D2O, 162 [illegible]

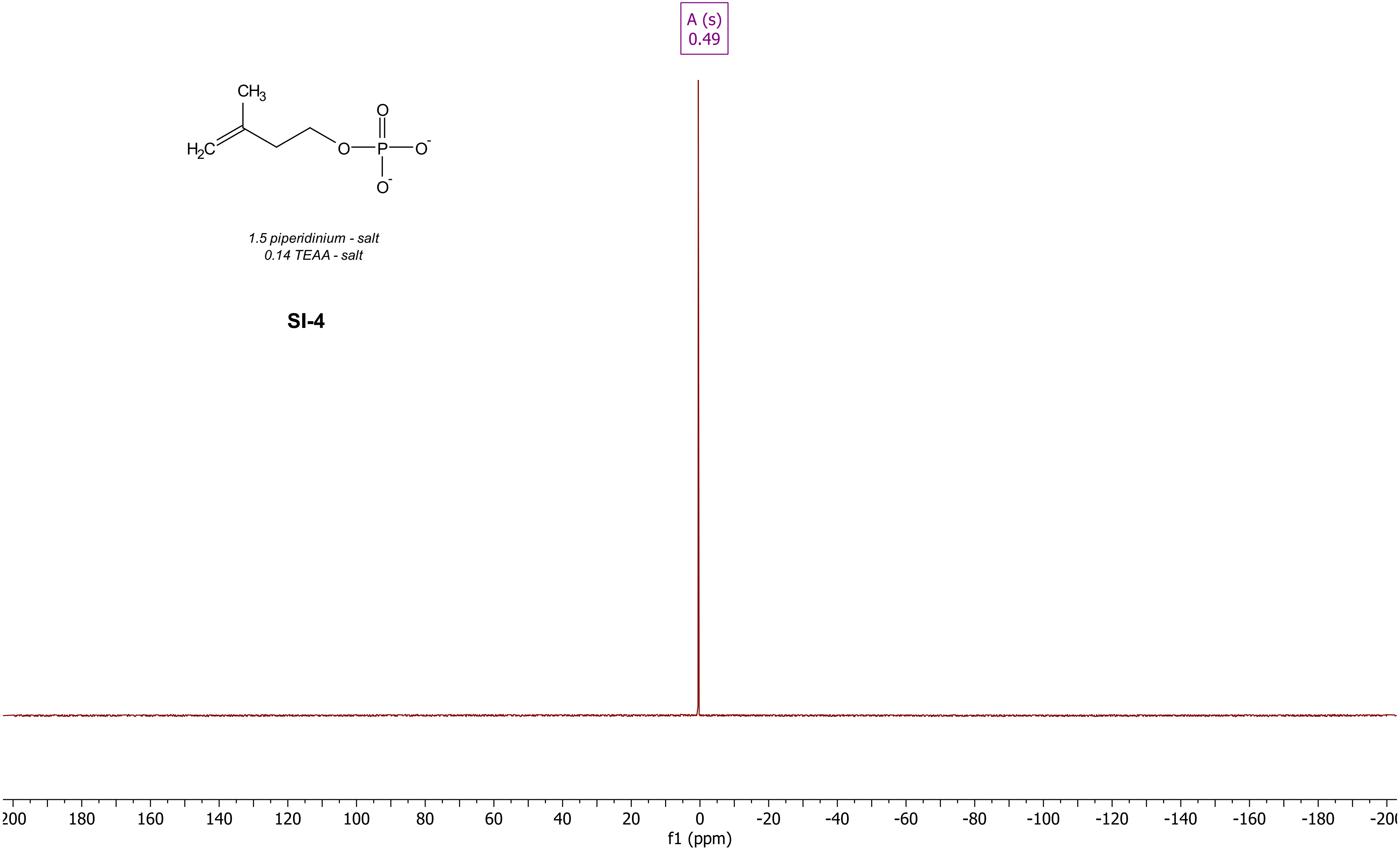

Compound SI-4: Isoprenylphosphate, $^{13}C\{^{1}H\}$ - NMR (D2O, 101 [illegible]

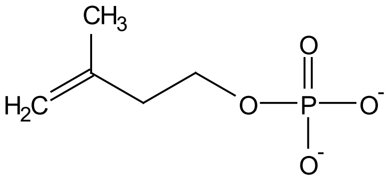

1.5 piperidinium - salt
0.14 TEAA - salt

SI-4

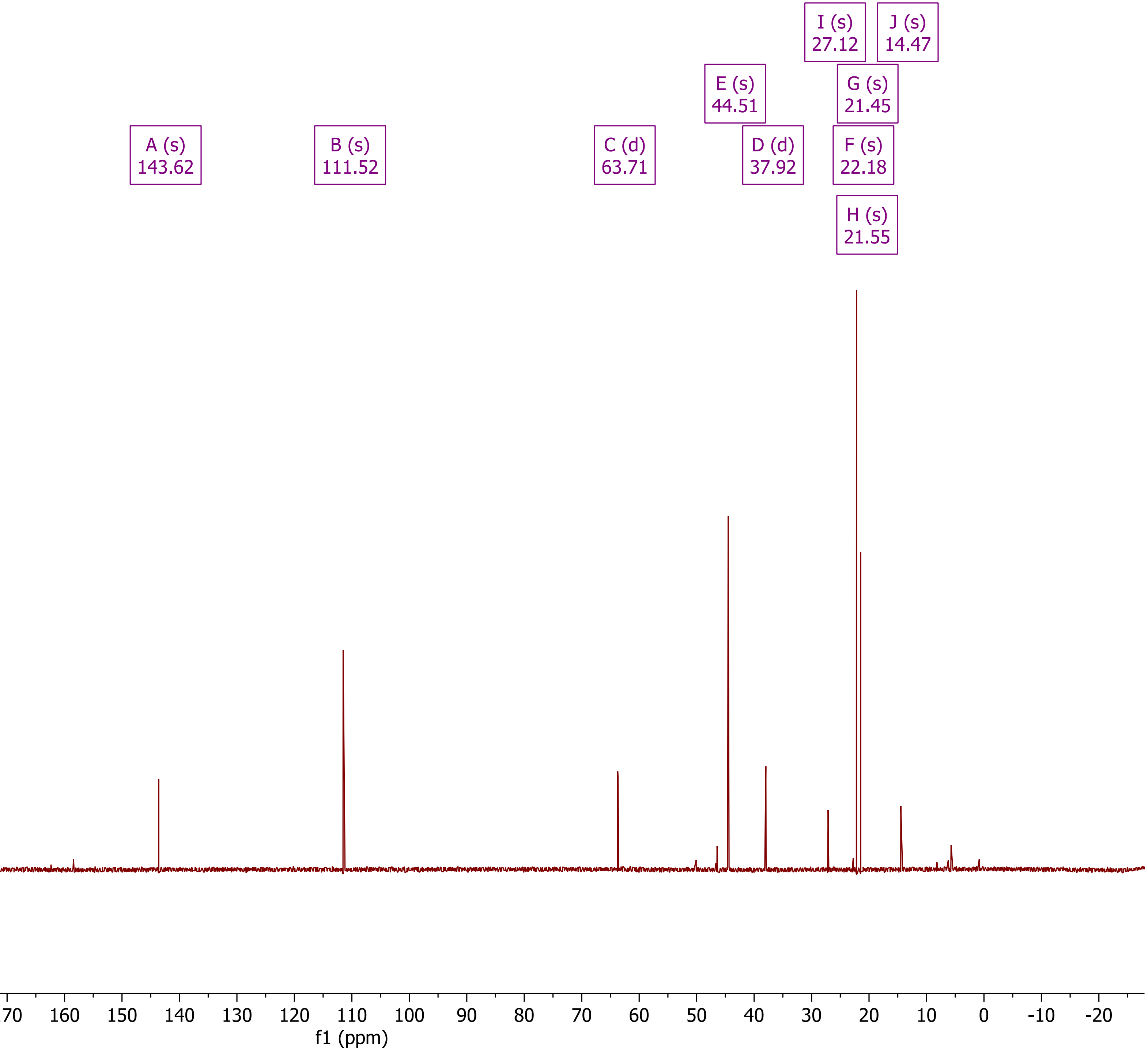

Compound 43: $^{18}O_2$ - IPP, ^{1}H - NMR (D_2O, 400 MHz)

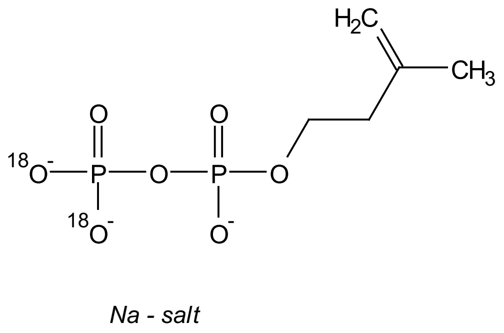

43

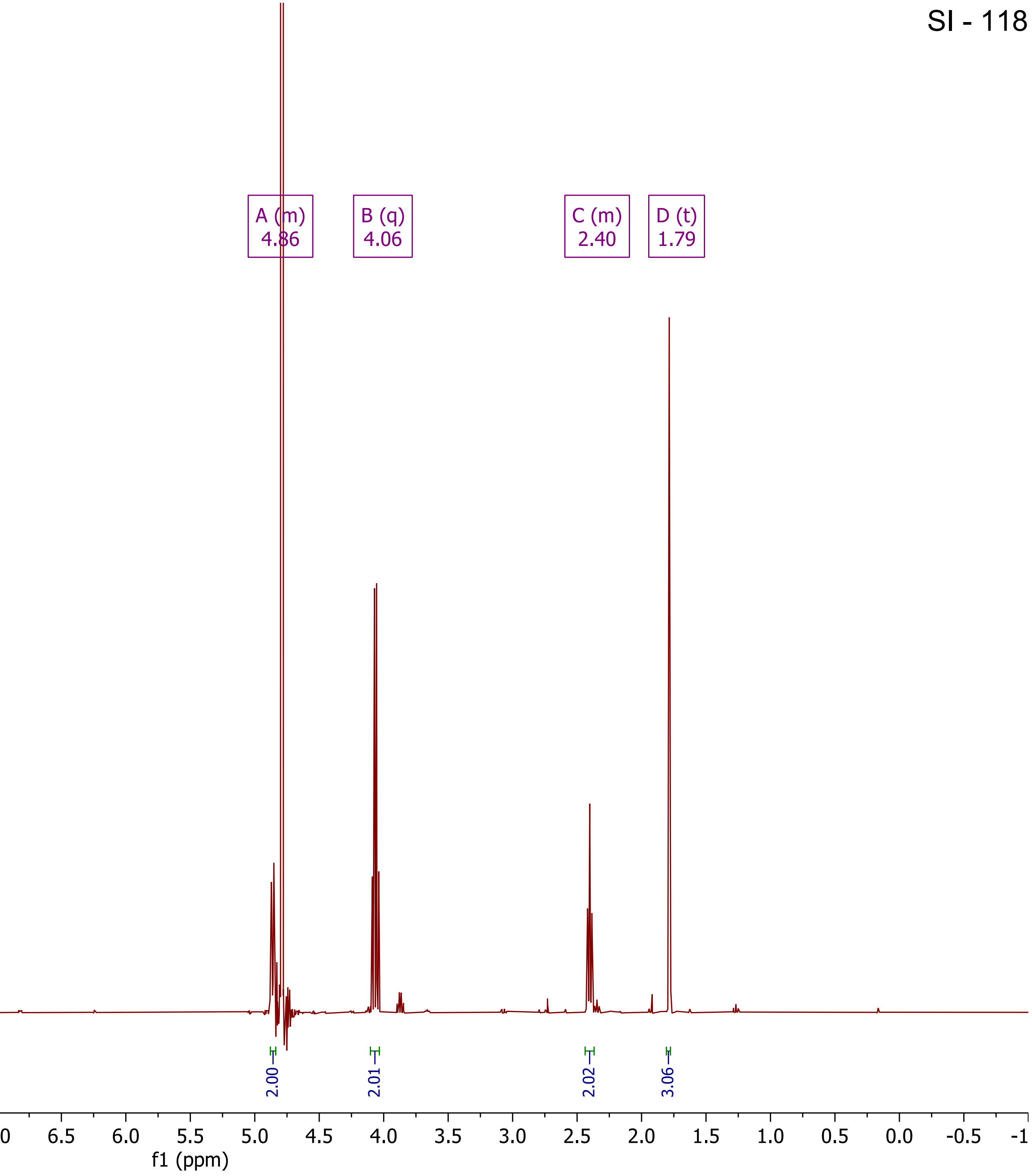

Compound 43: $^{18}O_2$ - IPP, $^{31}P\{^1H\}$ - NMR (D_2O, 162 MHz)

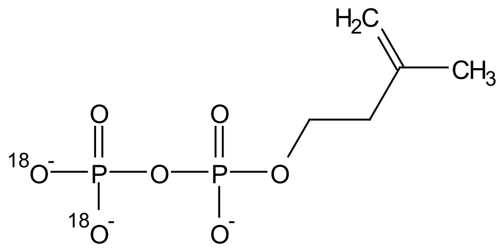

Na - salt

43

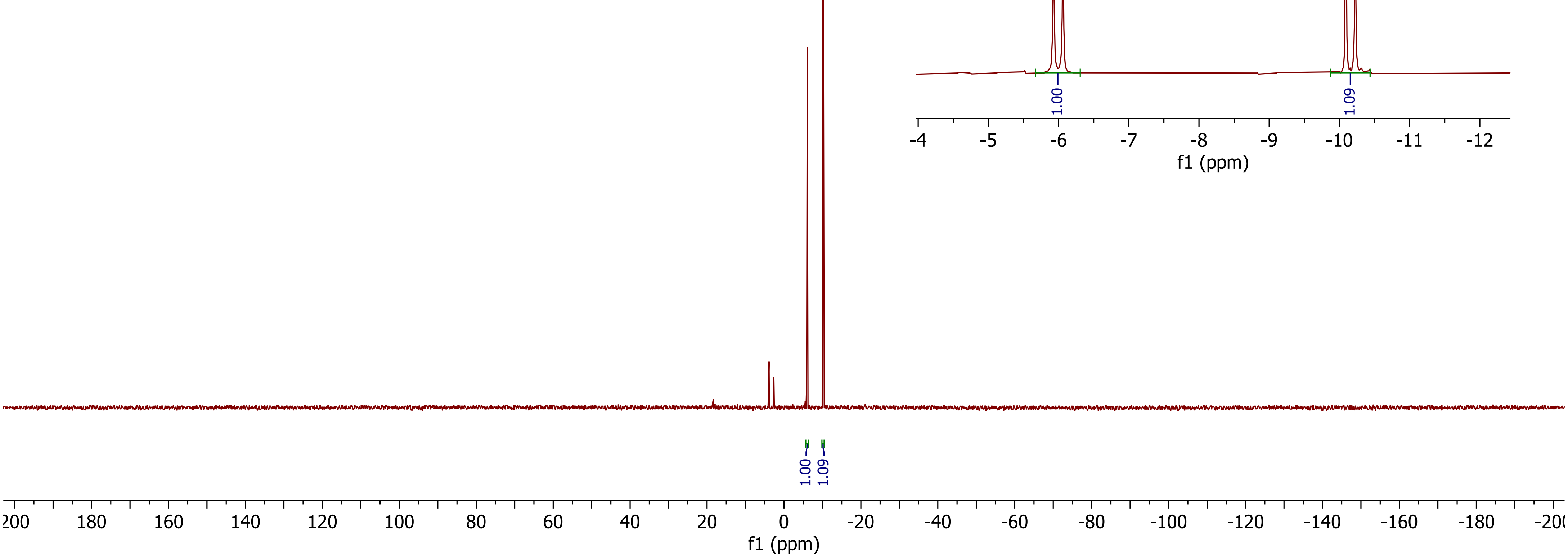

Compound 43: $^{18}O_2$ - IPP, $^{13}C\{^1H\}$ - NMR (D_2O, 101 MHz)

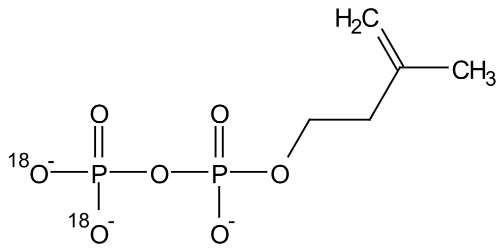

Na - salt

43

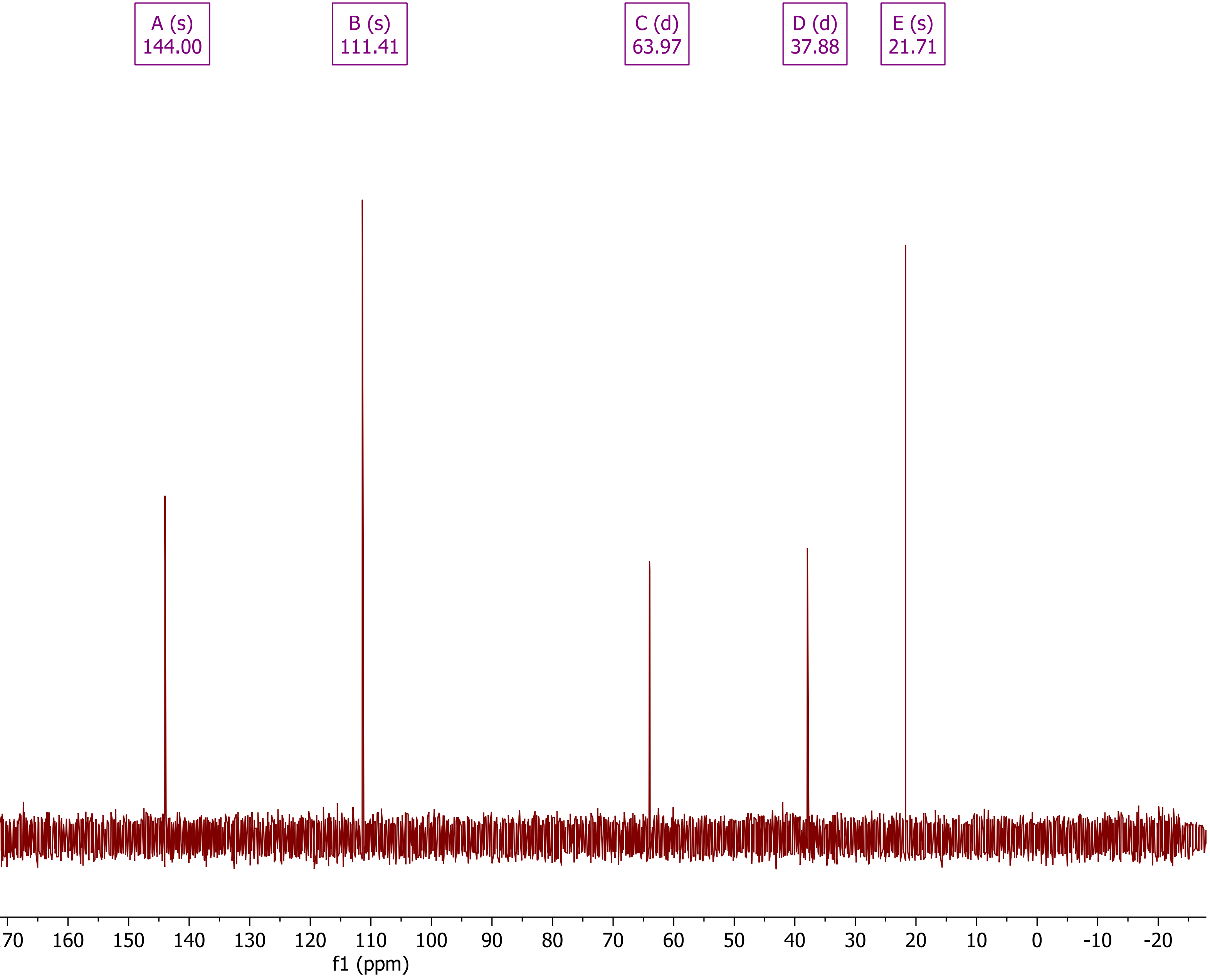

Compound 44: ^{18}O4 - ppGpp, ^{1}H - NMR (D_2O, 400 MHz)

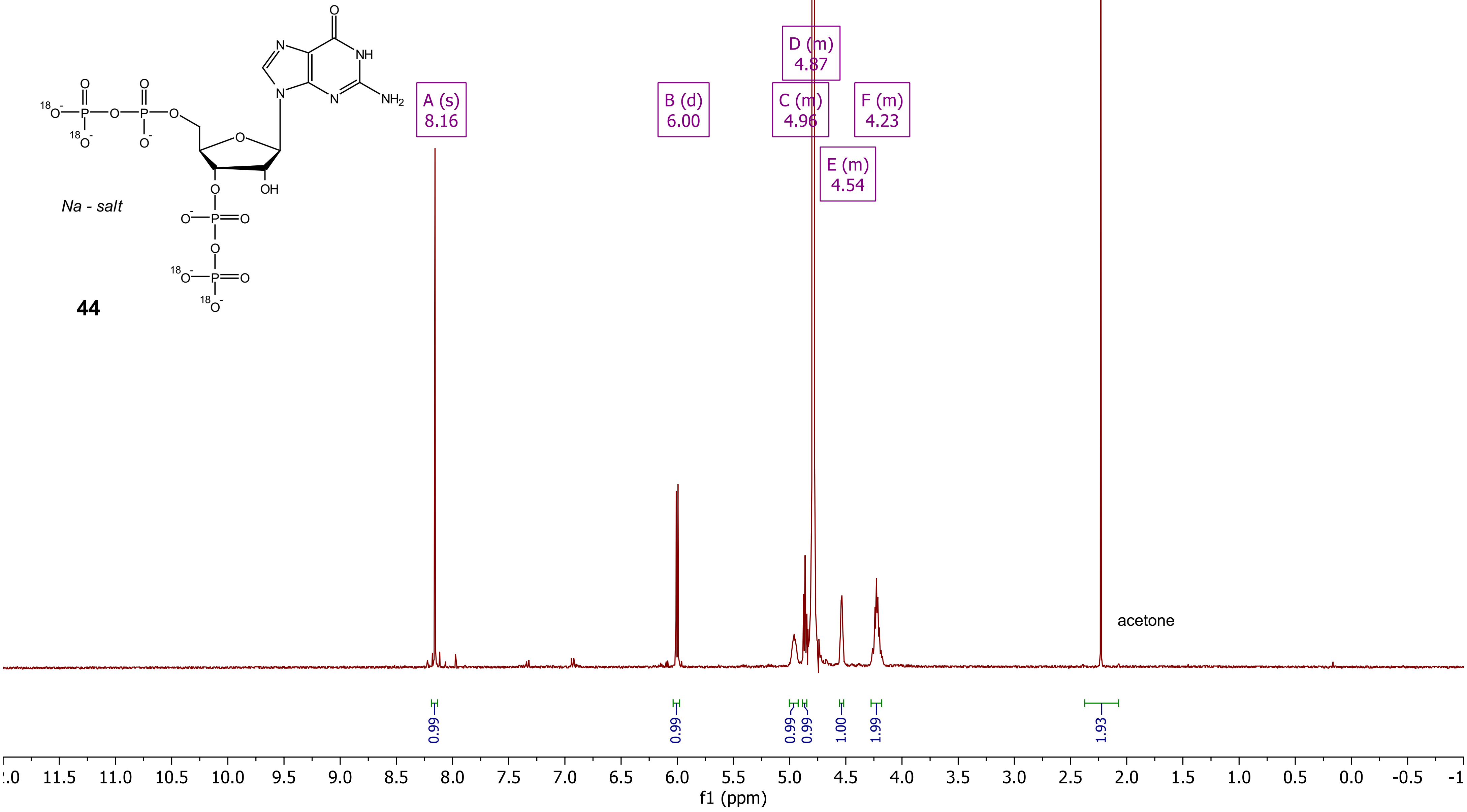

Compound 44: $^{18}O_2$ - ppGpp, $^{31}P\{^1H\}$ - NMR (D_2O, 162 MHz)

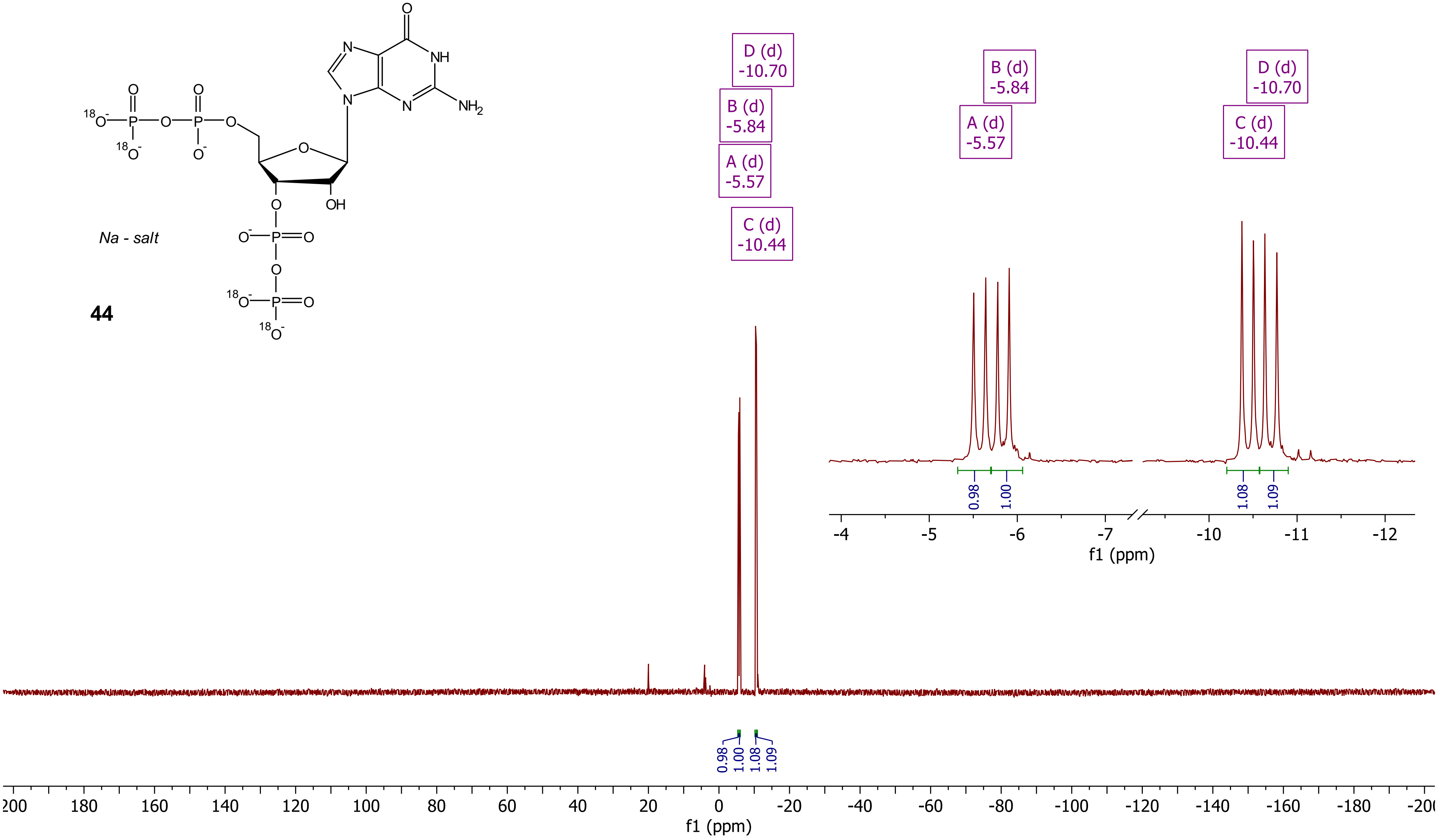

Na - salt

44

Compound 44: $^{18}O_2$ - ppGpp, $^{13}C\{^1H\}$ - NMR (D_2O, 101 MHz)

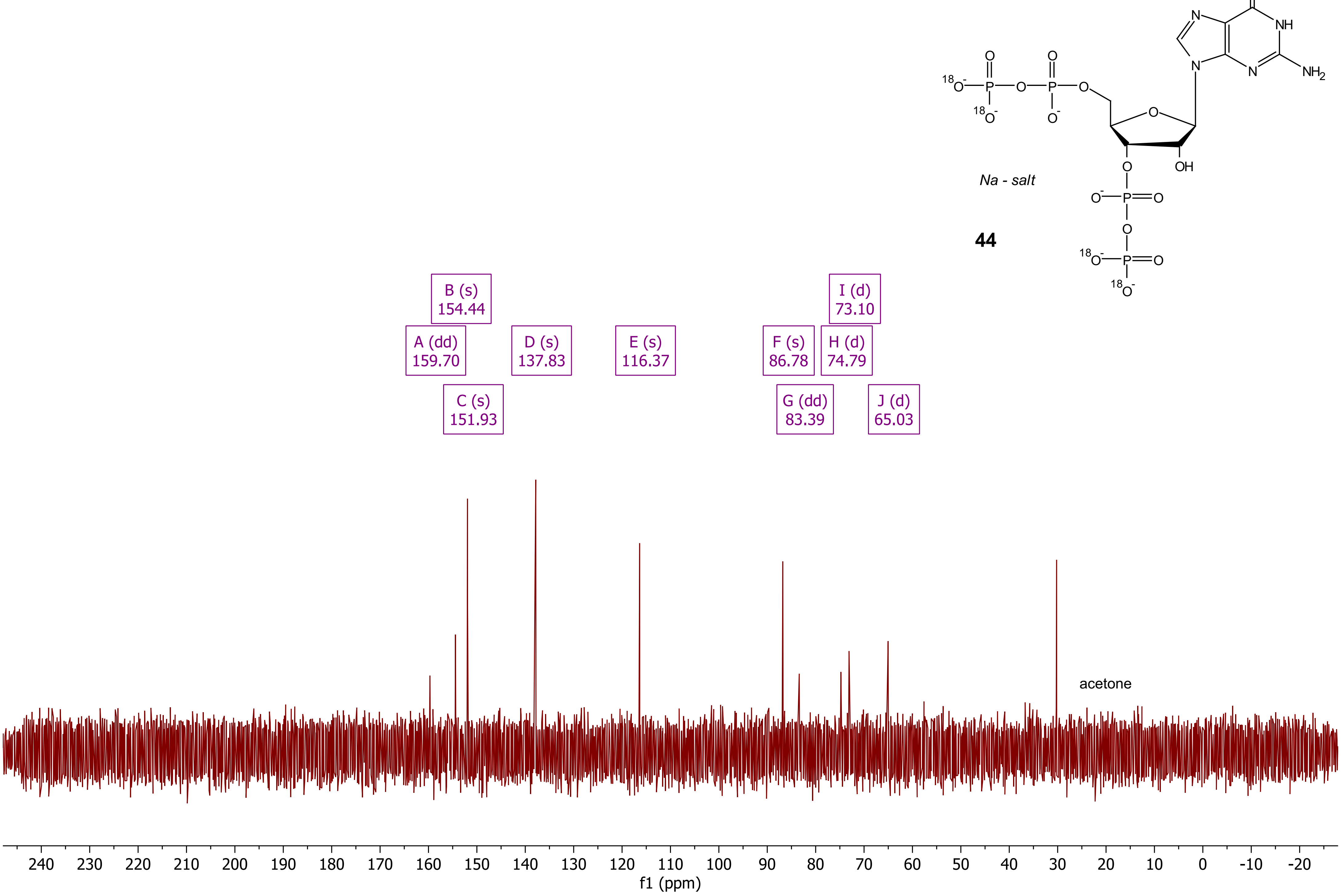

Compound 45: **$^{18}O_2$-ppGp, *^{1}H - NMR (D_2O, 400 MHz)***

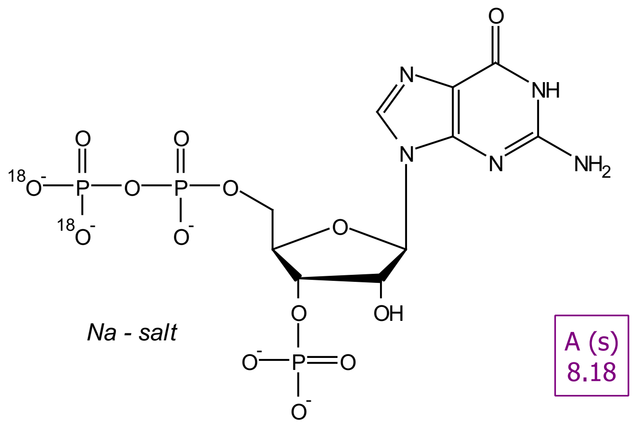

45

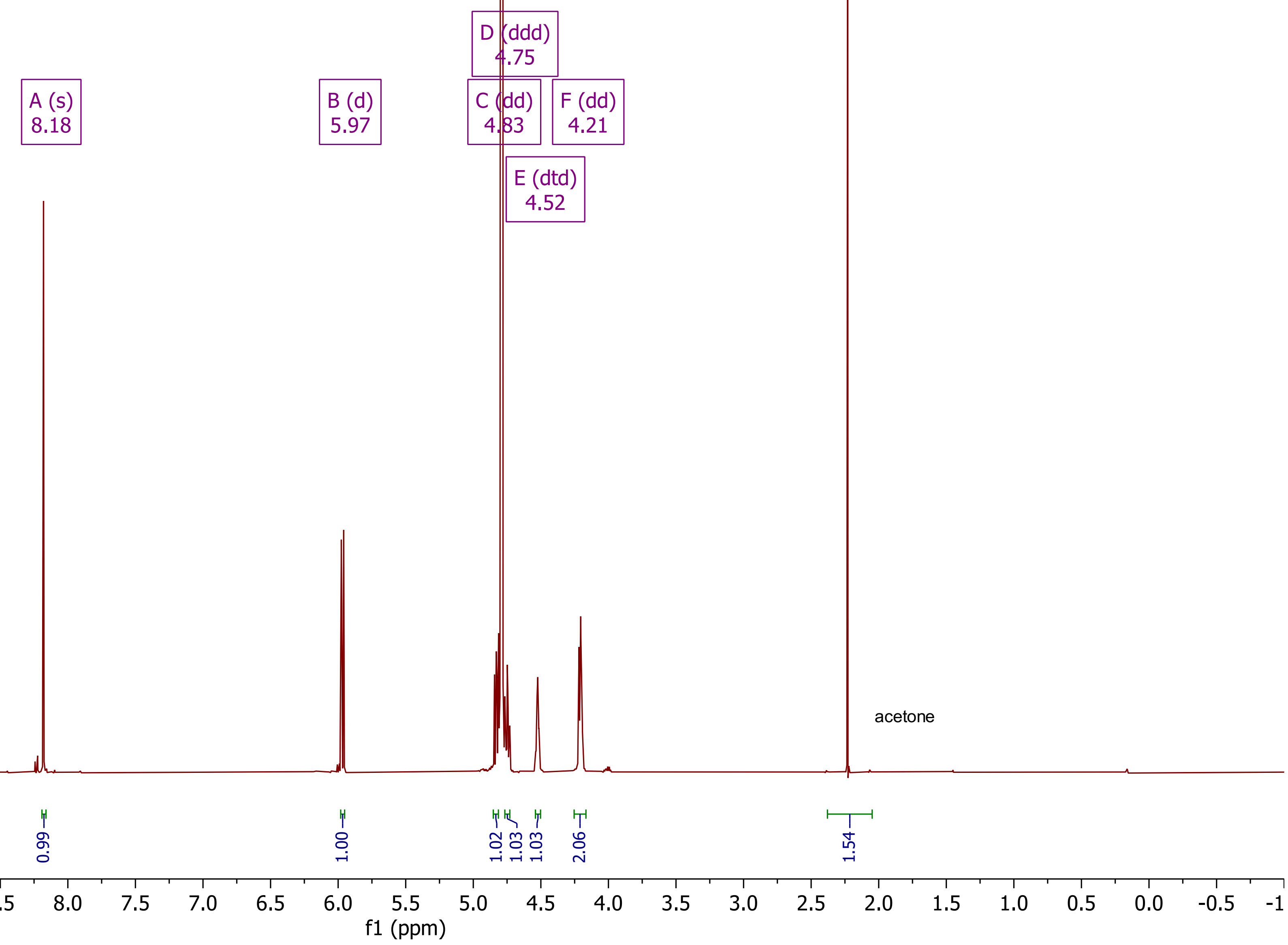

Compound 45: **$^{18}O_2$-ppGp,** ***$^{31}P\{^1H\}$ - NMR (D_2O, 162 MHz)***

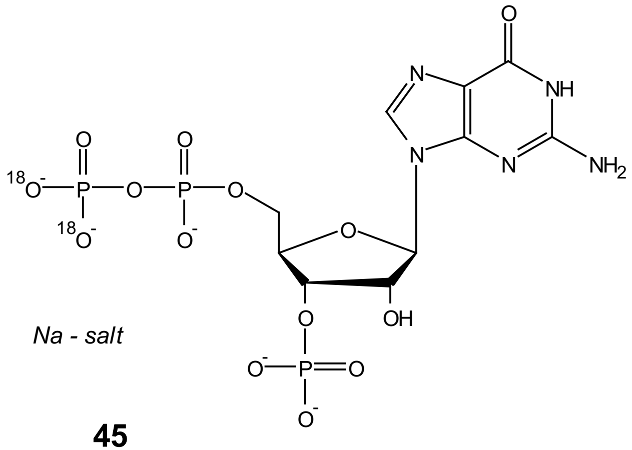

Na - salt

45

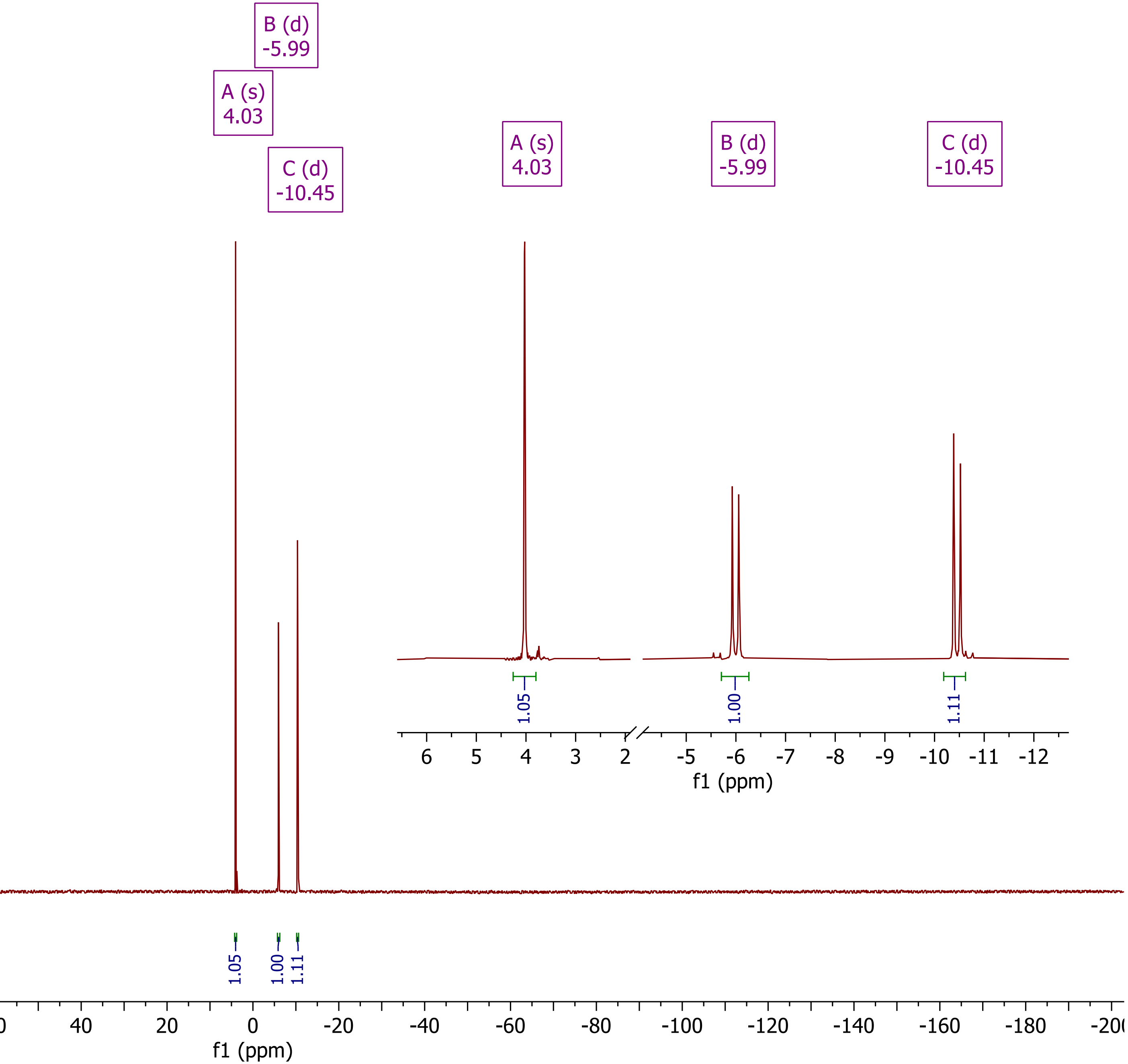

Compound 45: **$^{18}O_2$-ppGp,** ***$^{13}C\{^1H\}$* - NMR (D_2O, 101 MHz)**

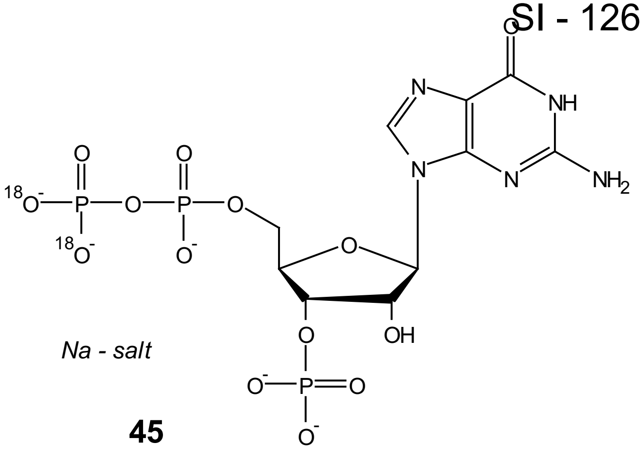

45

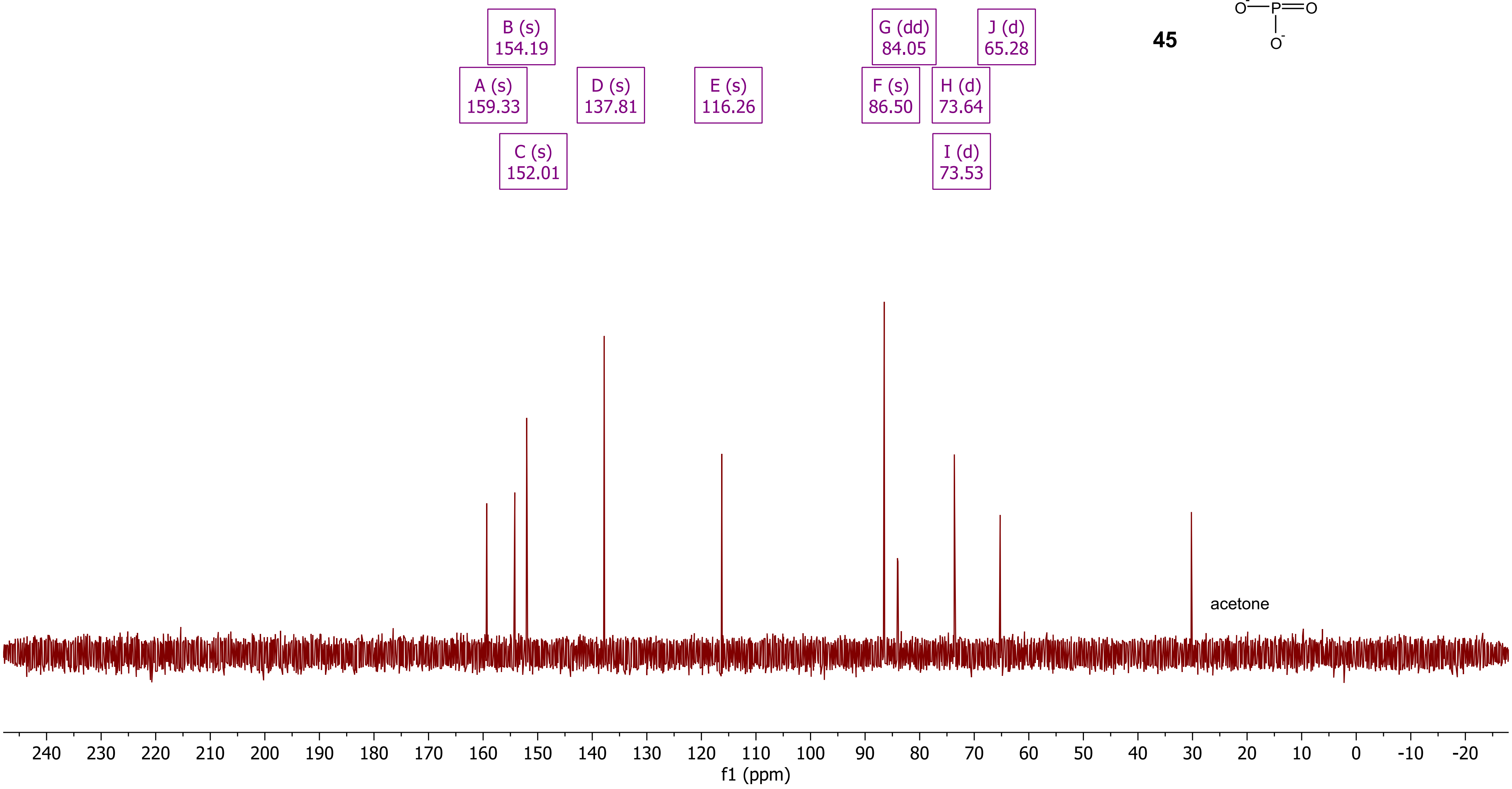

Compound SI-9: $^{18}O_{12}$ - AB_{12} - IP6, ^{1}H - NMR (D_2O, 400 MHz)

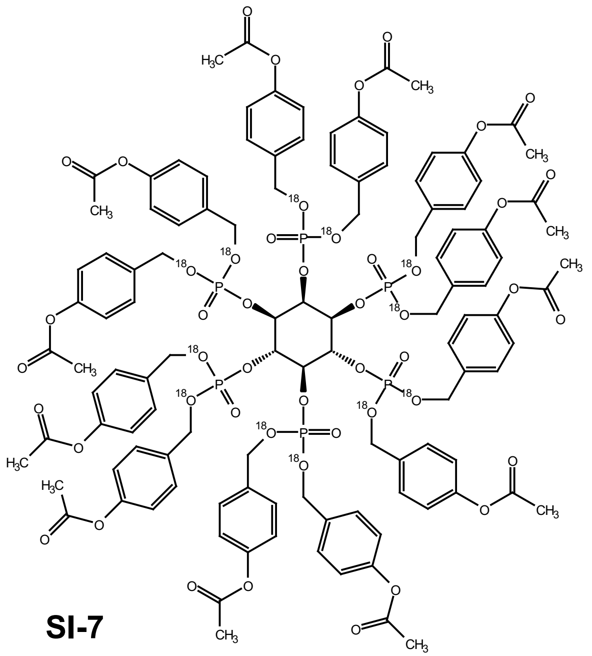

SI-7

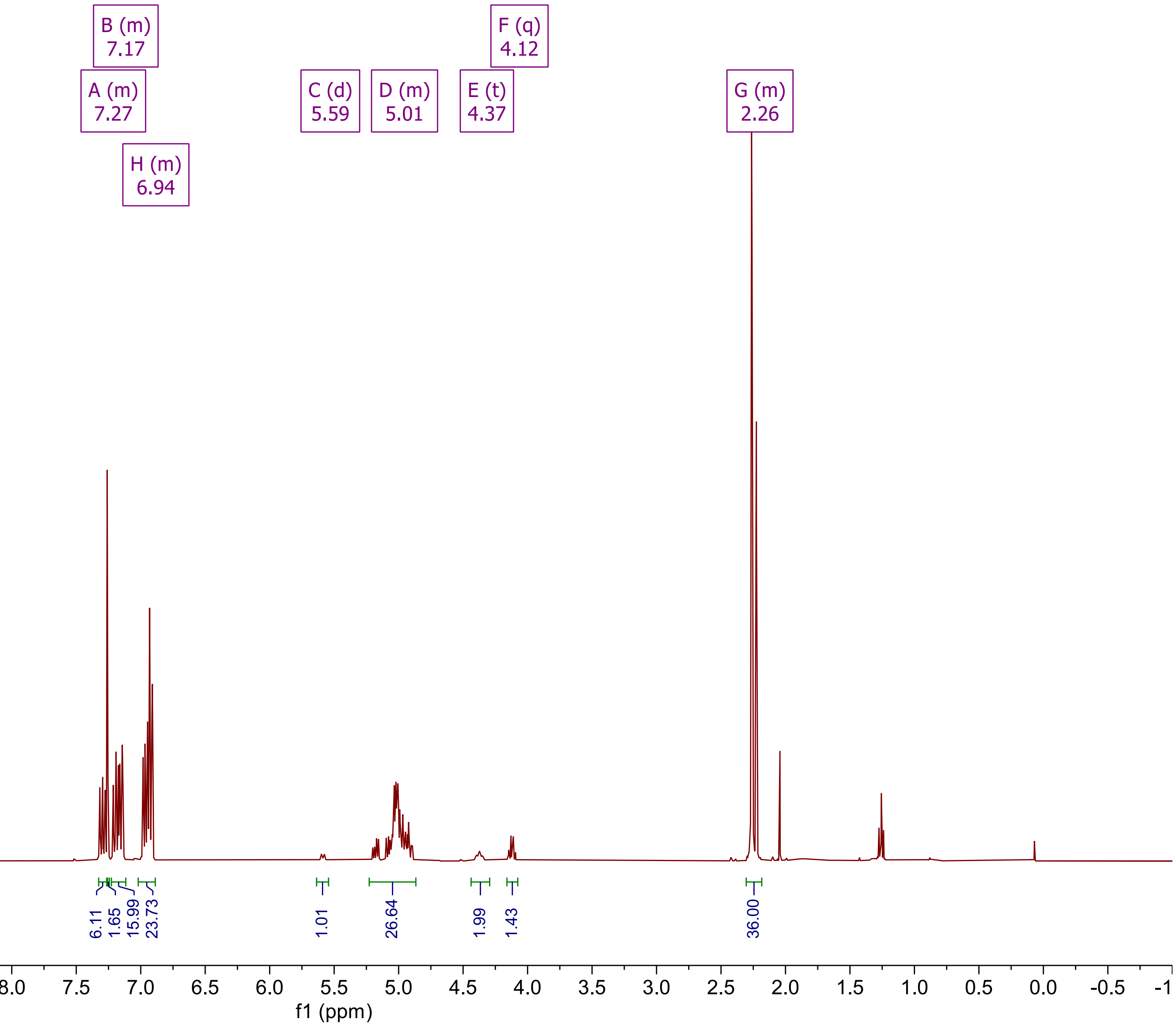

Compound XX: $^{18}O_{12}$ - AB_{12} - IP6, $^{31}P\{^1H\}$ - NMR (D_2O, 162 MHz)

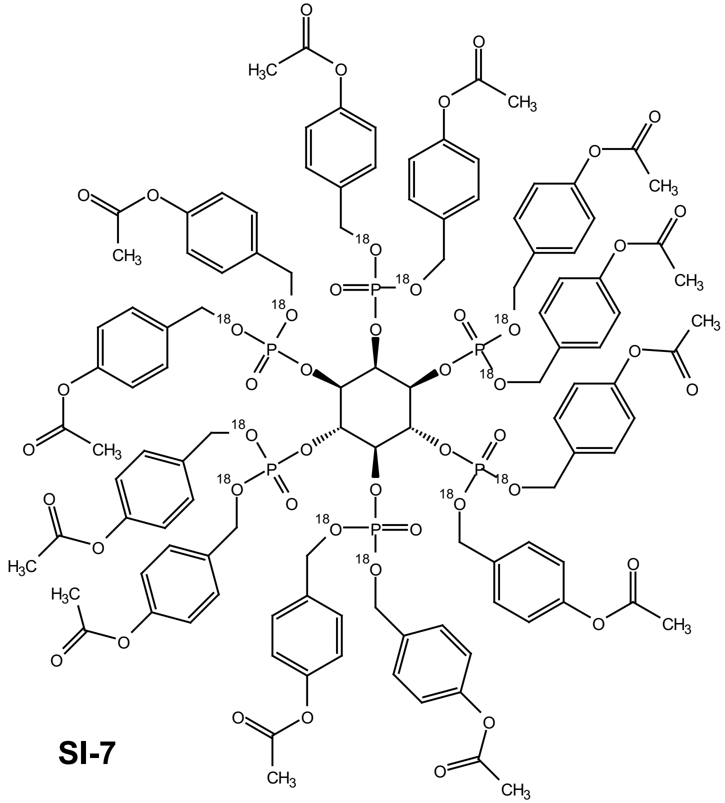

SI-7

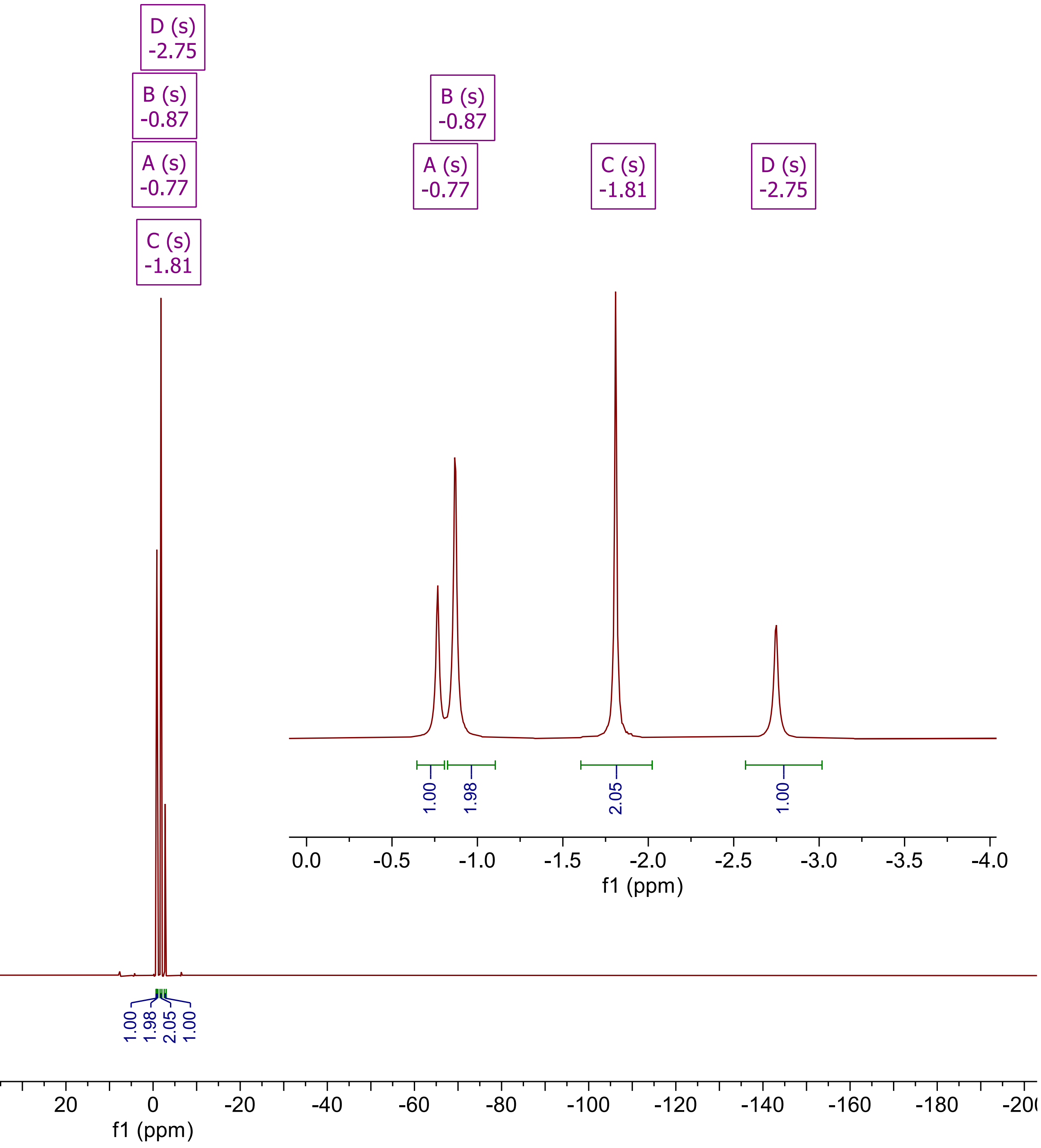

Compound 46: $^{18}O_{12}$ - IP6, ^{1}H - NMR (D_2O, 400 MHz)

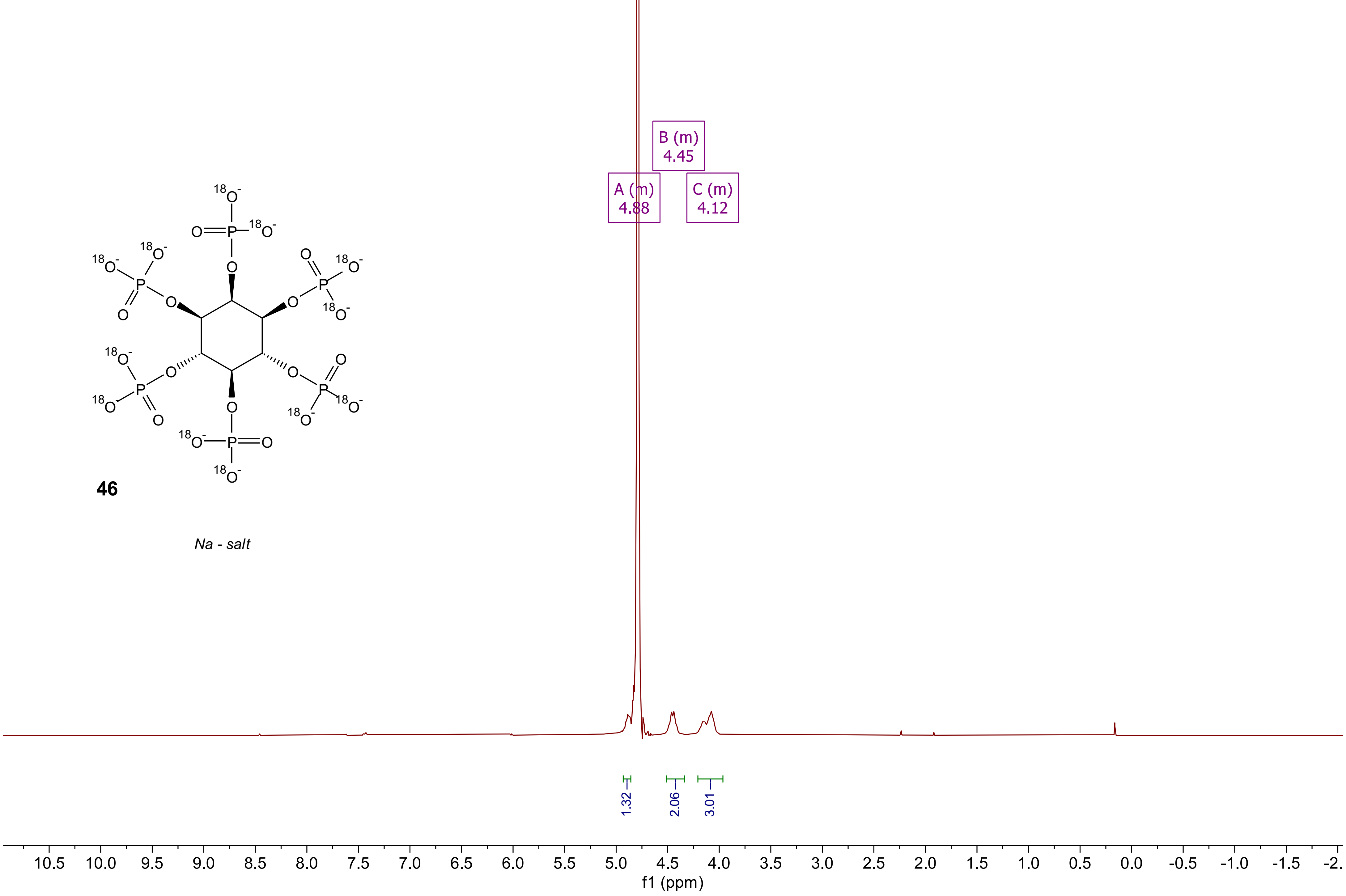

Compound 46: $^{18}O_{12}$ - IP6, $^{31}P\{^1H\}$ - NMR (D_2O, 162 MHz)

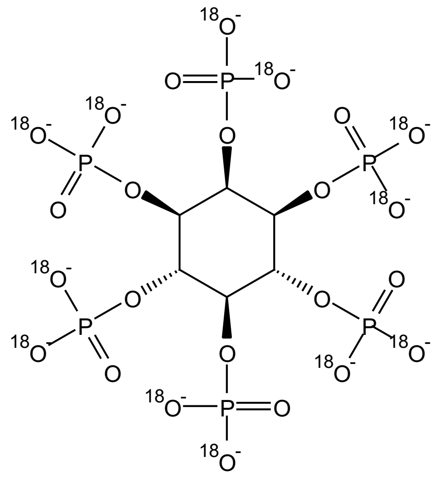

46

Na - salt

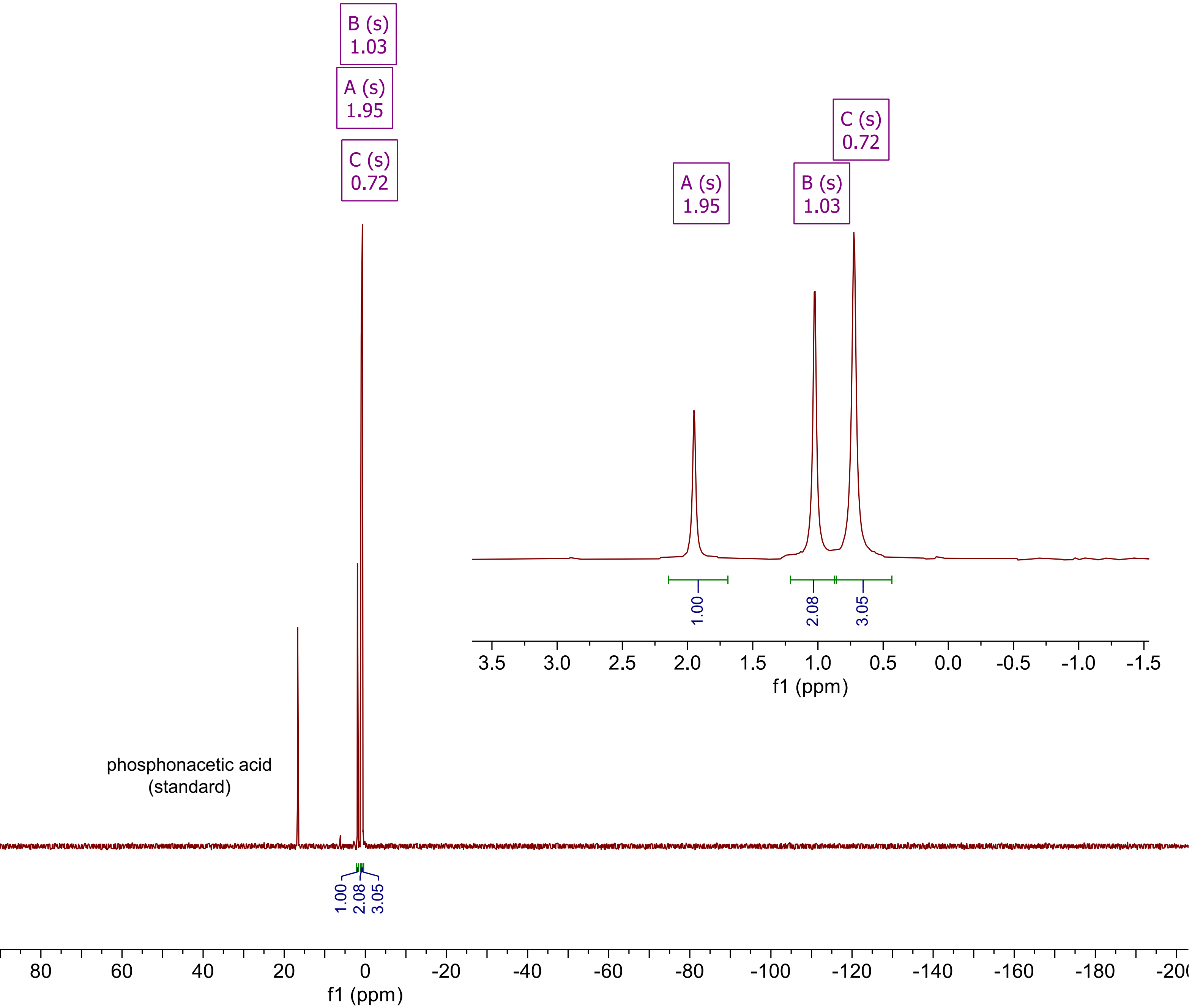

Compound SI-9: $^{18}O_2$ - AB_{11} - PMB - 5-IP7, ^{1}H - NMR (D_2O, 400 MHz)

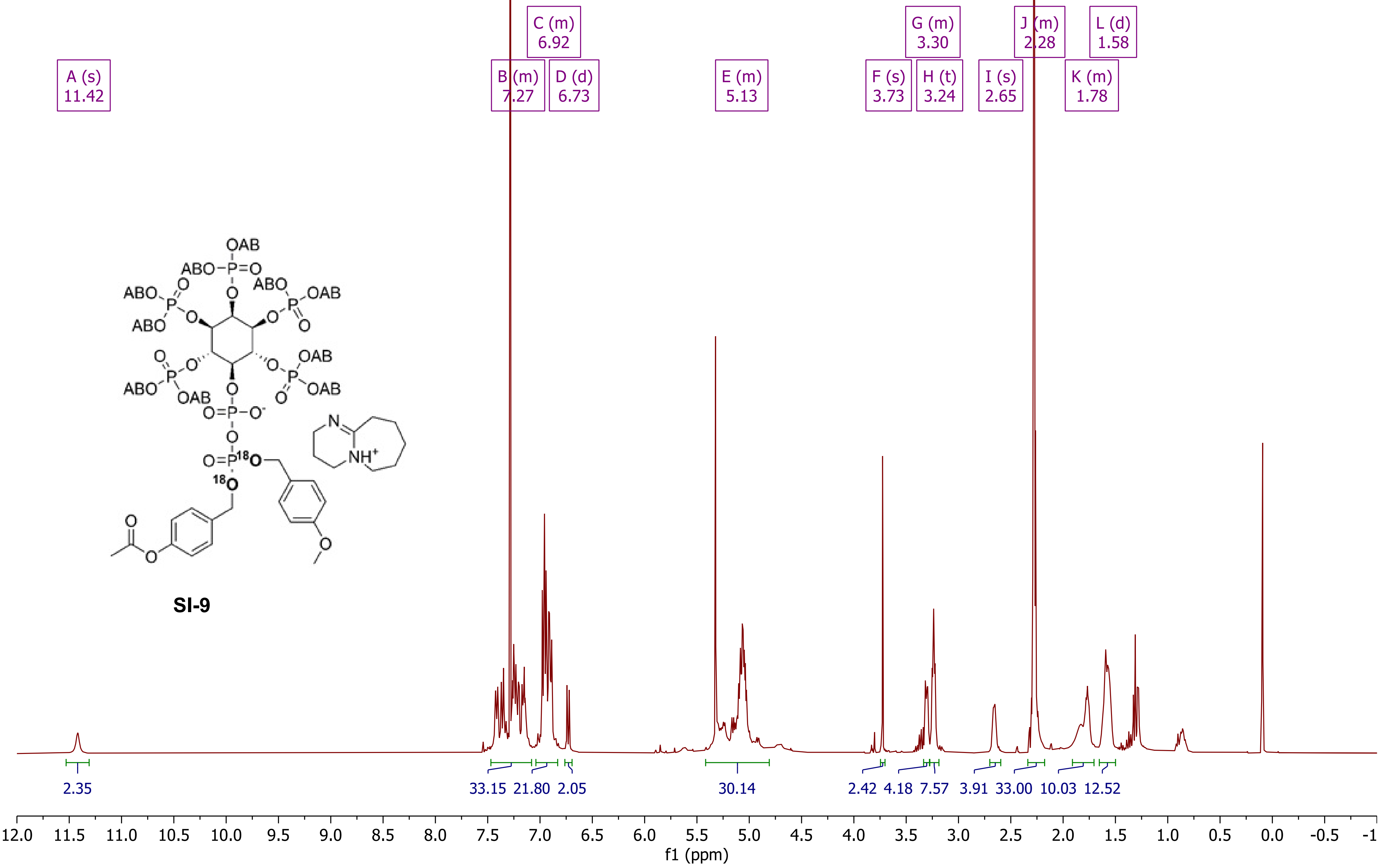

SI-9

Compound SI-9: $^{18}O_2$ - AB_{11} - PMB - 5-IP7, $^{31}P\{^1H\}$ - NMR (D_2O, 162 MHz)

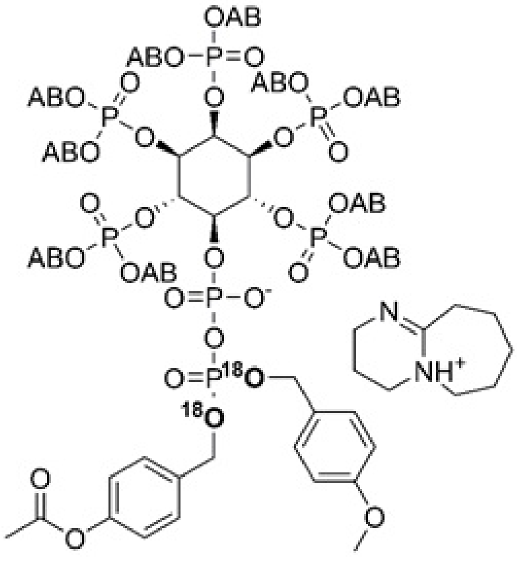

SI-9

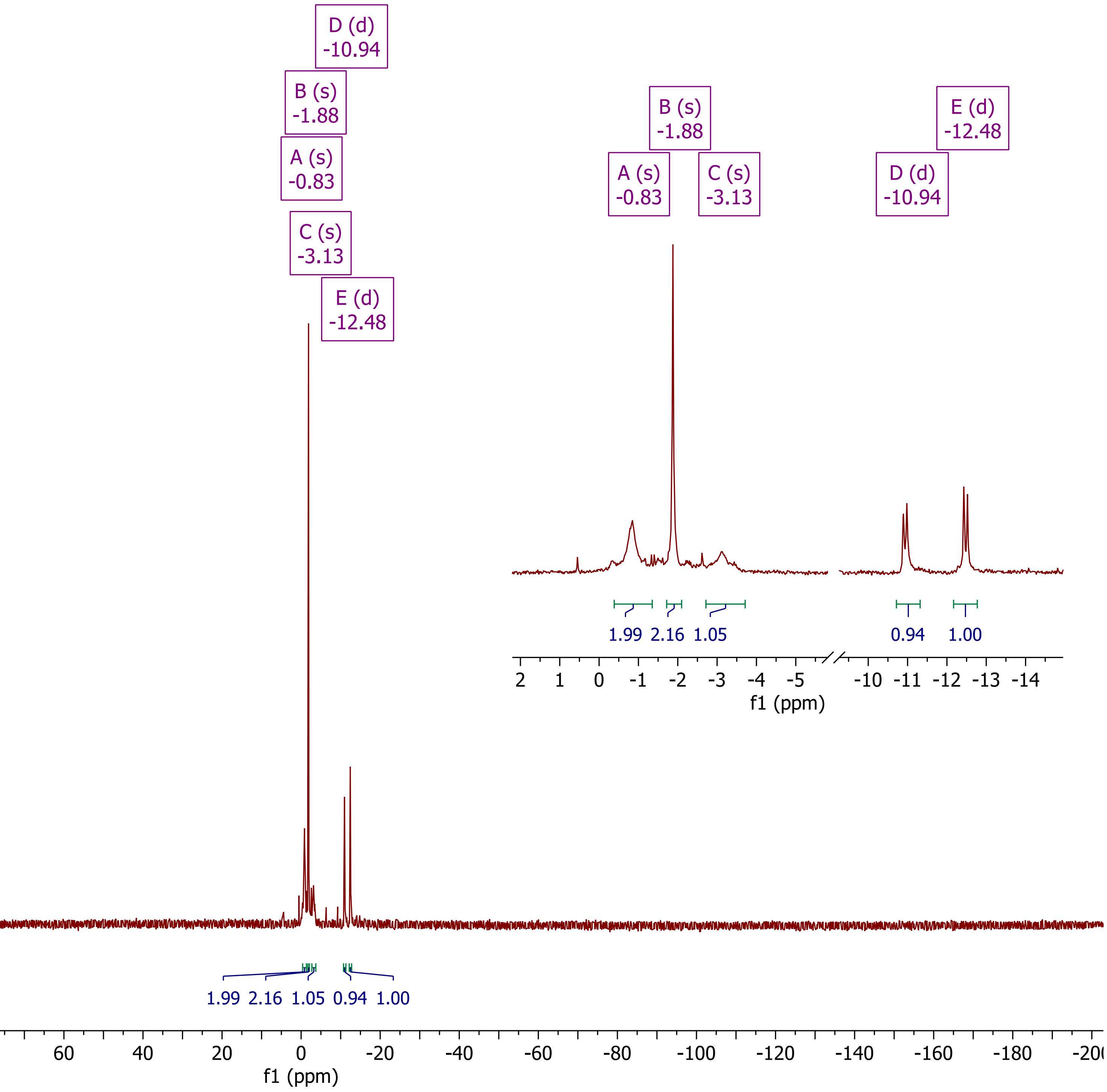

Compound SI-9: $^{18}O_2$ - AB_{11} - PMB - 5-IP7, $^{13}C\{^1H\}$ - NMR (D_2O, 101 MHz)

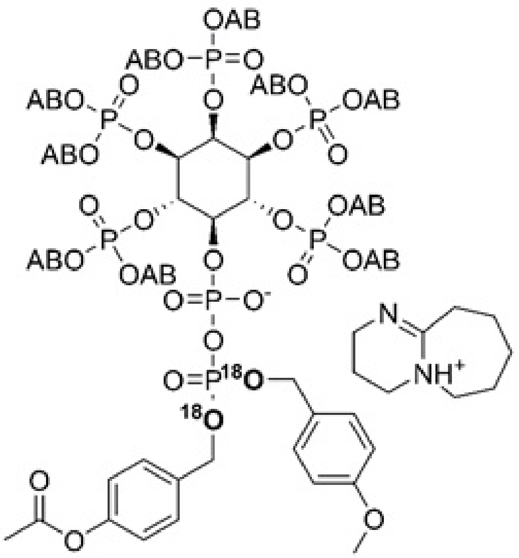

SI-9

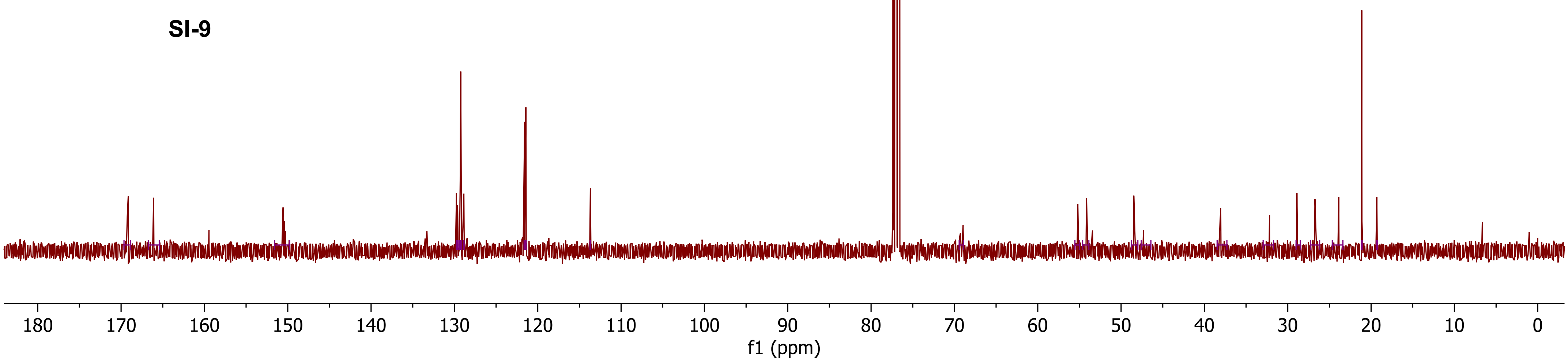

Compound SI-10: $^{18}O_2$ - AB_{11} - 5 - IP7, ^{1}H - NMR (D_2O, 400 MHz)

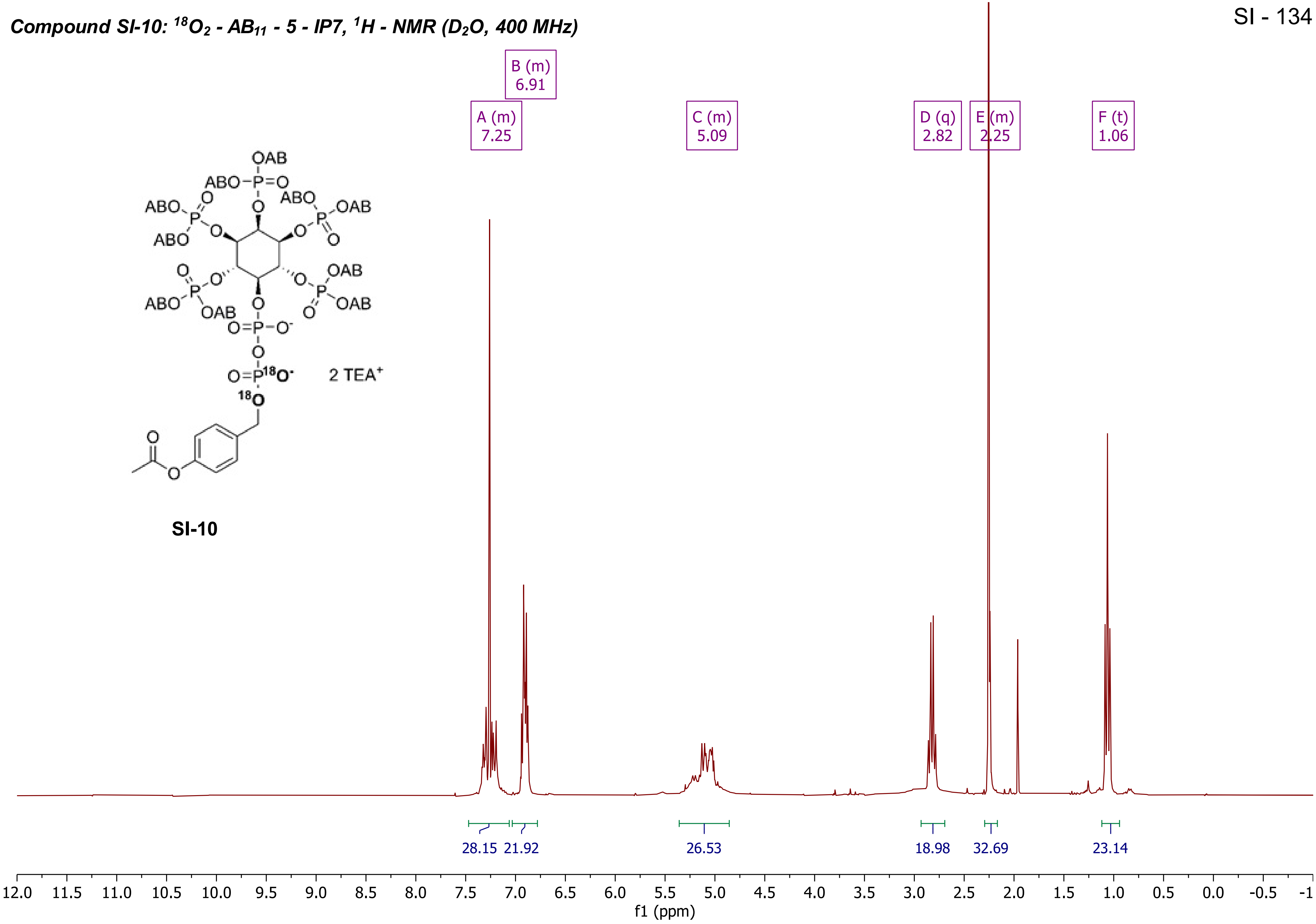

SI-10

Compound SI-10: $^{18}O_2$ - AB_{11} - 5 - IP7, $^{31}P\{^1H\}$ - NMR (D_2O, 162 MHz)

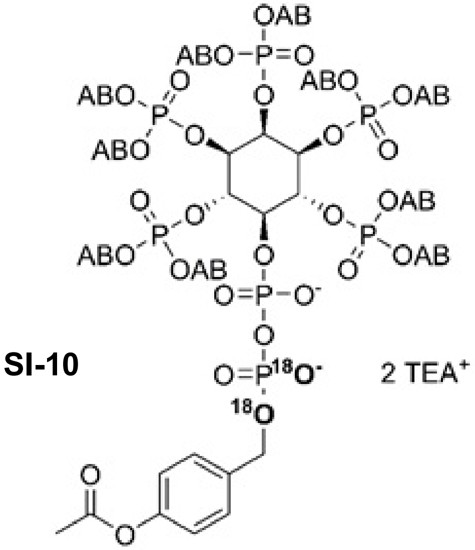

SI-10

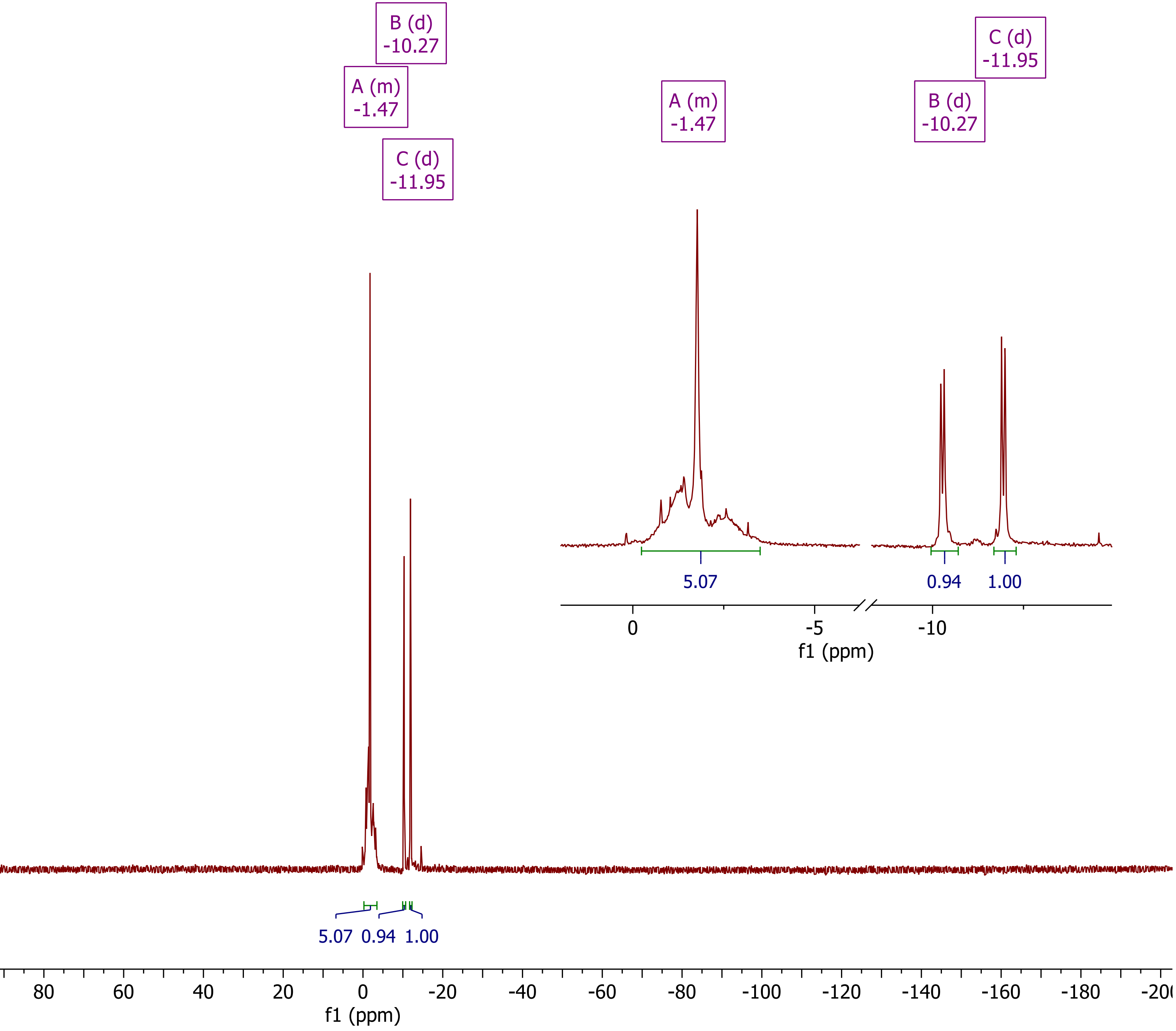

Compound 10: $^{18}O_2$ - AB_{11} - 5 - IP7, $^{13}C\{^1H\}$ - NMR (D_2O, 101 MHz)

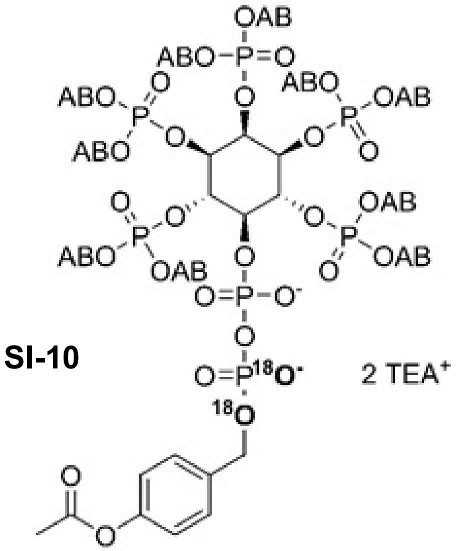

SI-10

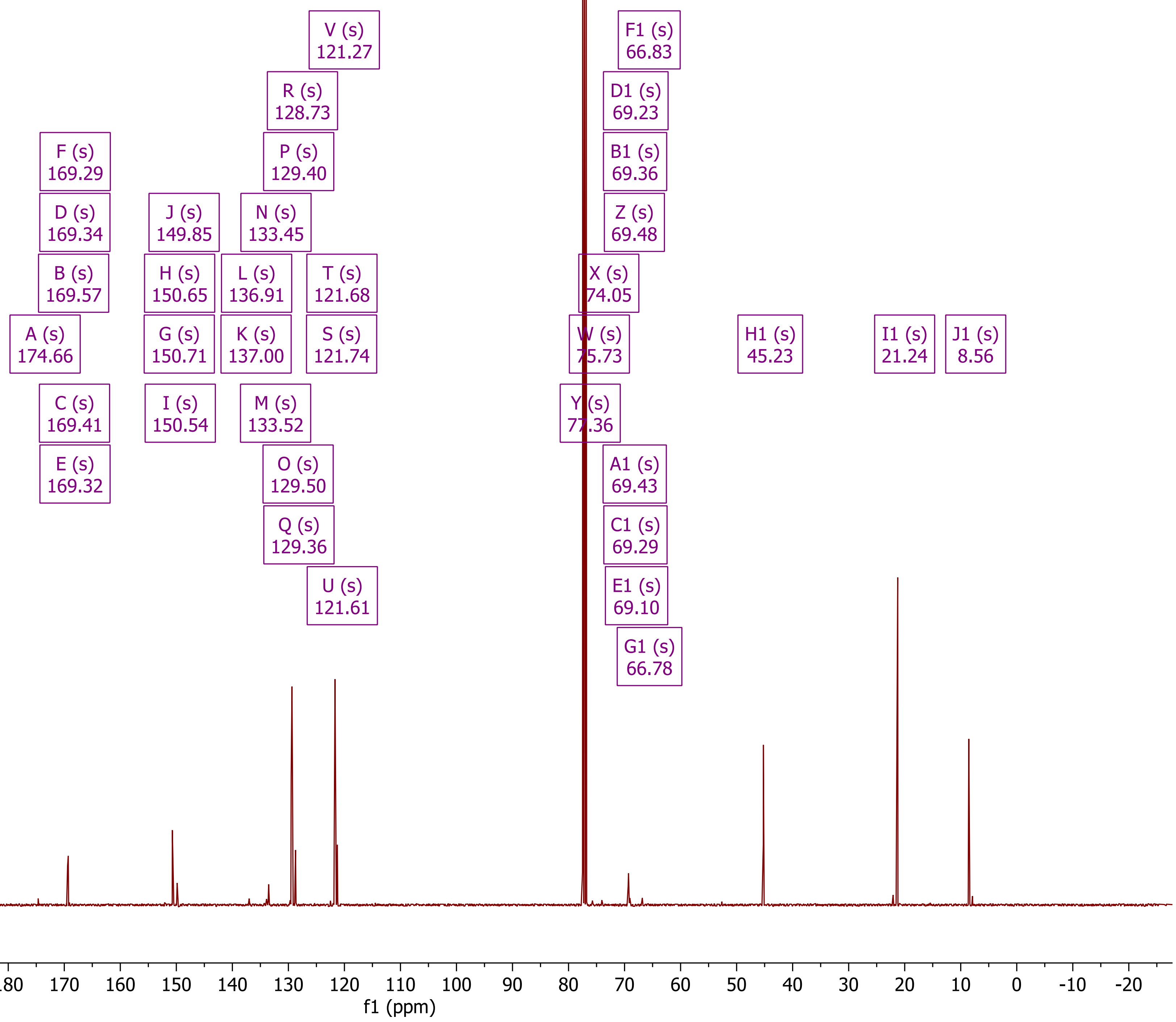

Compound 47: $^{18}O_2$ - 5 - IP7, ^1H - NMR (D_2O, 400 MHz)

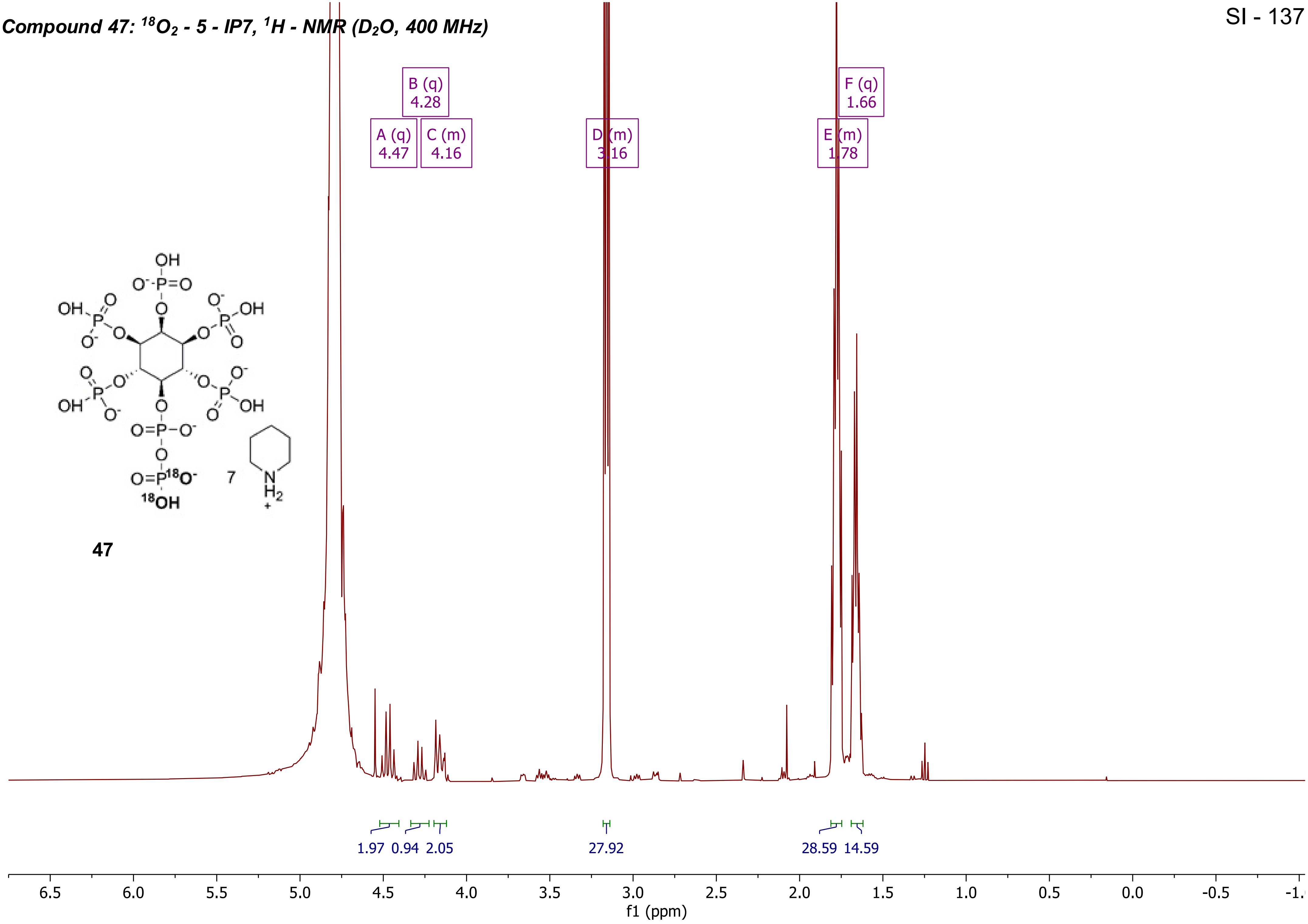

Compound 47: $^{18}O_2$ - 5 - IP7, $^{31}P\{^1H\}$ - NMR (D_2O, 162 MHz)

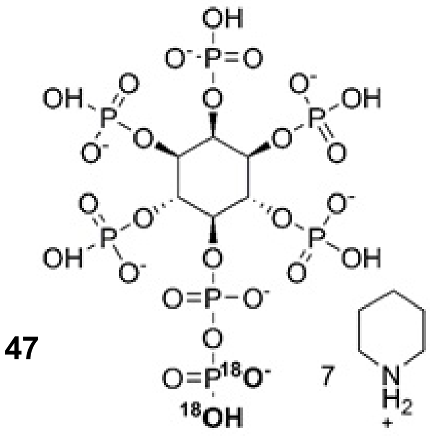

47

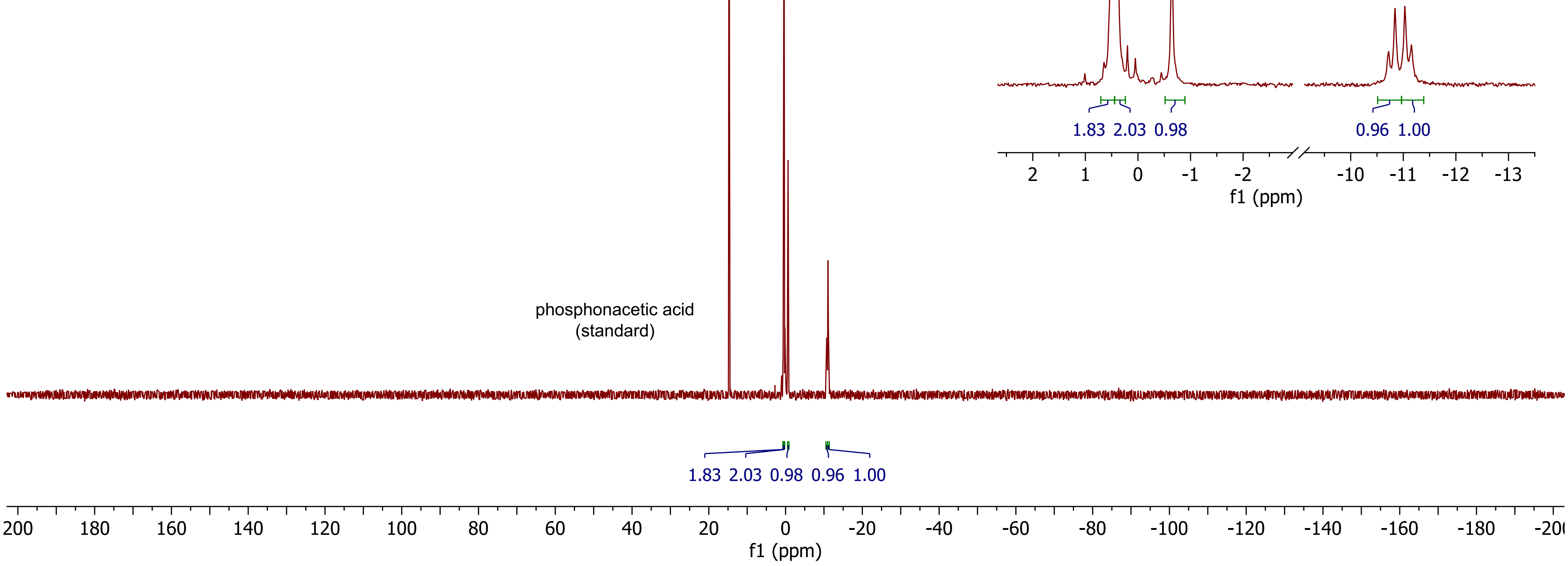

Compound SI-12: $^{18}O_2$ - XEP_5 - Bn_2 - 1 - IP7, ^1H - NMR (D_2O, 400 MHz)

SI-12

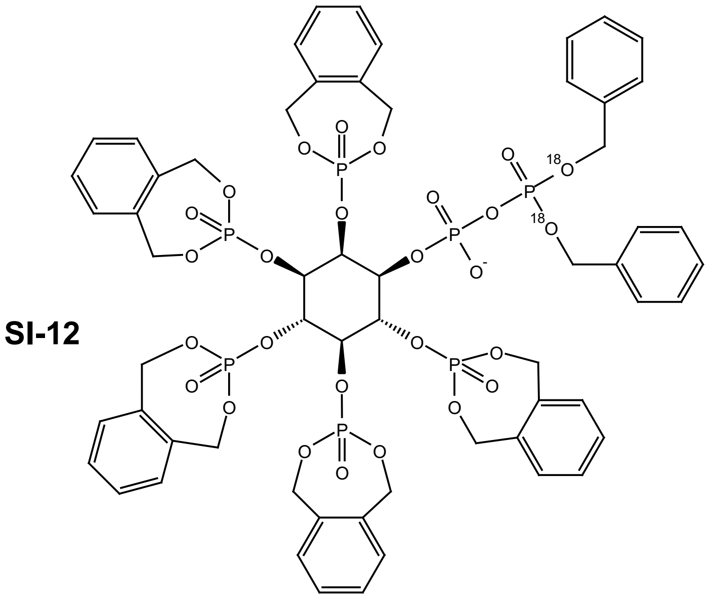

DBU - salt

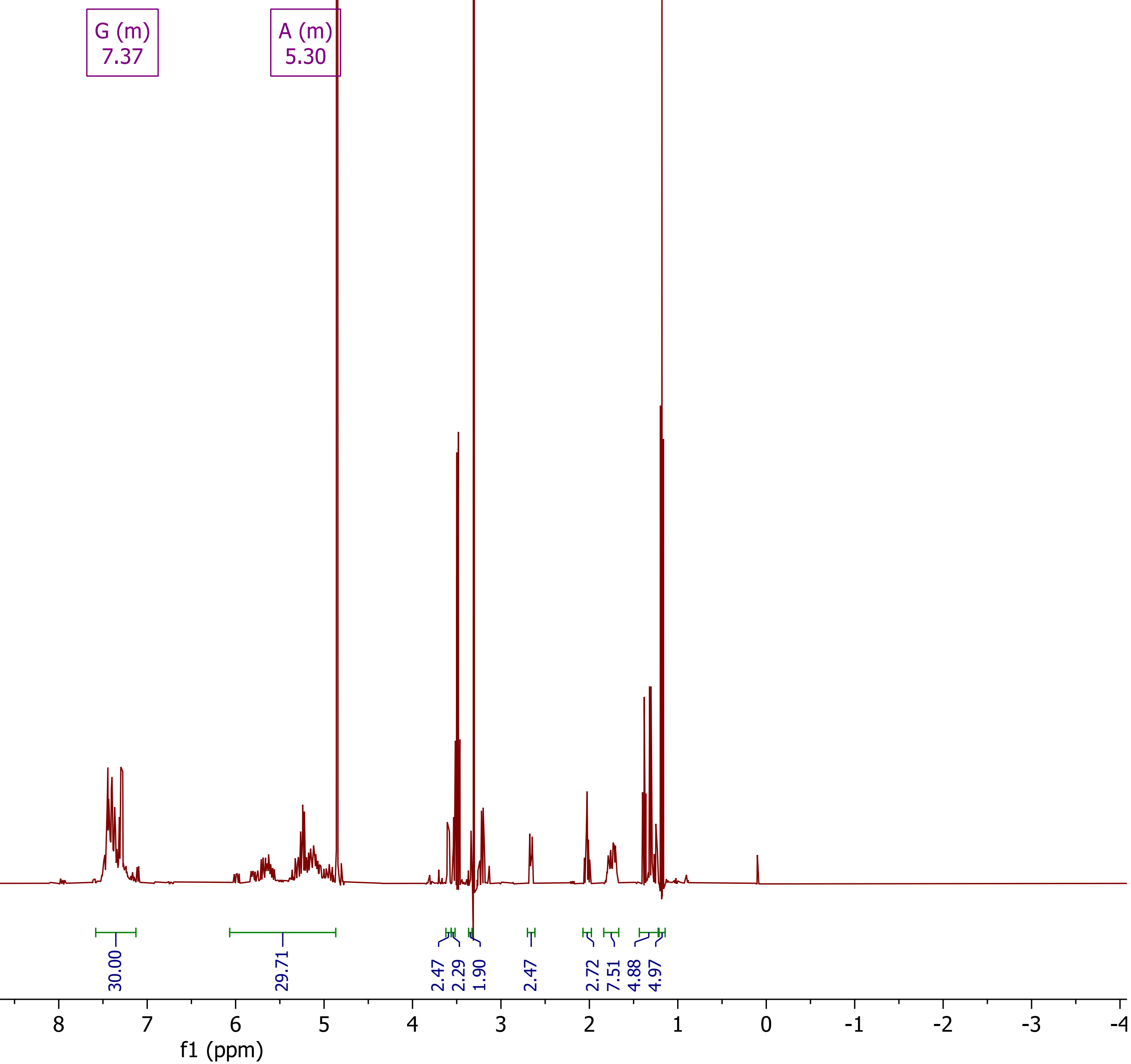

Compound SI-12: $^{18}O_2$ - XEP$_5$ - Bn$_2$ - 1 - IP7, $^{31}P\{^1H\}$ - NMR (D_2O, 162 MHz)

SI-12

DBU - salt

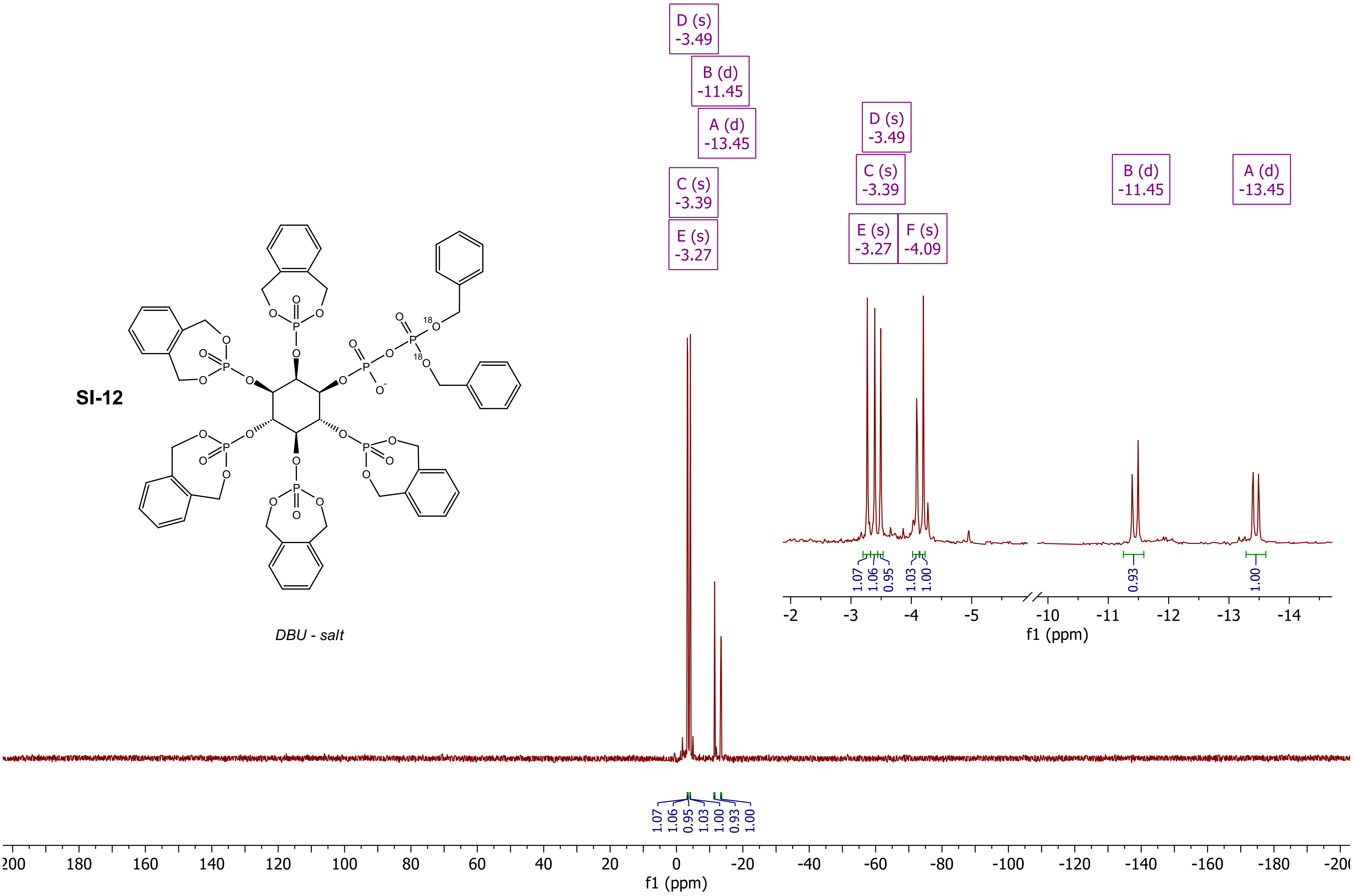

Compound SI-13: $^{18}O_2$ - 1 - IP7, $^{31}P\{^1H\}$ - NMR (D_2O, 162 MHz)

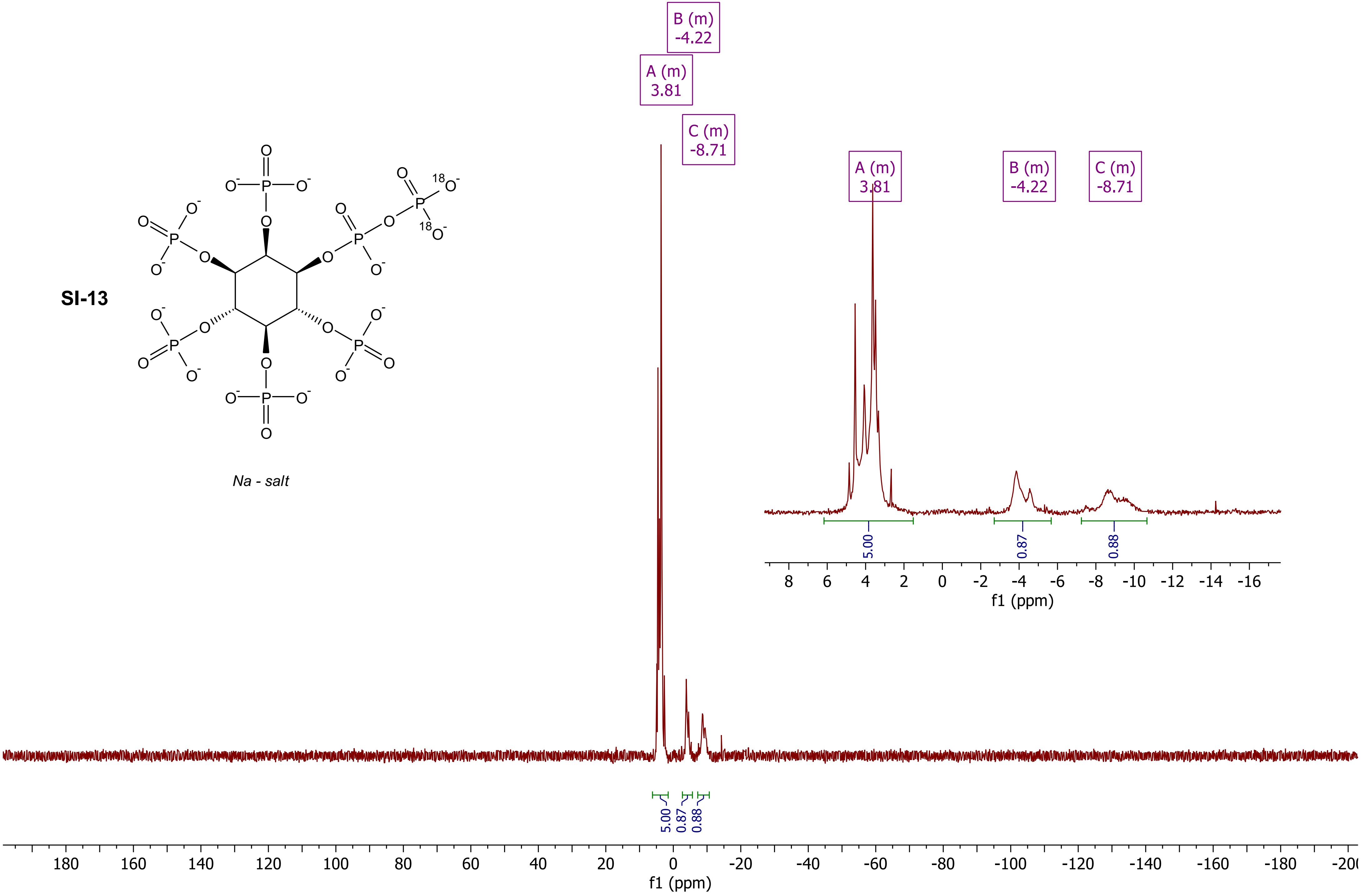

HRMS (ESI) Analysis of compound 11: 4-(1,3-dioxolan-2-yl)phenol

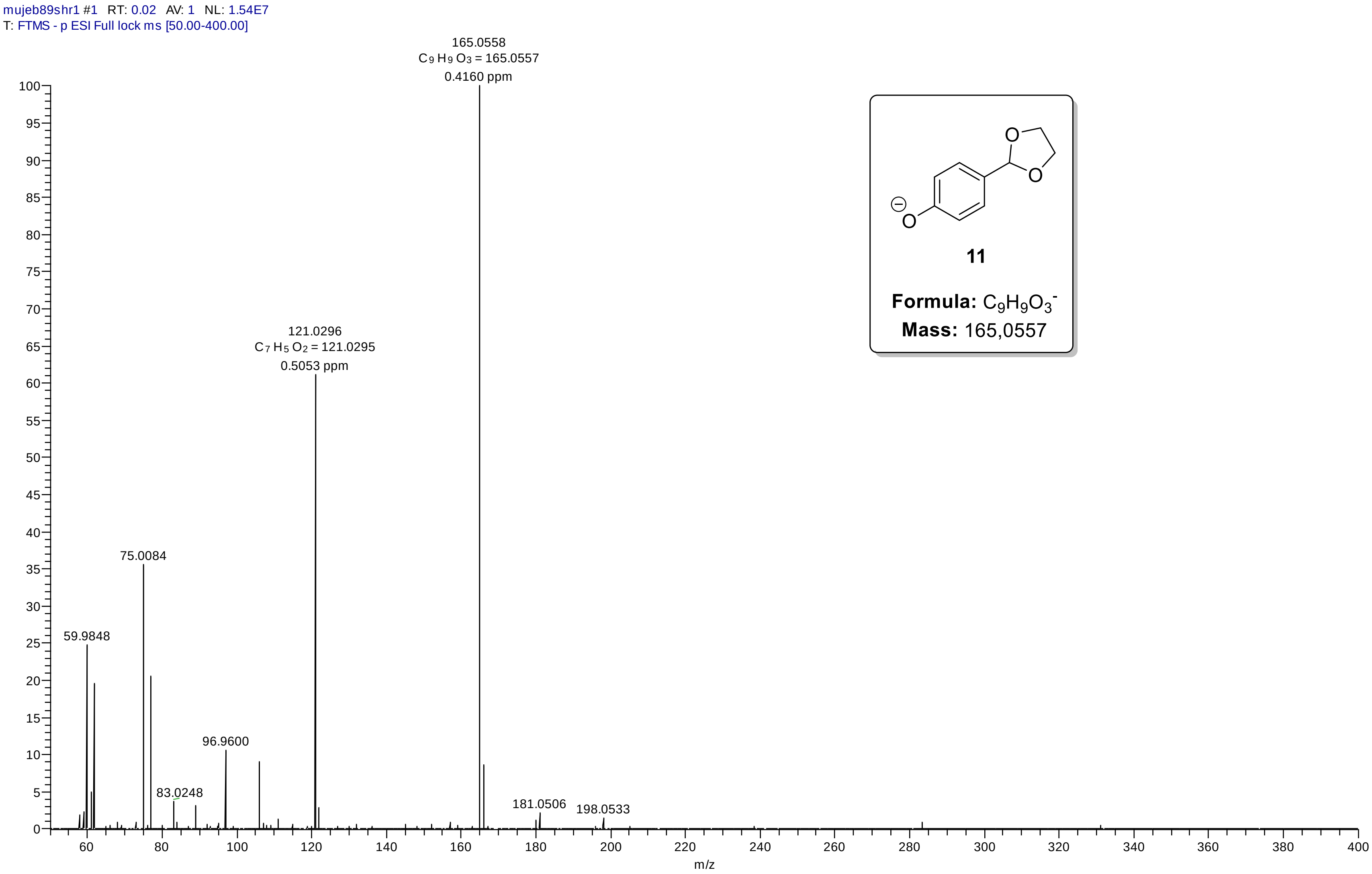

11

Formula: $C_9H_9O_3^-$

Mass: 165,0557

HRMS (ESI) Analysis of compound 16: ^{18}O-4-((hydroxyl)methyl)phenol

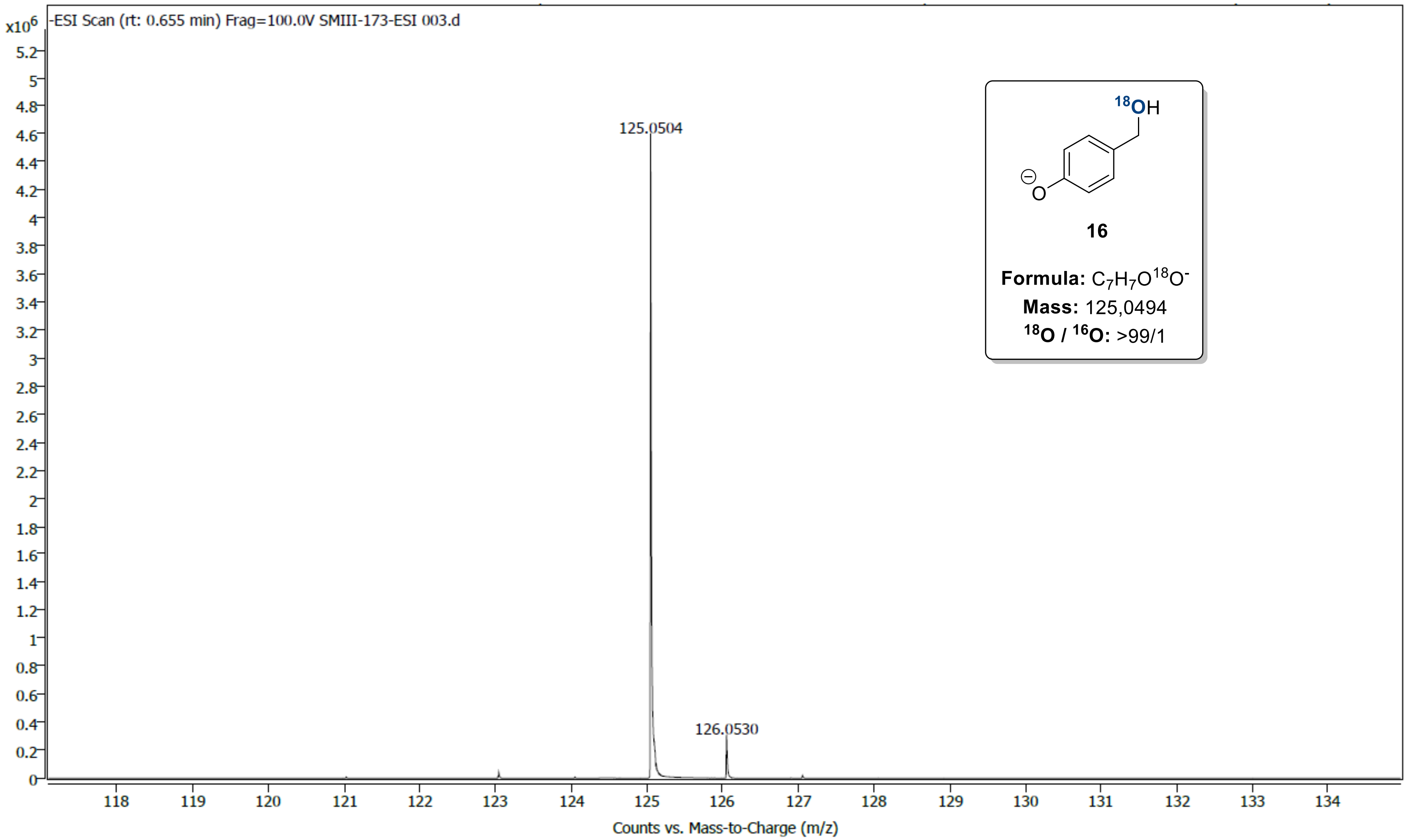

HRMS (ESI) Analysis of compound 17: ^{18}O-4-((hydroxyl)methyl)phenyl acetate

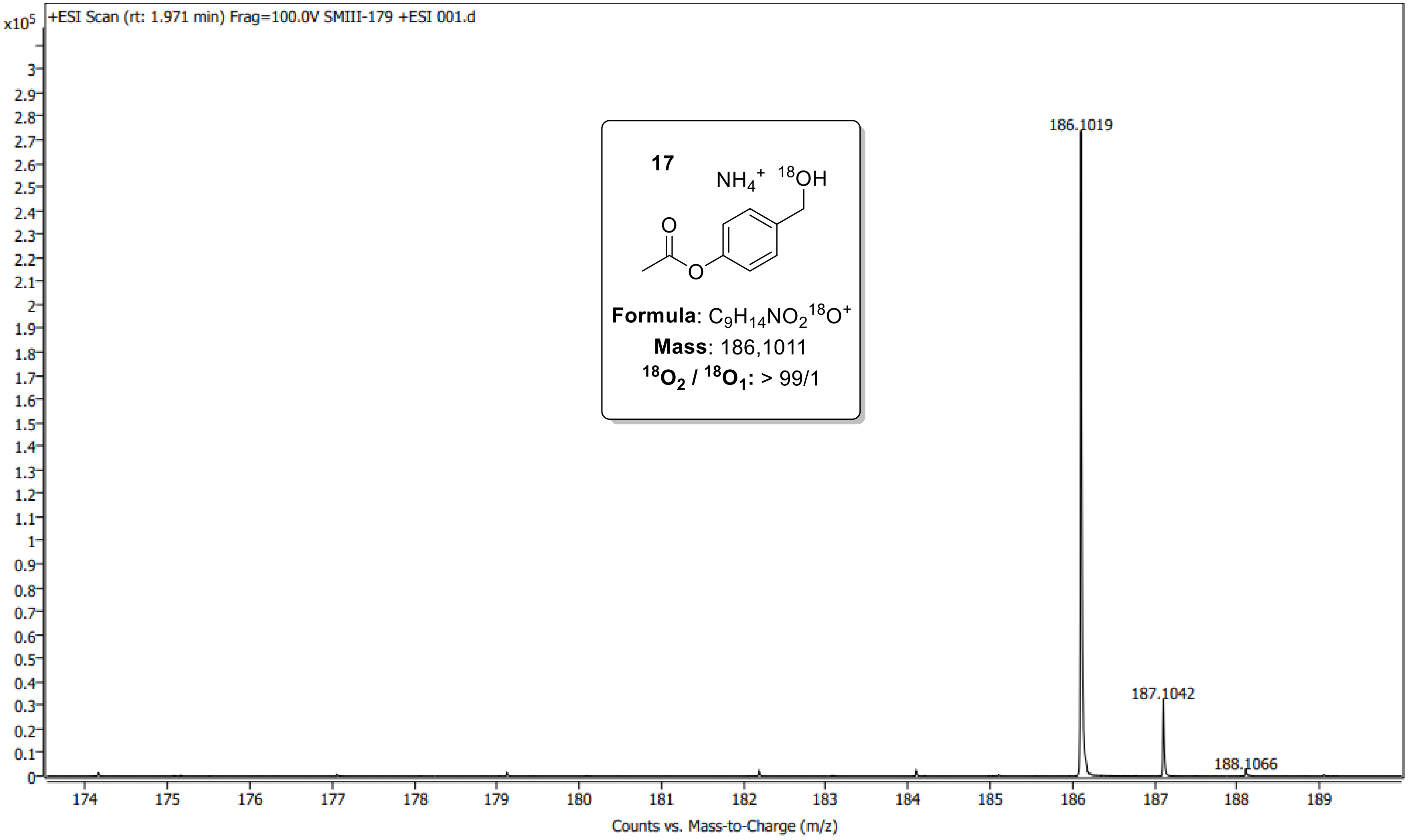

HRMS (ESI) Analysis of compound 18: ^{18}O-(4-methoxyphenyl)methanol

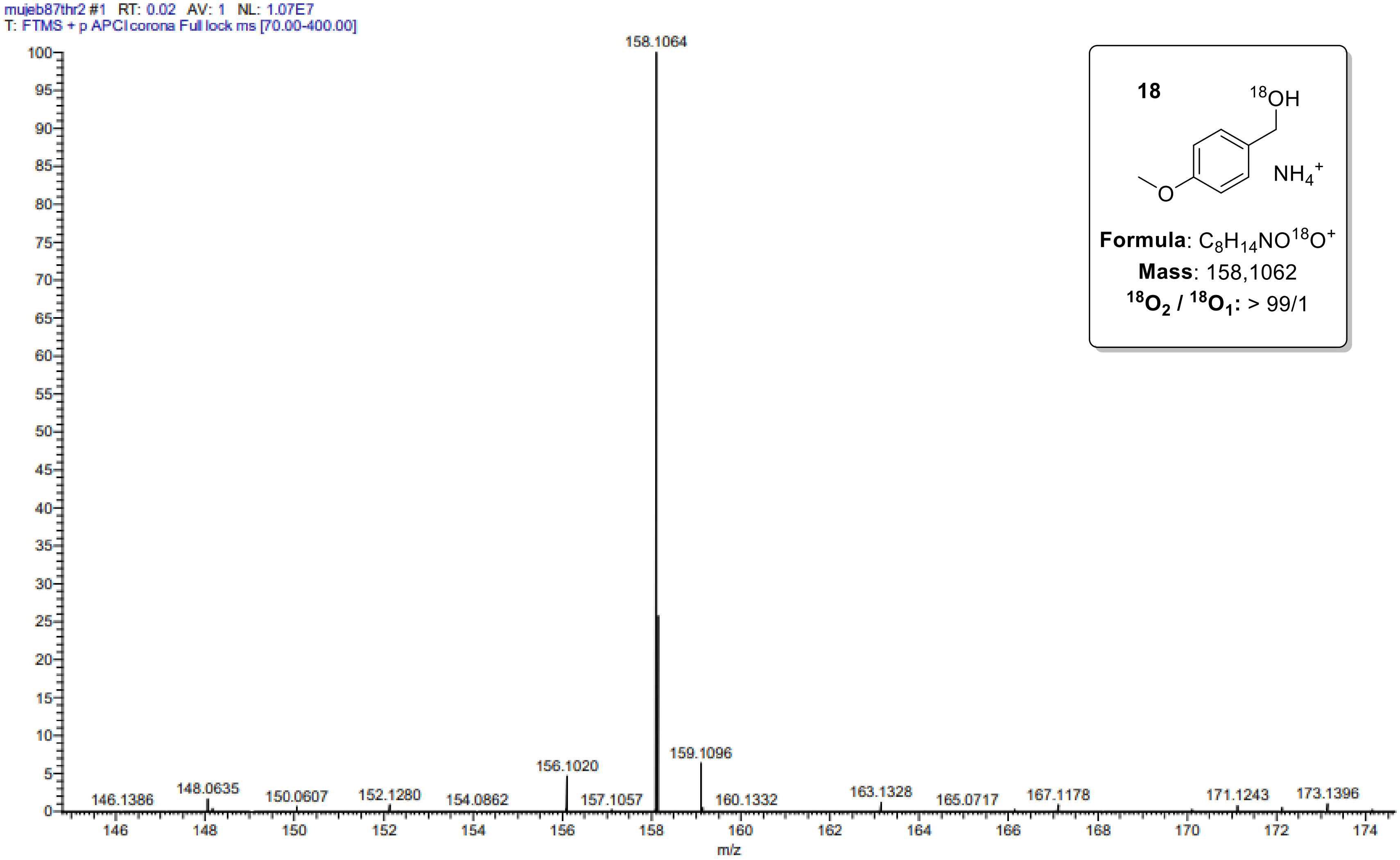

HRMS (ESI) Analysis of compound SI-2: 4-(TIPSO)butanoate

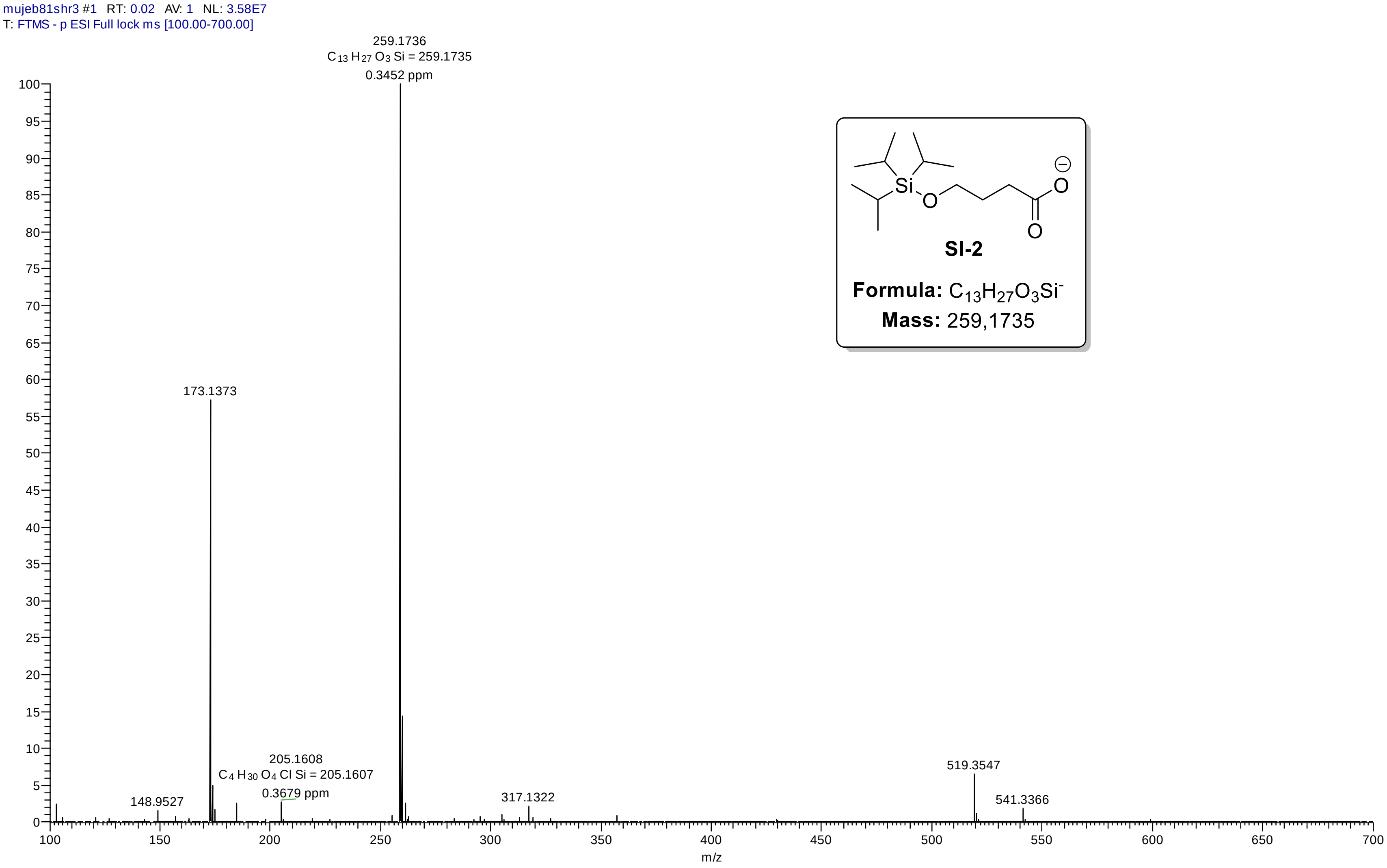

HRMS (ESI) Analysis of compound 19: ^{18}O-4-((hydroxyl)methyl)phenyl 4-((triisopropylsilyl)oxy)butanoate

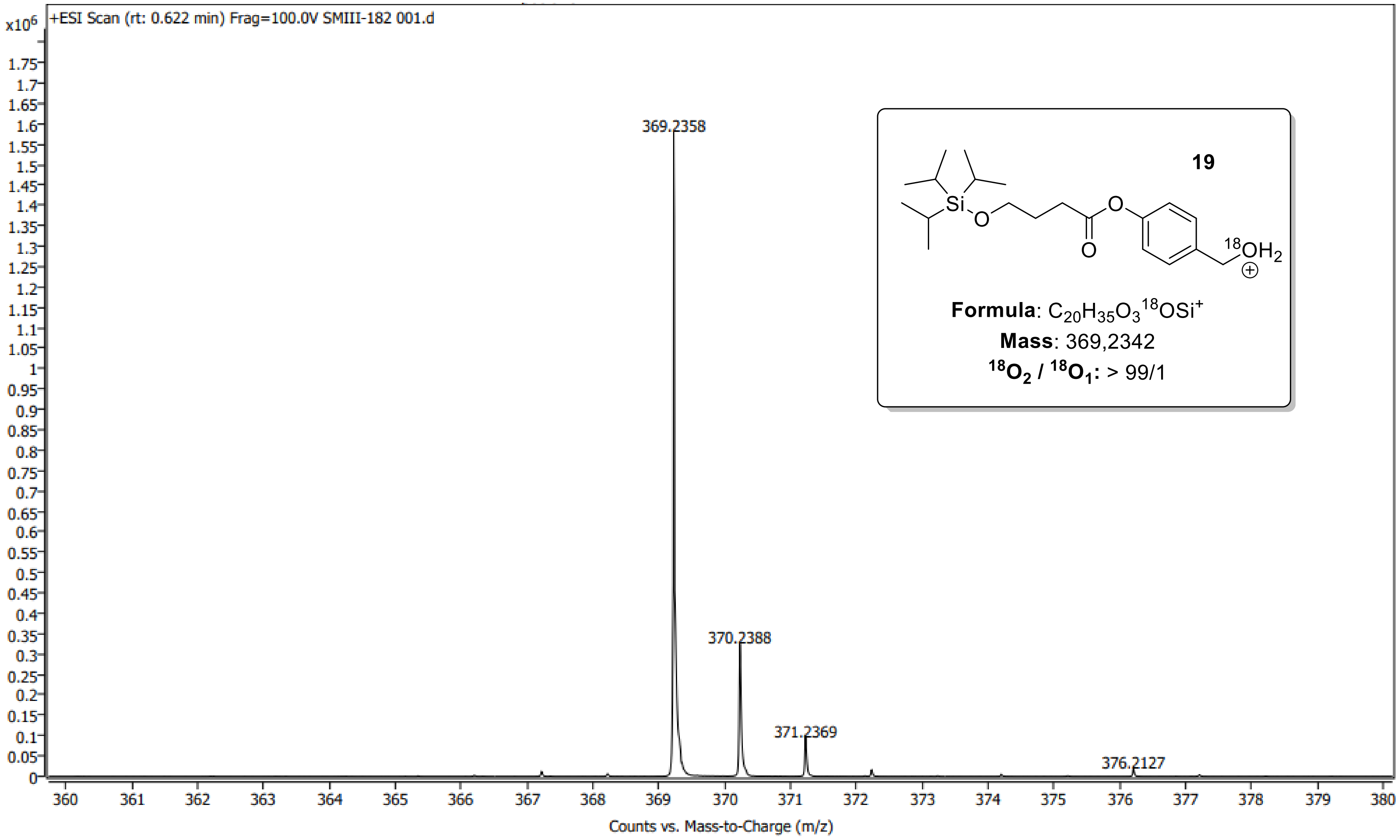

HRMS (ESI) Analysis of compound 20: ^{18}O-4-((hydroxyl)methyl)phenyl 5-(Fmoc-amino)pentanoate (BigFmAlkohol)

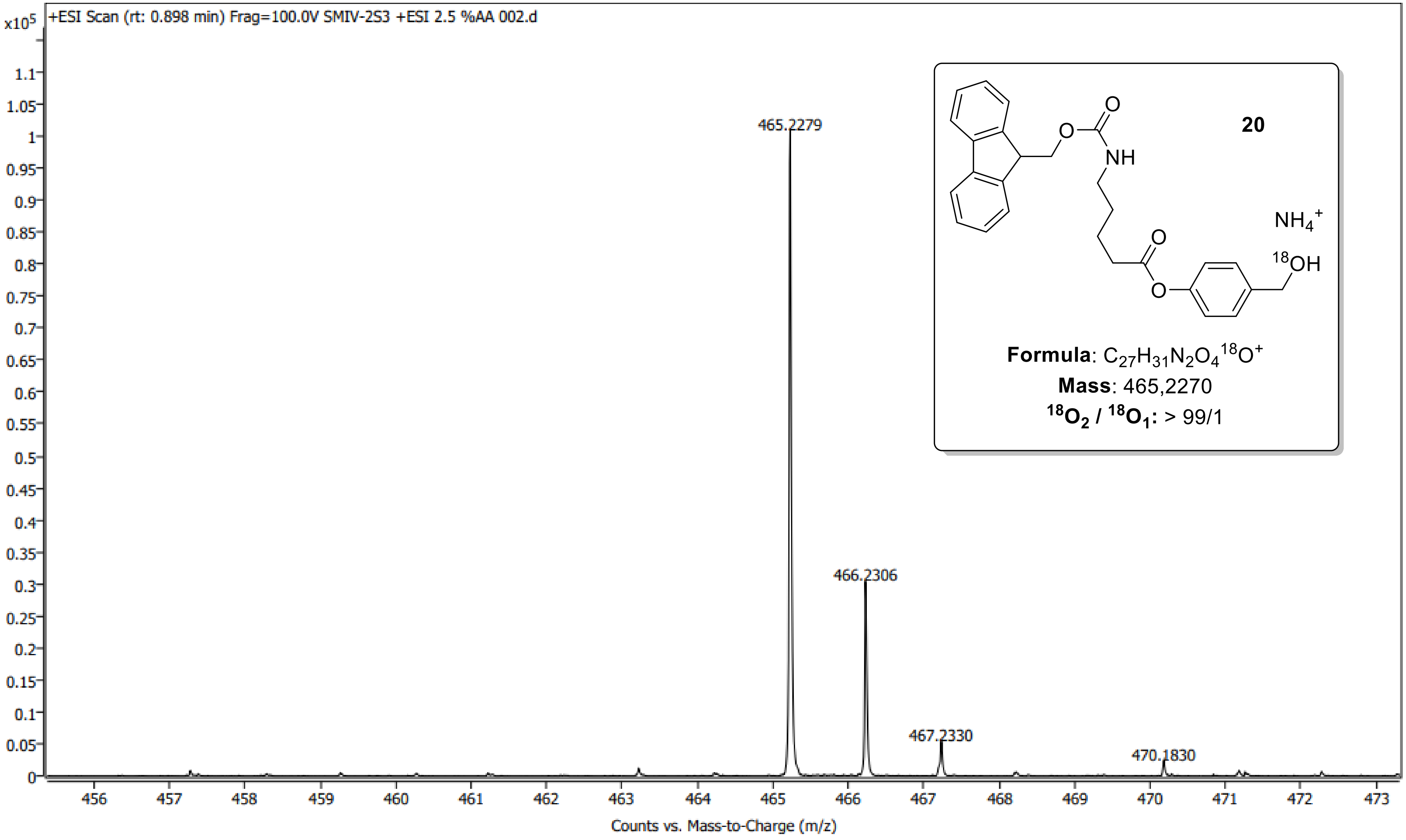

HRMS (ESI) Analysis of compound 27: $^{18}O_2$-((((diisopropylamino)phosphanediyl)bis(methylene))bis(4,1-phenylene) diacetate ($^{18}O_2$ - AB-Amidite)

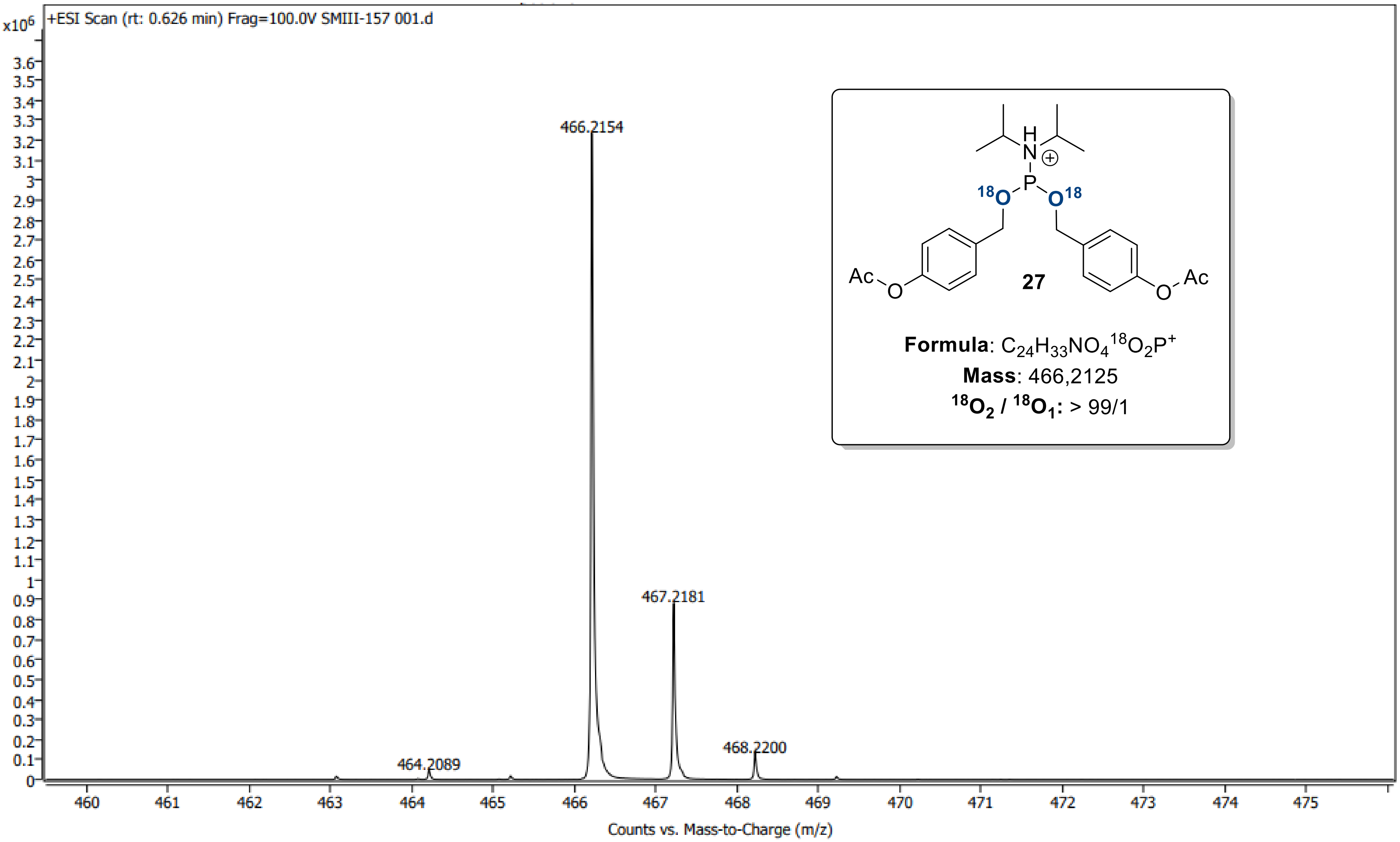

HRMS (ESI) Analysis of compound 28: $^{18}O_2$-bis(4-methoxybenzyl)diisopropylphosphoramidite ($^{18}O_2$ - PMB-Amidite)

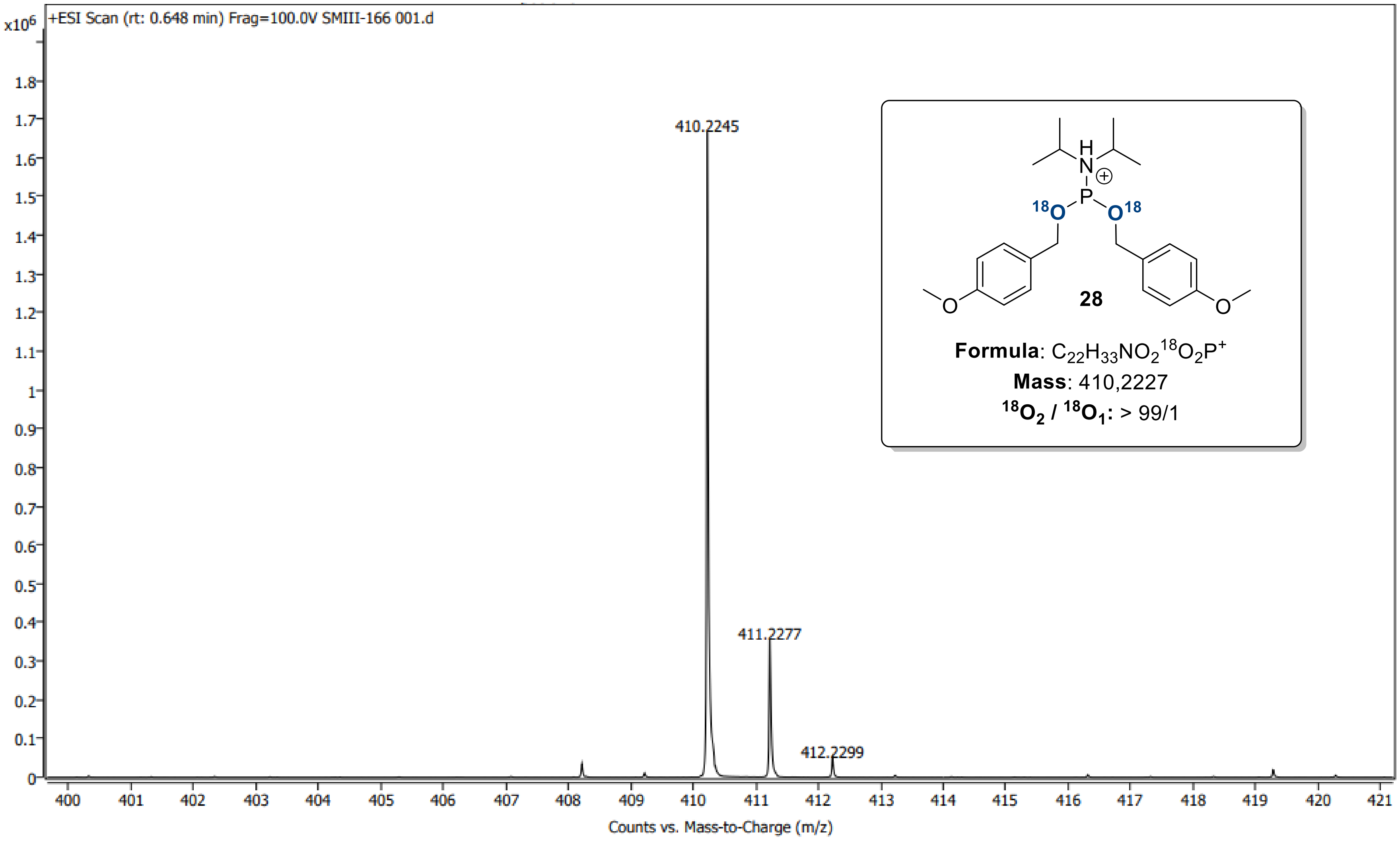

HRMS (ESI) Analysis of compound 31: ((((diisopropylamino)phosphanediyl)bis(oxy-^{18}O))bis(methylene))bis(4,1-phenylene) bis(4-((triisopropylsilyl)oxy)butanoate)

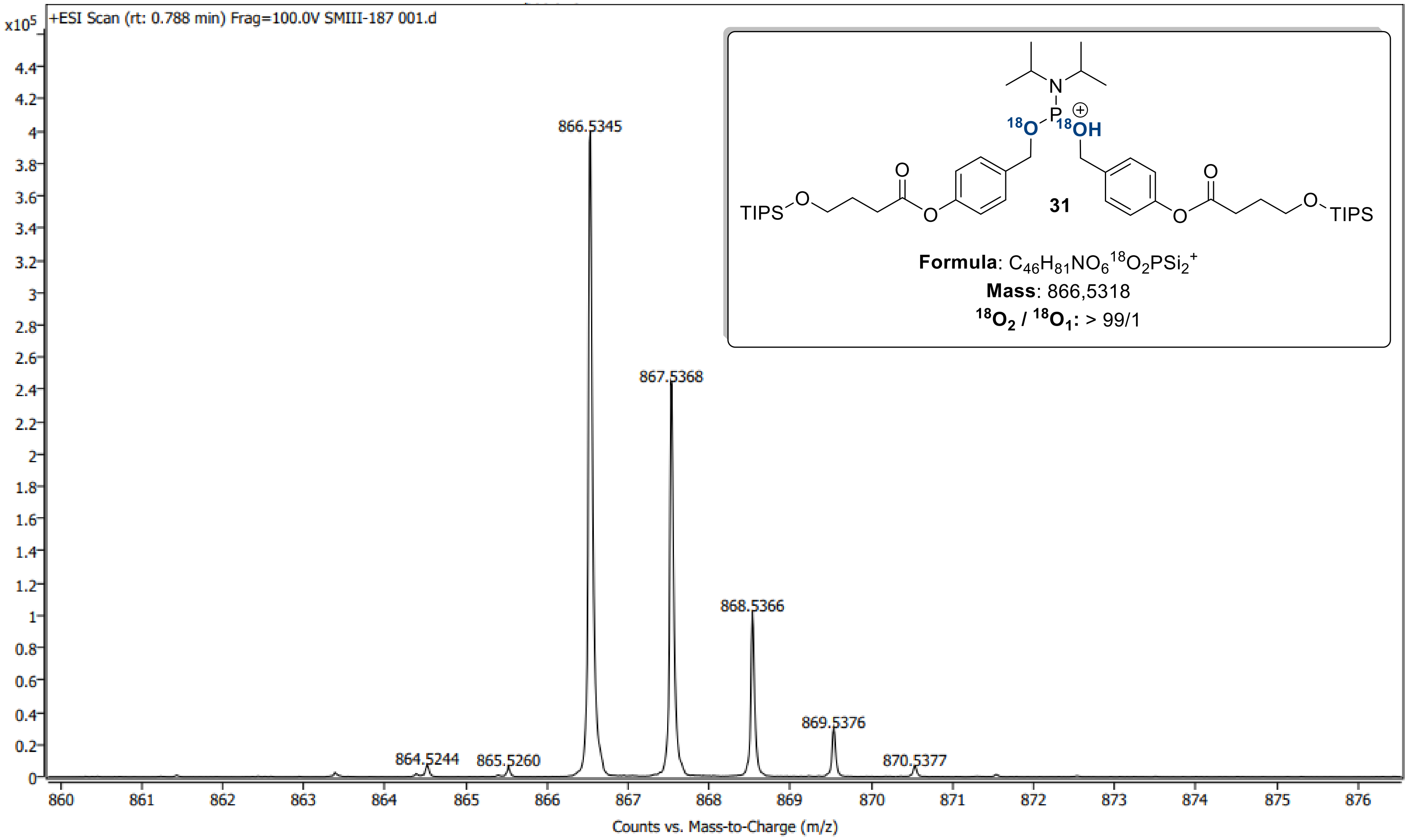

HRMS (ESI) Analysis of compound 32: ((((diisopropylamino)phosphanediyl)bis(oxy-18O))bis(methylene))bis(4,1-phenylene) bis(5-((((9H-fluoren-9-yl)methoxy)carbonyl)amino)pentanoate) (32)

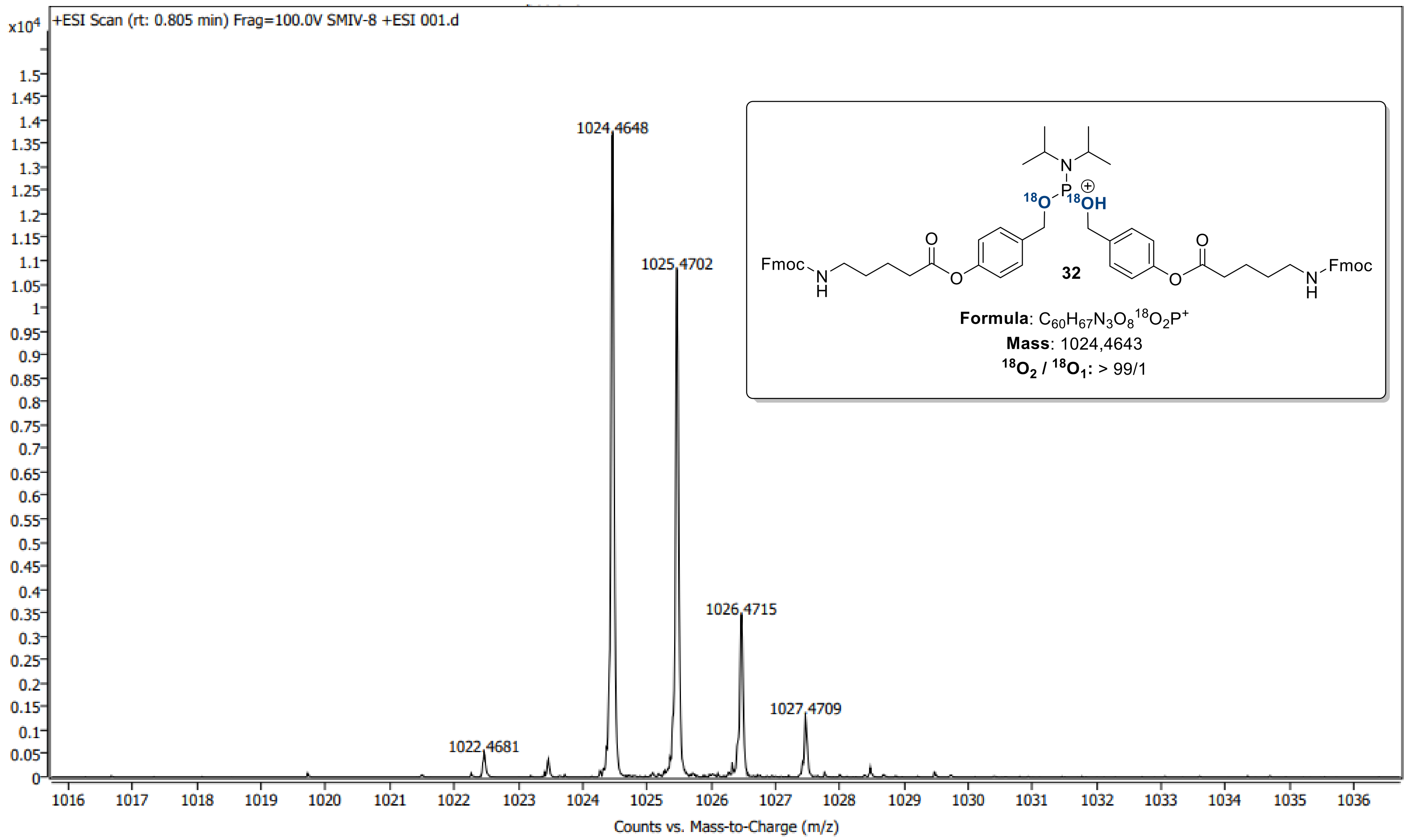

HRMS (ESI) Analysis of compound 29: 4-(((bis(diisopropylamino)phosphaneyl)oxy)methyl)phenyl acetate

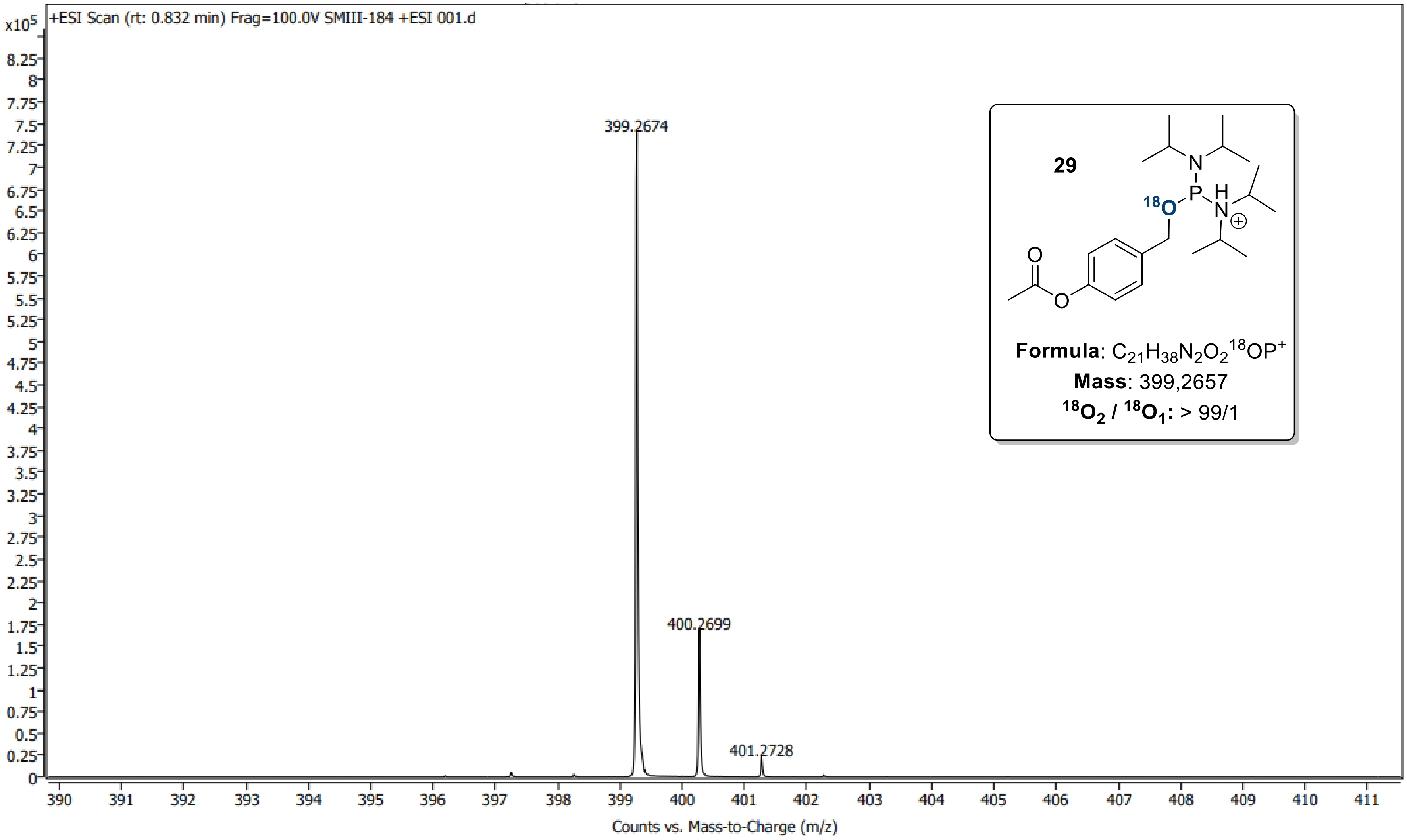

HRMS (ESI) Analysis of compound 30: 4-(((diisopropylamino)((4-methoxybenzyl)oxy-^{18}O)phosphaneyl)oxy)methyl)phenyl acetate

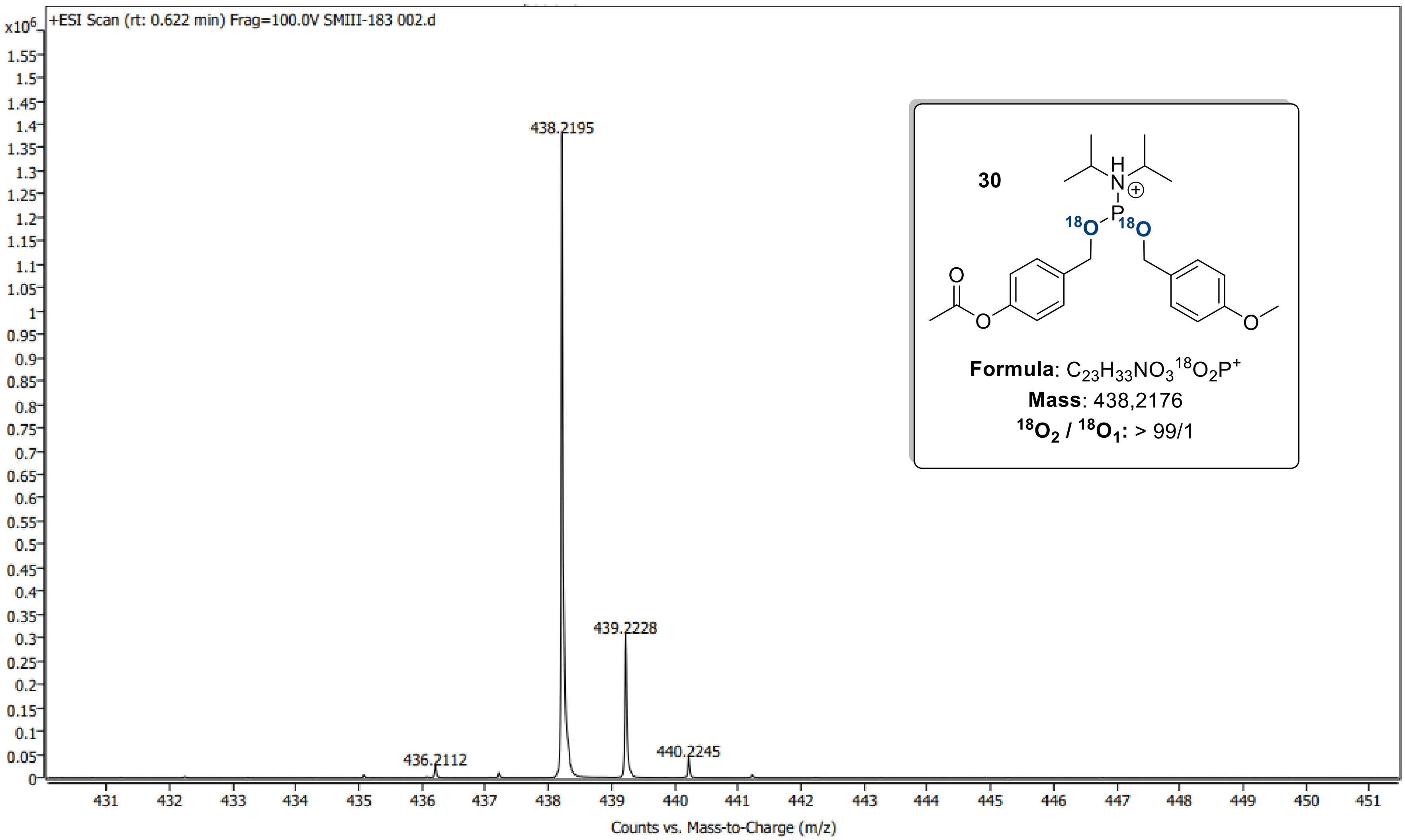

HRMS (ESI) Analysis of compound 33: ^{18}O-DNA-precursor P-Amidite

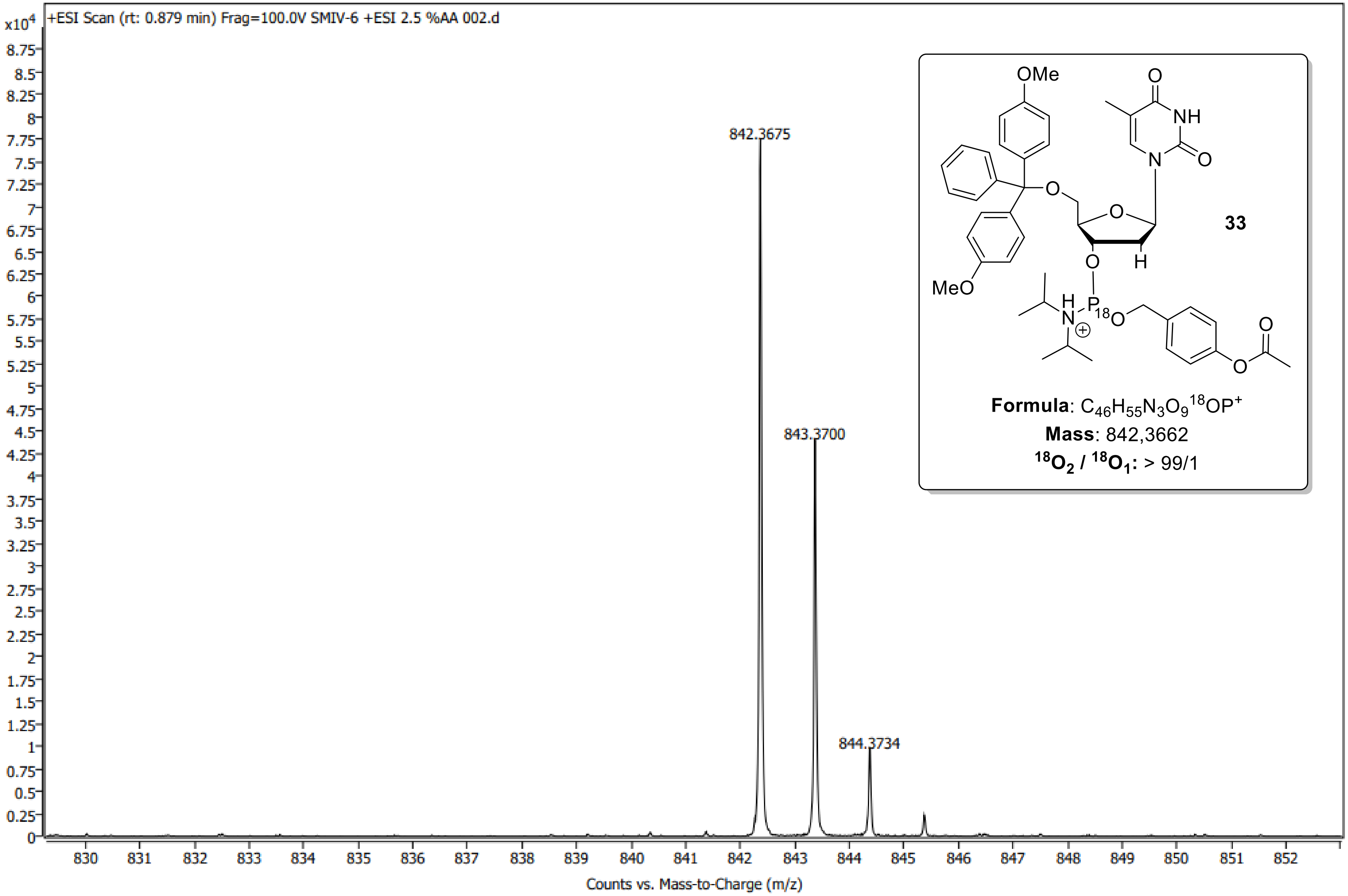

HRMS (ESI) Analysis of compound SI-3: ^{16}O-DNA-precursor P-Amidite

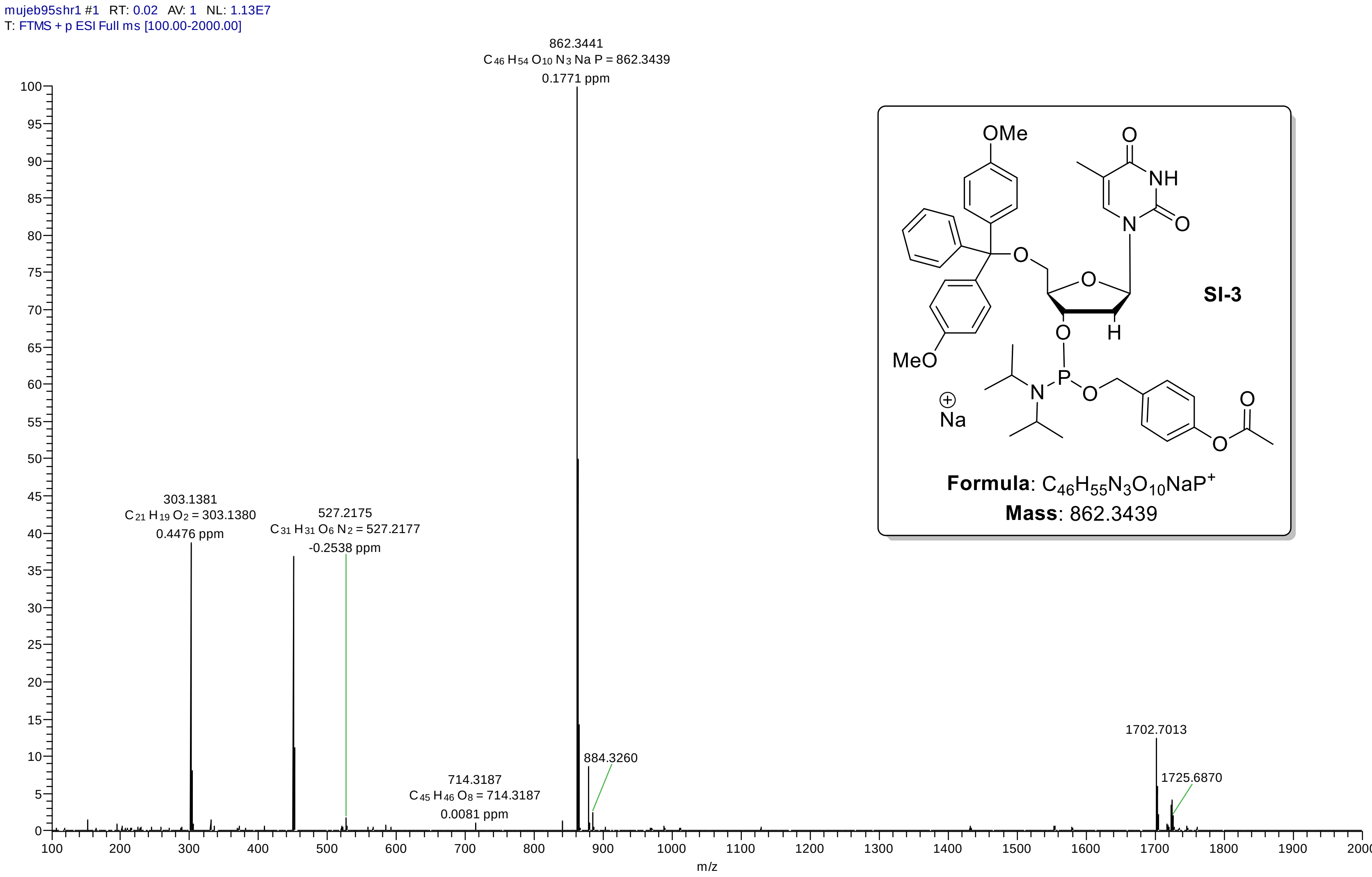

HRMS (ESI) Analysis of compound 34: $^{18}O_2$-Adenosine-5'-monophosphate ($^{18}O_2$-AMP, 34)

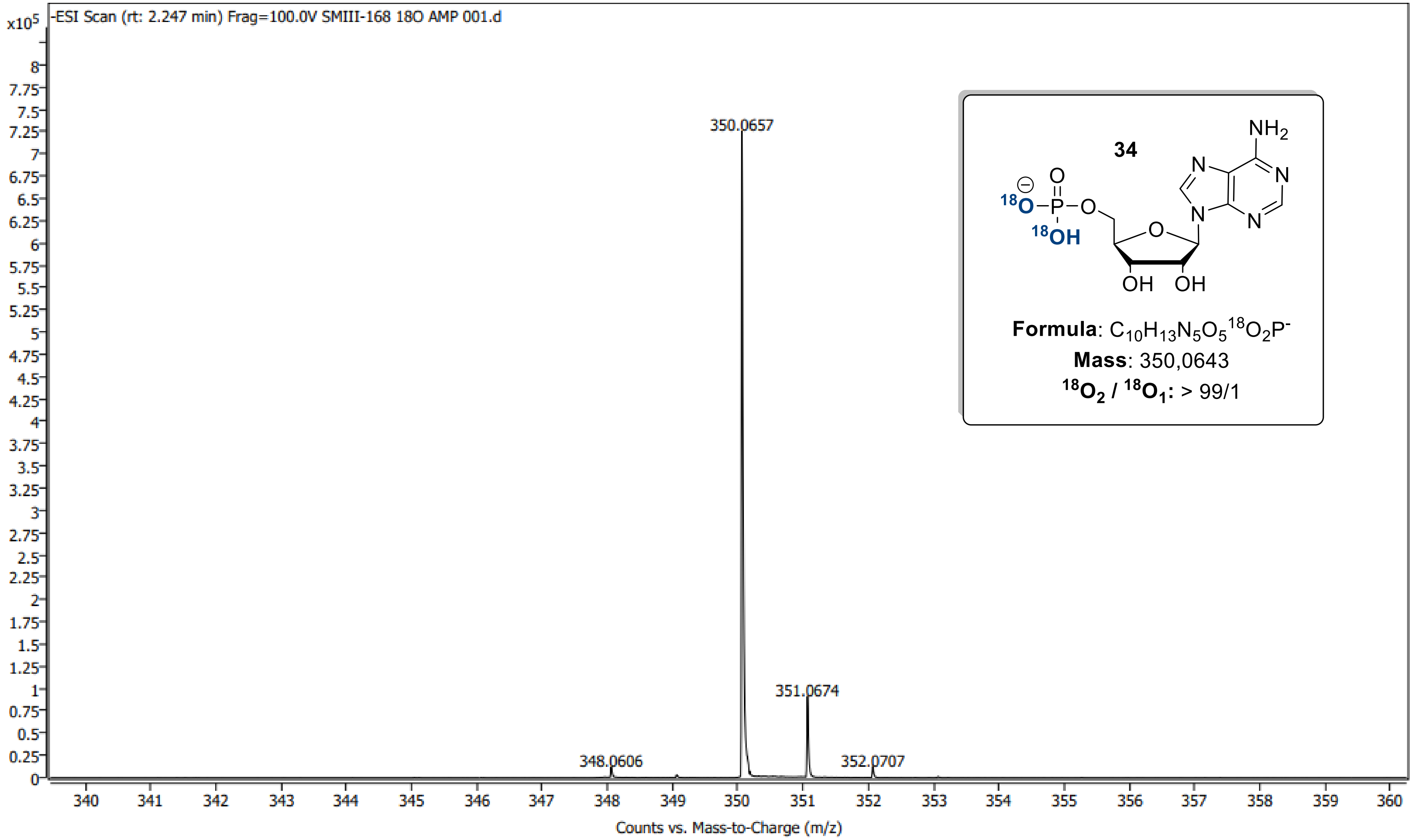

HRMS (ESI) Analysis of compound 6: $^{18}O_2$-Guanosine-5'-monophosphate ($^{18}O_2$-GMP)

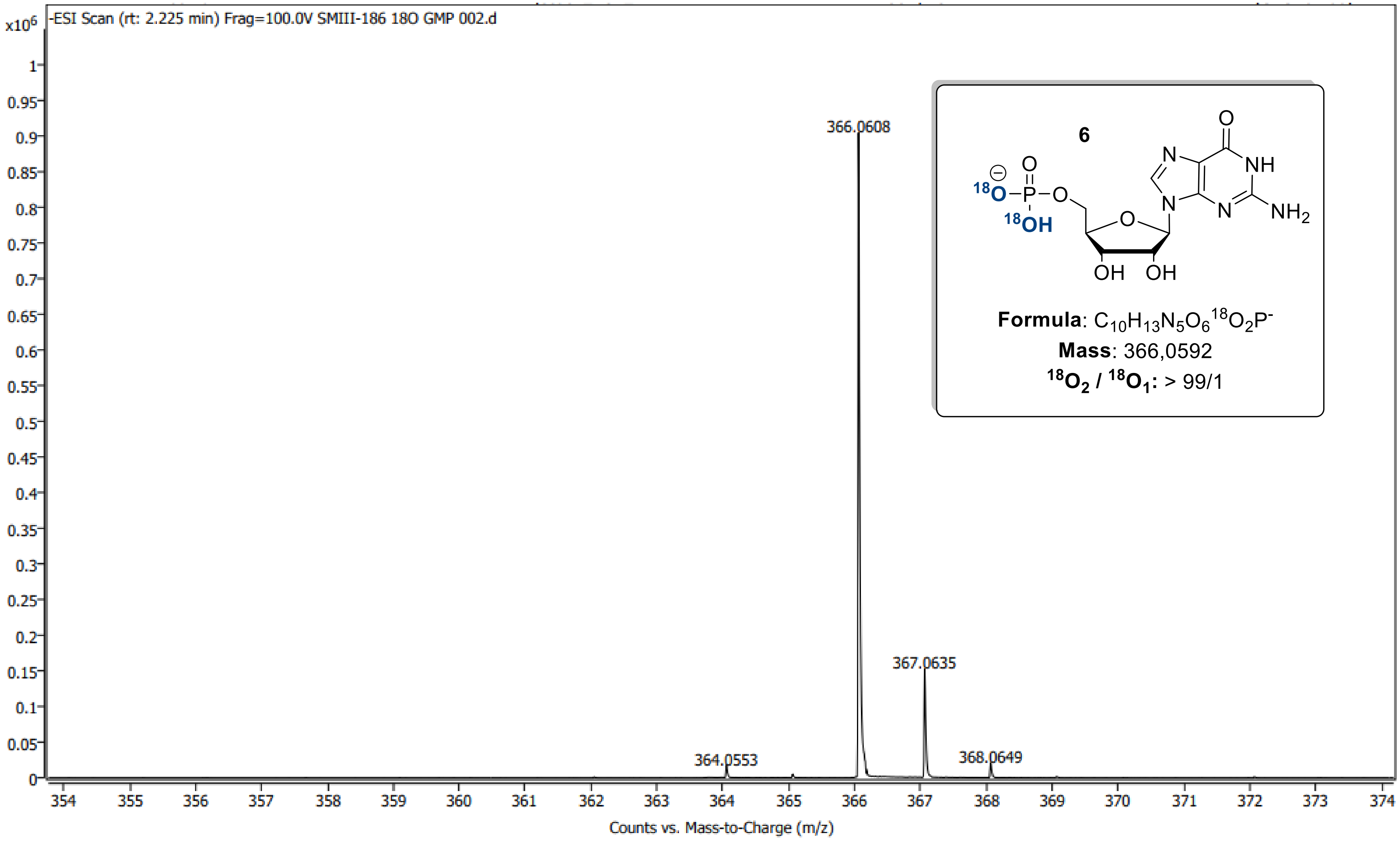

HRMS (ESI) Analysis of compound 35: β-$^{18}O_2$-Adenosine-5'-diphosphate (β-$^{18}O_2$-ADP)

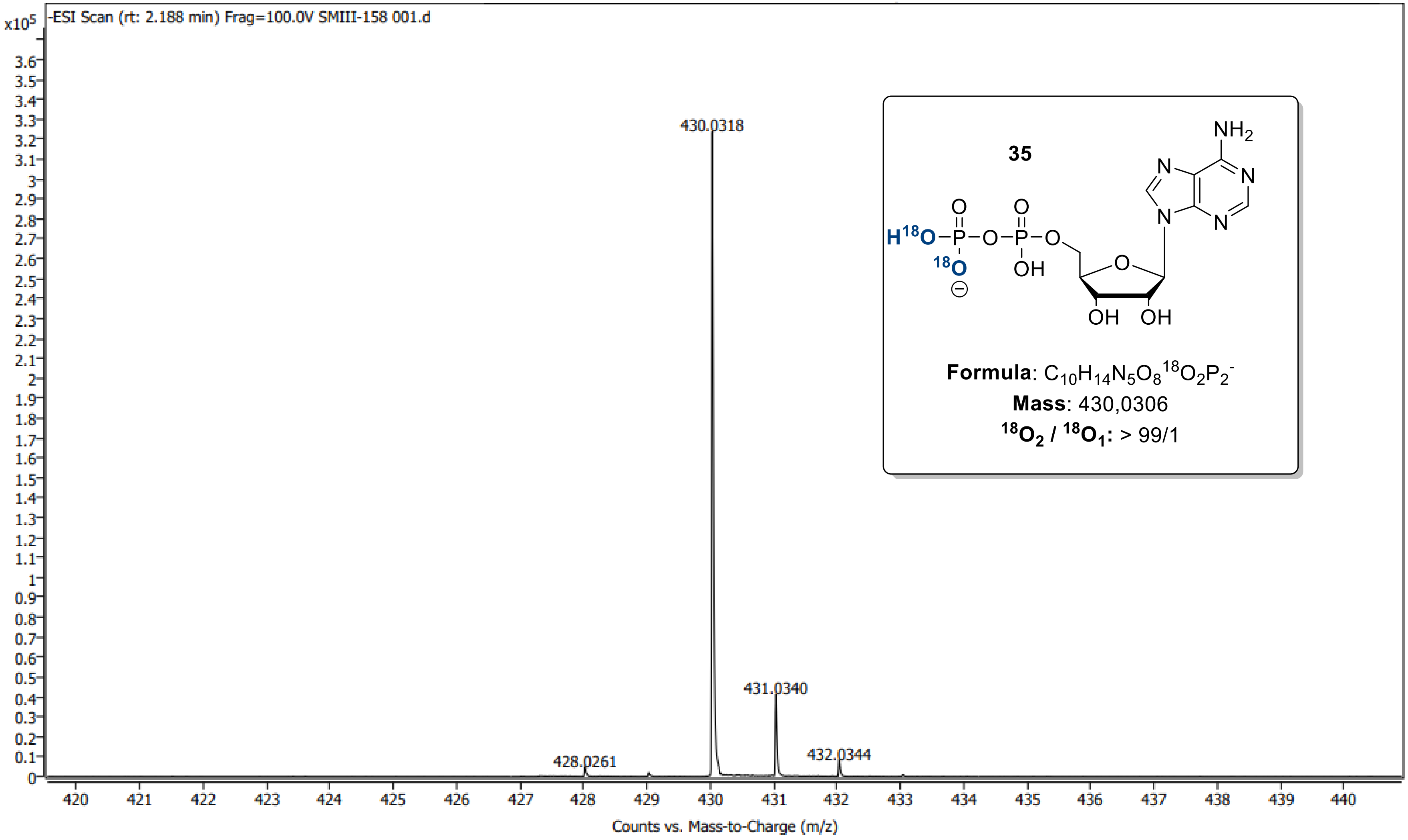

HRMS (ESI) Analysis of compound 36: γ-$^{18}O_2$-Adenosine-5'-triphosphate (γ-$^{18}O_2$-ATP)

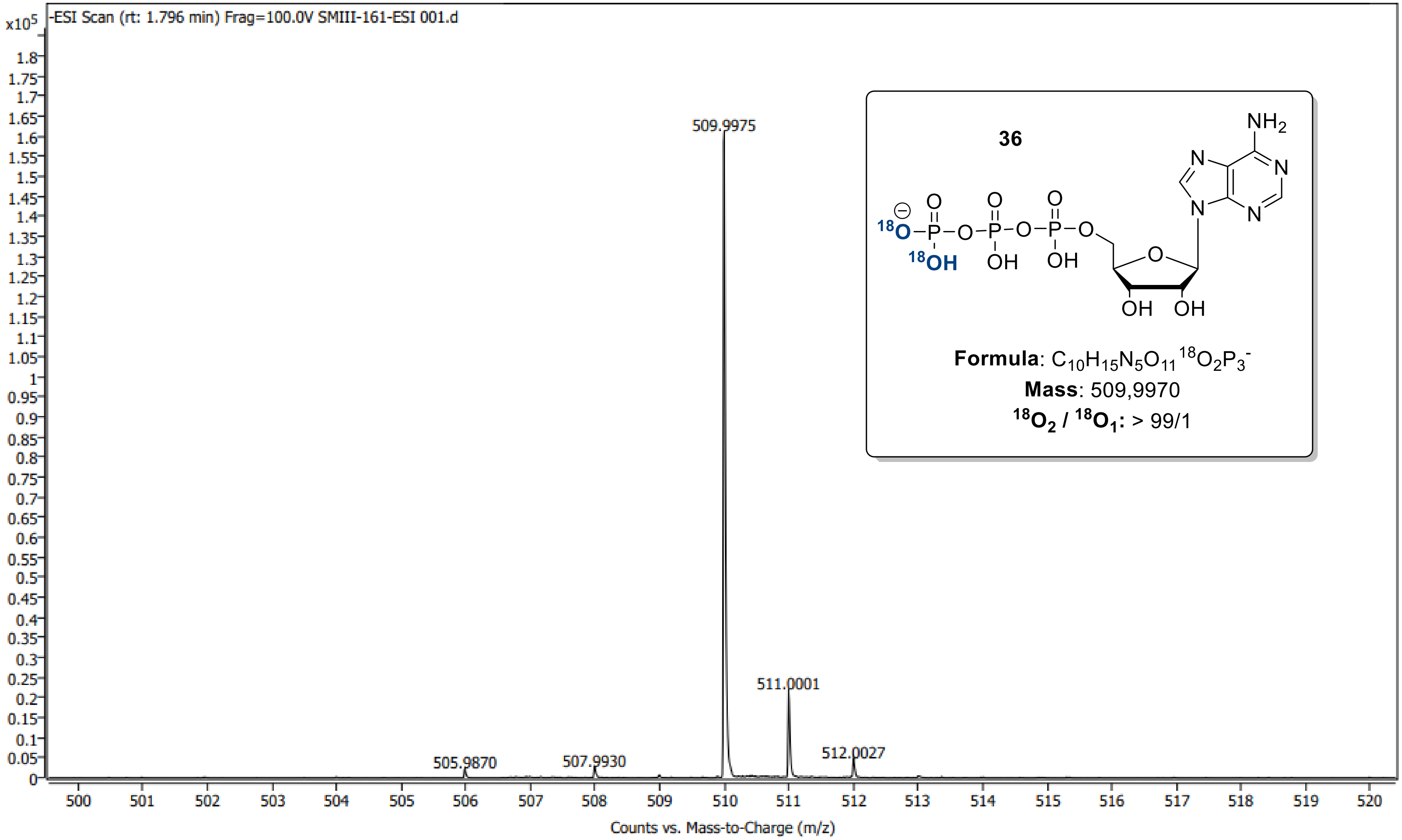

HRMS (ESI) Analysis of compound 37: γ-$^{18}O_2$-Guanosine-5'-triphosphate (γ-$^{18}O_2$-GTP)

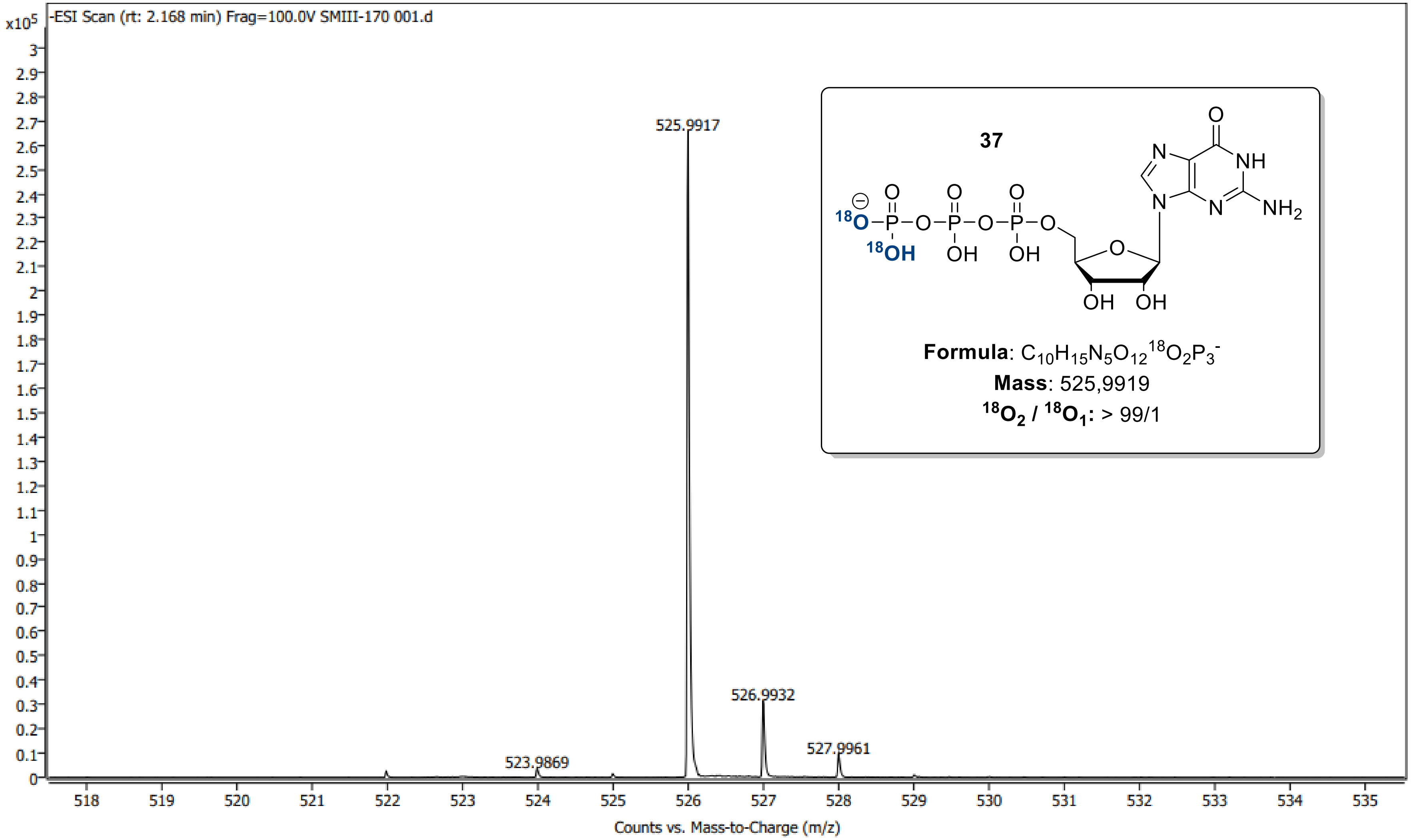

HRMS (ESI) Analysis of compound 38: γ-$^{18}O_2$-Uridine-5'-triphosphate (γ-$^{18}O_2$-UTP)

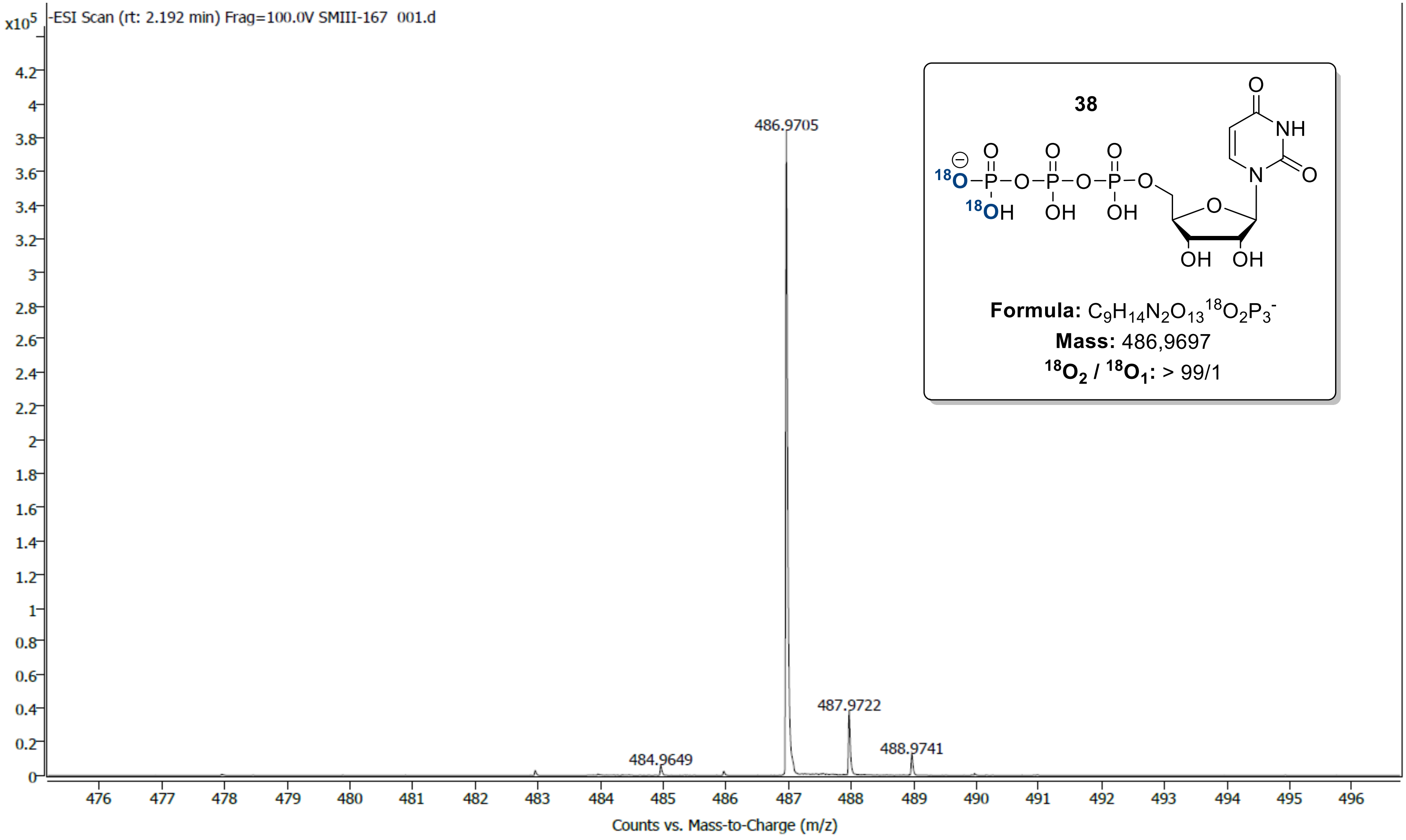

HRMS (ESI) Analysis of compound 39: ((2R,3S,4R,5R)-5-(6-amino-9H-purin-9-yl)-3,4-dihydroxytetrahydrofuran-2-yl)methyl tetraphosphate-$^{18}O_2$

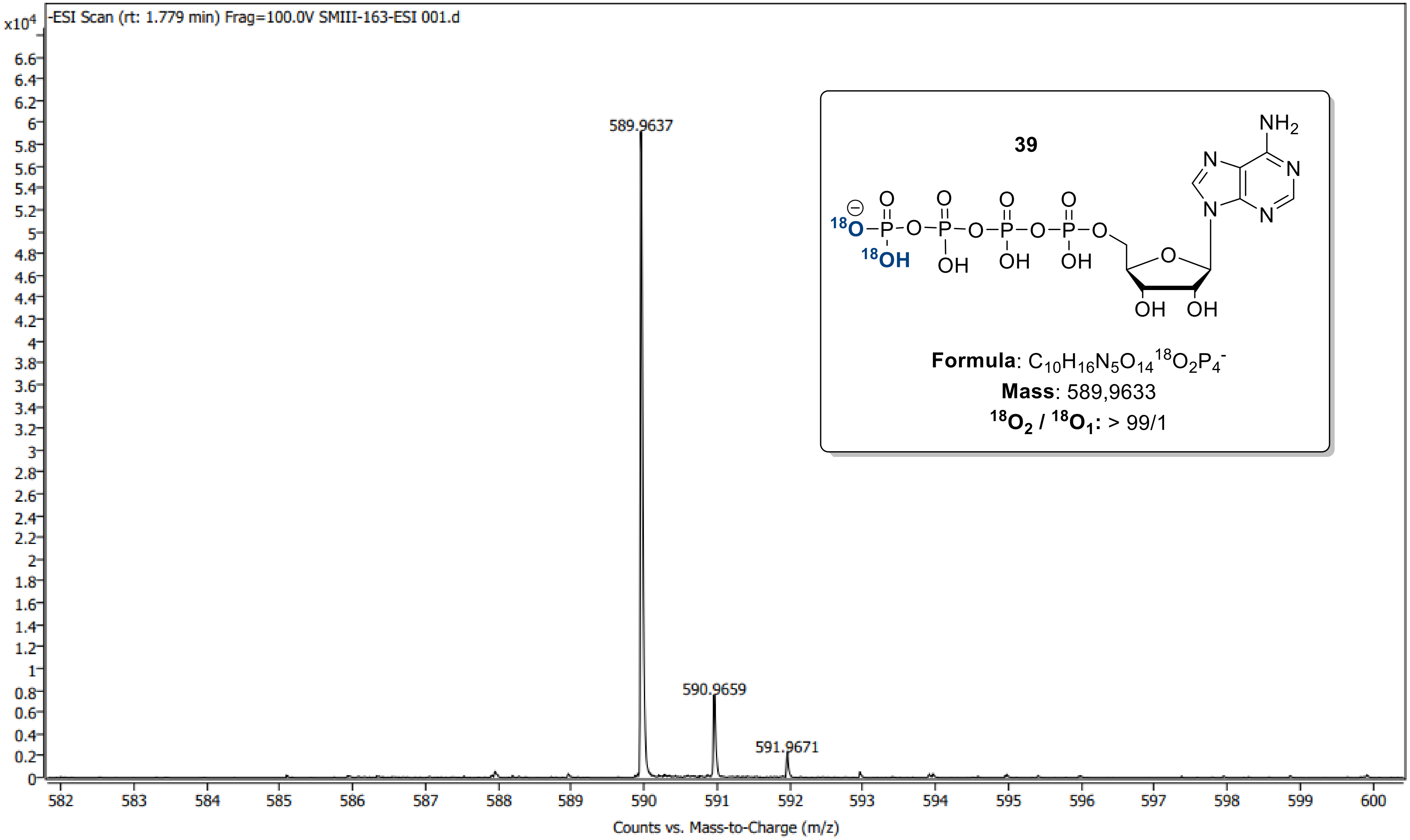

HRMS (ESI) Analysis of compound 40: ^{18}O-Diadenosinetriphosphate (β-^{18}O-Ap3A)

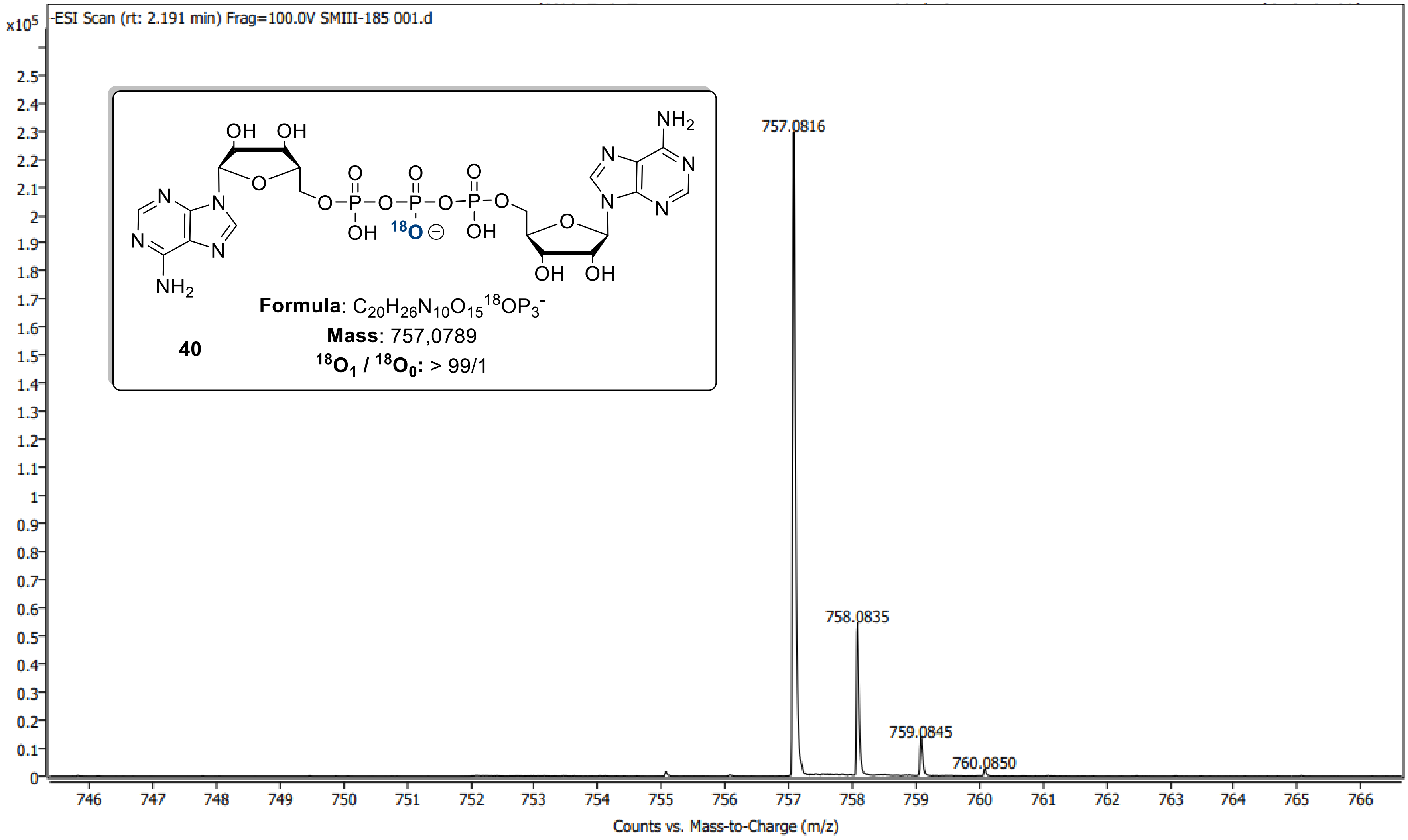

HRMS (ESI) Analysis of compound 54: 5'-$^{18}O_2$-Adenosine-3'-5'-bisphosphate (5'-$^{18}O_2$-pAp)

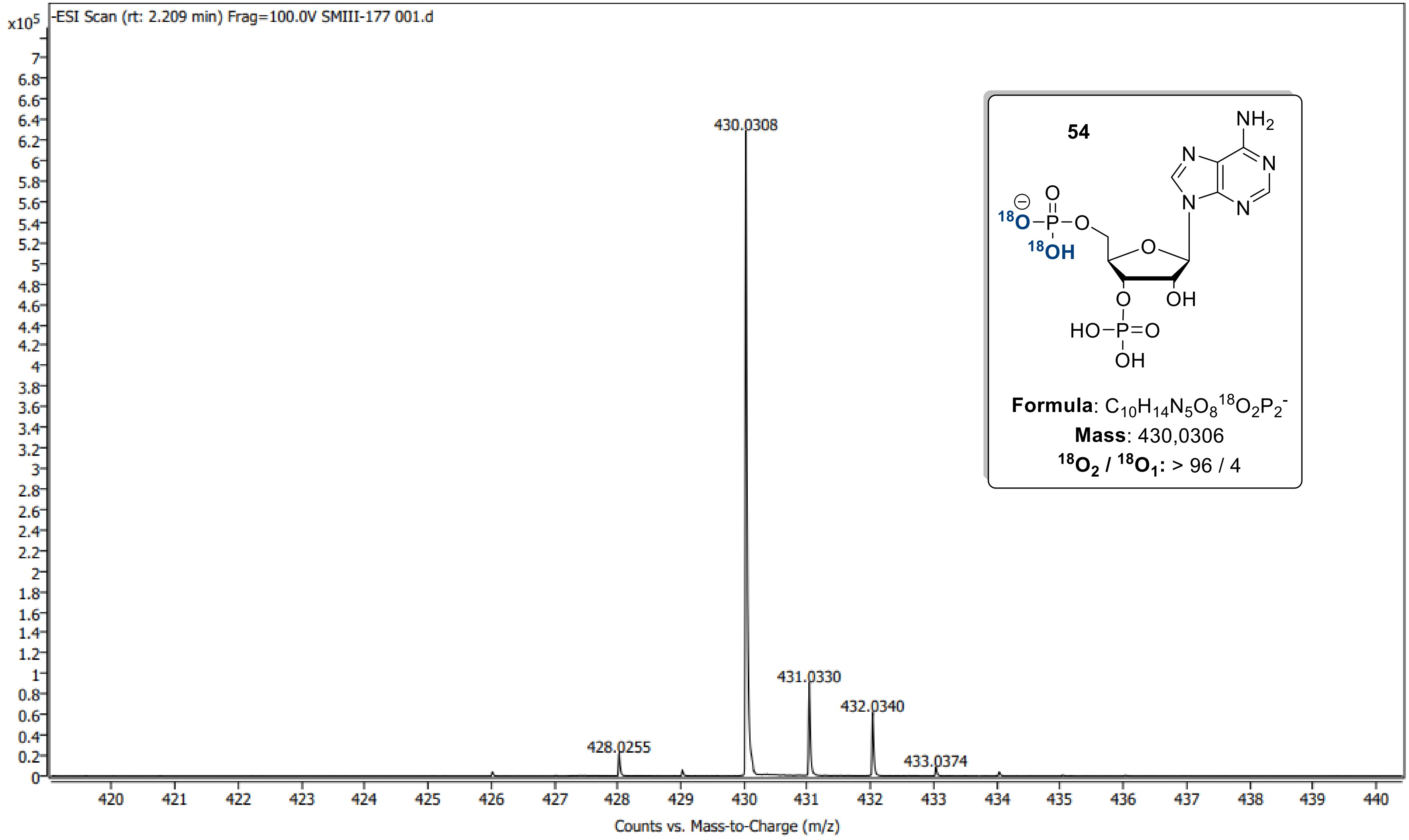

HRMS (ESI) Analysis of compound 41: Adenosine-3'-phosphat-5'-α-$^{18}O_2$-phosphosulfate (5'-$^{18}O_2$-PAPS)

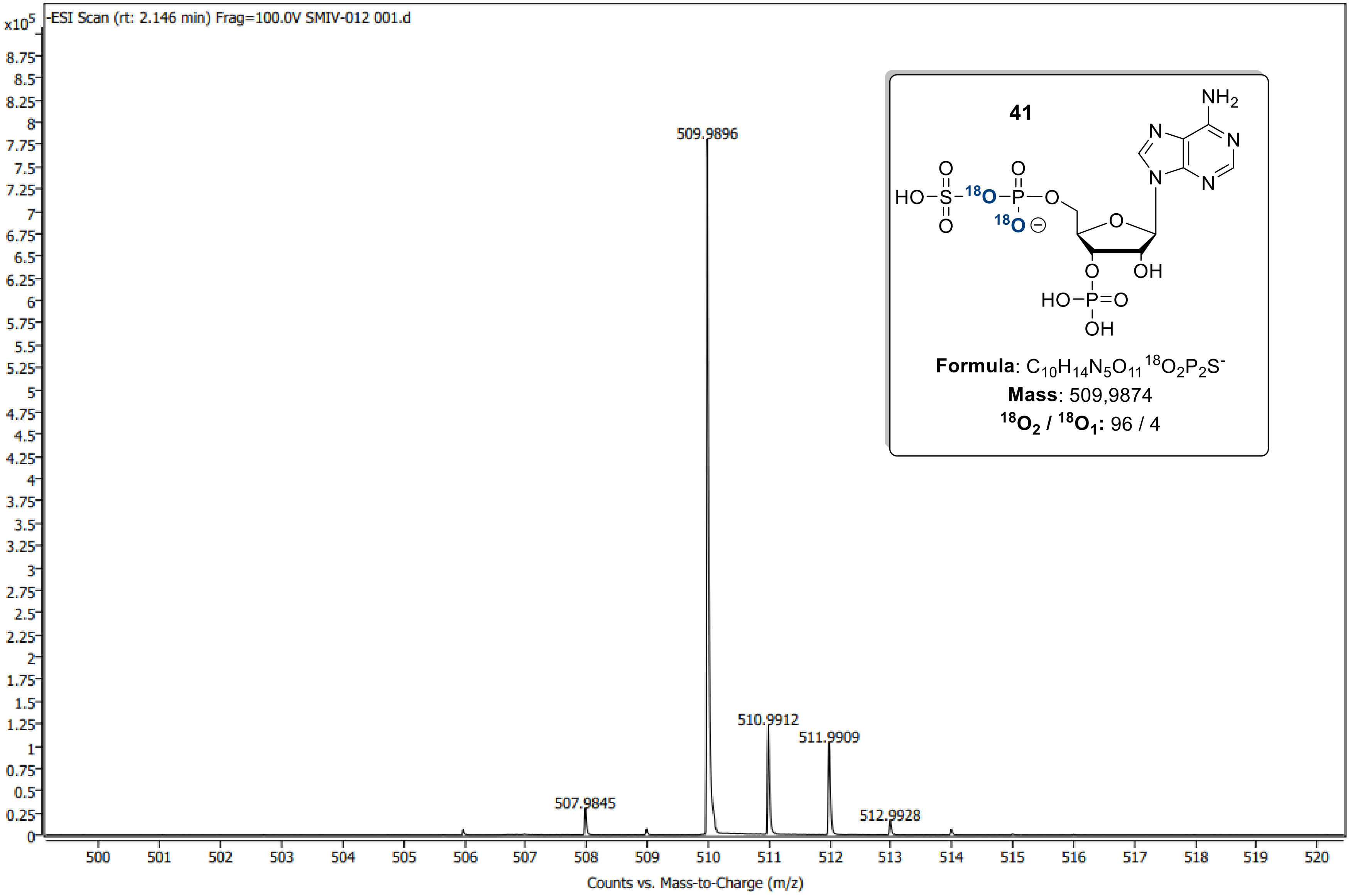

HRMS (ESI) Analysis of compound 42 $^{18}O_4$-Tetraphosphate ($^{18}O_4$–P4)

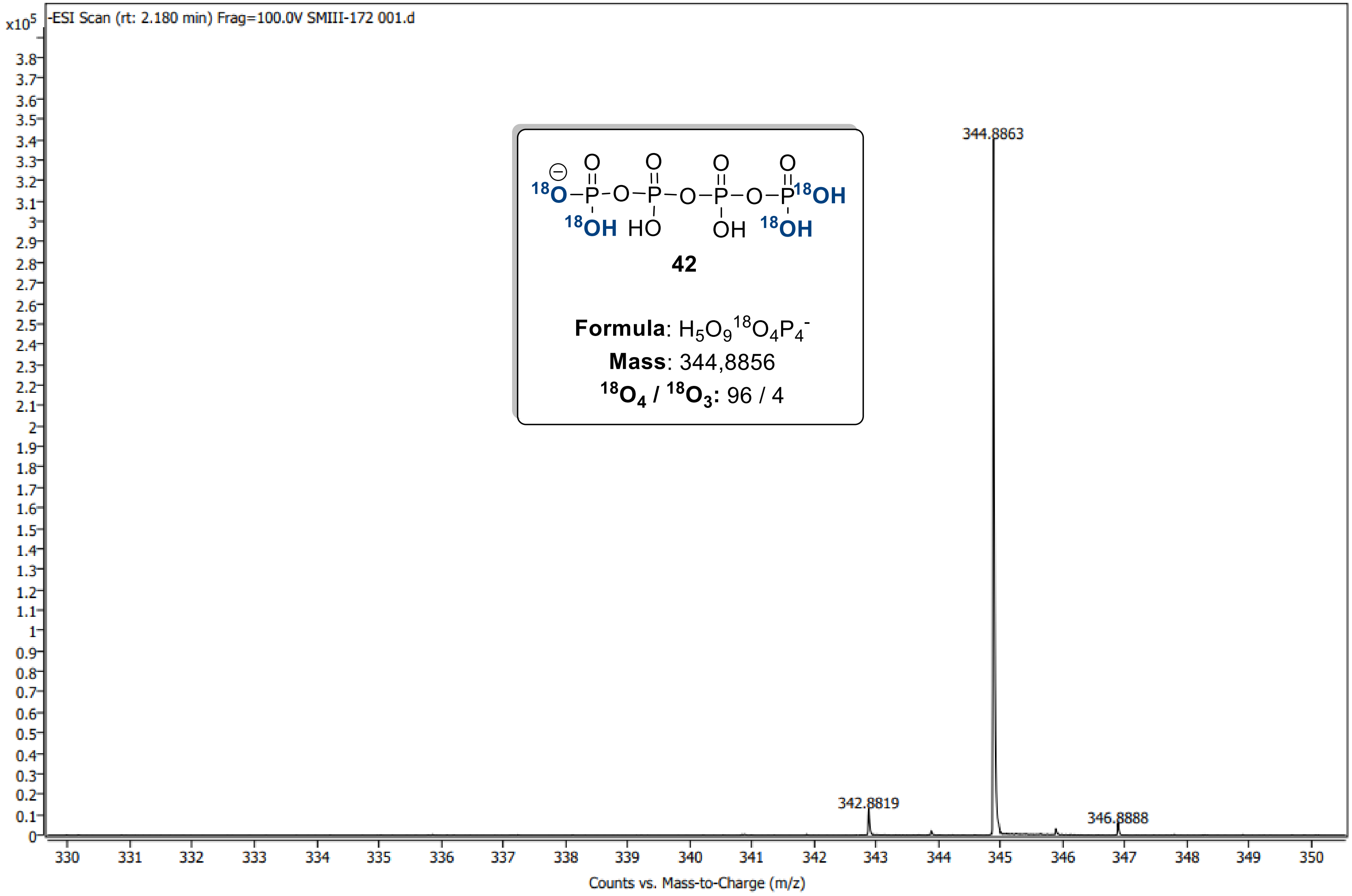

HRMS (ESI) Analysis of compound SI-4: Isoprenylmonophosphate

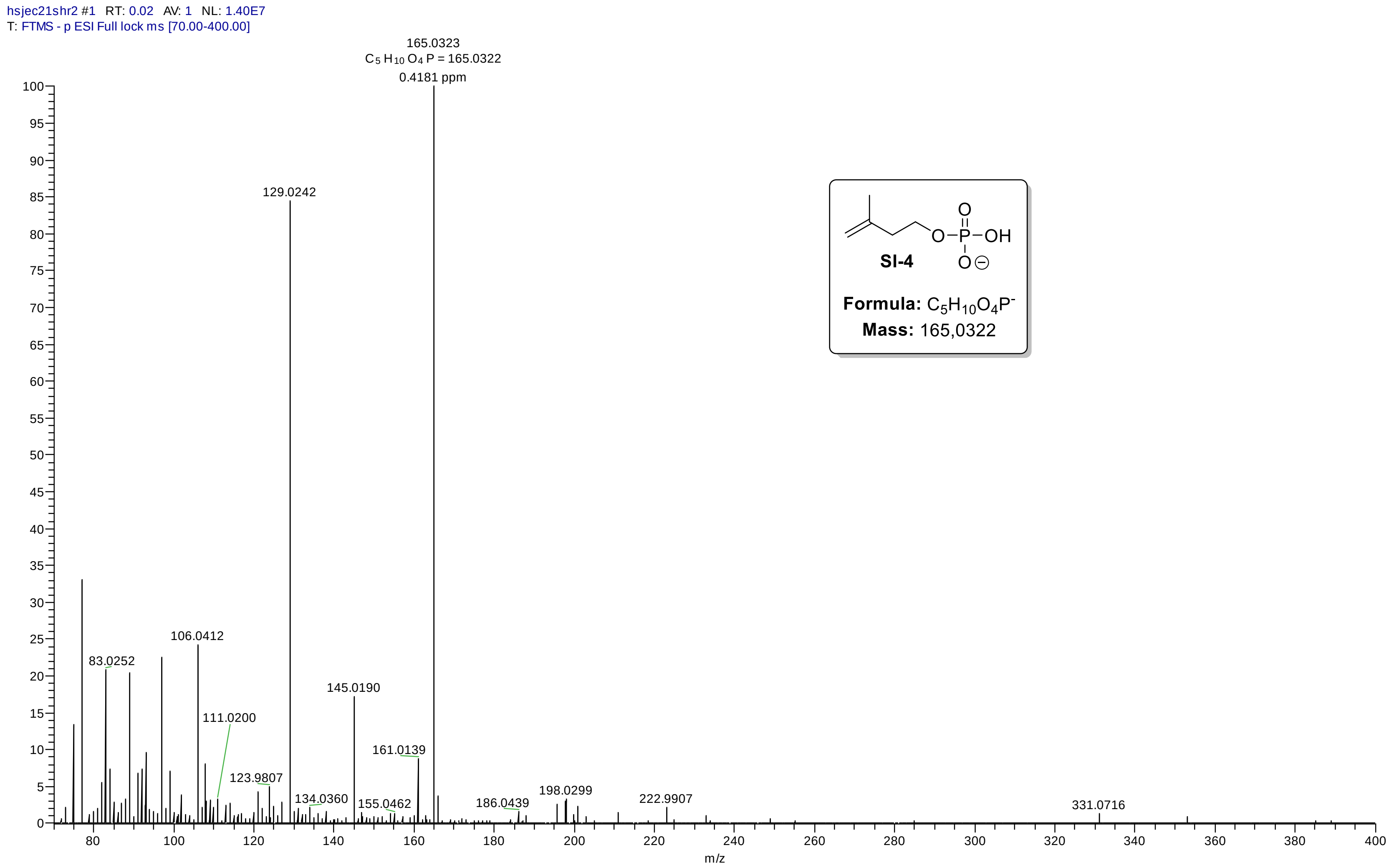

HRMS (ESI) Analysis of compound 43: β-$^{18}O_2$-Isoprenyl-diphosphate (β-$^{18}O_2$-IPP)

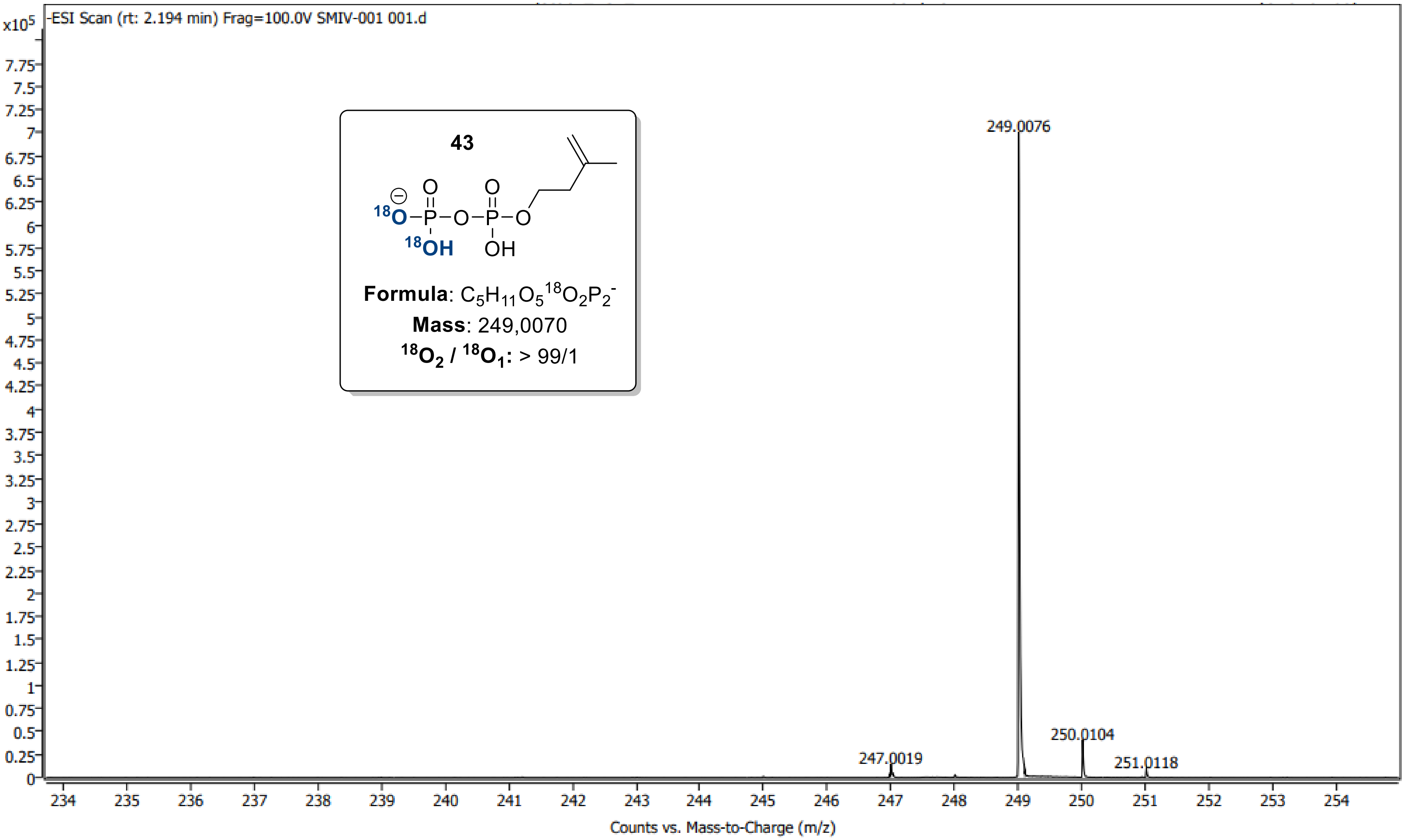

HRMS (ESI) Analysis of compound 44: Guanosine-3'-5'-bis(β-$^{18}O_2$-diphosphate) ($^{18}O_4$ - ppGpp)

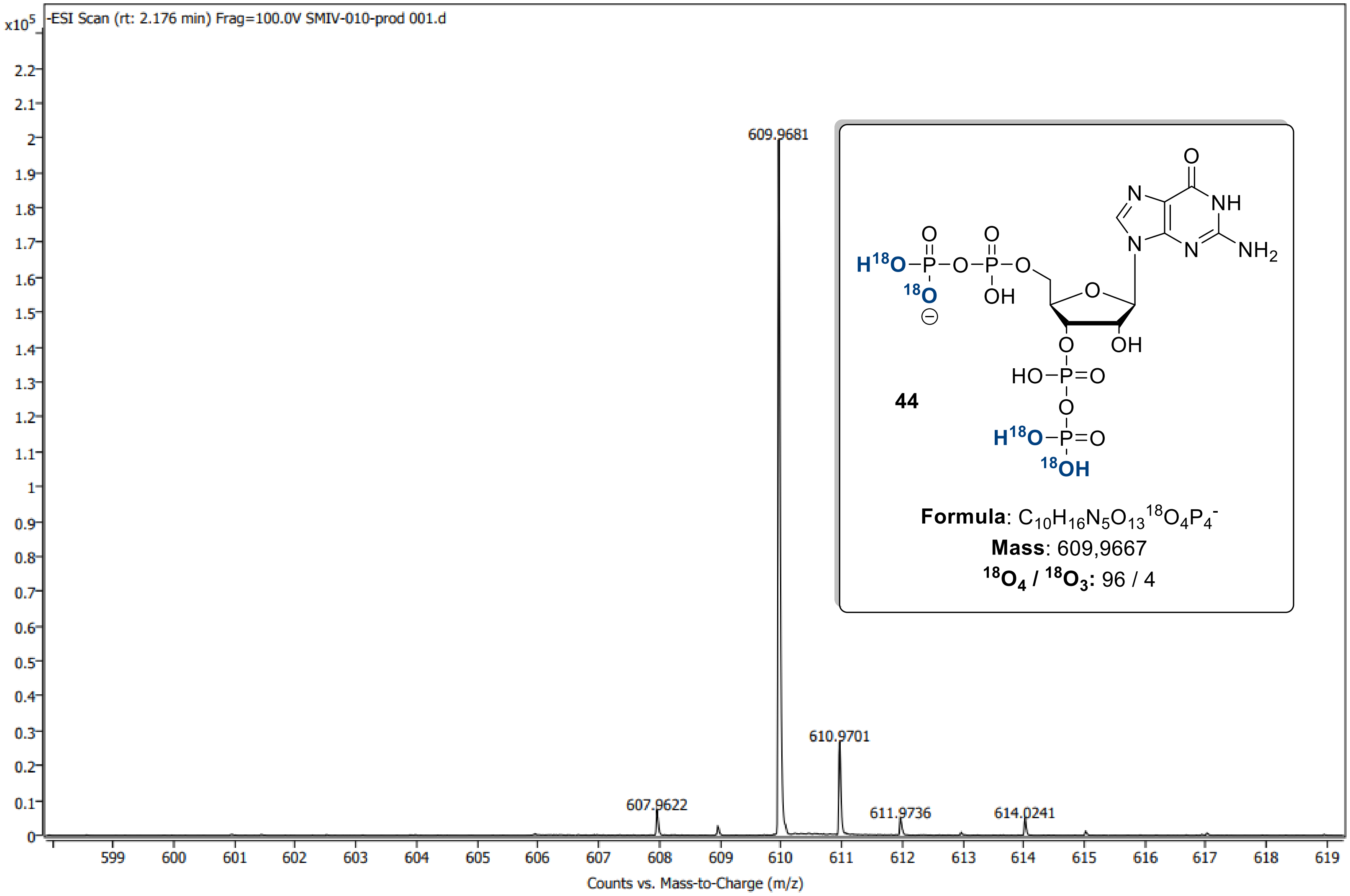

HRMS (ESI) Analysis of compound 45: Guanosine-3'-phosphate-5'-β-$^{18}O_2$-diphosphate ($^{18}O_2$-ppGp)

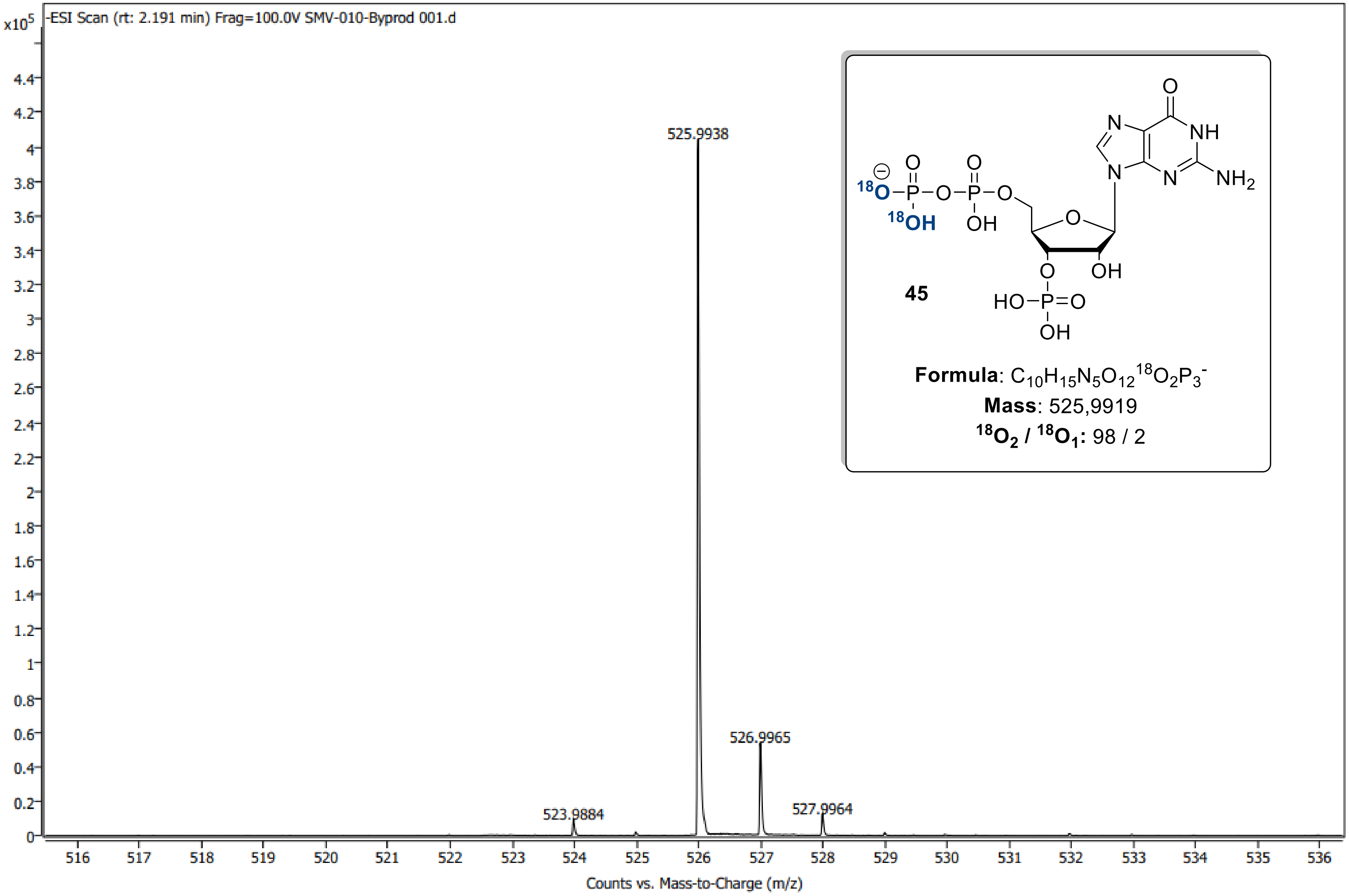

HRMS (ESI) Analysis of compound SI-7: AB-protected $^{18}O_{12}$ – myp-Inositolhexakisphosphate (AB_{12}-$^{18}O_{12}$-$InsP_6$)

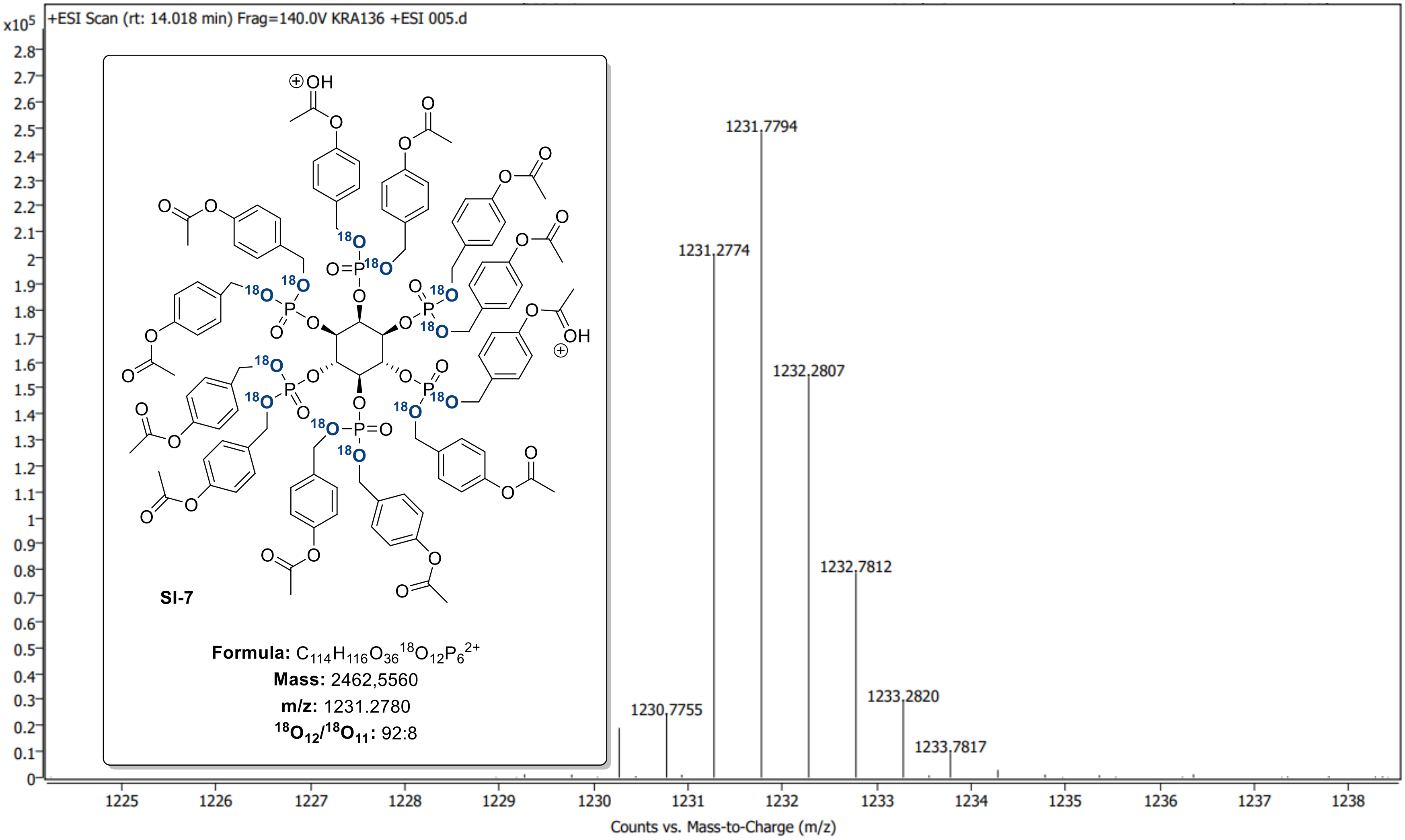

HRMS (ESI) Analysis of compound 46: $^{18}O_{12}$ – myo-Inositolhexakisphosphate ($^{18}O_{12}$-InsP$_6$)

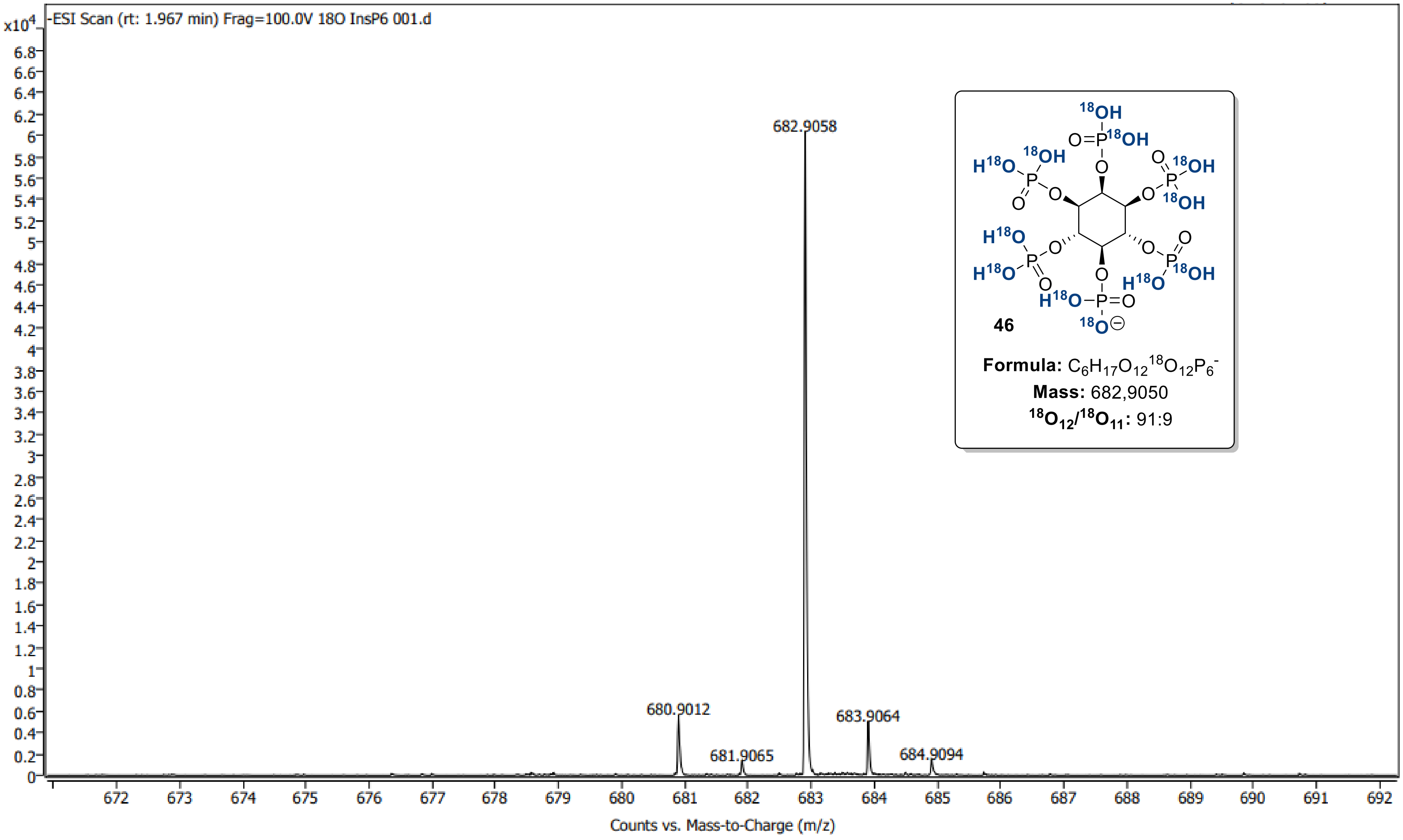

HRMS (ESI) Analysis of compound SI-9: AB$_{11}$-PMB-protected $^{18}O_2$- 5-Diphospho-inositol pentakisphosphate derivative

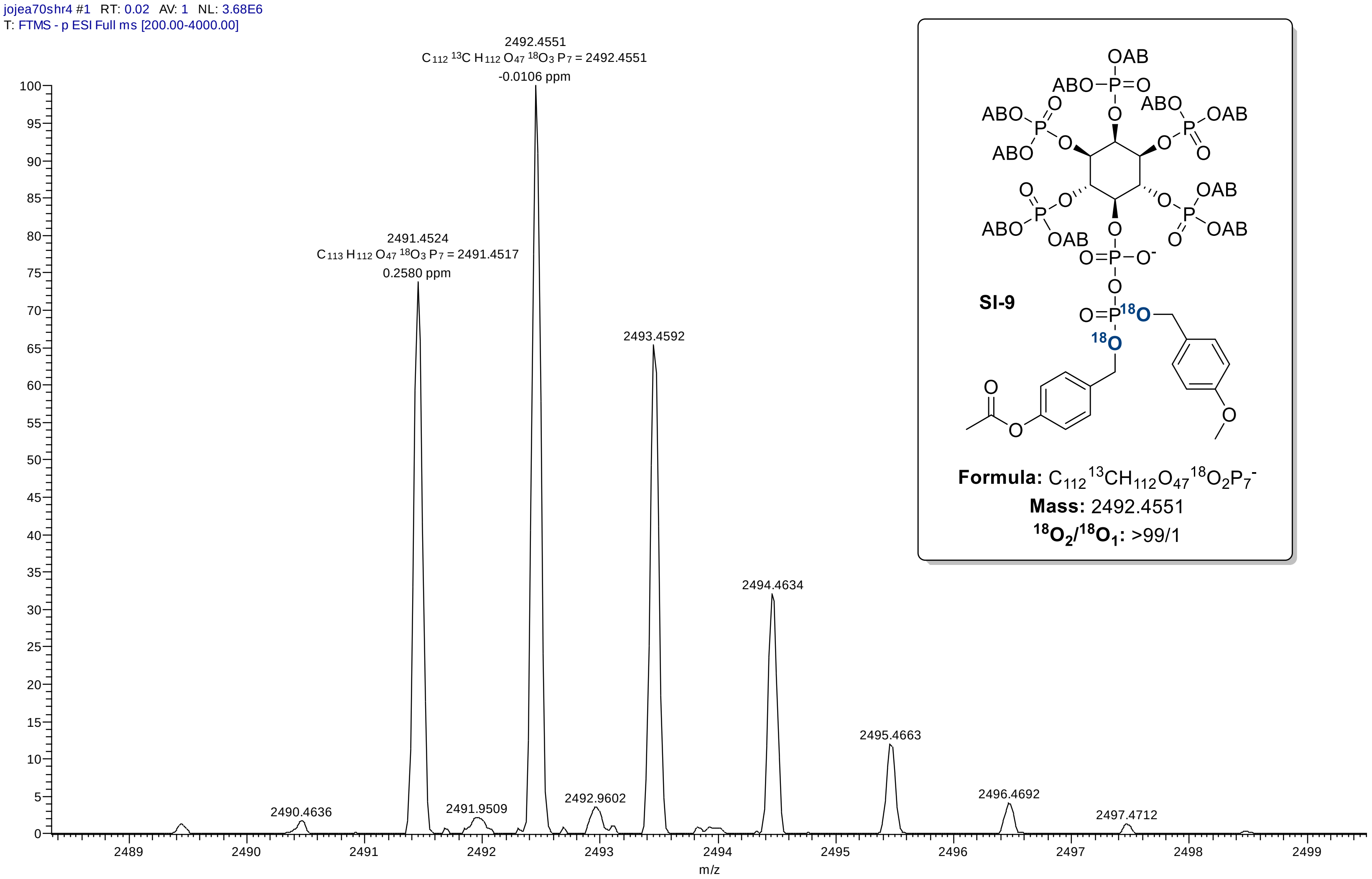

HRMS (ESI) Analysis of compound SI-10: AB$_{11}$-protected $^{18}O_2$- 5-Diphospho-inositol pentakisphosphate derivative

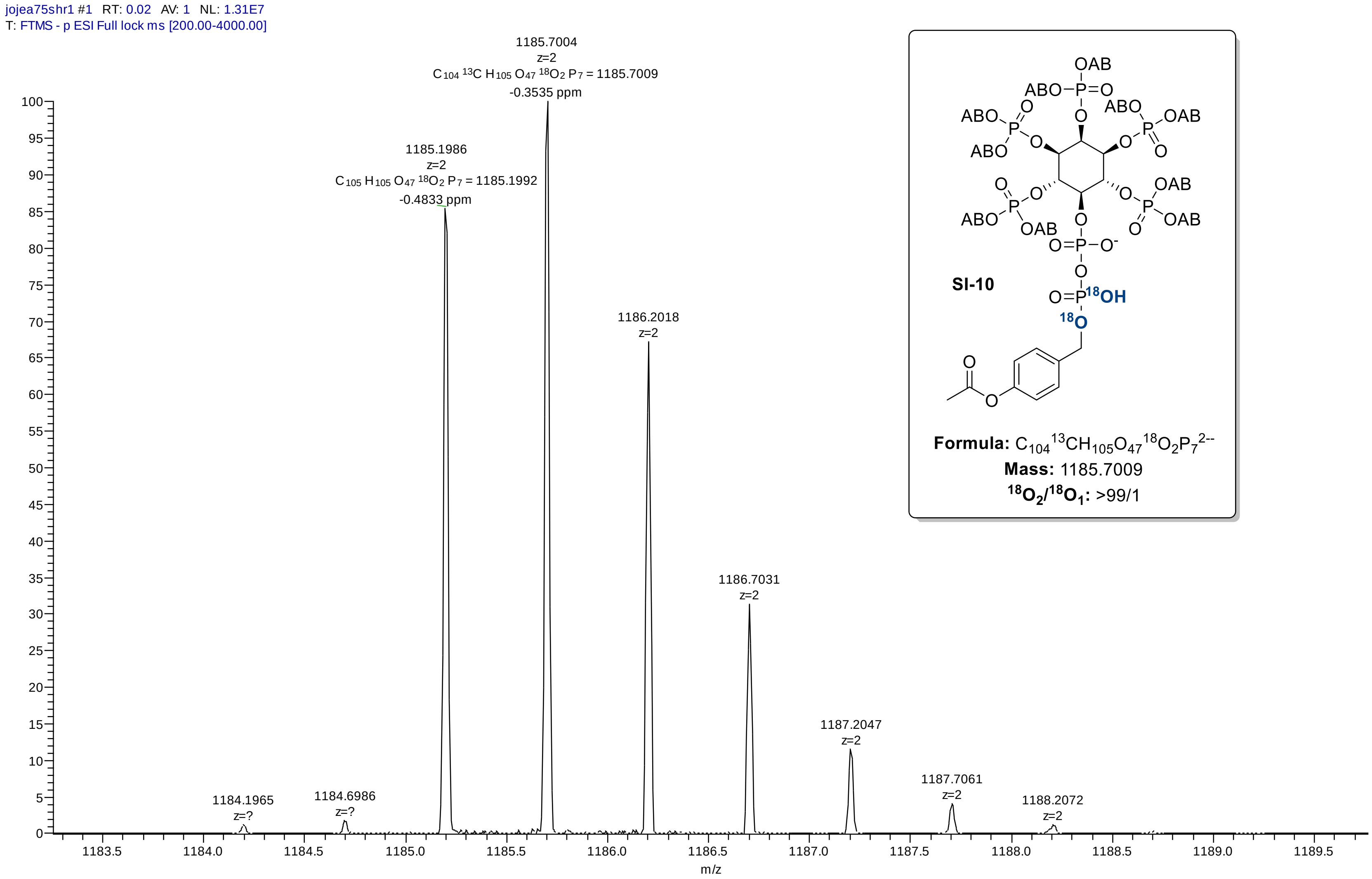

SI-10

Formula: $C_{104}{}^{13}CH_{105}O_{47}{}^{18}O_2P_7{}^{2-}$

Mass: 1185.7009

$^{18}O_2/^{18}O_1$: >99/1

HRMS (ESI) Analysis of compound 47: $^{18}O_2$- 5-Diphospho-inositol pentakisphosphate (β-$^{18}O_2$-5-InsP$_7$)

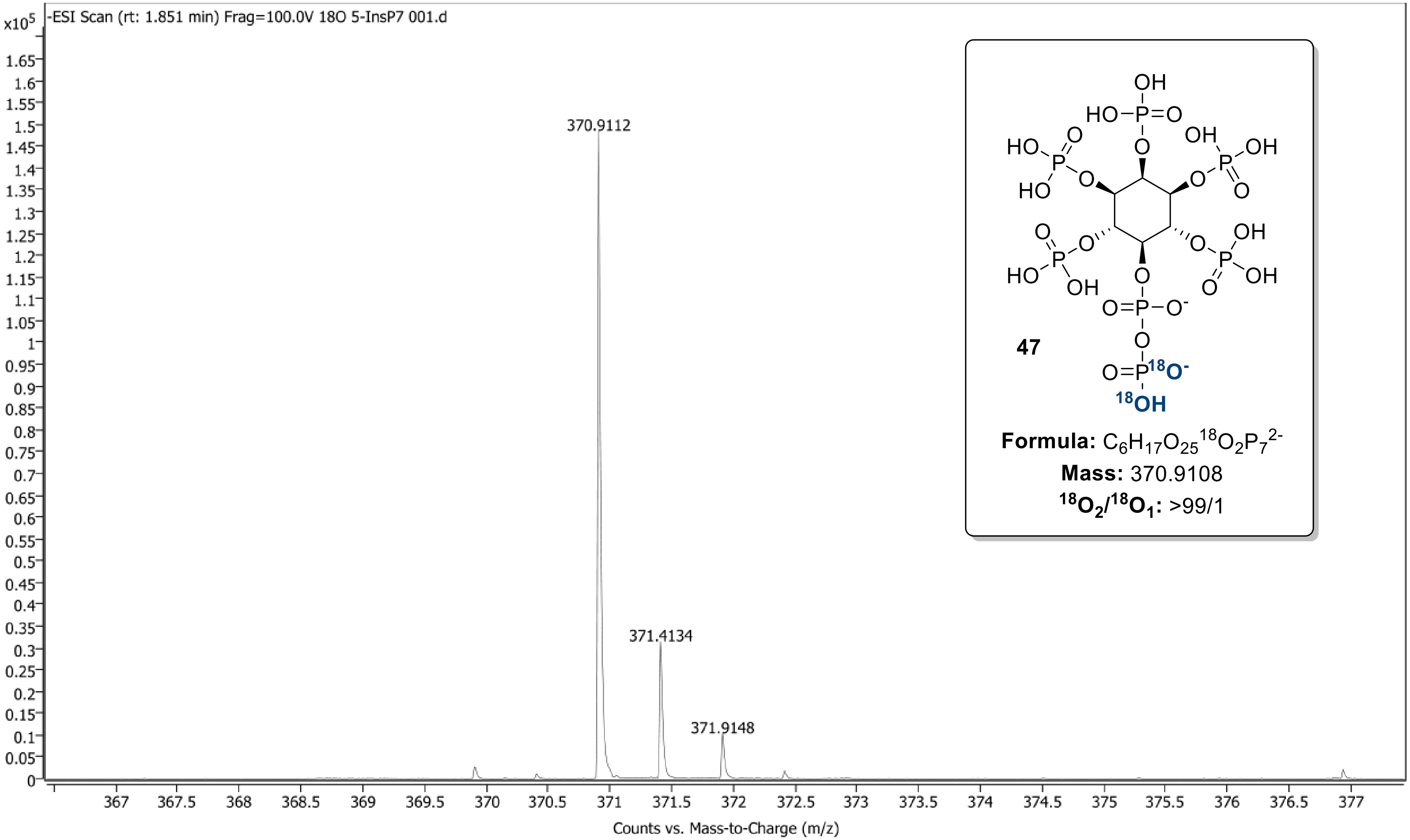

HRMS (ESI) Analysis of compound SI-12: o-Xylene/benzyl-protected β-$^{18}O_2$-5-InsP$_7$

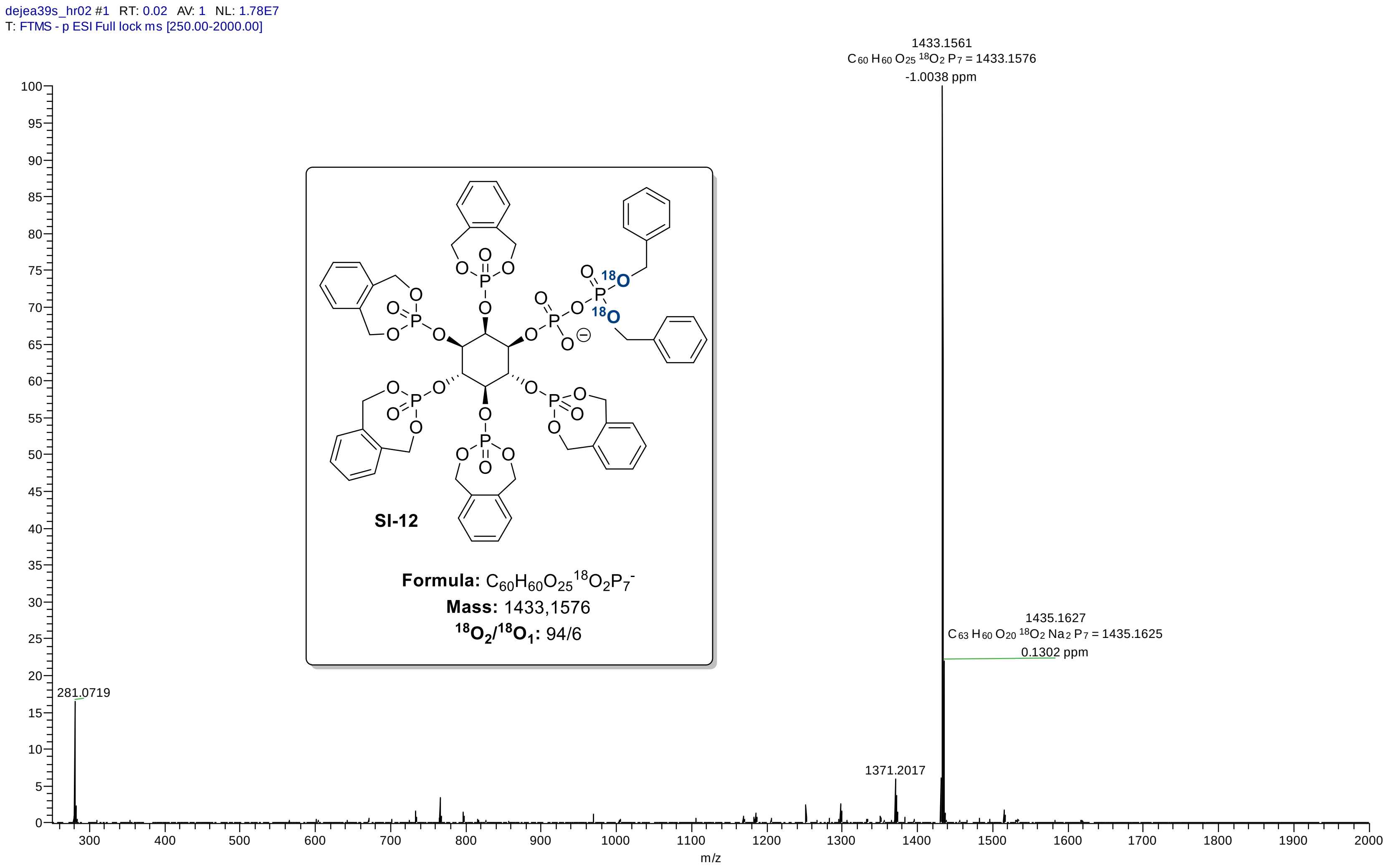

HRMS (ESI) Analysis of compound SI-13: β-$^{18}O_2$- 1-Diphospho-inositol pentakisphosphate (β-$^{18}O_2$-1-InsP$_7$)

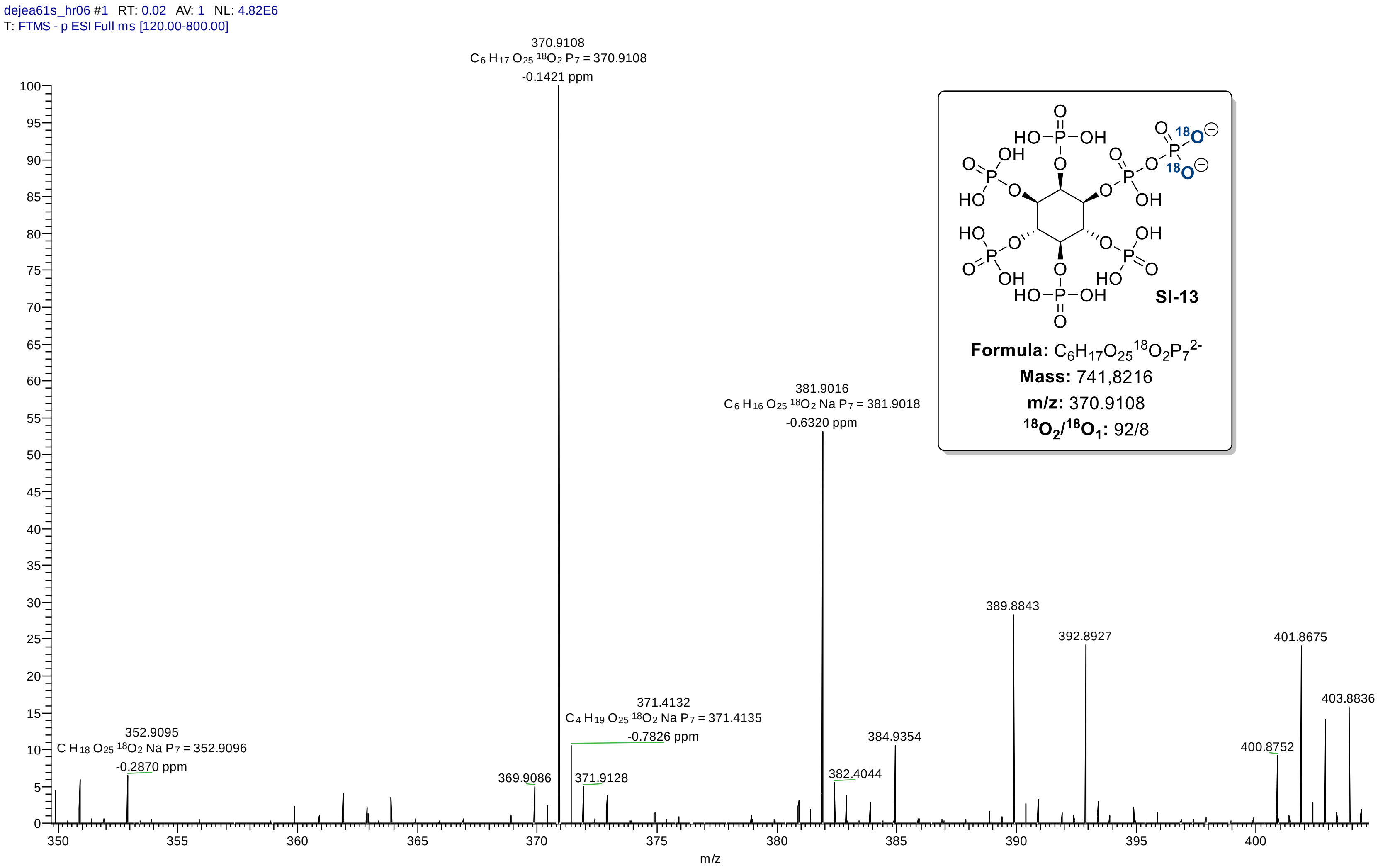

HRMS (ESI) Analysis of compound SI-16: DMT-5'-ATATATAT

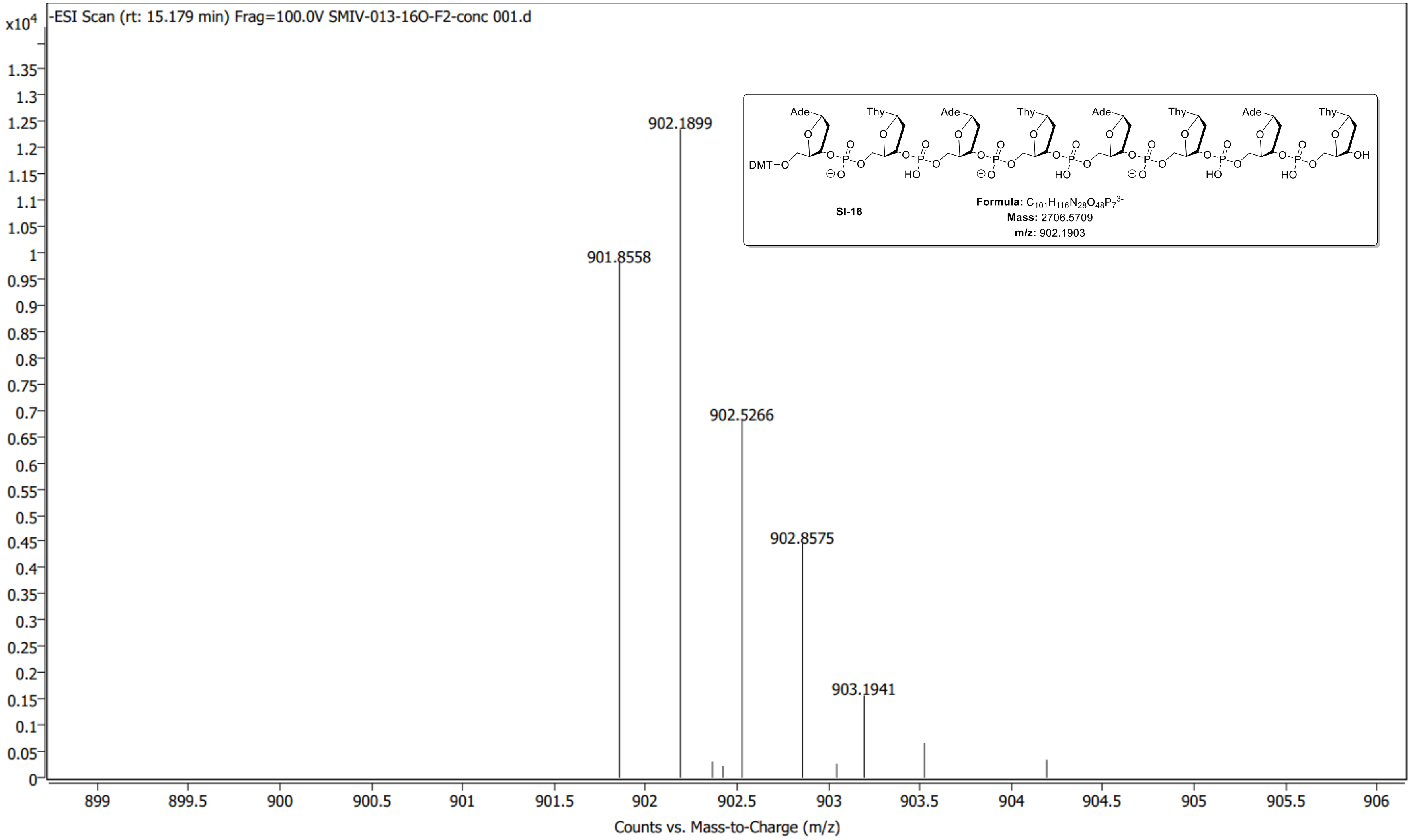

HRMS (ESI) Analysis of compound SI-17: $^{18}O_3$-DMT-5'-ATATATAT

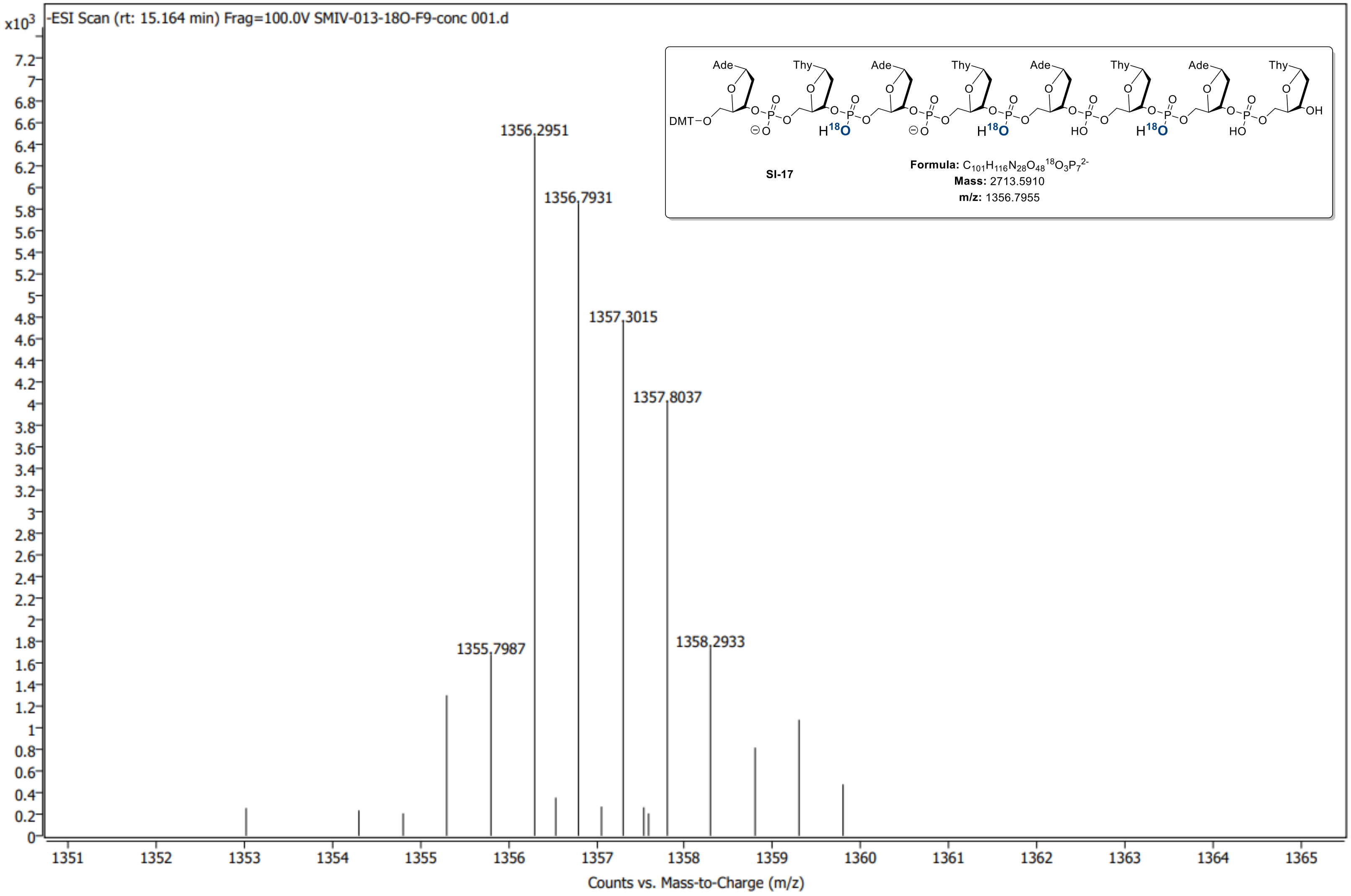

HRMS (ESI) Analysis of compound SI-18: 5'-ATATATAT

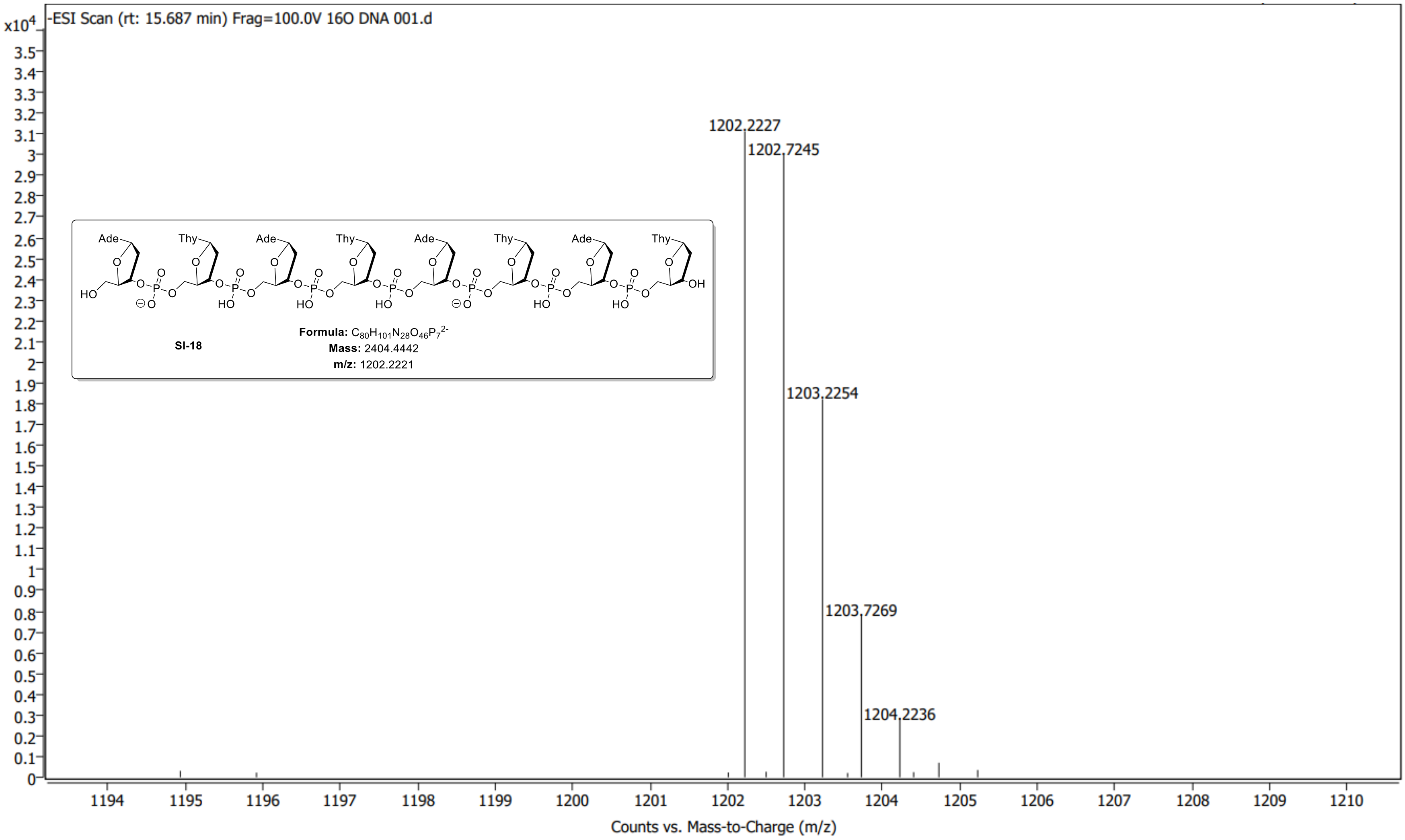

HRMS (ESI) Analysis of compound 48: $^{18}O_3$-5'-ATATATAT

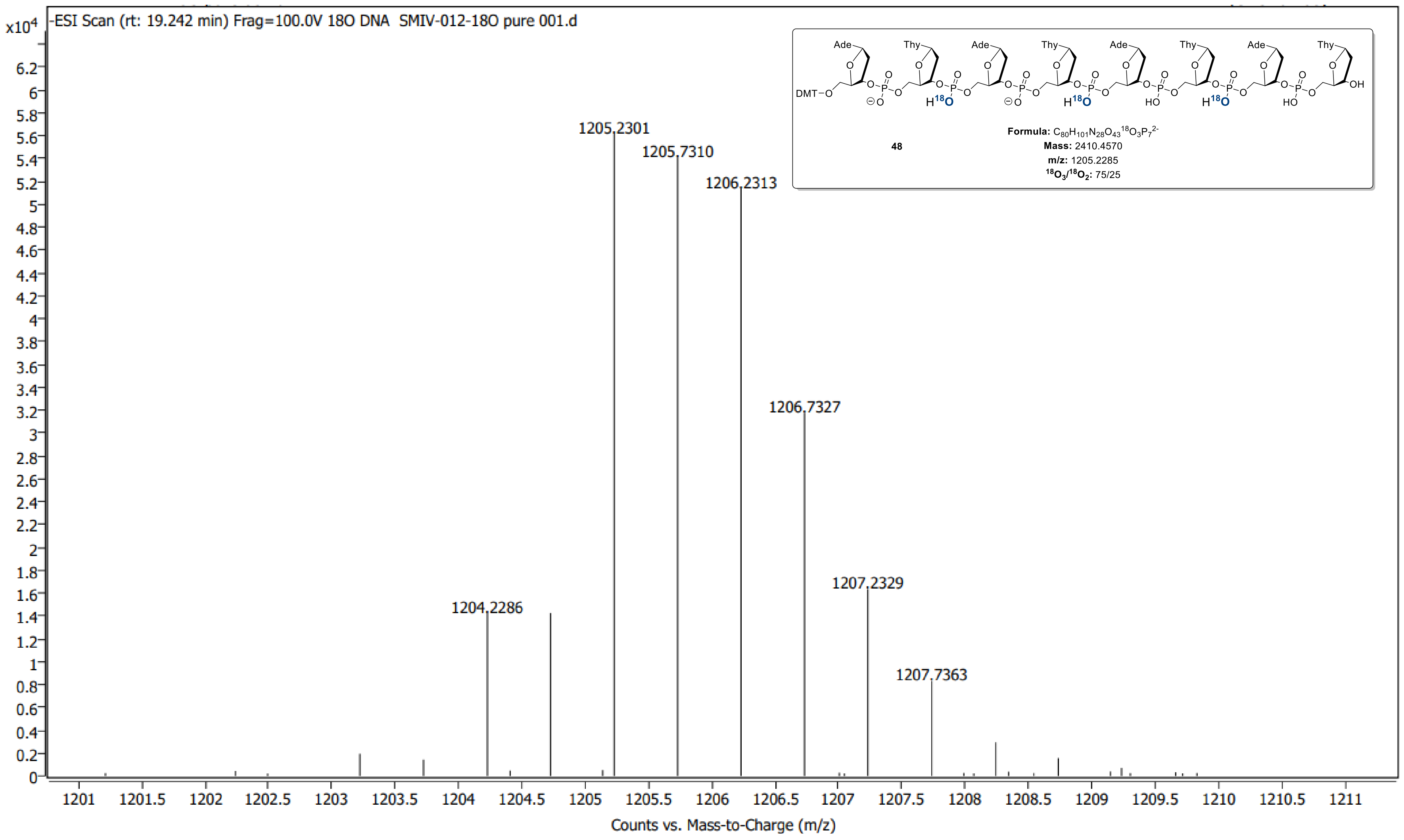

Supplement: Supplementary file 1 — Supporting Information [file ANIE-61-0-s001.pdf]
